# Anaerobic Hydroxylation of C(sp^3^)–H Bonds Enabled by the Synergistic Nature of Photoexcited Nitroarenes

**DOI:** 10.1021/jacs.2c13502

**Published:** 2023-01-25

**Authors:** Joshua M. Paolillo, Alana D. Duke, Emma S. Gogarnoiu, Dan E. Wise, Marvin Parasram

**Affiliations:** Department of Chemistry, New York University, New York, New York 10003, United States

## Abstract

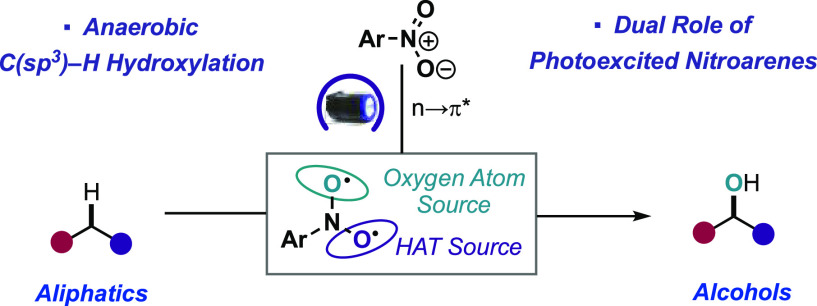

A photoexcited-nitroarene-mediated anaerobic C–H hydroxylation of aliphatic systems is reported. The success of this reaction is due to the bifunctional nature of the photoexcited nitroarene, which serves as the C–H bond activator and the oxygen atom source. Compared to previous methods, this approach is cost- and atom-economical due to the commercial availability of the nitroarene, the sole mediator of the reaction. Because of the anaerobic conditions of the transformation, a noteworthy expansion in substrate scope can be obtained compared to prior reports. Mechanistic studies support that the photoexcited nitroarenes engage in successive hydrogen atom transfer and radical recombination events with hydrocarbons, leading to *N*-arylhydroxylamine ether intermediates. Spontaneous fragmentation of these intermediates leads to the key oxygen atom transfer products.

The direct conversion of aliphatic C–H bonds to valuable alcohol groups represents a critical contemporary challenge in organic chemistry.^1^ The difficulty resides in selectively activating strong C(sp^3^)–H bonds and subsequently achieving efficient C–O bond formation without affecting oxidatively sensitive functional groups. The synthetic community has provided innovative solutions in pursuit of the installation of oxygen atoms on aliphatic scaffolds (Scheme 1). Direct oxidation of C–H bonds is commonly featured in batch-scale processes, but these typically employ harsh oxidizing conditions that restrict substrate scope.^2^ Furthermore, achieving site-selective C–H oxidative functionalization and preference for the alcohol over other overoxidation byproducts is arduous with this approach (Scheme 1A). Site-selectivity challenges have been elegantly addressed with the use of directing groups in transition-metal-catalyzed C–H hydroxylation reactions.^3^ However, many of these strategies require nonremovable directing groups and precious metal catalysts that contribute to high costs in industrial processes (Scheme 1B).^4^ Biomimetic Mn/Fe-catalyzed and/or enzyme-catalyzed C–H hydroxylation reactions have recently emerged as powerful alternatives to precious metal approaches (Scheme 1C).^5^ However, low reaction efficiency, concerns with overoxidation, the high cost of ligands, and the cost of engineering enzymes deter widespread implementation. Markedly, the use of additional oxidants is required for all three of these approaches, which further limits the reaction scope and synthetic utility of these methods. Herein we report a metal-free C–H hydroxylation of aliphatic systems promoted by photoexcited nitroarenes (Scheme 1D). Notably, the biradical nature of the photoexcited nitroarenes enables both the C–H activation step and the oxygen atom transfer step, obviating the need for additional oxidants and providing a mild, general, and cost-effective means for C(sp^3^)–H hydroxylation.

**Scheme 1:**
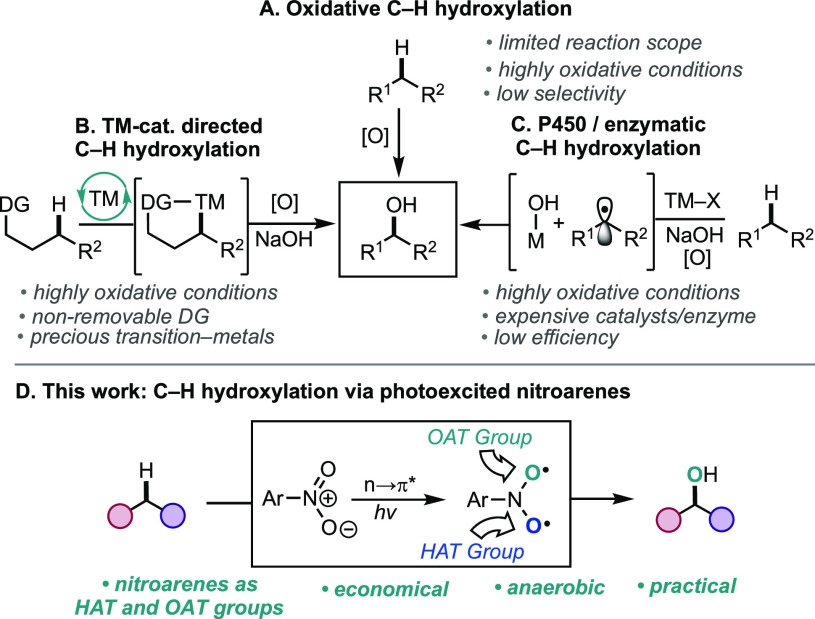
C–H Hydroxylation Approaches

Contemporaneous reports from our laboratory^6^ and Leonori’s group^7^ illustrate that visible-light excitation of nitroarenes leads to a triplet biradical intermediate, which enables the cleavage of alkenes into carbonyl derivatives. Mechanistic studies by Döpp,^8^ our group,^6^ and others^9^ have showcased that the aforementioned triplet biradical intermediate is capable of C–H bond activation via intramolecular hydrogen atom transfer (HAT) with *o*-alkyl groups of nitroarenes. Seminal works from the groups of Hamilton,^10^ Severin,^11^ and Berman^12^ provide evidence that C–H oxidation can be achieved via oxygen atom transfer (OAT) from nitroarenes under harsh UV irradiation. Recently, Cao, Lu, and Yan disclosed that photoexcited β-aryl-substituted nitroarenes can trigger an intramolecular OAT event leading to tertiary diaryl alcohols.^13^ Although both approaches are of significant novelty, they suffer from limited reaction scope and issues with overoxidation. Based on the capability of photogenerated nitroarenes to serve as the C–H bond activator and the oxygen atom source, we questioned whether a selective, intermolecular, anaerobic C–H hydroxylation of aliphatic precursors could be achieved under visible-light irradiation.

To test this hypothesis, we investigated the reaction outcome for the hydroxylation of benzylic and unactivated C–H bonds with indane and **1ag**, respectively, in the presence of electron-deficient nitroarenes under 390 nm photoirradiation (see the Supporting Information (SI)). After an extensive optimization campaign, a few conclusions could be drawn from the benchmark studies: (1) 2-chloro-4-nitropyridine and 3,5-bis(trifluoromethyl)nitrobenzene were highly efficient for C–H hydroxylation of benzylic and unactivated C–H bonds, respectively; (2) the use of hexafluoroisopropanol (HFIP) as an additive was critical in suppressing overoxidation of the formed C–H hydroxylation product, presumably through hydrogen-bonding interactions;^14^ (3) control studies indicated that light and the other reaction components are necessary for the transformation.

With the optimized reaction conditions established, we first examined the scope of benzylic C–H hydroxylation using conditions A (Table 1). It was found that cyclic benzylic compounds of various ring sizes performed well under the reaction conditions (**2a**–**c**). Alcohols **2a** and **2b** were both isolated in high yields with good selectivity for the alcohol in comparison to a previously reported metal-free C–H oxidation method that favors the formation of the ketone overoxidation products.^15^ Substituted indanes featuring a Boc-protected amine (**2d**) and a triflate (**2e**) were tolerated under the reaction conditions, affording the hydroxylated products in moderate yields. Celestolide (**1f**), a valuable molecule in flavors and fragrances, was successfully hydroxylated, resulting in a 57% yield of the alcohol product (**2f**). Next, the scope of ethylbenzenes was explored. Electron-rich and neutral substrates performed well, resulting in good yields of the alcohol products (**2g**, **2h**). Substrates with electron-withdrawing groups (**2j**, **2k**) and halogens (**2l**, **2m**) were also amenable to reaction conditions, albeit with slightly lower yields. It is worth mentioning that halogen substituents have previously been unsuitable in C(sp^3^)–H oxidation reactions, as in a literature report that obtained the ketone analogue of **2l** in 19% compared to our selective hydroxylation in 50% yield.^16^ The reaction of ethylbenzene substituted with a boronic pinacol ester (**1n**), an oxidatively sensitive functional group used in cross-coupling chemistry, successfully afforded the hydroxylated product (**2n**) in 44% yield. While toluene derivatives (**2o**, **2p**) were successfully hydroxylated to the corresponding benzyl alcohols in moderate yields, a higher equivalence of HFIP was required to prevent overoxidation to the corresponding aldehydes. In substrates containing multiple equivalent benzylic sites (**2q**, **2r**), the reaction selectively produced the monohydroxylated product. For substrates containing asymmetric benzylic positions, the reaction was selective for secondary oxidation over primary (**2s**), secondary oxidation over tertiary (**2t**), and primary oxidation over tertiary (**2u**), giving an overall reactivity profile of secondary > primary > tertiary for benzylic C(sp^3^)–H oxidation. Other secondary benzylic substrates of various chain lengths and functional groups (**1v**–**y**) were tested under the reaction conditions and successfully afforded the hydroxylated products (**2v**–**y**). Notably, a free hydroxyl (**1w**) and a carboxylic acid (**1x**) were tolerated and afforded the corresponding diol (**2w**) and lactone (**2x**) products, respectively. Additionally, benzylcyclopropane (**1y**) was hydroxylated (**2y**) in 62% yield with no ring-opening products detected.^17^ Next, we examined the synthetic utility of this method for the hydroxylation of medicinally relevant and bioactive compounds with benzylic sites (**1z**, **1aa**, **1ab**), all of which performed well under the reaction conditions. Specifically, ibuprofen derivative **1z** was hydroxylated to give a 77% yield of **2z**, representing a slightly higher efficiency in comparison to the reported P450-catalyzed C–H hydroxylation (72%).^18^ Despite the successes in functional group tolerance of this protocol, heterocycles were an unsuccessful class of substrates, with no conversion of starting material detected.^19^

**Table 1:**
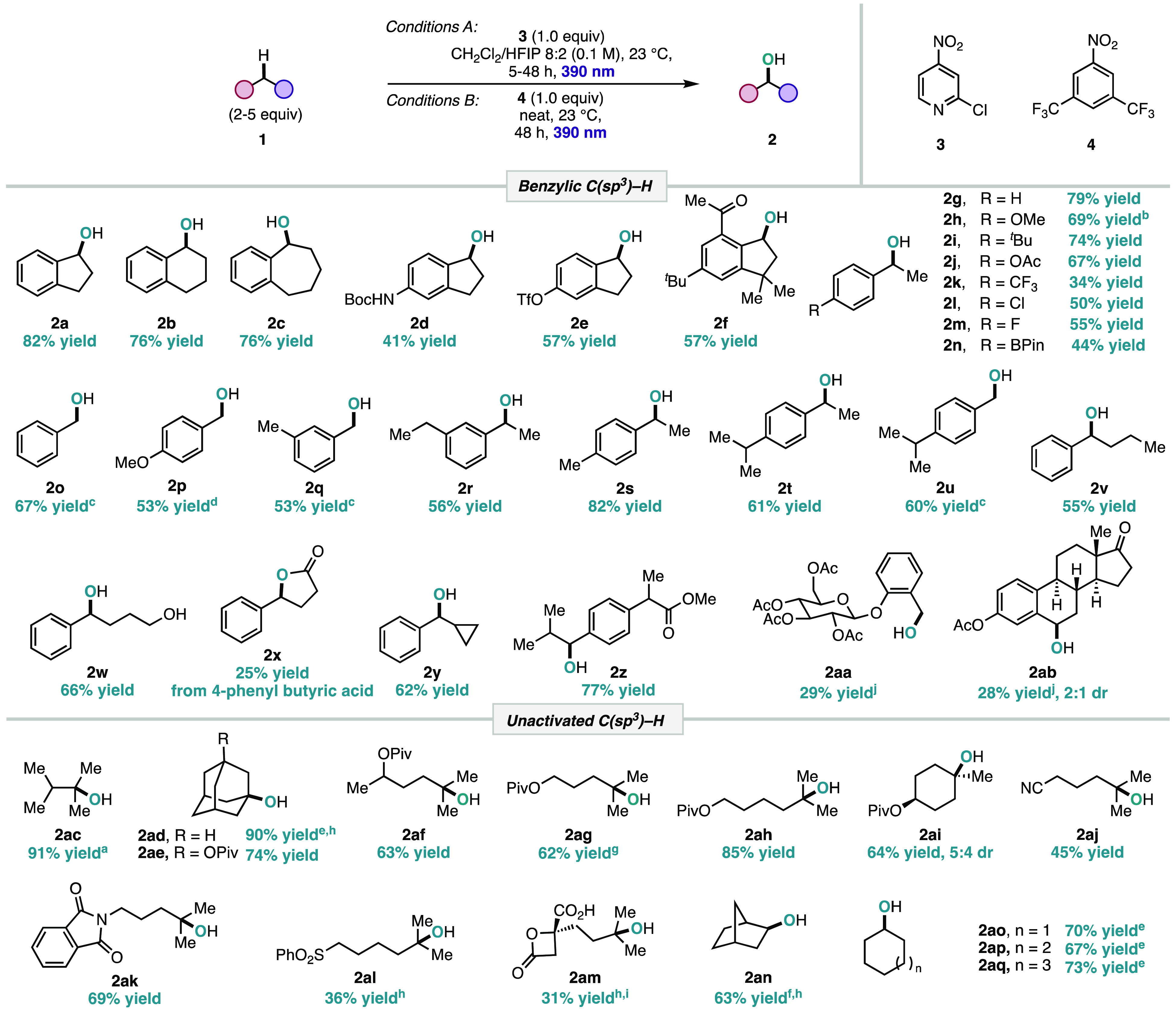
Scope of the Photoinduced Nitroarene-Promoted C(sp^3^)–H Hydroxylation^k^

a ^1^H NMR yield using CH_2_Br_2_ as an external standard.

b 50% light intensity.

c HFIP as the solvent

d Reductive workup.

e 2 equiv of HFIP.

f 4 equiv of HFIP.

g Conducted on gram scale.

h 1.0 M in CH_2_Cl_2_.

i Yield after 72 h.

j Average of two ^1^H NMR yields using CH_2_Br_2_ and 1,3,5-trimethoxybenzene as external standards.

k Isolated yields are reported, unless otherwise noted.

Next, we analyzed the scope of the C–H hydroxylation of unactivated C–H bonds using conditions B. We started by investigating weaker 3° C–H bonds in the context of unactivated systems (**2ac**, **2ad**), which were hydroxylated in good to excellent yields. The reaction conditions were then successfully translated to other 3° C(sp^3^)–H bonds. Substrates containing distal pivalate groups underwent smooth and selective 3° C–H hydroxylation in good yields (**2af**–**ai**). This matches the selectivity pattern seen in C–H hydroxylations of alkanes reported in the literature, whereby polar deactivating groups reduce unwanted oxidation at proximal positions.^20^ Various sensitive polar groups such as nitrile (**1aj**), phthalimide (**1ak**), and sulfonyl (**1al**) were tolerated under the reaction conditions and resulted in selective hydroxylation at the tertiary position (**2aj**–**al**). This selectivity pattern was leveraged to demonstrate the applicability of our method in the synthesis of bioactive molecules. Alcohol **2am**, a direct precursor to harringtonine, a natural product with anticancer activity,^21^ was successfully synthesized from the anticancer precursor to deoxyharringtonine (**1am**) in 31% yield after 72 h, despite the presence of the deactivating group proximal to the tertiary position. This result indicates the applicability of this method to late-stage functionalization of complex molecules. We then extended our reaction conditions to the C–H hydroxylation of challenging secondary C–H bonds. Direct hydroxylation of secondary C–H bond sites on simple hydrocarbons was achieved under the reaction conditions, resulting in the corresponding alcohols **2an**–**aq** in good yields. As with secondary benzylic C(sp^3^)–H oxidation, HFIP served as an additive to suppress the overoxidation of the alcohol products to the ketones. Despite reported technical challenges with scaling up batch photochemical reactions due to low quantum efficiency,^22^ we were able to demonstrate that direct C–H hydroxylation of **1ag** resulted in a 62% isolated yield of **2ag** on a gram scale.

After establishing the reaction scope, we turned our attention to investigating the mechanism of the transformation (Scheme 2). Intermolecular kinetic isotope effect (KIE) studies of the benzylic C–H hydroxylation resulted in a *k*_H_/*k*_D_ value of 1.7, which is similar to those for reported benzylic C–H hydroxylation protocols (Scheme 2A).^23^ Intramolecular and parallel KIE experiments both resulted in *k*_H_/*k*_D_ values of 1.6 (see the SI). These KIE experiments support that HAT of the C(sp^3^)–H bond with the photoexcited nitroarene participates in the rate-limiting step of the transformation. Next, radical probe **5** was subjected to the reaction conditions to verify the formation of radical intermediates (Scheme 2B).^24^ The formation of naphthalene derivative **8** was observed as a product with concomitant formation of the direct anaerobic oxidation products **6** and **7**. The former likely occurs via radical ring opening of **5** and subsequent aromatization via hydroxylation/dehydration (see the SI), thus verifying the intermediacy of carbon-centered radicals. To detect the formation of elusive reaction intermediates during the transformation, the hydroxylation of **1ac** was monitored using PhotoNMR spectroscopy at 23 °C (see the SI).^25^ Although the reaction did not go to completion due to inefficient stirring, the radical recombination product **9** was detected (Scheme 2C). Further support for **9** was acquired via high-resolution mass spectrometry (HRMS) studies of the crude reaction mixture. The azoarene (**13**) and azoxyarene (**14**) byproducts were isolated from the reaction mixture of **2ag** and characterized by NMR and HRMS (Scheme 3). These byproducts, which form as the nitroarene is consumed and are present at the end of the reaction, are presumably generated via condensation of in situ-formed aniline and *N*-hydroxyaniline with the nitrosoarene.^12,26^ Markedly, observation of these side products illustrates that fragmentation of the radical recombination product (**12**) leads to the desired C–H hydroxylation products (**2**) and the nitrosoarene byproduct.

**Scheme 2:**
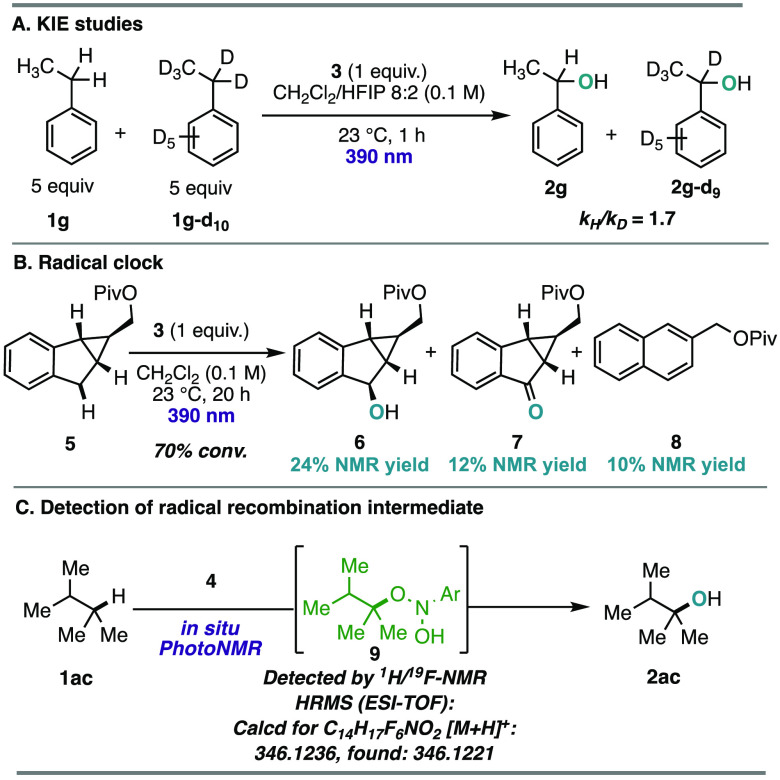
Mechanistic Studies

**Scheme 3:**
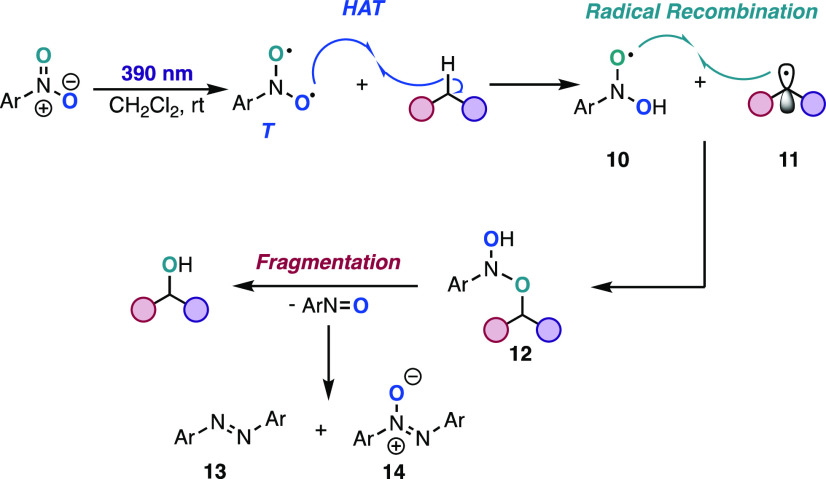
Proposed Mechanism

Based on the above studies, we propose the following mechanism for this transformation (Scheme 3). Direct photoexcitation of the nitroarene leads to the triplet biradical intermediate, which undergoes HAT with the C(sp^3^)–H bond of the hydrocarbon to generate alkyl radical **11** and oxygen-centered dihydroxyaniline radical **10.** Radical recombination of **10** and **11** leads to intermediate **12**.^27^ An alternate chain mechanism where **11** recombines with the ground-state nitroarene to generate **12** is not supported based on our radical chain studies and a nitroarene crossover experiment (see the SI). Also, hydrolysis of **12** with adventitious water to generate the alcohol product is unlikely based on the lack of ^18^O incorporation when H_2_^18^O was added to the reaction conditions (see the SI). Finally, fragmentation of **12** leads to the oxygen atom transfer product and the nitroso byproduct, the latter of which rapidly condenses under the reaction conditions to form side products **13** and **14**.^28^

In summary, we have reported an anaerobic C–H hydroxylation of aliphatic systems promoted by photoexcited nitroarenes. Based on the bifunctional reactivity of photoexcited nitroarenes, the formed triplet biradical excited state can enable the activation of C(sp^3^)–H bonds and the oxygen atom transfer event. Notably, this C–H hydroxylation protocol does not require additional oxidants and/or transition metals, making this a cost-effective and atom-economical approach compared to established methods. Moreover, because of the anaerobic nature of the transformation, C–H hydroxylation of aliphatic systems possessing oxidatively sensitive functional groups can be achieved without issues of overoxidation. Radical clock studies support that the C–H bond activation occurs via HAT with the photoexcited nitroarene, and kinetic isotopic experiments indicate that this pathway is involved in the rate-limiting step of the reaction. PhotoNMR studies and HRMS analysis provide evidence for the formation of the putative radical recombination intermediate, *N*-arylhydroxylamine ether, which undergoes fragmentation, leading to the C–H hydroxylation products. Overall, this work demonstrates that photoexcited nitroarenes enable synthetically useful C–H hydroxylation events in a mild, practical, and sustainable manner. We anticipate that this method will become a universal paradigm for sustainable oxygen atom transfer events for applications in the late-stage synthesis of medicinally relevant compounds.

## Supporting Information Available

Experimental details, optimization studies, characterization data, and NMR spectra (PDF)
