# Supplementary material for: Anaerobic Hydroxylation of C(sp3)–H Bonds Enabled by the Synergistic Nature of Photoexcited Nitroarenes
Source: J Am Chem Soc. 2023 Jan 25;145(5):2794–9. doi: 10.1021/jacs.2c13502 (PMC10032565; doi:10.1021/jacs.2c13502)
Supplement: Supplementary file 1 — ja2c13502_si_001.pdf [file ja2c13502_si_001.pdf]

Anaerobic Hydroxylation of C(sp^3)–H Bonds Enabled by the Synergistic Nature of Photoexcited Nitroarenes

Joshua M. Paolillo,[‡] Alana D. Duke,[‡] Emma S. Gogarnoiu,[§] Dan E. Wise,[§] and Marvin Parasram*

Department of Chemistry, New York University, 24 Waverly Place, 3rd floor, New York, NY 10003

Supporting Information

General information	1
Optimization of the reaction parameters for benzylic systems	2
Optimization of the reaction parameters for unactivated systems	6
General procedures	8
Substrate synthesis	9
Characterization of hydroxylated products	12
Mechanism studies	26
Kinetic isotope effect studies	26
Radical clock studies	30
Radical cation probe	37
Labeling studies	40
Detecting reaction byproducts	43
PhotoNMR studies	45
Light On/Off Studies	49
Scale up procedure	51
UV-Vis Spectrum of Nitroarenes	52
NMR spectra	53
NMR yield spectra	102
References	107

General information

All reactions were carried out in oven-dried glassware under a nitrogen atmosphere, unless otherwise stated. Solvents were dried and deoxygenated by passing through alumina in a solvent purification system. $CDCl_3$, DMSO-d_6, acetic acid-d_4 and CD_2Cl_2 were purchased from Cambridge Isotope Laboratories. All alkane substrates were purchased from commercial sources, unless otherwise noted, and used without further purification.

All NMR spectra (^{1}H, ^{13}C, and ^{19}F) were recorded on Bruker 400 MHz and 500 MHz Avance spectrometers. The chemical shifts (δ) are given in parts per million and referenced to residual solvent peaks. Coupling constants (*J*) are reported in Hertz (Hz) to the nearest 0.1 Hz. The following multiplicity abbreviations are used: s singlet, d doublet, t triplet, m multiplet. GC chromatograms were taken on an Agilent 8890 GC with 5977B MSD, and helium as the carrier gas. High-resolution mass spectra (HRMS) were obtained on an Agilent 6224 TOF LC/MS which was acquired through the support of New York University. UV-visible spectra were recorded on a Cary 100 Bio UV-Visible spectrophotometer. We utilized 34 W Kessil Lamps with varying wavelengths as well as 18 W EvoluChem 405 nm LEDs for our photochemical set ups.

Optimization of the reaction parameters for benzylic systems

Table S1: Wavelength, Solvent, and Concentration Screen for benzylic substrates

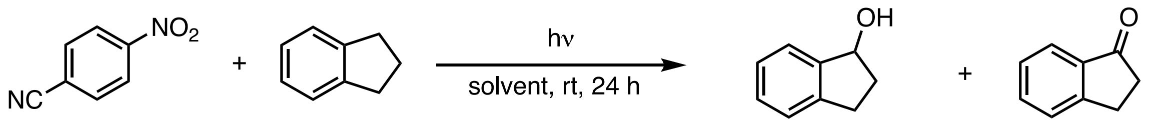

Wavelength (nm)	Solvent	Concentration (M)	Conversion[a] (%)	^{1}H NMR Yield of **2a**[b] (%)	^{1}H NMR Yield of **2a`**[b] (%)
456	MeCN	0.1	0	0	0
440	MeCN	0.1	80	55	14
427	MeCN	0.1	90	60	7
405	MeCN	0.1	95	50	59
390	MeCN	0.1	100	60	56
427	MeCN	0.2	70	30	35
427	MeCN	0.05	100	55	50
427	MeCN	0.025	100	40	20
427	MeCN	0.01	100	50	28
427	MTBE	0.1	77	64	24
427	DMSO	0.1	70	25	50
427	DMAc	0.1	90	0	0
427	MeOH	0.1	91	65	40
427	IPA	0.1	90	62	23
427	DCM	0.1	100	80	25
**390**	**DCM**	**0.1**	**100**	**80**	**20**

Reactions were performed on a 0.1 mmol scale. [a]Conversion based on consumption of nitroarene. [b]Yields calculated using CH_2Br_2 as an external standard.

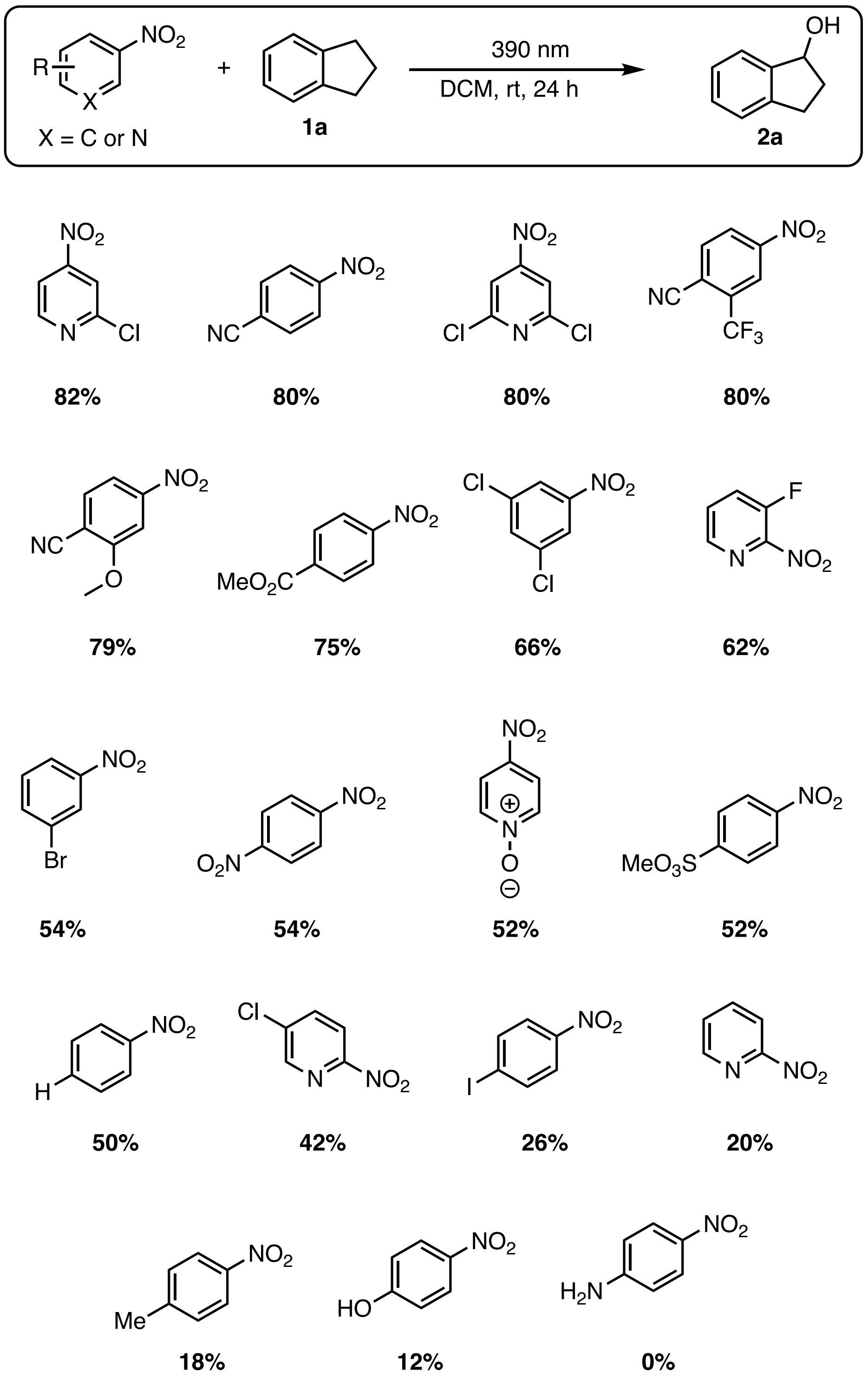

Figure S1: Benzylic Nitroarene Screen. Reactions were performed on a 0.1 mmol scale and the yield of **2a** was determined by ^{1}H NMR using CH_2Br_2 as an external standard.

Table S2: Equivalence and Additive Screening for benzylic substrates

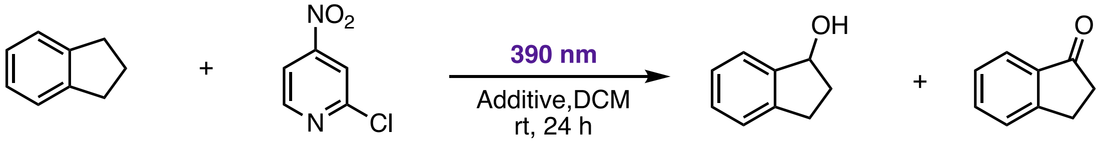

1a **3** **2a** **2a`**

Additive	Additive Equiv.	Equiv. **1a**	^{1}H NMR Yield **2a**[a] (%)	^{1}H NMR Yield **2a`**[a] (%)
None	-	0.66	40	40
None	-	1	70	25
None	-	2	85	14
None	-	5	86	13
HCl (2M)	3	2	22	48
Acetic acid	3	2	40	32
Triethyl amine	3	2	22	0
K_2CO_3	3	2	74	25
Timethylborate	3	2	70	20
Hantzsch Ester	3	2	0	0
Urea	2	2	81	30
$PhB(OH)_2$	2	2	55	30
Trifluoroethanol	20	2	81	32
**HFIP**	**20**	**2**	**92**	**4**

Reactions were performed on a 0.1 mmol scale. [a]Yields calculated using CH_2Br_2 as an external standard.

Table S3: Solvent Ratio Screen for benzylic susbtrates

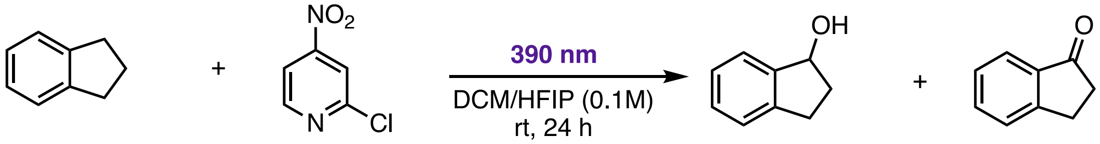

1a **3** **2a** **2a`**

DCM:HFIP	Equiv. **1a**	Equiv. **3**	Conversion[a] (%)	^{1}H NMR Yield **2a**[b] (%)	^{1}H NMR Yield **2a`**[b] (%)
100:0	2	1	100	85	14
9:1	2	1	100	83	15
**8:2**	**2**	**1**	**100**	**92**	**4**
8:2	1	1.5	85	32	51
3:1	2	1	100	91	4
1:1	2	1	100	92	3
1:3	2	1	100	90	3
0:100	2	1	100	91	4
0:100	1	1.5	82	38	40

[a]Conversion based on consumption of 3. [b]Yields were calculated using CH_2Br_2 as an external standard.

Table S4: Control Experiments

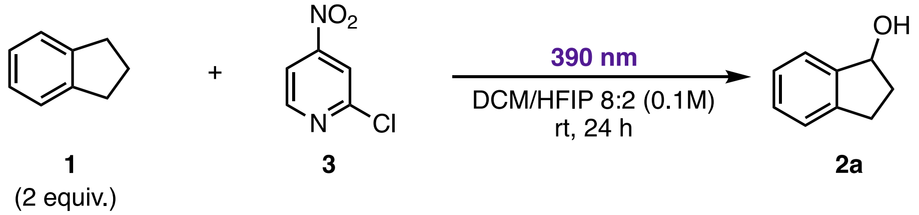

Deviation from Standard Conditions	Solvent	^{1}H NMR yield **2a**[a] (%)
No **3**	DCM/HFIP 8:2	NR
No light, at 80 ºC	Trifluorotoluene/HFIP 8:2	NR
Degassed[b]	DCM/HFIP 8:2	91
Under air	DCM/HFIP 8:2	89

Reactions were performed on a 0.1 mmol scale. [a]Yields were calculated using CH_2Br_2 as an external standard. [b]Reaction mixture thoroughly degassed by three freeze-pump-thaw cycles.

Table S5: Byproduct Control Experiments

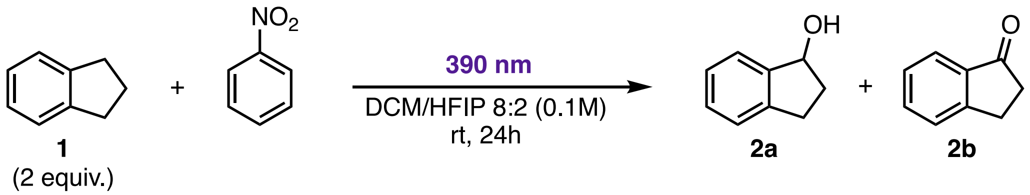

Deviation from Conditions	^{1}H NMR yield **2a**[a] (%)	^{1}H NMR yield **2b**[a] (%)
**none**	**9**	**24**
**Nitrosobenzene** instead of Nitrobenzene	NR	NR
**Azoxybenzene** instead of Nitrobenzene	NR	NR

Reactions were performed on a 0.1 mmol scale. [a]Yields were calculated using CH_2Br_2 as an external standard.

Optimization of the reaction parameters for unactivated systems

Table S6: Solvent screen for unactivated systems

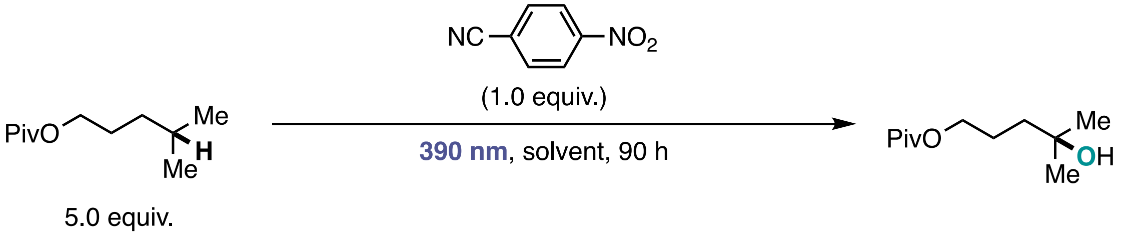

Solvent	^{1}H NMR Yield[a] (%)
MeCN	13
Trifluorotoluene (TFT)	20
DCM	13
MeOH	ND
DMF	ND
Et_2O	ND

Reactions performed on a 0.1 mmol scale. [a]Yields were calculated using CH_2Br_2 as an external standard.

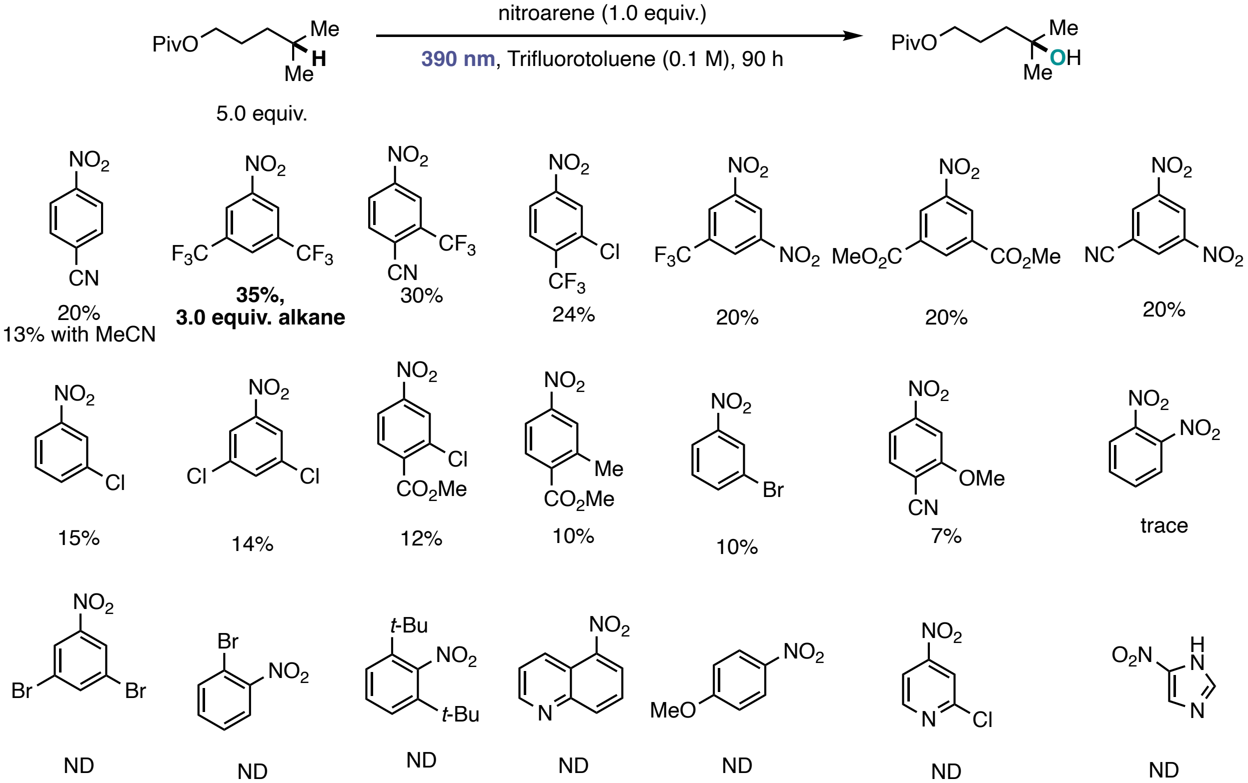

Figure S2: Nitroarene screen for unactivated substrates. Reactions performed on a 0.1 mmol scale. Yields were calculated with ^{1}H NMR using CH_2Br_2 as an external standard.

Table S7: Equivalence and concentration screen for unactivated systems

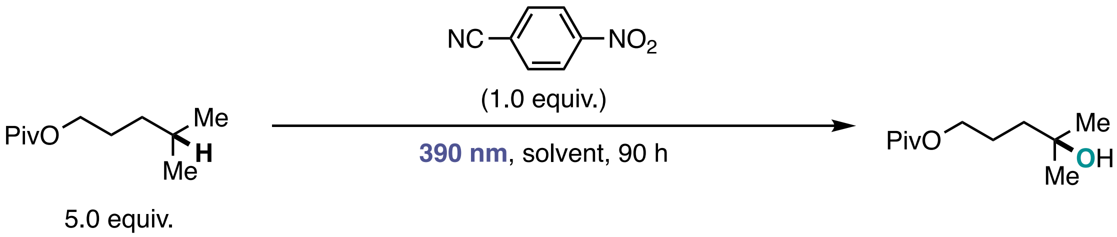

Solvent	Conc.	Equiv. alkane	^{1}H NMR Yield[a] (%)
TFT	0.1 M	3.0	35
MeCN	0.1 M	3.0	43
MeCN	0.1 M	2.0	34
MeCN	0.1 M	1.0	21
MeCN	0.1 M	4.0	53
MeCN	0.1 M	5.0	63
**-**	**neat**	**5.0**	**72**
Hexafluoroisopropanol	0.1 M	3.0	7

Reactions performed on a 0.1 mmol scale. [a]Yields were calculated using CH_2Br_2 as an external standard.

Table S8: Secondary unactivated optimization

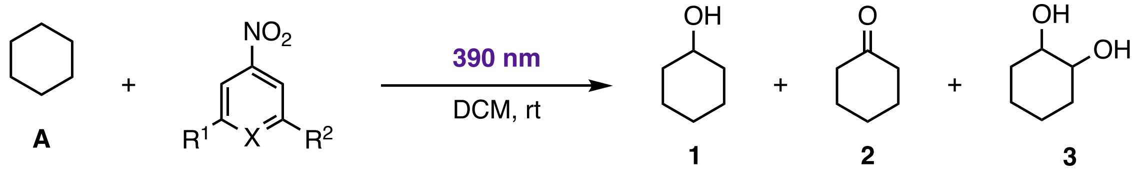

Nitroarene	Conc.	Time	Equiv. A	^{1}H NMR Yield **1**[a] (%)	^{1}H NMR Yield **2**[a] (%)	^{1}H NMR Yield **3**[a] (%)
R^1= H, X = N, R^2= Cl	0.1 M	24 h	2.0	20	11	ND
R^1= H, X = N, R^2= Cl	0.1 M	48 h	2.0	26	19	ND
R^1= CF_3, X = C, R^2= CF_3	0.1 M	48 h	2.0	26	38	ND
R^1= CF_3, X = C, R^2= CF_3	0.1 M	48 h	4.0	48	25	ND
R^1= CF_3, X = C, R^2= CF_3	0.1 M	48 h	5.0	58	26	ND
R^1= CF_3, X = C, R^2= CF_3	1.0 M	48 h	5.0	32	43	16
R^1= CF_3, X = C, R^2= CF_3	neat	48 h	5.0	32	49	20
**R^1= CF_3, X = C, R^2= CF_3**	**2 equiv. HFIP**	**48 h**	**5.0**	**72**	**10**	**ND**

Reactions run on a 0.1 mmol scale. [a]Yields were calculated using CH_2Br_2 as an external standard.

General procedures

General Procedure A: Standard C–H Hydroxylation Procedure for Benzylic Substrates

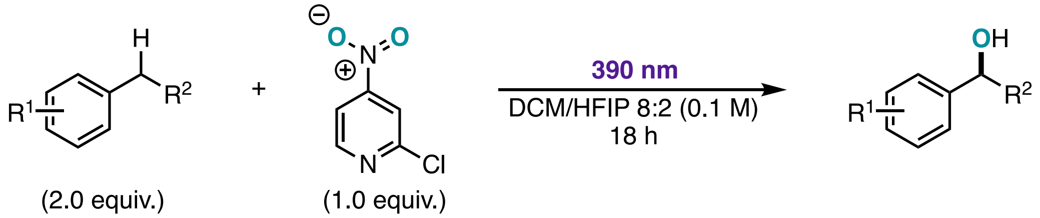

2-chloro-4-nitropyridine (159 mg, 1 mmol, 1 equiv.) & alkane (2 mmol, 2 equiv.) were added to a 6-dram vial (flame dried or oven dried) equipped with a stir bar. The reaction vial was purged with N_2 flow for 15 min followed by the addition of 8:2 dichloromethane/hexafluoroisopropanol (10 mL, 0.1 M). The vial was placed on a stir plate in front of a 390 nm lamp with a cooling fan. The stir rate was set to 800 rpm and the reaction was irradiated for 18 hours or until the reaction was completed as determined by GCMS analysis. After the reaction had reached completion, the solvent was removed *in vacuo*. The residue was then diluted with 5 mL of diethyl ether followed by 10 mL of hexanes. The mixture was stirred for 1 hour and was then filtered to remove solids. The filtrate was then transferred to a separatory funnel and washed with 15 mL of 0.5 M HCl. The layers were separated, and the aqueous layer was extracted 3 × with 10 mL of diethyl ether. The combined organic layers were washed once with 25 mL of DI H_2O, then once with brine, and then was dried over sodium sulfate. The solvent was removed *in vacuo* and the crude product was purified by column chromatography to afford the desired alcohol product.

General Procedure B: Standard C–H Hydroxylation Procedure for Unactivated Substrates

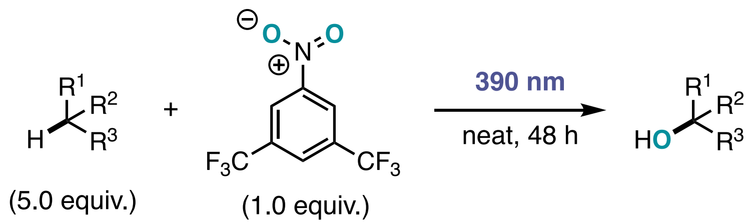

A flame-dried 2-dram vial equipped with a stir bar was purged with N_2 flow for 15 min. 3,5-trifluoromethylnitrobenzene (172.7 µL, 1.0 mmol, 1.0 equiv.), alkane (5.0 mmol, 5 equiv.), and, where applicable, hexafluoroisopropanol (200 - 400 µL, 2.0 – 4.0 equiv.) were added. Anhydrous, degassed DCM was added where applicable. The vial was placed on a stir plate in front of a 390 nm lamp with a cooling fan, the stir rate was set to 1500 rpm, and the reaction was irradiated for 48 hours. Solvent was removed *in vacuo*. The crude product was then purified by column chromatography (0 - 2% MeOH/DCM) to afford the alcohol product.

Substrate synthesis

Literature Reported

6,7,8,9-tetrahydro-5H-benzo[7]annulene **(1c)**[1], *tert*-butyl (2,3-dihydro-1*H*-inden-5-yl)carbamate **(1d)**[2], 1-ethyl-4-isopropylbenzene **(1t)**[3], Benzylcyclopropane **(1y)**[4], (2-methylphenyl)-2,3,4,6-tetra-O-acetyl-β-D-glucopyranoside **(1aa)**[5], 3-(Acetoxy)-estra-1,3,5-(10)-trien-17-one **(1ab)**[6], (3*s*,5*s*,7*s*)-adamantan-1-yl pivalate **(1ae)**[7], 4-methylpentyl pivalate **(1ag)**,[43] *trans*-4-Methylcyclohexyl 2,2-dimethylpropanoate **(1ai)**,[42] 2-(4-methylpentyl)isoindoline-1,3-dione **(1ak)**[8], (*R*)-2-isopentyl-4-oxooxetane-2-carboxylic acid **(1am)**[9] and (propyl-1-*d*)benzene[45] were synthesized according to literature.

Substrate Synthesis

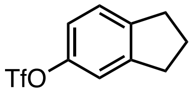

2,3-dihydro-1H-inden-5-yl trifluoromethanesulfonate (1e)

To a flame dried round bottom flask equipped with a stir bar was added 2,3-dihydro-1H-inden-5-ol (2.68 g, 1 equiv., 20.0 mmol), dichloroethane (40 mL, 0.5 M), and pyridine (3.16 g, 3.24 mL 2 equiv., 40.0 mmol). The mixture was cooled to 0 °C and trifluoromethanesulfonic anhydride was added dropwise to the reaction mixture. The mixture was allowed to warm to room temperature and stirred for 1 hour. Upon completion of the reaction as determined by TLC and GCMS analysis, the mixture was diluted with 20 mL of diethyl ether and quenched with 30 mL of 2M HCl. The contents of the flask were transferred to a separatory funnel and the layers were separated. The organic layer was washed once with 15 mL of 2M HCl, then 20 mL of saturated sodium bicarbonate solution, then once with 20 mL of brine. The organic layer was then dried over sodium sulfate and concentrated to afford the crude product, which was purified by flask chromatography (9:1 hexanes/EtOAc) to afford **1e** as a clear, colorless oil (4.82 g, 91% yield).

^{1}H NMR (500 MHz, $CDCl_3$) (δ, ppm): 7.25 (d, J = 8.1 Hz, 1H), 7.12 (d, J = 2.4 Hz, 1H), 7.02 (dd, J = 8.2, 2.4 Hz, 1H), 2.94 (dt, J = 15.2, 7.5 Hz, 4H), 2.14 (p, J = 7.5 Hz, 2H).
^{13}C NMR (126 MHz, $CDCl_3$) (δ, ppm): 148.4, 146.9, 125.6, 119.0, 118.9 (q, J = 320.7 Hz), 117.5, 33.1, 32.4, 25.9.
^{19}F NMR (471 MHz, $CDCl_3$) (δ, ppm): -72.9.
HRMS (ESI-TOF): Calcd. for $C_{10}H_9F_3O_3S$ $[M]^+$ for 266.0224, found 266.0228.

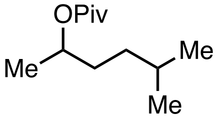

5-methylhexan-2-yl pivalate (1af)

Modified from analogous literature reported method.[42] All spectra were in accordance with literature reported spectra.[44]

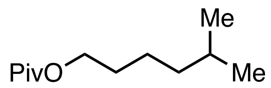

5-methylhexyl pivalate (1ah)

Modified from analogous literature reported method.[42] Anhydrous DCM, 5-methylhexan-ol (871 mg, 1 equiv. 7.50 mmol), DMAP (91.6 mg, 0.1 equiv., 0.750 mmol) were added to flame dried RBF. This was allowed to stir at rt for 5 minutes, then triethylamine (1.52 g, 2 equiv., 15.0 mmol) and Pivalate anhydride (1.68 g, 1.2 equiv, 9.00 mmol) were added. The reaction was allowed to stir at rt for 15 hours. H_2O was added to quench, and the reaction was extracted with DCM (3 × 15 mL). The organic layer was washed multiple times with water and NaOH solution, then brine, and concentrated. Purified with 10 % DCM in Hexanes.

^{1}H NMR (400 MHz, $CDCl_3$) (δ, ppm): 4.05 (t, *J* = 6.6 Hz, 2H), 1.65 – 1.28 (m, 6H), 1.20 (m, 1H), 1.19 (s, 9H), 0.87 (d, *J* = 6.6 Hz, 6H).
HRMS (ESI-TOF): $C_{12}H_{24}O_2$ $[M]^+$ 200.1776, found 200.1775.

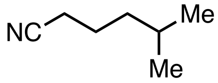

5-methylhexanenitrile (1aj)

Potassium cyanide (1.50 g, 23.0 mmol, 1.1 equiv.) was combined with tributylamine (775 mg, 4.18 mmol, 0.2 equiv.), 4-methylpentyl bromide (3.45 g, 20.9 mmol, 1.0 equiv.) in 3.5 mL water. The mixture was then refluxed at 105 °C for 20 hrs. The reaction was cooled to room temperature and product was extracted in DCM (3 × 20 mL). The combined organic layer was washed with 1M HCL then water, and dried with Na_2SO_4. Solvent was removed *in vacuo* to provide to yield yellow liquid (1.09 g, 47% yield). All spectra were in accordance with literature reported spectra.[10]

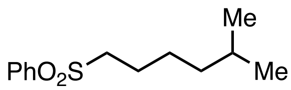

((5-methylhexyl)sulfonyl)benzene (1al)

Modified analogous literature reported method.[11] To a flame dried round bottom flask equipped with a stir bar was added (methylsulfonyl)benzene (2.34 g, 15.0 mmol), 2 M lithium diisopropylamine in THF (7.88 mL, 1.05 equiv., 15.8 mmol), and THF (150 mL) at 0 ºC and stirred 1 hour. Then 1-bromo-4-methylpentane (2.60 g, 1.05 equiv., 15.8 mmol) was added dropwise and the reaction was allowed to warm room temperature for 5 hours. Upon completion of the reaction, 15 mL of water was added. The contents of the flask were transferred to a separatory funnel and dilted with 100 mL of DCM. The product was extracted in DCM (3 × 100 mL). The combined organic layers were washed with brine, dried $NaSO_4$ and the solvent was removed *in vacuo*. The crude mixture was purified using flash chromatography (30% ethyl acetate in hexanes) to afford the pure sulfone **1al** as clear oil (2.47 g, 68%).

^{1}H NMR (500 MHz, $CDCl_3$) (δ, ppm): 7.93 – 7.88 (m, 2H), 7.69 – 7.61 (m, 1H), 7.57 (dd, *J* = 8.5, 7.1 Hz, 2H), 3.12 – 3.05 (m, 2H), 1.74 – 1.64 (m, 2H), 1.47 (septet, *J* = 13.3, 6.7 Hz, 1H), 1.39 – 1.29 (m, 2H), 1.17 – 1.09 (m, 2H), 0.83 (d, *J* = 6.6 Hz, 5H).
^{13}C NMR (126 MHz, $CDCl_3$) (δ, ppm): 139.4, 133.7, 129.3, 128.1, 56.4, 38.3, 27.8, 26.2, 23.0, 22.5.
HRMS (ESI-TOF): calcd. for $C_{13}H_{21}O_2S$ $[M+H]^+$ 241.1257, found 241.1249.

Characterization of hydroxylated products

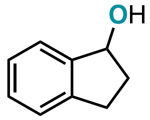

1-indanol (2a)
Prepared according to general procedure A (18 hours). The title compound was isolated via flash chromatography (gradient 95:5 – 8:2 hexanes/EtOAc) as a tan solid (110 mg, 82% yield). All analytical data for **2a** was in accordance with literature data.[12]

^{1}H NMR (500 MHz, $CDCl_3$) (δ, ppm): 7.47 – 7.39 (m, 1H), 7.30 – 7.21 (m, 3H), 5.25 (t, J = 6.1 Hz, 1H), 3.07 (ddd, J = 16.0, 8.6, 4.8 Hz, 1H), 2.83 (ddd, J = 15.6, 8.3, 6.6 Hz, 1H), 2.50 (dddd, J = 13.2, 8.3, 6.9, 4.8 Hz, 1H), 1.95 (dddd, J = 13.5, 8.5, 6.6, 5.2 Hz, 1H), 1.74 (s, 1H).
^{13}C NMR (126 MHz, $CDCl_3$) (δ, ppm): 145.1, 143.4, 128.4, 126.8, 125.0, 124.3, 76.6, 36.1, 29.9.

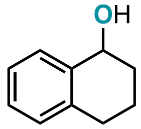

1,2,3,4-tetrahydronaphthalen-1-ol (2b)
Prepared according to general procedure A (18 hours). The title compound was isolated via flash chromatography (gradient 95:5 – 8:2 hexanes/EtOAc) as a light-yellow oil (114 mg, 76% yield). All analytical data for **2b** was in accordance with literature data.[13]

^{1}H NMR (500 MHz, $CDCl_3$) (δ, ppm): 7.48 – 7.40 (m, 1H), 7.24 – 7.17 (m, 2H), 7.15 – 7.09 (m, 1H), 4.79 (t, J = 4.8 Hz, 1H), 2.84 (dt, J = 16.5, 5.5 Hz, 1H), 2.78 – 2.68 (m, 1H), 2.06 – 1.87 (m, 3H), 1.85 – 1.75 (m, 1H), 1.72 (s, 1H).
^{13}C NMR (126 MHz, $CDCl_3$) (δ, ppm): 138.9, 137.2, 129.1, 128.7, 127.7, 126.3, 68.2, 32.4, 29.3, 18.9.

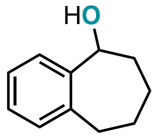

6,7,8,9-tetrahydro-5H-benzo[7]annulen-5-ol (2c)
Prepared according to general procedure A (18 hours). The title compound was isolated via flash chromatography (gradient 95:5 – 8:2 hexanes/EtOAc) as a white solid (124 mg, 76% yield). All analytical data for **2c** was in accordance with literature data.[14]

^{1}H NMR (500 MHz, $CDCl_3$) (δ, ppm): 7.44 (d, J = 7.5 Hz, 1H), 7.21 (td, J = 7.4, 1.5 Hz, 1H), 7.15 (td, J = 7.3, 1.5 Hz, 1H), 7.10 (dd, J = 7.4, 1.5 Hz, 1H), 5.02 – 4.87 (m, 1H), 2.93 (ddd, J = 14.4, 8.1, 1.8 Hz, 1H), 2.72 (ddd, J = 14.3, 10.5, 1.7 Hz, 1H), 2.05 (ddd, J = 10.6, 6.9, 3.2 Hz, 1H), 1.96 (tt, J = 9.4, 2.2 Hz, 1H), 1.87 – 1.71 (m, 4H), 1.54 – 1.38 (m, 1H).
^{13}C NMR (101 MHz, $CDCl_3$) (δ, ppm): 144.4, 140.9, 129.6, 127.1, 126.2, 124.7, 74.1, 36.7, 35.9, 27.9, 27.7.

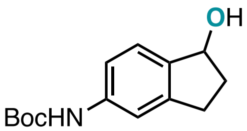

tert-butyl (1-hydroxy-2,3-dihydro-1H-inden-5-yl)carbamate (2d)
Prepared according to general procedure A (18 hours). The title compound was isolated via flash chromatography (gradient 95:5 – 8:2 hexanes/EtOAc) as a white solid (102 mg, 41% yield).

^{1}H NMR (500 MHz, $CDCl_3$) (δ, ppm): 7.41 (s, 1H), 7.31 (d, J = 8.1 Hz, 1H), 7.14 – 7.05 (m, 1H), 6.47 (s, 1H), 5.20 (t, J = 5.8 Hz, 1H), 3.04 (ddd, J = 16.1, 8.5, 5.2 Hz, 1H), 2.79 – 2.72 (m, 1H), 2.52 – 2.42 (m, 1H), 1.95 (dddd, J = 13.1, 8.4, 6.2, 4.9 Hz, 1H).
^{13}C NMR (126 MHz, $CDCl_3$) (δ, ppm): 152.9, 144.8, 139.9, 138.8, 124.7, 117.5, 115.1, 80.7, 76.2, 36.3, 30.1, 28.5.
HRMS (ESI-TOF): calcd. for $C_{14}H_{19}NO_3Na$ $[M+Na]^+$ 272.1257, found 272.1251.

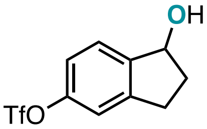

1-hydroxy-2,3-dihydro-1H-inden-5-yl trifluoromethanesulfonate (2e)
Prepared according to general procedure A (18 hours). The title compound was isolated via flash chromatography (gradient 95:5 – 7:2 hexanes/EtOAc) as a yellow oil (160 mg, 57% yield).

^{1}H NMR (500 MHz, $CDCl_3$) (δ, ppm): 7.45 (d, J = 8.1 Hz, 1H), 7.19 – 7.06 (m, 2H), 5.23 (t, J = 6.3 Hz, 1H), 3.07 (ddd, J = 16.4, 8.7, 4.5 Hz, 1H), 2.88 – 2.80 (m, 1H), 2.54 (dddd, J = 13.1, 8.3, 6.9, 4.5 Hz, 1H), 2.13 (s, 1H), 1.99 (dddd, J = 13.0, 8.7, 7.0, 5.6 Hz, 1H).
^{13}C NMR (126 MHz, $CDCl_3$) (δ, ppm): 149.8, 146.0, 145.4, 125.9, 119.9, 118.8 (q, J = 320.7 Hz), 118.0, 75.5, 36.3, 29.9.
^{19}F NMR (471 MHz, $CDCl_3$) (δ, ppm): -72.9.
HRMS (ESI-TOF): calcd. for $C_{10}H_7F_3O_3S$ $[M+H-H_2O]^+$ 265.0141, found 265.0136.

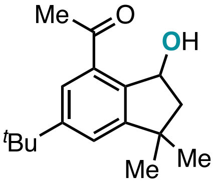

1-(6-(tert-butyl)-3-hydroxy-1,1-dimethyl-2,3-dihydro-1H-inden-4-yl)ethan-1-one (2f)
Prepared according to general procedure A (18 hours). The title compound was isolated via flash chromatography (8:2 hexanes/EtOAc) as a white solid (147 mg, 57% yield). All analytical data for **2f** was in accordance with literature data.[15]

^{1}H NMR (400 MHz, $CDCl_3$) (δ, ppm): 7.77 (d, J = 1.8 Hz, 1H), 7.43 (d, J = 1.7 Hz, 1H), 5.38 (dd, J = 7.6, 3.9 Hz, 1H), 4.52 (s, 1H), 2.68 (s, 3H) 2.29 (dd, J = 13.5, 7.6 Hz, 1H), 2.05 (dd, J = 13.6, 3.9 Hz, 1H), 1.40 (s, 3H), 1.37 (s, 9H), 1.26 (s, 3H).
^{13}C NMR (101 MHz, $CDCl_3$) (δ, ppm): 202.9, 154.5, 152.4, 142.4, 133.8, 126.4, 124.8, 73.4, 48.9, 43.0, 35.1, 31.6, 31.1, 30.1, 28.1.

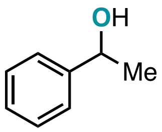

1-phenylethanol (2g)
Prepared according to general procedure A (18 hours). The title compound was isolated via flash chromatography (95:5 – 8:2 hexanes/EtOAc) as a colorless oil (96 mg, 79% yield). All analytical data for **2g** was in accordance with literature data.[12]

^{1}H NMR (500 MHz, $CDCl_3$) (δ, ppm): 7.41 – 7.33 (m, 4H), 7.31 – 7.26 (m, 1H), 4.90 (q, J = 6.5 Hz, 1H), 1.89 (s, 1H), 1.50 (d, J = 6.5 Hz, 3H).
^{13}C NMR (126 MHz, $CDCl_3$) (δ, ppm): 145.9, 128.6, 127.6, 125.5, 70.5, 25.2.

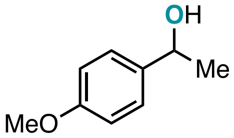

1-(4-methoxyphenyl)ethan-1-ol (2h)
Prepared according to general procedure A (18 hours) with 50% light intensity. The title compound was isolated via flash chromatography (95:5 – 8:2 hexanes/EtOAc) as a light-yellow oil (105 mg, 69% yield). All analytical data for **2h** was in accordance with literature data.[12]

^{1}H NMR (500 MHz, $CDCl_3$) (δ, ppm): 7.30 (d, J = 8.7 Hz, 2H), 6.89 (d, J = 8.7 Hz, 2H), 4.86 (q, J = 6.4 Hz, 1H), 3.81 (s, 3H), 1.78 (s, 1H), 1.48 (d, J = 6.5 Hz, 3H).
^{13}C NMR (126 MHz, $CDCl_3$) (δ, ppm): 159.1, 138.1, 126.8, 114.0, 70.1, 55.4, 25.2.

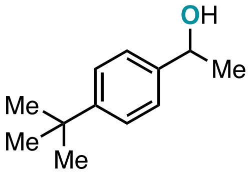

1-(4-(tert-butyl)phenyl)ethan-1-ol (2i)
Prepared according to general procedure A (18 hours). The title compound was isolated via flash chromatography (gradient 95:5 – 8:2 hexanes/EtOAc) as a colorless oil (131 mg, 74% yield). All analytical data for **2i** was in accordance with literature data.[16]

^{1}H NMR (400 MHz, $CDCl_3$) (δ, ppm): 7.39 (d, J = 8.4 Hz, 2H), 7.32 (d, J = 8.4 Hz, 2H), 4.88 (q, J = 6.5 Hz, 1H), 1.84 (s, 1H), 1.50 (d, J = 6.5 Hz, 3H), 1.33 (s, 9H).
^{13}C NMR (101 MHz, $CDCl_3$) (δ, ppm): 150.6, 142.9, 125.5, 125.3, 70.3, 34.6, 31.5, 25.1.

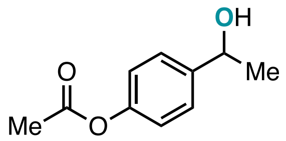

4-(1-hydroxyethyl)phenyl acetate (2j)

Prepared according to general procedure A (18 hours). The title compound was isolated via flash chromatography (8:2 hexanes/EtOAc) as a colorless oil (121 mg, 67% yield). All analytical data for **2j** was in accordance with literature data.[17]

^{1}H NMR (500 MHz, $CDCl_3$) (δ, ppm): 7.37 (d, J = 8.5 Hz, 2H), 7.05 (d, J = 8.6 Hz, 2H), 4.88 (q, J = 6.4 Hz, 1H), 2.29 (s, 3H), 2.00 (s, 1H), 1.47 (d, J = 6.5 Hz, 3H).
^{13}C NMR (126 MHz, $CDCl_3$) (δ, ppm): 169.7, 150.0, 143.5, 126.6, 121.7, 70.0, 25.3, 21.2.

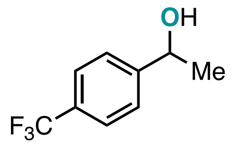

1-(4-(trifluoromethyl)phenyl)ethan-1-ol (2k)

Prepared according to general procedure A (24 hours). The title compound was isolated via flash chromatography (8:2 hexanes/EtOAc) as a colorless oil (65 mg, 34% yield). All analytical data for **2k** was in accordance with literature data.[18]

^{1}H NMR (400 MHz, $CDCl_3$) (δ, ppm): 7.60 (d, J = 8.3 Hz, 2H), 7.48 (d, J = 8.8 Hz, 2H), 4.96 (q, J = 6.5 Hz, 1H), 2.09 (s, 1H), 1.50 (d, J = 6.5 Hz, 3H).
^{13}C NMR (126 MHz, $CDCl_3$) (δ, ppm): 149.8, 129.7 (q, J = 32.4 Hz), 125.7, 125.5 (q, J = 3.8 Hz), 124.3 (q, J = 271.9 Hz), 69.9, 25.5.
^{19}F NMR (377 MHz, $CDCl_3$) (δ, ppm): -62.5.

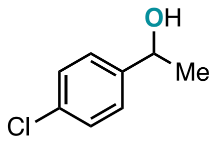

1-(4-chlorophenyl)ethan-1-ol (2l)

Prepared according to general procedure A (18 hours). The title compound was isolated via flash chromatography (8:2 hexanes/EtOAc) as a light-yellow oil (78 mg, 50% yield). All analytical data for **2l** was in accordance with literature data.[13]

^{1}H NMR (500 MHz, $CDCl_3$) (δ, ppm): 7.36 – 7.27 (m, 4H), 4.86 (q, J = 6.5 Hz, 1H), 1.95 (s, 1H), 1.46 (d, J = 6.5 Hz, 3H).
^{13}C NMR (126 MHz, $CDCl_3$) (δ, ppm): 144.4, 133.2, 128.7, 126.9, 69.8, 25.4.

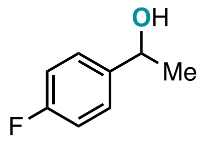

1-(4-fluorophenyl)ethan-1-ol (2m)

Prepared according to general procedure A (18 hours). The title compound was isolated via flash chromatography (8:2 hexanes/EtOAc) as a colorless oil (77 mg, 55% yield). All analytical data for **2m** was in accordance with literature data.[19]

^{1}H NMR (500 MHz, $CDCl_3$) (δ, ppm): 7.37 – 7.30 (m, 2H), 7.09 – 6.97 (m, 2H), 4.88 (q, *J* = 6.5 Hz, 1H), 1.87 (s, 1H), 1.47 (d, *J* = 6.5 Hz, 3H).
^{13}C NMR (126 MHz, $CDCl_3$) (δ, ppm): 162.2 (d, *J* = 245.1 Hz), 141.6 (d, *J* = 3.2 Hz), 127.1 (d, *J* = 8.1 Hz), 115.3 (d, *J* = 21.2 Hz), 69.9, 25.4.
^{19}F NMR (471 MHz, $CDCl_3$) (δ, ppm): -115.3.

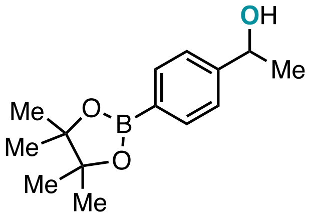

1-(4-(4,4,5,5-tetramethyl-1,3,2-dioxaborolan-2-yl)phenyl)ethan-1-ol (2n)

Prepared according to general procedure A (18 hours). The title compound was isolated via flash chromatography (gradient 95:5 – 8:2 hexanes/EtOAc) as a colorless oil (110 mg, 44% yield). All analytical data for **2n** was in accordance with literature data.[20]

^{1}H NMR (400 MHz, $CDCl_3$) (δ, ppm): 7.80 (d, *J* = 8.1 Hz, 2H), 7.38 (d, *J* = 7.9 Hz, 2H), 4.92 (q, *J* = 6.5 Hz, 1H), 1.49 (d, *J* = 6.5 Hz, 3H), 1.34 (s, 12H).
^{13}C NMR (101 MHz, $CDCl_3$) (δ, ppm): 149.1, 135.2, 124.8, 83.9, 70.6, 25.3, 25.0.

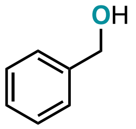

Phenylmethanol (2o)

Prepared according to general procedure A (18 hours) with HFIP (0.1 M) as the solvent. The title compound was isolated via flash chromatography (gradient 95:5 – 8:2 hexanes/EtOAc) as a colorless oil (72 mg, 67% yield). All analytical data for **2o** was in accordance with literature data.[18]

^{1}H NMR (500 MHz, $CDCl_3$) (δ, ppm): 7.37 (m, 4H), 7.30 (m, 1H), 4.69 (s, 2H), 1.86 (s, 1H).
^{13}C NMR (126 MHz, $CDCl_3$) (δ, ppm): 141.0, 128.7, 127.8, 127.1, 65.5.

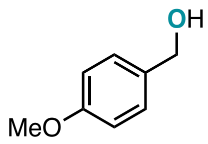

(4-methoxyphenyl)methanol (2p)

Prepared according to general procedure A (18 hours), but with an alternative workup. To a round bottom flask equipped with a stir bar was added CuO (7.96 mg, 0.100 mmol, 0.1 equiv.) and borane-ammonina complex (92.6 mg, 3.00 mmol, 3 equiv.), followed by 10 mL of ethanol. The crude reaction mixture was added dropwise and the progress was monitored by TLC and GCMS. Upon completion of the reaction, reaction was transferred to a separatory funnel and diluted with water (25 mL) and DCM (10 mL). The layers were separated, and the aqueous layer was extracted in DCM (3 × 15 mL). The combined organic layers were dried over sodium sulfate and the solvent was removed *in vacuo*. The title compound was isolated via flash chromatography (gradient 95:5 – 9:1 hexanes/EtOAc) as a light-yellow oil (73 mg, 53% yield). All analytical data for **2p** was in accordance with literature data.[21]

^{1}H NMR (500 MHz, $CDCl_3$) (δ, ppm): 7.29 (d, *J* = 8.6 Hz, 2H), 6.89 (d, *J* = 8.7 Hz, 2H), 4.61 (s, 2H), 3.81 (s, 3H), 1.78 (s, 1H).
^{13}C NMR (126 MHz, $CDCl_3$) (δ, ppm): 159.3, 133.3, 128.8, 114.1, 65.1, 55.4.

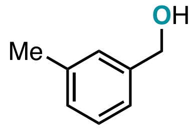

***m*-tolylmethanol (2q)**

Prepared according to general procedure A (18 hours) with HFIP (0.1 M) as the solvent. The title compound was isolated via flash chromatography (gradient 95:5 – 8:2 hexanes/EtOAc) as a colorless oil (65 mg, 53% yield). All analytical data for **2q** was in accordance with literature data.[21]

^{1}H NMR (400 MHz, $CDCl_3$) (δ, ppm): 7.30 – 7.22 (m, 1H), 7.20 – 7.15 (m, 2H), 7.12 (d, *J* = 7.6 Hz, 1H), 4.66 (s, 2H), 2.37 (s, 3H), 1.74 (s, 1H).
^{13}C NMR (101 MHz, $CDCl_3$) (δ, ppm): 140.9, 138.4, 128.6, 128.5, 127.9, 124.2, 65.5, 21.5.

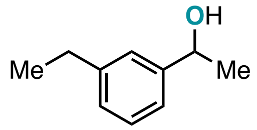

1-(3-ethylphenyl)ethan-1-ol (2r)

Prepared according to general procedure A (18 hours). The title compound was isolated via flash chromatography (8:2 hexanes/EtOAc) as a light-yellow oil (83 mg, 56% yield). All analytical data for **2r** was in accordance with literature data.[22]

^{1}H NMR (500 MHz, $CDCl_3$) (δ, ppm): 7.28 – 7.22 (m, 1H), 7.22 – 7.13 (m, 2H), 7.09 (d, *J* = 7.2 Hz, 1H), 4.85 (q, *J* = 6.5 Hz, 1H), 2.64 (q, *J* = 7.6 Hz, 2H), 1.83 (s, 1H), 1.47 (d, *J* = 6.5 Hz, 3H), 1.23 (t, *J* = 7.6 Hz, 3H).
^{13}C NMR (126 MHz, $CDCl_3$) (δ, ppm): 146.0, 144.7, 128.6, 127.2, 125.1, 122.8, 70.6, 29.0, 25.2, 15.7.

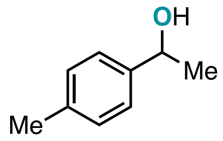

1-(p-tolyl)ethan-1-ol (2s)
Prepared according to general procedure A (18 hours). The title compound was isolated via flash chromatography (gradient 95:5 – 8:2 hexanes/EtOAc) as a colorless oil (112 mg, 82% yield). All analytical data for **2s** was in accordance with literature data.[12]

^{1}H NMR (500 MHz, $CDCl_3$) (δ, ppm): 7.27 (d, J = 8.1 Hz, 2H), 7.17 (d, J = 7.9 Hz, 2H), 4.87 (q, J = 6.5 Hz, 1H), 2.35 (s, 3H), 1.72 (s, 1H), 1.49 (d, J = 6.5 Hz, 3H).
^{13}C NMR (126 MHz, $CDCl_3$) (δ, ppm): 143.0, 137.3, 129.3, 125.5, 70.4, 25.2, 21.2.

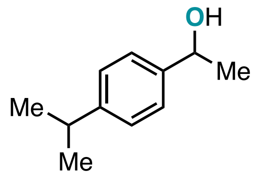

1-(4-isopropylphenyl)ethan-1-ol (2t)
Prepared according to general procedure A (18 hours). The title compound was isolated via flash chromatography (gradient 95:5 – 8:2 hexanes/EtOAc) as a light-yellow oil (120 mg, 61% yield). All analytical data for **2t** was in accordance with literature data.[23]

^{1}H NMR (400 MHz, $CDCl_3$) (δ, ppm): 7.31 (d, J = 8.1 Hz, 2H), 7.22 (d, J = 8.1 Hz, 2H), 4.88 (q, J = 6.4 Hz, 1H), 2.91 (hept, J = 6.9 Hz, 1H), 1.71 (s, 1H), 1.50 (d, J = 6.5 Hz, 3H), 1.25 (d, J = 6.9 Hz, 6H).
^{13}C NMR (101 MHz, $CDCl_3$) (δ, ppm): 148.4, 143.3, 126.7, 125.6, 70.4, 34.0, 25.1, 24.2.

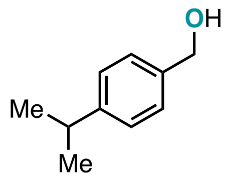

(4-isopropylphenyl)methanol (2u)
Prepared according to general procedure A (18 hours). The title compound was isolated via flash chromatography (85:15 hexanes/EtOAc) as a colorless oil (89 mg, 60% yield). All analytical data for **2u** was in accordance with literature data.[24]

^{1}H NMR (500 MHz, $CDCl_3$) (δ, ppm): 7.30 (d, J = 8.2 Hz, 2H), 7.23 (d, J = 8.2 Hz, 2H), 4.66 (s, 2H), 2.92 (hept, J = 6.9 Hz, 1H), 1.63 (s, 1H), 1.26 (d, J = 7.0 Hz, 6H).
^{13}C NMR (126 MHz, $CDCl_3$) (δ, ppm): 148.7, 138.5, 127.3, 126.8, 65.5, 34.0, 24.1.

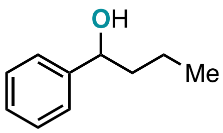

1-phenylbutan-1-ol (2v)
Prepared according to general procedure A (18 hours). The title compound was isolated via flash chromatography (gradient 95:5 – 8:2 hexanes/EtOAc) as a colorless oil (83 mg, 55% yield). All analytical data for **2v** was in accordance with literature data.[24]

^{1}H NMR (500 MHz, $CDCl_3$) (δ, ppm): 7.39 – 7.32 (m, 4H), 7.31 – 7.27 (m, 1H), 4.68 (dd, J = 7.6, 5.8 Hz, 1H), 1.88 – 1.75 (m, 2H), 1.74 – 1.66 (m, 1H), 1.57 – 1.36 (m, 1H), 1.36 – 1.28 (m, 1H), 0.94 (t, J = 7.4 Hz, 3H).
^{13}C NMR (126 MHz, $CDCl_3$) (δ, ppm): 145.1, 128.6, 127.6, 126.0, 74.6, 41.4, 19.2, 14.1.

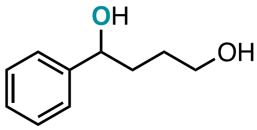

1-phenylbutane-1,4-diol (2w)
Prepared according to general procedure A (18 hours). The title compound was isolated via flash chromatography (1:1 hexanes/EtOAc) as a light-yellow oil (109 mg, 66% yield). All analytical data for **2w** was in accordance with literature data.[25]

^{1}H NMR (500 MHz, $CDCl_3$) (δ, ppm): 7.38 – 7.31 (m, 4H), 7.33 – 7.23 (m, 1H), 4.73 (td, J = 6.4, 1.8 Hz, 1H), 3.75 – 3.63 (m, 2H), 2.32 (s, 2H), 1.87 (qd, J = 7.2, 1.3 Hz, 2H), 1.77 – 1.59 (m, 2H).
^{13}C NMR (126 MHz, $CDCl_3$) (δ, ppm): 144.8, 128.6, 127.7, 125.9, 74.6, 63.0, 36.3, 29.3.

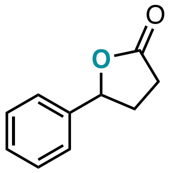

5-phenyldihydrofuran-2(3H)-one (2x)
Prepared according to general procedure A (18 hours). The title compound was isolated via flash chromatography (9:1 hexanes/EtOAc) as a colorless oil (41 mg, 25% yield). All analytical data for **2x** was in accordance with literature data.[26]

^{1}H NMR (500 MHz, $CDCl_3$) (δ, ppm): 7.44 – 7.36 (m, 2H), 7.34 (td, J = 7.0, 1.3 Hz, 3H), 5.52 (dd, J = 7.9, 6.4 Hz, 1H), 2.74 – 2.60 (m, 3H), 2.27 – 2.12 (m, 1H).
^{13}C NMR (126 MHz, $CDCl_3$) (δ, ppm): 176.8, 139.4, 128.8, 128.5, 125.3, 81.2, 31.0, 28.9.

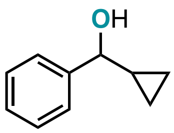

cyclopropyl(phenyl)methanol (2y)
Prepared according to general procedure A (18 hours). The title compound was isolated via flash chromatography (gradient 95:5 – 8:2 hexanes/EtOAc) as a colorless oil (91 mg, 62% yield). All analytical data for **2y** was in accordance with literature data.[27]

^{1}H NMR (500 MHz, $CDCl_3$) (δ, ppm): 7.50 – 7.40 (m, 2H), 7.36 (m, 2H), 7.32 – 7.27 (m, 1H), 4.02 (d, J = 8.3 Hz, 1H), 1.99 (s, 1H), 1.23 (qt, J = 8.2, 4.9 Hz, 1H), 0.79 – 0.60 (m, 1H), 0.57 (ddd, J = 8.5, 5.5, 4.4 Hz, 1H), 0.56 – 0.44 (m, 1H), 0.38 (dq, J = 9.7, 4.9 Hz, 1H).
^{13}C NMR (126 MHz, $CDCl_3$) (δ, ppm): 143.9, 128.5, 127.7, 126.2, 78.7, 19.3, 3.7, 3.0.

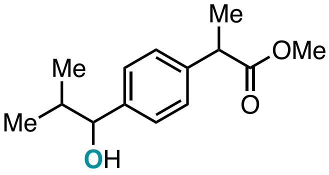

methyl 2-(4-(1-hydroxy-2-methylpropyl)phenyl)propanoate (2z)
Prepared according to general procedure A (18 hours). The title compound was isolated via flash chromatography (gradient 95:5 – 8:2 hexanes/EtOAc) as a colorless oil (182 mg, 77% yield). All analytical data for **2z** was in accordance with literature data.[28]

^{1}H NMR (400 MHz, $CDCl_3$) (δ, ppm): 7.26 (s, 4H), 4.34 (d, J = 6.8 Hz, 1H), 3.72 (q, J = 7.2 Hz, 1H), 3.65 (s, 3H), 2.02 – 1.85 (m, 2H), 1.49 (d, J = 7.2 Hz, 3H), 0.99 (d, J = 6.6 Hz, 3H), 0.80 (d, J = 6.8 Hz, 3H).
^{13}C NMR (101 MHz, $CDCl_3$) (δ, ppm): 175.2, 142.7, 139.7, 127.4, 127.0, 79.8, 52.1, 45.2, 35.3, 19.1, 18.7, 18.3.

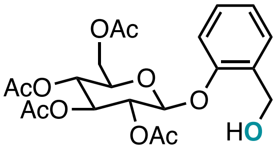

(2R,3R,4S,5R,6S)-2-(acetoxymethyl)-6-(2-(hydroxymethyl)phenoxy)tetrahydro-2H-pyran-3,4,5-triyl triacetate (2aa)
Prepared according to general procedure A (24 hours). ^{1}H NMR yield determined using CH_2Br_2 as an external standard (Yield = 26%). All analytical data for **2aa** was in accordance with literature data.[29]

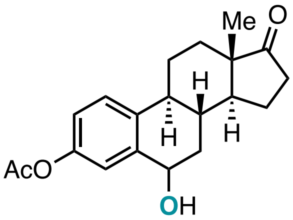

(8R,9S,13S,14S)-6-hydroxy-13-methyl-17-oxo-7,8,9,11,12,13,14,15,16,17-decahydro-6H-cyclopenta[a]phenanthren-3-yl acetate (2ab)
Prepared according to general procedure A (24 hours). ^{1}H NMR yield determined using CH_2Br_2 as an external standard (Yield = 26%). All analytical data for **2ab** was in accordance with literature data as a mixture of diastereomers (2:1).[30]

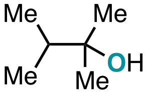

2,3-dimethylbutan-2-ol (2ac)
Prepared according to general procedure B. ^{1}H NMR yield determined using CH_2Br_2 as an external standard (Yield = 91%). All analytical data for **2ac** was in accordance with literature data.[31]

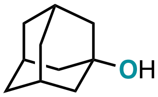

Adamantan-1-ol (2ad)
Prepared according to general procedure B. The title compound was isolated via flash column chromatography (0 – 2 % MeOH/DCM) as a white crystalline solid (69 mg, 90% yield). All analytical data for **2ad** was in accordance with literature data.[32]

^{1}H NMR (400 MHz, $CDCl_3$) (δ, ppm): 2.17 – 2.10 (m, 3H), 1.71 (d, J = 2.9 Hz, 6H), 1.66 – 1.55 (m, 6H), 1.41 (s, br, 1H).
^{13}C NMR (100 MHz, $CDCl_3$) (δ, ppm): 68.4, 45.5, 36.2, 30.9.

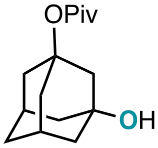

3-hydroxyadamantan-1-yl pivalate (2ae)
Prepared according to general procedure B. The title compound was isolated via flash column chromatography (0 – 2 % MeOH/DCM) as a white crystalline solid (187 mg, 74% yield).

^{1}H NMR (500 MHz, $CDCl_3$) (δ, ppm): 2.29 (p, J = 3.2 Hz, 2H), 2.06 (s, 2H), 2.03 – 1.94 (m, 4H), 1.70 – 1.61 (m, 4H), 1.56 – 1.45 (m, 2H), 1.12 (s, 9H).
^{13}C NMR (100 MHz, $CDCl_3$) (δ, ppm): 177.9, 80.6, 70.3, 49.1, 44.1, 39.9, 39.3, 34.9, 31.3, 27.3.
HRMS (ESI-TOF): calcd. for $C_{15}H_{25}O_3$ $[M+H]^+$ 253.1798, found 253.1804.

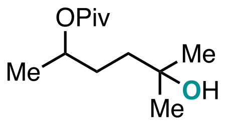

5-hydroxy-5-methylhexan-2-yl pivalate (2af)

Prepared according to general procedure B. The title compound was isolated via flash column chromatography (0 – 2 % MeOH/DCM) as a colorless oil (44 mg, 63% yield).

^{1}H NMR (400 MHz, $CDCl_3$) (δ, ppm): 4.92 – 4.79 (m, 1H), 1.70 – 1.32 (m, 4H), 1.21 (s, 6H), 1.20 (d, J = 11.4 Hz, 3H), 1.18 (s, 9H).
^{13}C NMR (100 MHz, $CDCl_3$) (δ, ppm): 178.4, 70.9, 70.8, 39.4, 38.9, 30.9, 29.5, 29.3, 27.3, 20.0.
HRMS (ESI-TOF): calcd. for $C_{12}H_{24}O_3Na$ $[M+Na]^+$ 239.1618, found 239.1608.

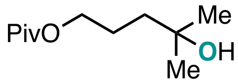

4-hydroxy-4-methylpentyl pivalate (2ag)

Prepared according to general procedure B on a 3.5 mmol scale. (see page 47)

^{1}H NMR (500 MHz, $CDCl_3$) (δ, ppm): 4.07 (td, J = 6.6, 2.2 Hz, 2H), 1.76 – 1.66 (m, 2H), 1.55 – 1.48 (m, 2H), 1.23 (s, 6H), 1.19 (s, 9H).
^{13}C NMR (126 MHz, $CDCl_3$) (δ, ppm):178.8, 70.8, 64.8, 40.1, 38.9, 29.4, 27.3, 24.0.

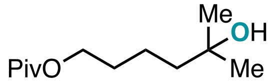

5-hydroxy-5-methylhexyl pivalate (2ah)

Prepared according to general procedure B. The title compound was isolated via flash column chromatography (0 – 2 % MeOH/DCM) as a colorless oil (46 mg, 85% yield).

^{1}H NMR (400 MHz, $CDCl_3$) (δ, ppm): 4.07 (t, J = 6.5 Hz, 2H), 1.64 (p, J = 6.7 Hz, 2H), 1.52 – 1.35 (m, 4H), 1.30 (s, br, 1H), 1.21 (s, 6H), 1.19 (s, 9H).
^{13}C NMR (100 MHz, $CDCl_3$) (δ, ppm): 178.8, 71.0, 64.3, 43.5, 38.9, 29.4, 29.2, 27.3, 20.8.
HRMS (ESI-TOF): calcd. for $C_{12}H_{24}O_3$ $[M+H]^+$ 217.1798, found 217.1788.

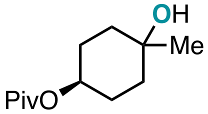

4-hydroxy-4-methylcyclohexyl pivalate (2ai)
Prepared according to general procedure B. The title compound was isolated via flash column chromatography (0 - 2% MeOH/DCM) as a colorless oil (69 mg, 64% yield). All analytical data was in accordance with literature data.[34]

^{1}H NMR (400 MHz, $CDCl_3$) (δ, ppm): 4.70 (tt, *J* = 8.5, 4.6 Hz, 1H), 1.82 – 1.64 (m, 6H), 1.50 (ddt, *J* = 13.4, 8.6, 4.8 Hz, 2H), 1.24 (s, 3H), 1.18 (s, 9H).
^{13}C NMR (100 MHz, $CDCl_3$) (δ, ppm): 178.3, 71.4, 68.9, 38.9, 36.6, 29.7, 27.3.

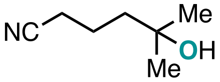

5-hydroxy-5-methylhexanenitrile (2aj)
Prepared according to general procedure B. The title compound was isolated via flash column chromatography (0 – 2% MeOH/DCM) as a gold oil (29 mg, 45% yield). All analytical data for **2aj** was in accordance with literature data.[35]

^{1}H NMR (400 MHz, $CDCl_3$) (δ, ppm): 2.39 (t, *J* = 7.1 Hz, 2H), 1.82 – 1.73 (m, 2H), 1.63 – 1.57 (m, 2H), 1.24 (s, 6H).
^{13}C NMR (100 MHz, $CDCl_3$) (δ, ppm): 119.9, 70.6, 42.5, 30.0, 21.1, 17.7.
LRMS (ESI) calcd. for $C_7H_{14}NO$ $[M+H]^+$ 128.10, found 128.15.

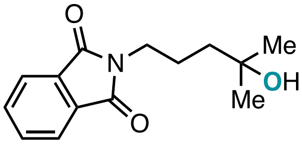

2-(4-hydroxy-4-methylpentyl)isoindoline-1,3-dione (2ak)
Prepared according to general procedure B. The title compound was isolated via flash column chromatography (0 – 5% EtOAc/hexanes) as a colorless oil (170 mg, 69% yield). All analytical data was in accordance with literature data.[36]

^{1}H NMR (400 MHz, $CDCl_3$) (δ, ppm): 7.84 (dd, *J* = 5.4, 3.1 Hz, 2H), 7.71 (dd, *J* = 5.5, 3.0 Hz, 2H), 3.72 (t, *J* = 7.2 Hz, 2H), 1.83 – 1.73 (m, 2H), 1.57 – 1.46 (m, 2H), 1.43 (s, br, 1H), 1.21 (s, 6H).
^{13}C NMR (100 MHz, $CDCl_3$) (δ, ppm): 168.6, 134.1, 132.3, 123.4, 70.8, 40.7, 38.5, 29.4, 23.9.

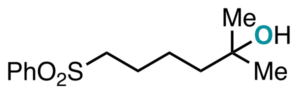

2-methyl-6-(phenylsulfonyl)hexan-2-ol (2al)
Prepared according to general procedure B. The title compound was isolated via flash column chromatography (0 – 5% MeOH/DCM) as a gold oil (92 mg, 36% yield).

^{1}H NMR (400 MHz, $CDCl_3$) (δ, ppm): 8.07 – 7.80 (m, 2H), 7.73 – 7.62 (m, 1H), 7.61 – 7.51 (m, 2H), 3.42 – 2.75 (m, 2H), 1.97 – 1.59 (m, 2H), 1.57 – 1.37 (m, 4H), 1.17 (s, 6H).
^{13}C NMR (100 MHz, $CDCl_3$) (δ, ppm): 139.3, 133.8, 129.4, 128.2, 70.8, 56.4, 43.2, 29.4, 23.3, 23.2.
HRMS (ESI-TOF): calcd. for $C_{13}H_{20}O_3SNa$ $[M+Na]^+$ 279.1025, found 279.1032.

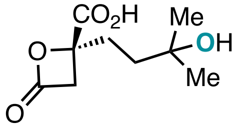

(*R*)-2-(3-hydroxy-3-methylbutyl)-4-oxooxetane-2-carboxylic acid (2am)
Prepared according to general procedure B. The title compound was isolated via flash column chromatography (2:1:97 AcOH/MeOH/DCM) as a brown oil (30.9 mg, 31% yield).

^{1}H NMR (400 MHz, Acetic Acid-d$_4$) (δ, ppm): 3.12 (d, J = 16.3 Hz, 1H), 2.82 (d, J = 15.7 Hz, 1H), 1.47 – 1.20 (m, 4H), 0.90 (s, 6H).
^{13}C NMR (100 MHz, Acetic Acid-d$_4$) (δ, ppm): 177.3, 166.6, 130.8, 86.7, 76.6, 37.3, 33.4, 29.6, 23.4.
HRMS (ESI-TOF): calcd. for $C_9H_{14}O_5Na$ $[M+Na]^+$ 225.0733, found 225.0738.

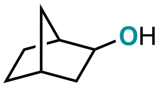

Bicyclo[2.2.1]heptan-2-ol (2an)
Prepared according to general procedure B. The title compound was isolated via flash column chromatography (0 - 2% MeOH/DCM) as a white crystalline solid (70 mg, 63% yield). All analytical data was in accordance with literature data.[32]

^{1}H NMR (400 MHz, $CDCl_3$) (δ, ppm): 3.76 (dt, J = 6.9, 1.6 Hz, 1H), 2.27 – 2.22 (m, 1H), 2.14 (d, J = 4.6 Hz, 1H), 1.69 – 1.63 (m, 1H), 1.57 (m, 1H), 1.44 – 1.37 (m, 2H), 1.28 (m, 1H), 1.12 (ddd, J = 9.8, 2.6, 1.4 Hz, 1H), 1.06 – 0.97 (m, 2H).
^{13}C NMR (100 MHz, $CDCl_3$) (δ, ppm): 75.1, 44.5, 42.5, 35.6, 34.5, 28.2, 24.5.

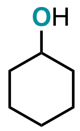

Cyclohexanol (2ao)
Prepared according to general procedure B. The title compound was isolated via flash column chromatography (0 - 2% MeOH/DCM) as a light-yellow oil (140 mg, 70% yield). All analytical data was in accordance with literature data.[19]

^{1}H NMR (400 MHz, $CDCl_3$) (δ, ppm): 3.61 (dt, *J* = 8.6, 4.1 Hz, 1H), 1.89 (td, *J* = 7.2, 6.2, 3.2 Hz, 2H), 1.77 – 1.69 (m, 2H), 1.58 – 1.42 (m, 2H), 1.33 – 1.11 (m, 5H).
^{13}C NMR (100 MHz, $CDCl_3$) (δ, ppm): 70.5, 35.7, 25.6, 24.3.

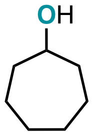

Cycloheptanol (2ap)
Prepared according to general procedure B. The title compound was isolated via flash column chromatography (1% MeOH/DCM) as a light-yellow oil (154 mg, 67% yield). All analytical data was in accordance with literature data.[32]

^{1}H NMR (400 MHz, CD_2Cl_2) (δ, ppm): 3.80 (septet, *J* = 8.4, 4.3 Hz, 1H), 1.93 – 1.82 (m, 2H), 1.71 – 1.46 (m, 7H), 1.39 (m, 3H).
^{13}C NMR (126 MHz, CD_2Cl_2) (δ, ppm): 73.17, 38.24, 28.72, 23.24.

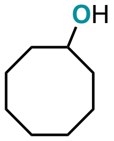

Cyclooctanol (2aq)
Prepared according to general procedure B. The title compound was isolated via flash column chromatography (0 - 2% MeOH/DCM) as a light-yellow oil (94 mg, 73% yield). All analytical data was in accordance with literature data.[19]

^{1}H NMR (400 MHz, $CDCl_3$) (δ, ppm): 3.83 (dt, *J* = 8.0, 3.8 Hz, 1H), 1.85 – 1.77 (m, 2H), 1.73 – 1.61 (m, 4H), 1.58 – 1.40 (m, 9H).
^{13}C NMR (100 MHz, $CDCl_3$) (δ, ppm): 72.3, 34.8, 27.5, 25.3, 22.8.

Mechanism studies

Kinetic isotope effect studies

Benzylic C–H hydroxylation. To a 0.5-dram oven-dried vial equipped with a Teflon stir bar was added 2-chloro-4-nitro-pyridine (15.9 mg, 1.00 equiv., 0.100 mmol), and 5.00 equiv. (0.500 mmol) of each of the C(sp^3)–H and C(sp^3)–D substrates (competition experiment) or either the C(sp^3)–H substrate or the C(sp^3)–D substrate (parallel experiment). Then, the vial was purged with N_2 flow for 5 min and DCM (1 mL), and 1,1,1,3,3,3-Hexafluoropropan-2-ol (336 mg, 210 µL, 20 equiv., 2.00 mmol) were added. The mixture was stirred at 800 rpm under irradiation by one 390 nm Kessil lamp. After 1 h, the irradiation was stopped, and pentadecane (21.2 mg, 26.9 µL, 1.00 equiv., 0.100 mmol) was added and the reaction was analyzed by GCMS using pentadecane as external standard. Each KIE measurement is the average of two independent experiments.

Intermolecular KIE experiments

Competition experiments

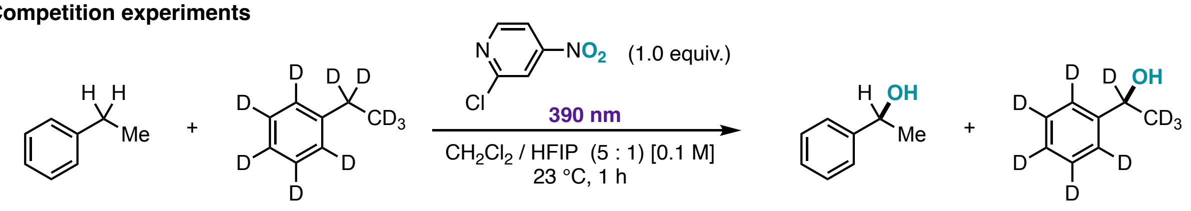

1g (5.0 equiv.) + **1g-d$_{10}$** (5.0 equiv.) → **2g** + **2g-d$_9$**

KIE = 1.70 (competition)

Parallel experiments

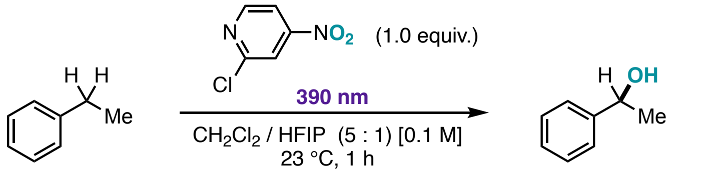

1g (5.0 equiv.) → **2g**

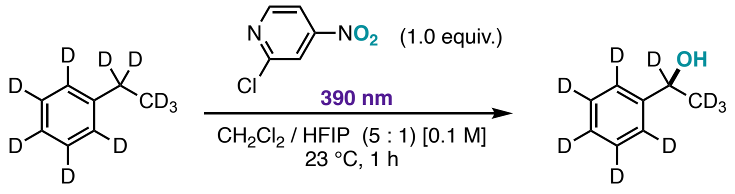

1g-d$_{10}$ (5.0 equiv.) → **2g-d$_9$**

KIE = 1.60 (parallel)

Figure S3: Reactions of **3** with **1g** and **1g-d$_{10}$** to determine KIE.

Competition	IS	Alcohol-H	Alcohol-D	Alc-H / IS	Alc-D / IS	KIE
**Run 1**	1454293	96836	56388	0.066586307	0.03877348	**1.72**
**Run 2**	1177476	96157	57403	0.08166366	0.048750887	**1.68**
					**Average**	**1.70**
**Parallel**	IS	Alcohol-H	Alcohol-D	Alc-H / IS	Alc-D / IS	**KIE**
**Run 1**	1254318	115012	-	0.091692856	-	
**Run 2**	1222906	111641	-	0.091291563	-	
**Run 1**	1421717	-	76658	-	0.05391931	**1.70**
**Run 2**	1593672	-	96954	-	0.06083686	**1.50**
					**Average**	**1.60**

Table S9: Experimental KIE data for the reaction of **3** with **1g** and **1g-d$_{10}$**.

Unactivated C–H hydroxylation. To a 0.5-dram oven-dried vial equipped with a Teflon stir bar was added 3,5-bis(trifluoromethyl)nitrobenzene (25.9 mg, 1.00 equiv., 0.100 mmol), and 5.00 equiv. (0.500 mmol) of each of the C(sp^3)–H and C(sp^3)–D substrates (competition experiment) or either the C(sp^3)–H substrate or the C(sp^3)–D substrate (parallel experiment). Then, the vial was purged with N_2 flow for 5 min and 1,1,1,3,3,3-Hexafluoropropan-2-ol (33.6 mg, 21.0 µL, 2.00 equiv., 0.200 mmol) were added. The mixture was stirred at 800 rpm under irradiation by one 390 nm Kessil lamp. After 2 h, the irradiation was stopped, the mixture was diluted with DCM (1 mL) and pentadecane (21.2 mg, 26.9 µL, 1.00 equiv., 0.100 mmol) was added and the reaction was analyzed by GCMS using pentadecane as external standard. Each KIE measurement is the average of two independent experiments.

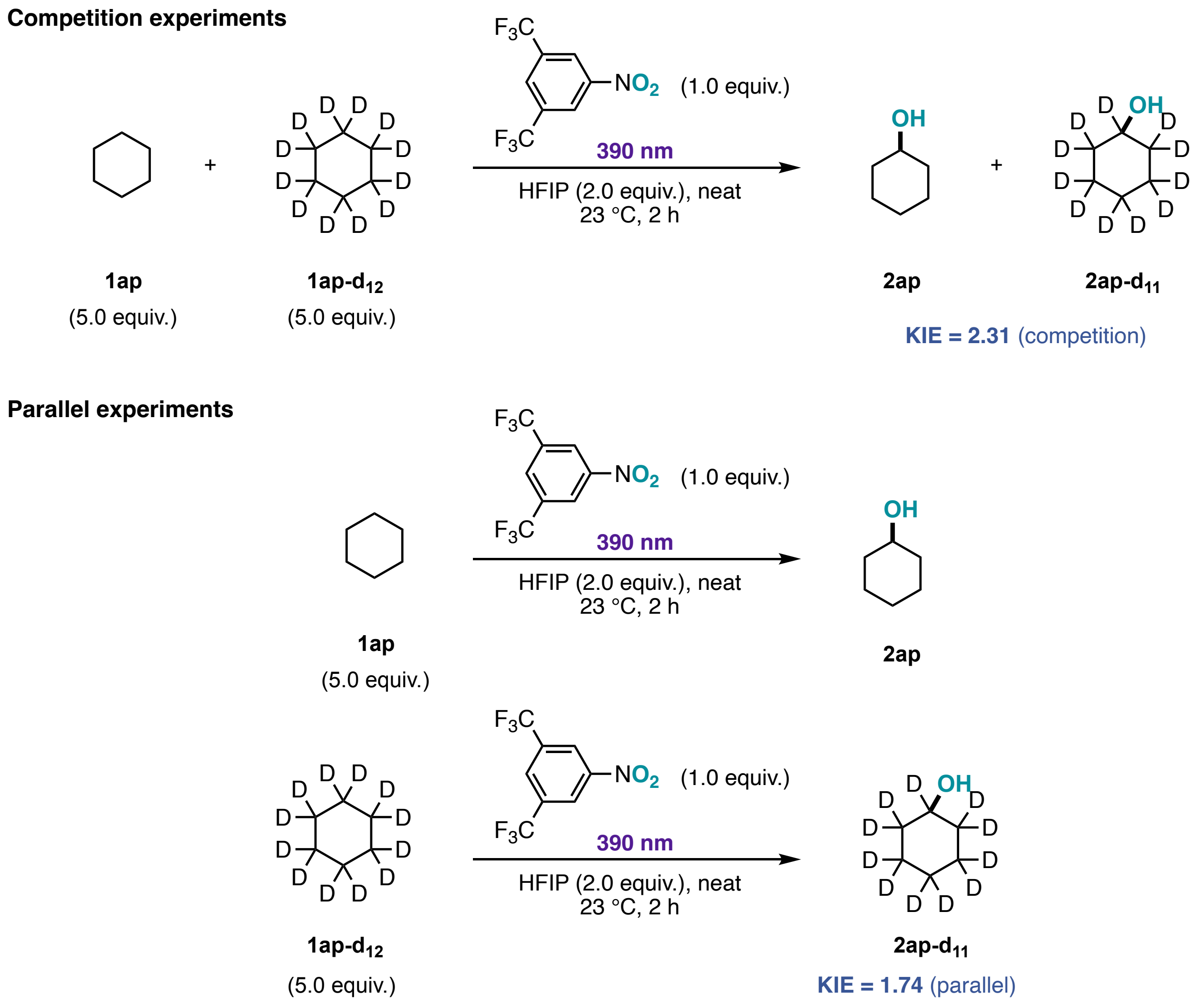

Figure S4: Reactions of **4** with **1ap** and **1ap-d$_{12}$** to determine KIE.

**Competition**	IS	Alcohol-H	Alcohol-D	Alc-H / IS	Alc-D / IS	**KIE**
**Run 1**	1263692	130615	58342	0.103359838	0.046167895	2.24
**Run 2**	1583525	149446	62714	0.094375523	0.039604048	2.38
					**Average**	**2.31**
**Parallel**	IS	Alcohol-H	Alcohol-D	Alc-H / IS	Alc-D / IS	**KIE**
**Run 1**	1417503	196804	-	0.138838507	-	
**Run 2**	1209083	213205	-	0.176336116	-	
**Run 1**	1690591	-	157334	-	0.093064496	1.89
**Run 2**	1040480	-	116228	-	0.111706136	1.58
					**Average**	**1.74**

Table S10: Experimental KIE data for the reaction of **4** with **1ap** and **1ap-d$_{12}$**.

Intramolecular Benzylic C–H hydroxylation. To a 0.5 dram oven-dried vial equipped with a Teflon stir bar was added 2-chloro-4-nitro-pyridine (15.9 mg, 1.00 equiv., 0.100 mmol), and (propyl-1-*d*)benzene (60.6 mg, 5.00 equiv., 0.500 mmol). Then, the vial was purged with N_2 flow for 5 min and DCM (80 µL) and 1,1,1,3,3,3-hexafluoroisopropan-2-ol (20 µL) were added. The mixture was stirred at 800 rpm under irradiation by one 390 nm Kessil lamp. After 1 h, the reaction was stopped, and the solvent was removed in vacuo. The crude mixture was dissolved in $CDCl_3$ and the reaction was analyzed by ^1H NMR.

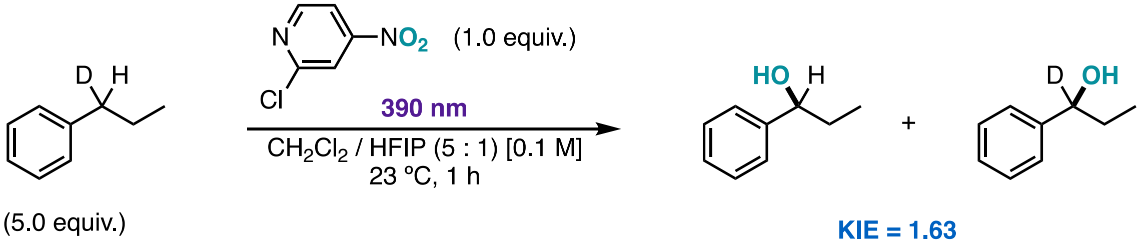

Figure S5: Reaction of **3** with (propyl-1-*d*)benzene to determine KIE

^1H NMR (500 MHz, $CDCl_3$) of Crude Intramolecular Benzylic C-H KIE Study

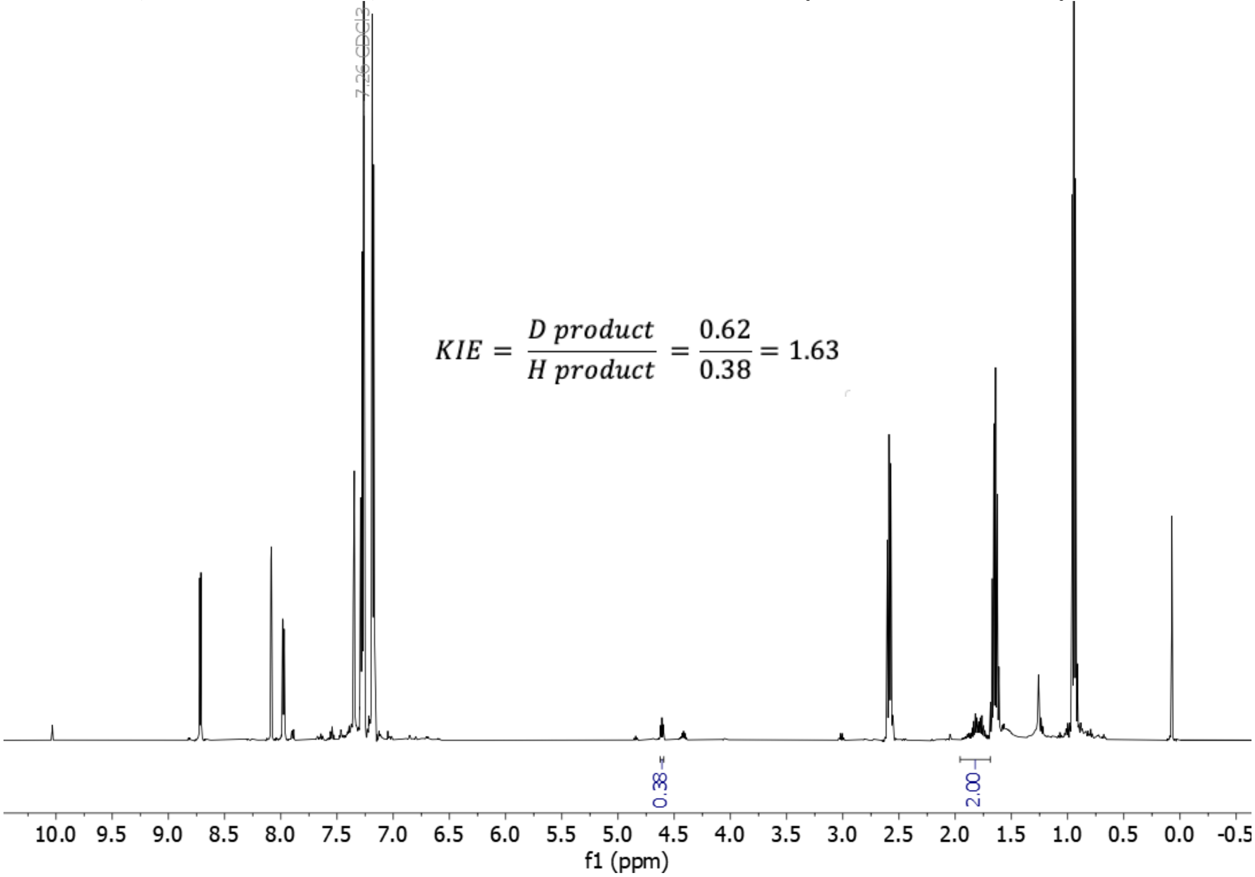

Radical clock studies

Synthesis of alcohol **S1**, according to literature procedure.[37]

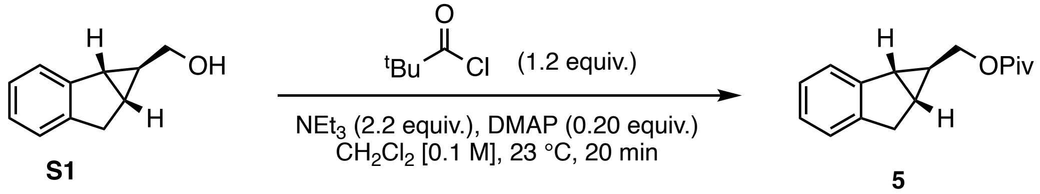

((1*S*,1a*S*,6a*R*)-1,1a,6,6a-tetrahydrocyclopropa[*a*]inden-1-yl)methyl pivalate (5)

A solution of alcohol **S1** (441 mg, 1.00 equiv., 2.75 mmol), DMAP (67.2 mg, 0.200 equiv., 550 µmol) in anhydrous DCM (20 mL) was allowed to stir at room temperature for 5 minutes, then triethylamine (626 mg, 862 µL, 2.25 equiv., 6.19 mmol) and pivaloyl chloride (398 mg, 407 µL, 1.2 equiv., 3.30 mmol) were added. The reaction was allowed to stir at room temperature for 20 mins, with monitoring by GCMS. Next, H_2O (20 mL) was added, and the reaction was extracted with DCM (3 × 20 mL). The organic layer was washed with 2 M NaOH then dried (Na_2SO_4) and concentrated. The title compound was isolated via flash column chromatography (0 – 10% EtOAc/Hexanes) as a colorless oil (438 mg, 65% yield).

^{1}H NMR (500 MHz, $CDCl_3$) (δ, ppm): 7.29 – 7.26 (m, 1H), 7.16 – 7.04 (m, 3H), 4.09 (dd, *J* = 11.5, 6.6 Hz, 1H), 3.99 (dd, *J* = 11.5, 7.5 Hz, 1H), 3.19 (dd, *J* = 17.1, 6.8 Hz, 1H), 2.97 (d, *J* = 17.4 Hz, 1H), 2.40 (dt, *J* = 6.5, 2.2 Hz, 1H), 1.87 – 1.80 (m, 1H), 1.22 (s, 9H), 0.84 – 0.77 (m, 1H).
^{13}C NMR (126 MHz, $CDCl_3$) (δ, ppm): 178.8, 145.4, 142.3, 126.2, 125.9, 125.4, 123.6, 66.2, 39.0, 35.3, 29.8, 28.6, 27.3, 21.6.
HRMS (ESI-TOF) Calcd. for $C_{16}H_{20}O_2$ $[M]^+$: 244.1463, Found: 244.1472.

^{1}H NMR (500 MHz, $CDCl_3$) of ((1*S*,1a*S*,6a*R*)-1,1a,6,6a-tetrahydrocyclopropa[*a*]inden-1-yl)methyl pivalate (5)

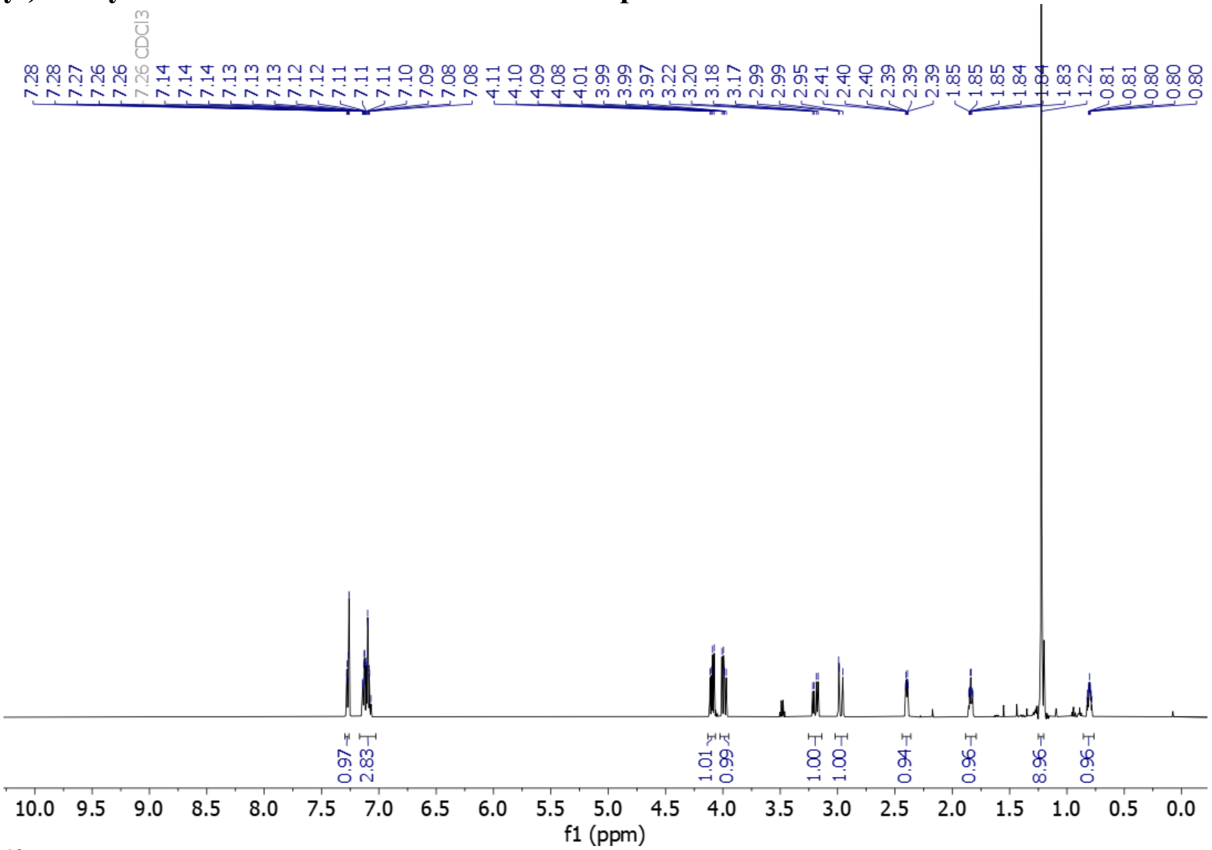

^{13}C NMR (126 MHz, $CDCl_3$) of ((1*S*,1a*S*,6a*R*)-1,1a,6,6a-tetrahydrocyclopropa[*a*]inden-1-yl)methyl pivalate (5)

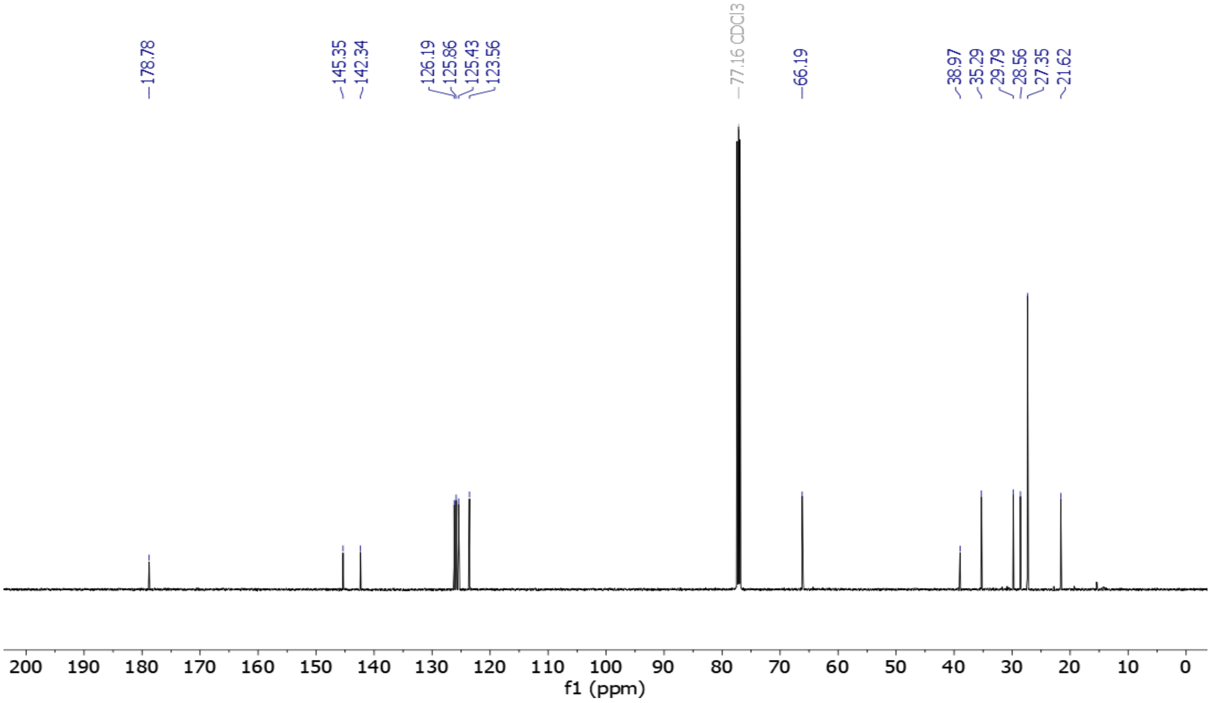

C–H hydroxylation with radical clock, 5. To a 1-dram oven-dried vial equipped with a Teflon stir bar was added 2-chloro-4-nitro-pyridine (47.6 mg, 1.00 equiv., 0.300 mmol) and **5** (73.3 mg, 1.00 equiv., 0.300 mmol). Then, the vial was purged with N_2 flow for 5 min and DCM (3 mL) was added. The mixture was stirred at 800 rpm under irradiation by one 390 nm Kessil lamp. After 20 h, the irradiation was stopped, and the volatiles were removed *in vacuo*. The residue was dissolved in $CDCl_3$ and CH_2Br_2 (52.1 mg, 20.9 µL, 1.00 equiv., 0.300 mmol) was added. The crude ^{1}H NMR spectrum was recorded using CH_2Br_2 as external standard. Next, the volatiles were once more removed *in vacuo*, the residue was dry loaded onto a silica gel column and the mixture was purified using 5 – 25% EtOAc/Hexanes.

The structures of alcohol **6** and ketone **7** were assigned based on their ^{1}H and ^{13}C NMR spectra and the characterization data for naphthalene **8** matched that previously reported in the literature.[38]

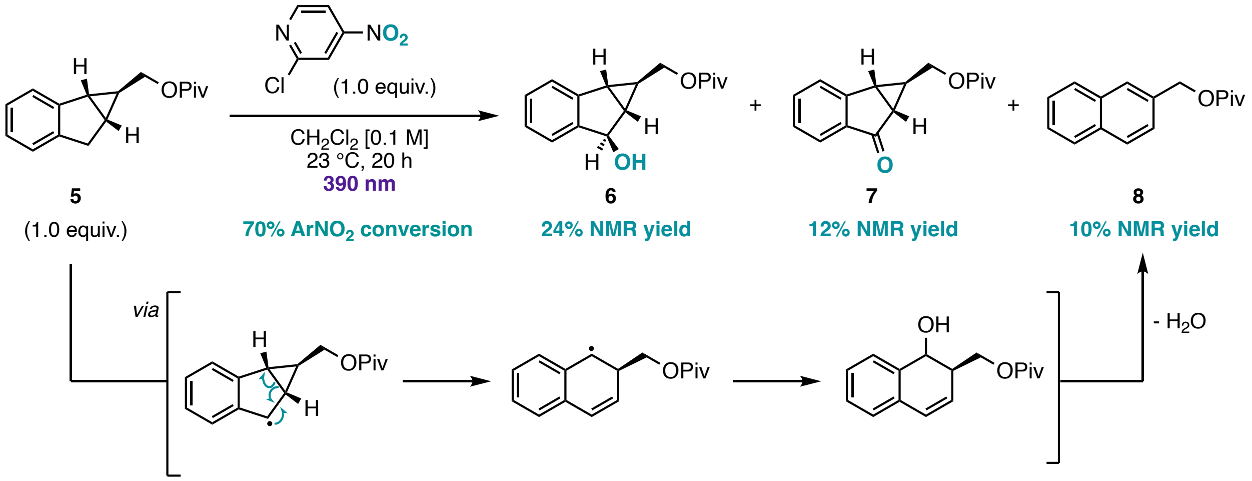

Figure S6: Radical clock studies

^{1}H NMR yield spectrum

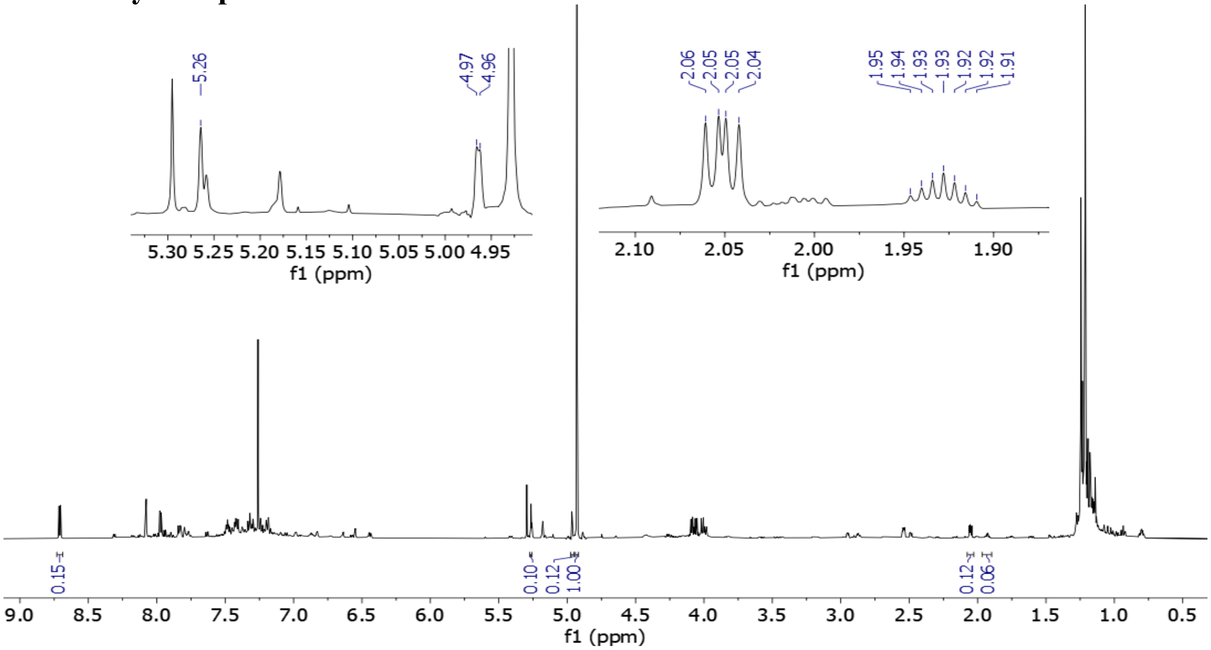

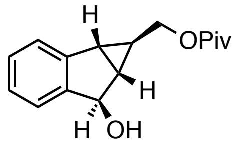

((1*R*,1a*R*,6*R*,6a*R*)-6-hydroxy-1,1a,6,6a-tetrahydrocyclopropa[*a*]inden-1-yl)methyl pivalate (6)

^{1}H NMR (400 MHz, $CDCl_3$) (δ, ppm): 7.44 – 7.39 (m, 1H), 7.33 (d, *J* = 7.8 Hz, 1H), 7.24 (dd, *J* = 7.6, 1.4 Hz, 1H), 7.23 – 7.18 (m, 1H), 4.97 (dd, *J* = 8.8, 1.8 Hz, 1H), 4.07 (dd, *J* = 11.5, 6.4 Hz, 1H), 4.01 (dd, *J* = 11.5, 7.3 Hz, 1H), 2.55 (dt, *J* = 4.9, 2.2 Hz, 1H), 2.06 (dd, *J* = 5.7, 3.7 Hz, 1H), 1.72 (d, *J* = 8.8 Hz, 1H), 1.22 (s, 9H), 0.81 (dddd, *J* = 7.3, 6.3, 3.7, 2.7 Hz, 1H).
^{13}C NMR (126 MHz, $CDCl_3$) (δ, ppm): 181.2, 145.2, 144.0, 128.9, 126.6, 126.1, 123.7, 76.1, 65.1, 39.0, 32.0, 31.4, 31.1, 28.3, 27.3.
HRMS (ESI-TOF) Calcd. for $C_{16}H_{20}O_3$ $[M]^+$: 260.1412, Found: 260.1420.

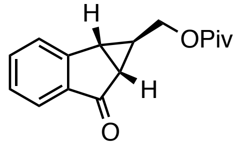

((1*R*,1a*R*,6a*R*)-6-oxo-1,1a,6,6a-tetrahydrocyclopropa[*a*]inden-1-yl)methyl pivalate (7)

^{1}H NMR (400 MHz, $CDCl_3$) (δ, ppm): 7.64 (d, *J* = 7.6 Hz, 1H), 7.48 (td, *J* = 7.4, 2.4 Hz, 1H), 7.42 (d, *J* = 7.6 Hz, 1H), 7.33 – 7.27 (m, 1H), 4.09 (d, *J* = 6.1 Hz, 2H), 2.95 (dd, *J* = 5.1, 3.1 Hz, 1H), 2.49 (dd, *J* = 5.0, 3.2 Hz, 1H), 1.93 (tt, *J* = 6.1, 3.1 Hz, 1H), 1.22 (s, 9H).
^{13}C NMR (100 MHz, $CDCl_3$) (δ, ppm): 201.5, 178.4, 153.3, 134.2, 134.1, 127.4, 124.9, 124.9, 63.6, 44.1, 39.0, 27.4, 27.3, 26.3.
HRMS (ESI-TOF) Calcd. for $C_{16}H_{18}O_3$ $[M]^+$: 258.1256, Found: 258.1249.

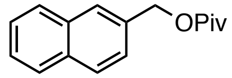

naphthalen-2-ylmethyl pivalate (8)

^{1}H NMR (400 MHz, $CDCl_3$) (δ, ppm): 7.87 – 7.78 (m, 4H), 7.52 – 7.47 (m, 2H), 7.45 (dd, *J* = 8.5, 1.7 Hz, 1H), 5.27 (s, 2H), 1.25 (s, 9H).
HRMS (ESI-TOF) Calcd. for $C_{16}H_{18}O_2Na$ $[M+Na]^+$: 265.1204, Found: 265.1212.

^{1}H NMR (400 MHz, $CDCl_3$) of ((1*R*,1a*R*,6*R*,6a*R*)-6-hydroxy-1,1a,6,6a-tetrahydrocyclopropa[*a*]inden-1-yl)methyl pivalate (6)

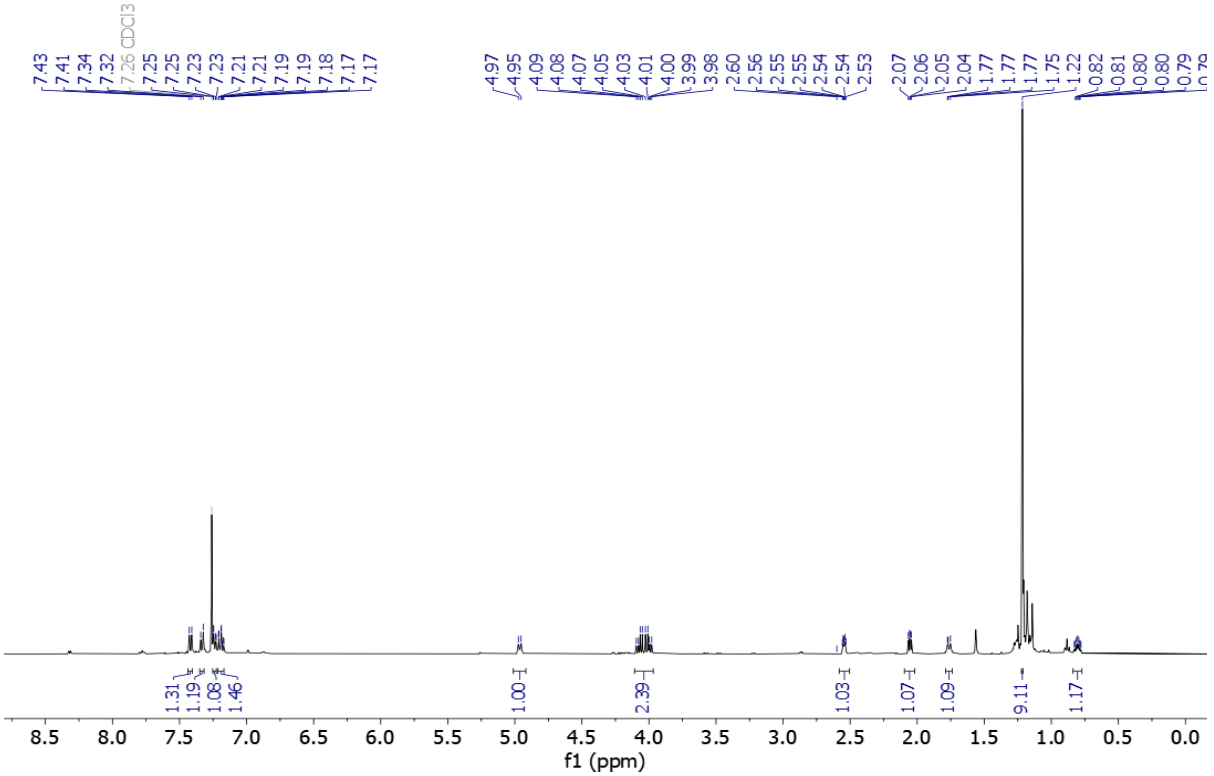

^{13}C NMR (126 MHz, $CDCl_3$) of ((1*R*,1a*R*,6*R*,6a*R*)-6-hydroxy-1,1a,6,6a-tetrahydrocyclopropa[*a*]inden-1-yl)methyl pivalate (6)

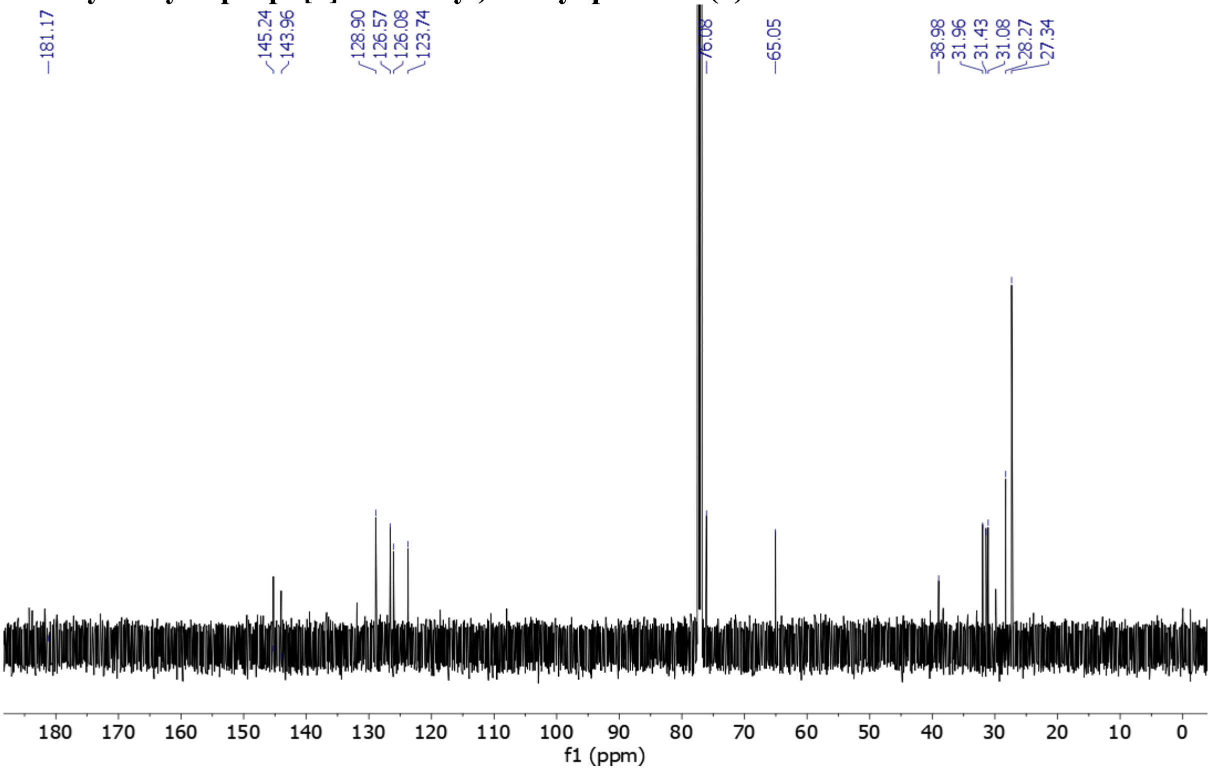

^{1}H NMR (400 MHz, CDCl$_3$) of ((1*R*,1a*R*,6a*R*)-6-oxo-1,1a,6,6a-tetrahydrocyclopropa[*a*]inden-1-yl)methyl pivalate (7)

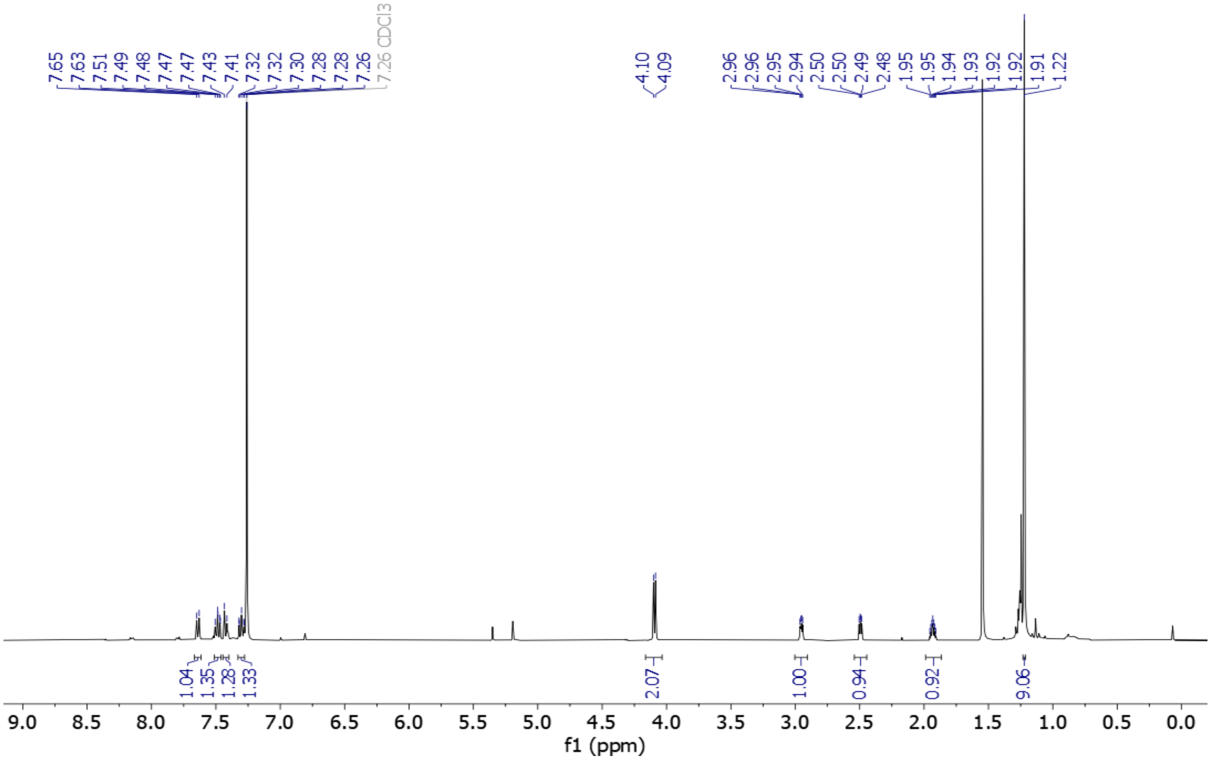

^{13}C NMR (100 MHz, CDCl$_3$) of ((1*R*,1a*R*,6a*R*)-6-oxo-1,1a,6,6a-tetrahydrocyclopropa[*a*]inden-1-yl)methyl pivalate (7)

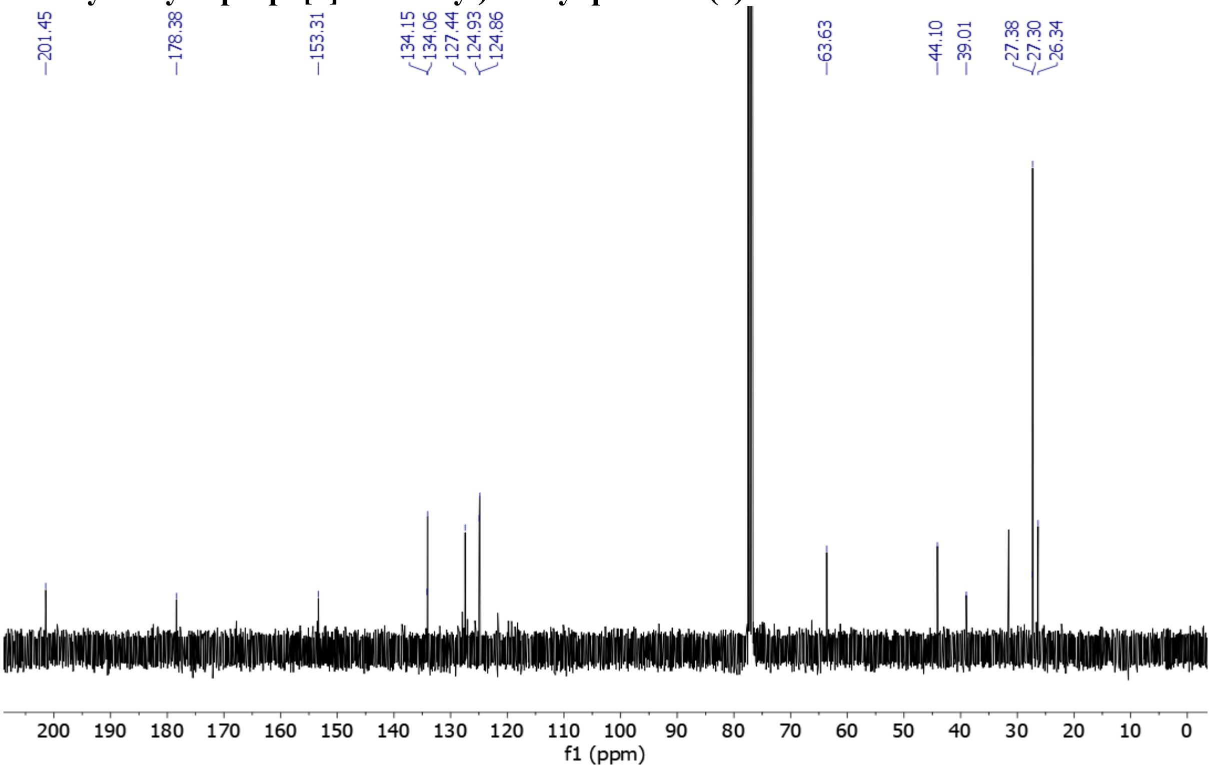

^{13}C DEPT NMR (100 MHz, CDCl$_3$) of ((1*R*,1a*R*,6a*R*)-6-oxo-1,1a,6,6a-tetrahydrocyclopropa[*a*]inden-1-yl)methyl pivalate (7)

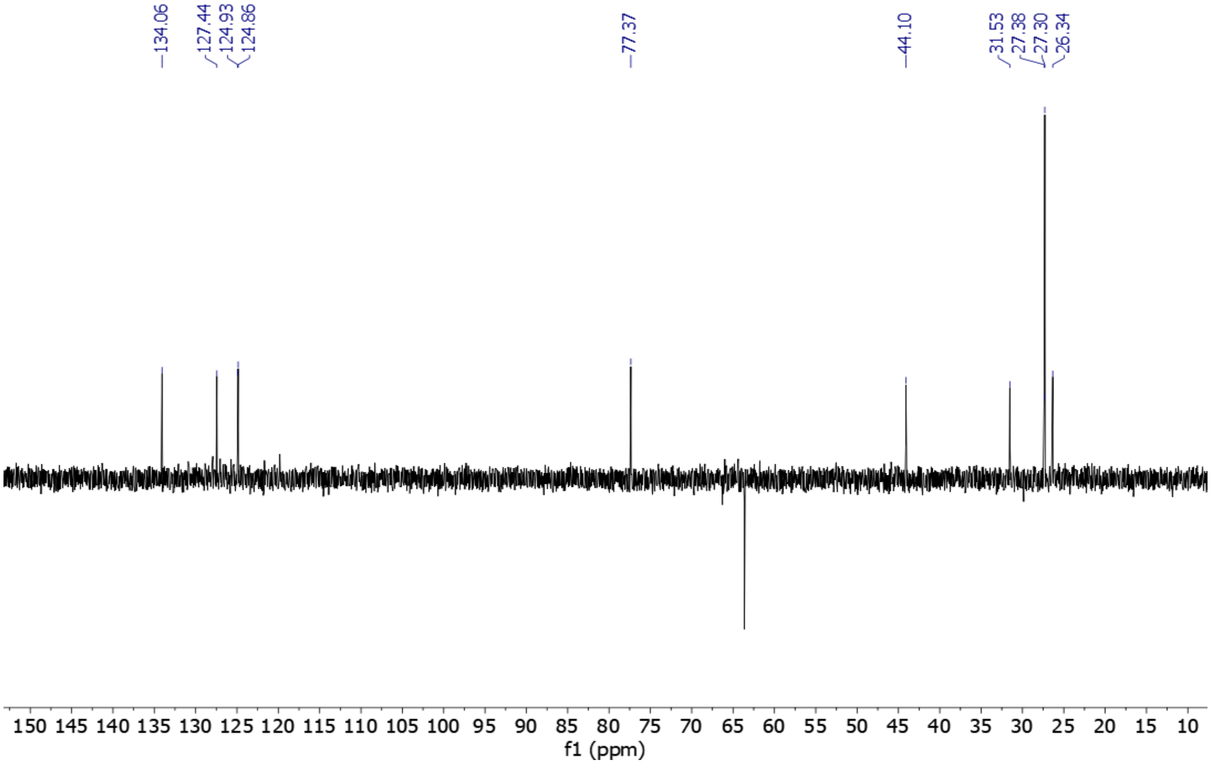

^{1}H NMR (400 MHz, CDCl$_3$) of naphthalen-2-ylmethyl pivalate (8)

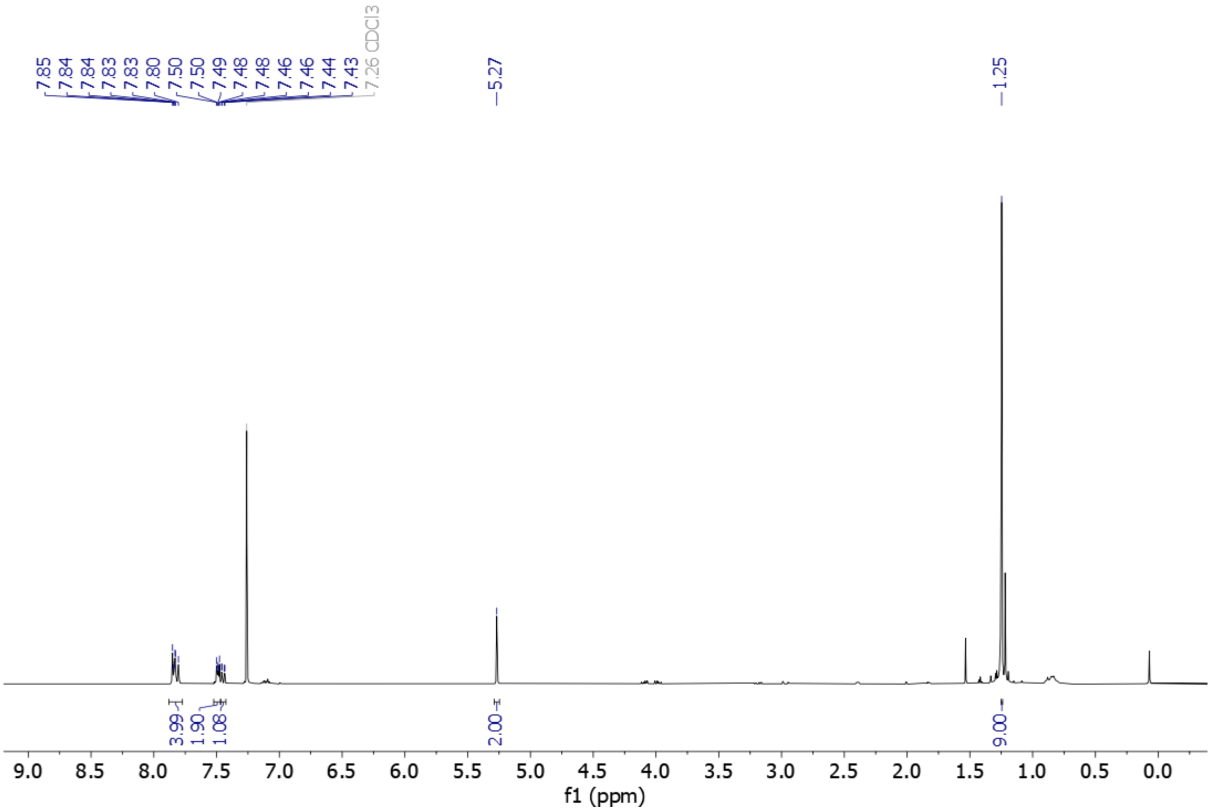

Radical cation probe

Synthesis of 4-ethyl-cyclopropylbenzene **S2**, according to literature procedure.[39]

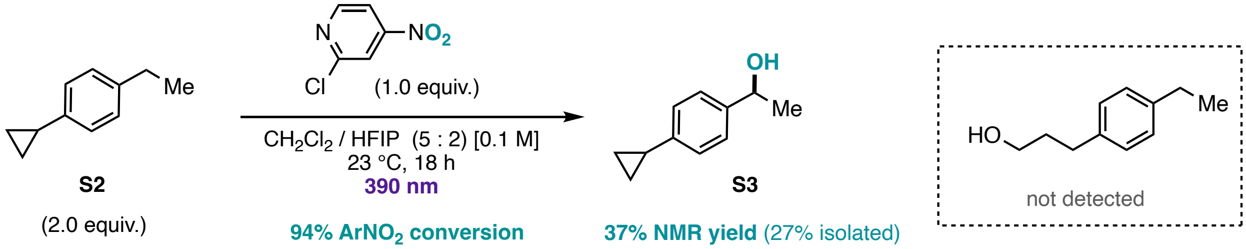

Figure S7: Reaction of radical cation probe **S2** with **3**

Following General Procedure A, the alcohol **S3**, resulting from secondary benzylic C–H hydroxylation was observed in 37% ^{1}H NMR yield using CH_2Br_2 as external standard. Next, the volatiles were removed *in vacuo*, the residue was dry loaded onto a silica gel column and the mixture was purified using 0 – 10% EtOAc/Hexanes. The title compound was isolated as a colorless oil (22 mg, 27% yield). Products resulting from ring opening of the cyclopropane due to an aryl radical cation intermediate were not observed.[40]

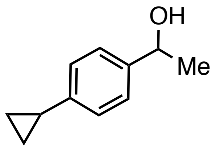

1-(4-cyclopropylphenyl)ethan-1-ol (S3)

^{1}H NMR (500 MHz, $CDCl_3$) (δ, ppm): 7.26 (d, *J* = 8.2 Hz, 2H), 7.06 (d, *J* = 8.1 Hz, 2H), 4.86 (q, *J* = 6.5 Hz, 1H), 1.89 (tt, *J* = 8.4, 5.1 Hz, 1H), 1.81 (s, 1H), 1.48 (d, *J* = 6.5 Hz, 3H), 0.98 – 0.93 (m, 2H), 0.72 – 0.66 (m, 2H).
^{13}C{^{1}H} NMR (126 MHz, $CDCl_3$) (δ, ppm): 143.5, 143.0, 125.9, 125.5, 70.4, 25.1, 15.2, 9.3, 9.3.
HRMS (ESI-TOF) Calcd. for $C_{16}H_{20}O_2$ $[M]^+$: 162.1045, Found: 162.1040.

^{1}H NMR yield spectrum of 1-(4-cyclopropylphenyl)ethan-1-ol (S3)

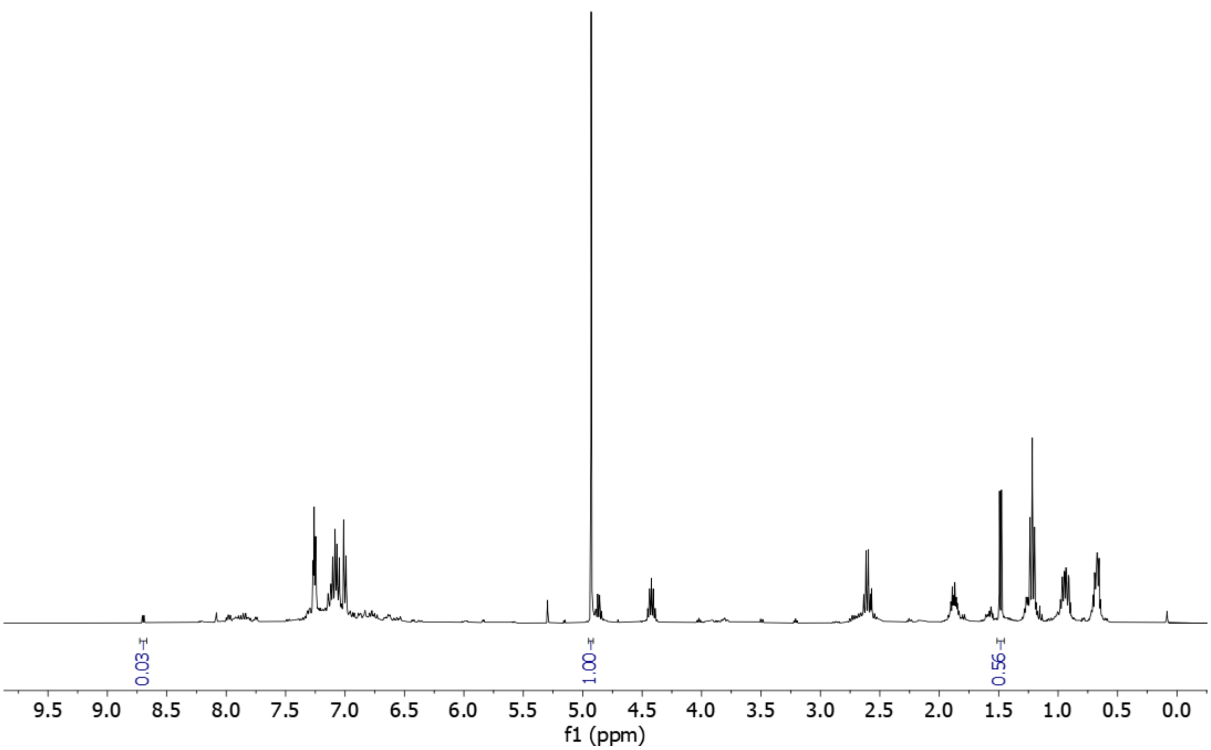

^{1}H NMR (400 MHz, $CDCl_3$) of 1-(4-cyclopropylphenyl)ethan-1-ol (S3)

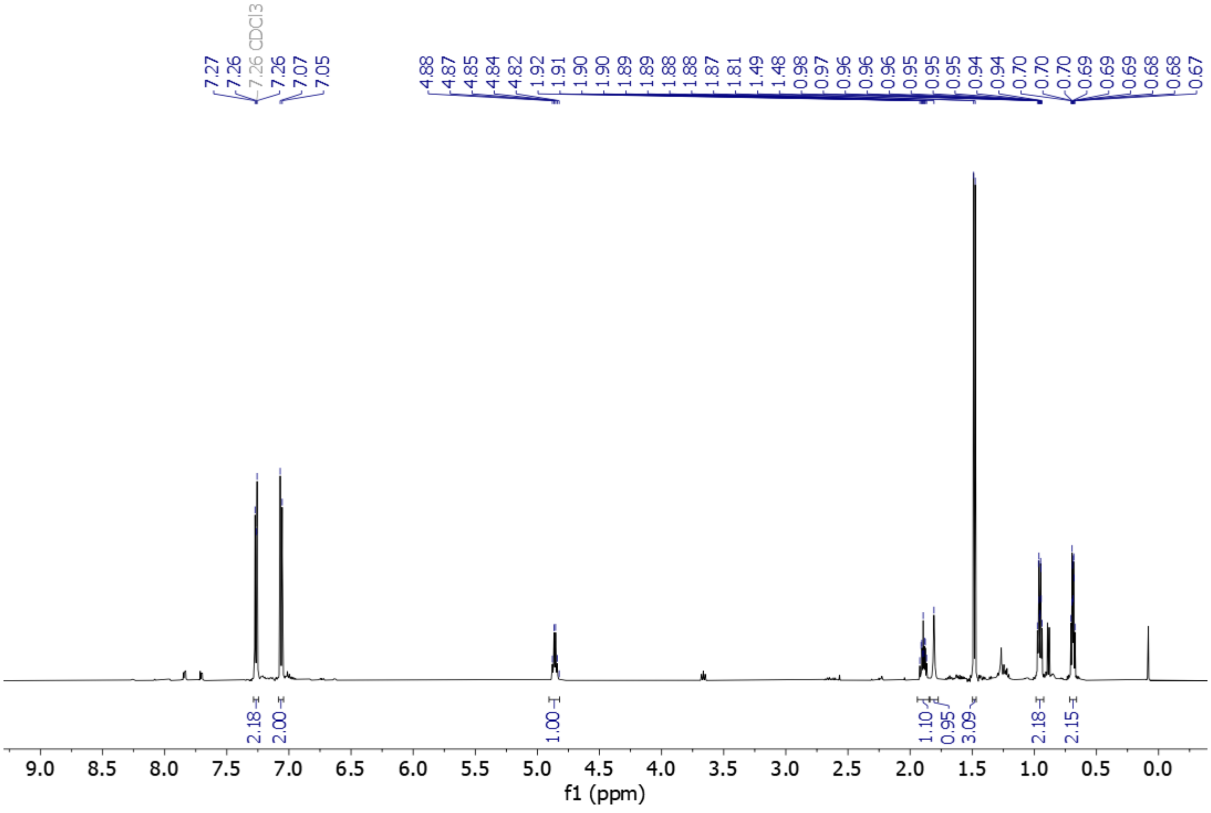

^{13}C NMR (100 MHz, CDCl$_3$) of 1-(4-cyclopropylphenyl)ethan-1-ol (S3)

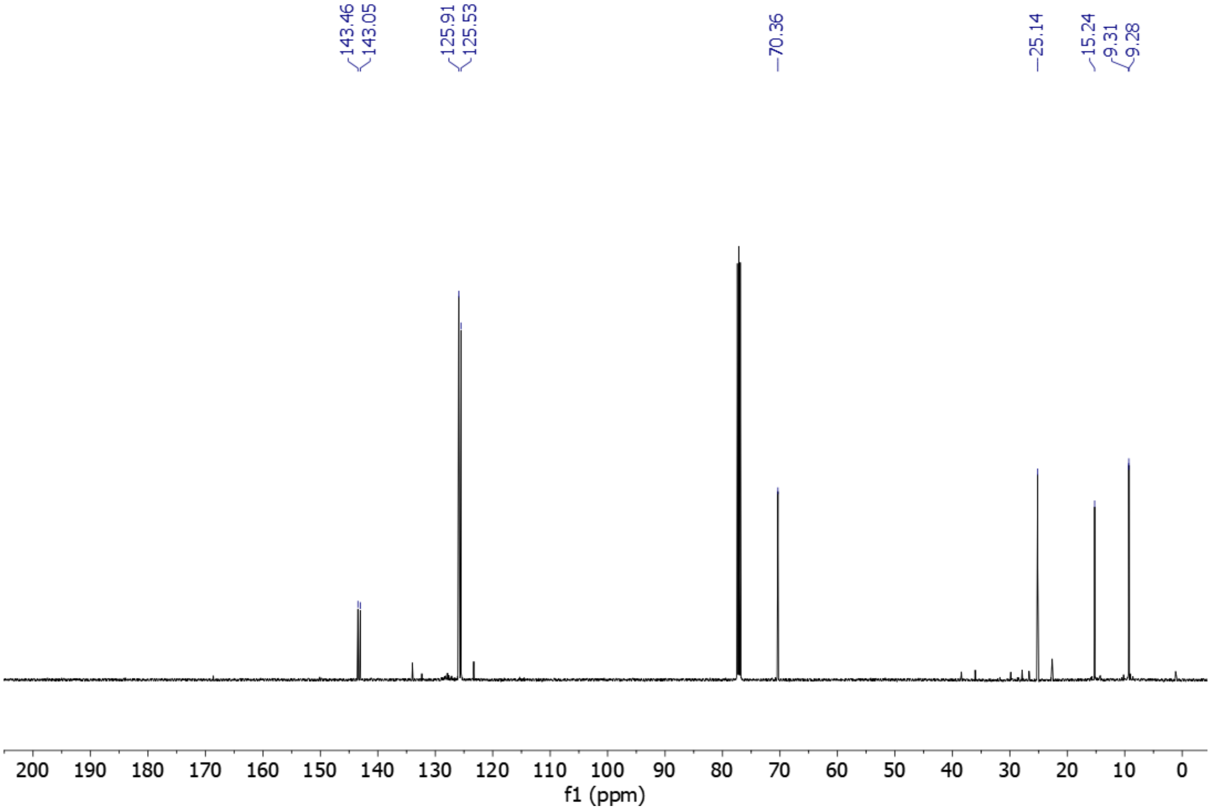

Labeling studies

The C–H hydroxylation of indan **1a** was carried out with nitroarene **3** under general conditions A with 20 equivalents of either $H_2{}^{18}O$, D_2O or HFIP-d_2 additives to investigate if ^{18}O or deuterium was incorporated into the alcohol product. After 18 h, the 390 nm irradiation was stopped and a 100 μL aliquot was taken directly from the reaction vessel, filtered through Celite with 1 mL EtOAc and analyzed by GCMS.

Based on GCMS analysis of the reaction mixtures, the isotope distributions of the 1-indanol product **2a** does not change across these three reactions (Figure S12), indicating that ^{18}O or deuterium were not incorporated. Moreover, the GCMS spectra from these three reactions matched that of commercial **2a**. Although H/D exchange cannot be completely ruled out from these additive studies, under this analysis no appreciable amounts of the ^{18}O or deuterated alcohol were observed by GCMS. Further work is necessary to fully understand the *N*-arylhydroxylamine ether fragmentation step and the origin of the alcohol proton.

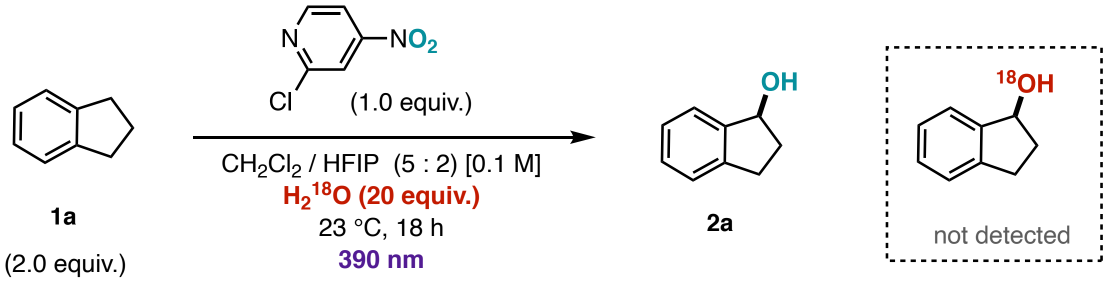

Figure S8: Reaction of **1a** with **3** with $H_2{}^{18}O$ additive

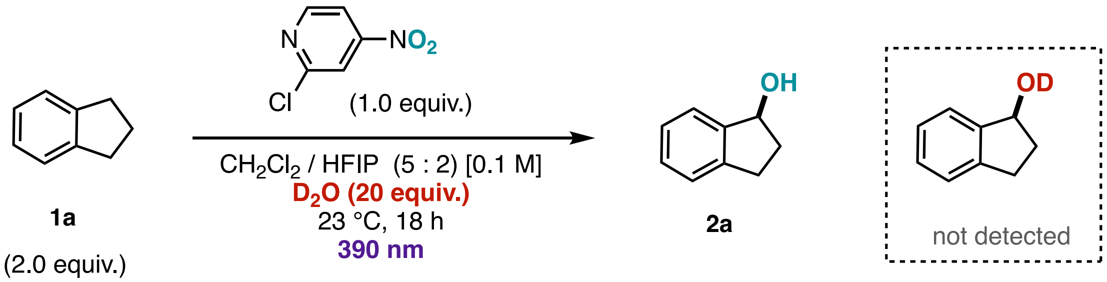

Figure S9: Reaction of **1a** with **3** with D_2O additive

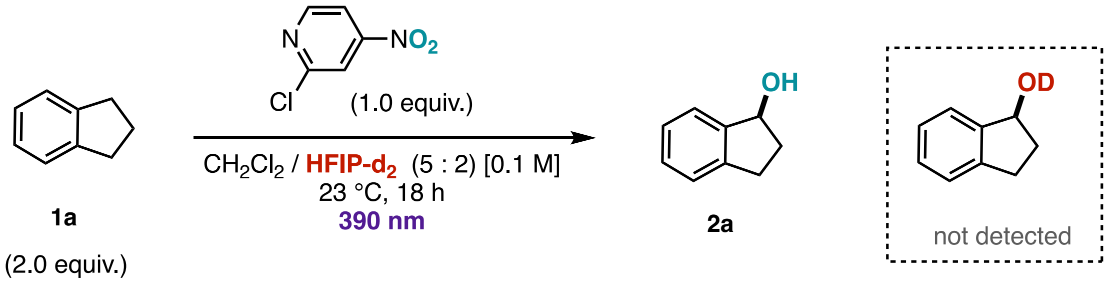

Figure S10: Reaction of **1a** with **3** with HFIP-d_2 additive

GC chromatograms of reactions with $H_2^{18}O$, D_2O and HFIP-d_2

Commercial indanol, **2a**

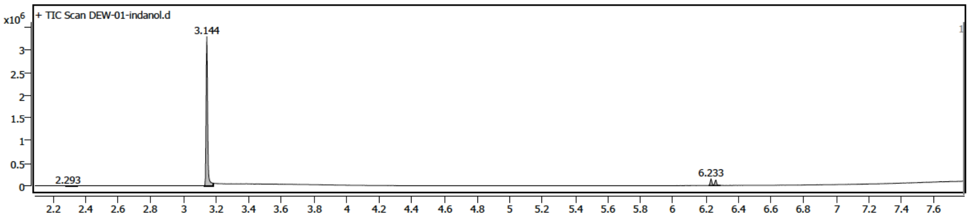

With $H_2^{18}O$ (20 equiv.)

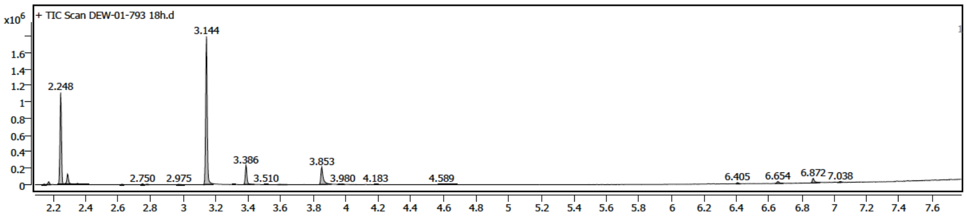

With D_2O (20 equiv.)

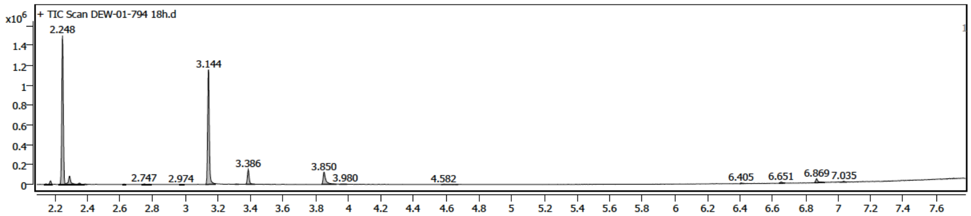

With HFIP-d_2 (20 equiv.)

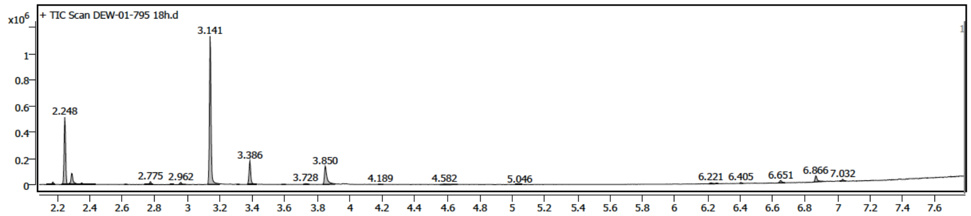

Figure S11: GCMS spectra of labeling study reactions

GCMS spectra of indanol peak, (rt: 3.13 – 3.15 min)

Commercial indanol, **2a**

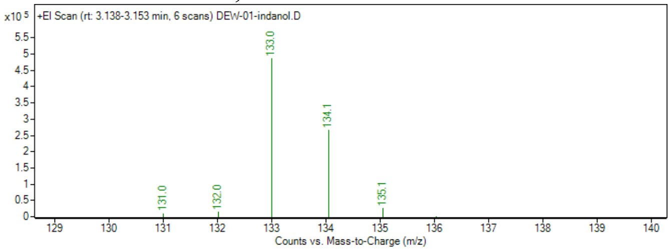

With $H_2{}^{18}O$ (20 equiv.)

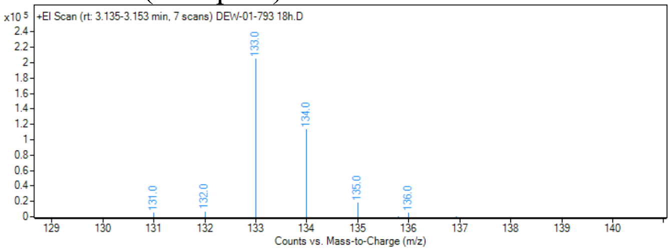

With D_2O (20 equiv.)

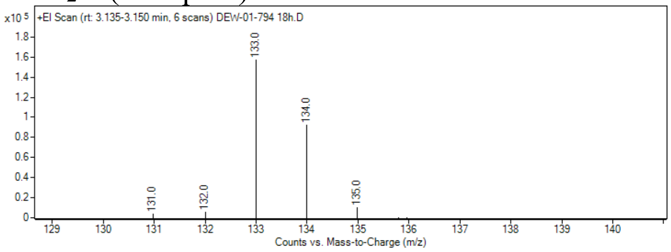

With HFIP-d_2 (20 equiv.)

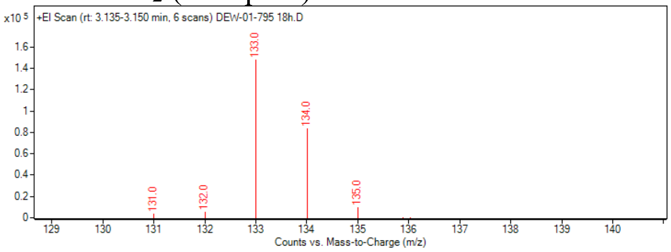

Figure S12: GCMS spectra of labeling studies forming **2a** and commercial **2a** comparison

Detecting reaction byproducts

Following General Procedure B, using unmodified reaction conditions. After 48 h, the irradiation was stopped, and the crude residue was adsorbed onto silica gel and purified by column chromatography (Hexanes). The unreacted alkane starting material **1ag**, nitroarene **4**, azobenzene **13** and azoxyarene **14** eluted in the first two column fractions which were analyzed by ^{1}H NMR and ^{19}F NMR spectroscopy and high-resolution mass spectrometry. NMR spectra of fraction 1 (top) and fraction 2 (bottom) are shown in the figures below. All spectroscopic data for azobenzene **13** matched that previously reported.[41]

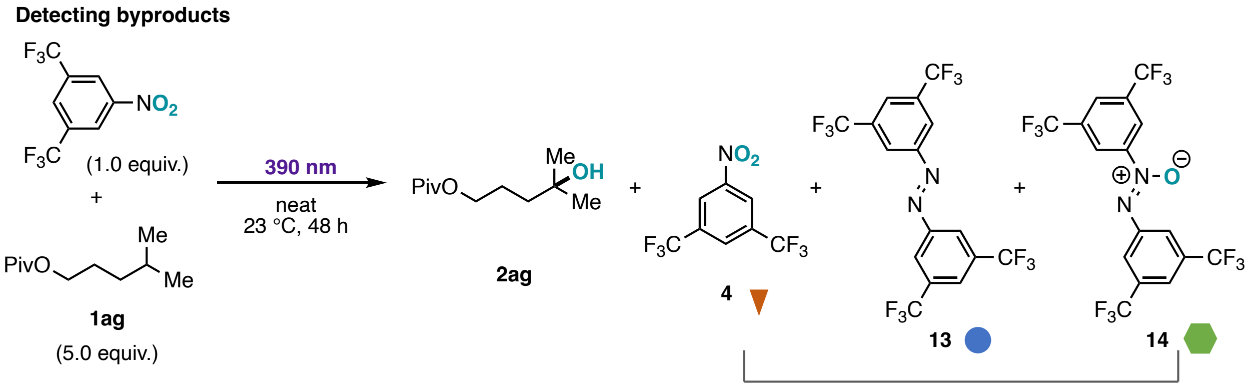

Scheme S1: Isolation of byproducts from the reaction of **1ag** with **4** under standard conditions.

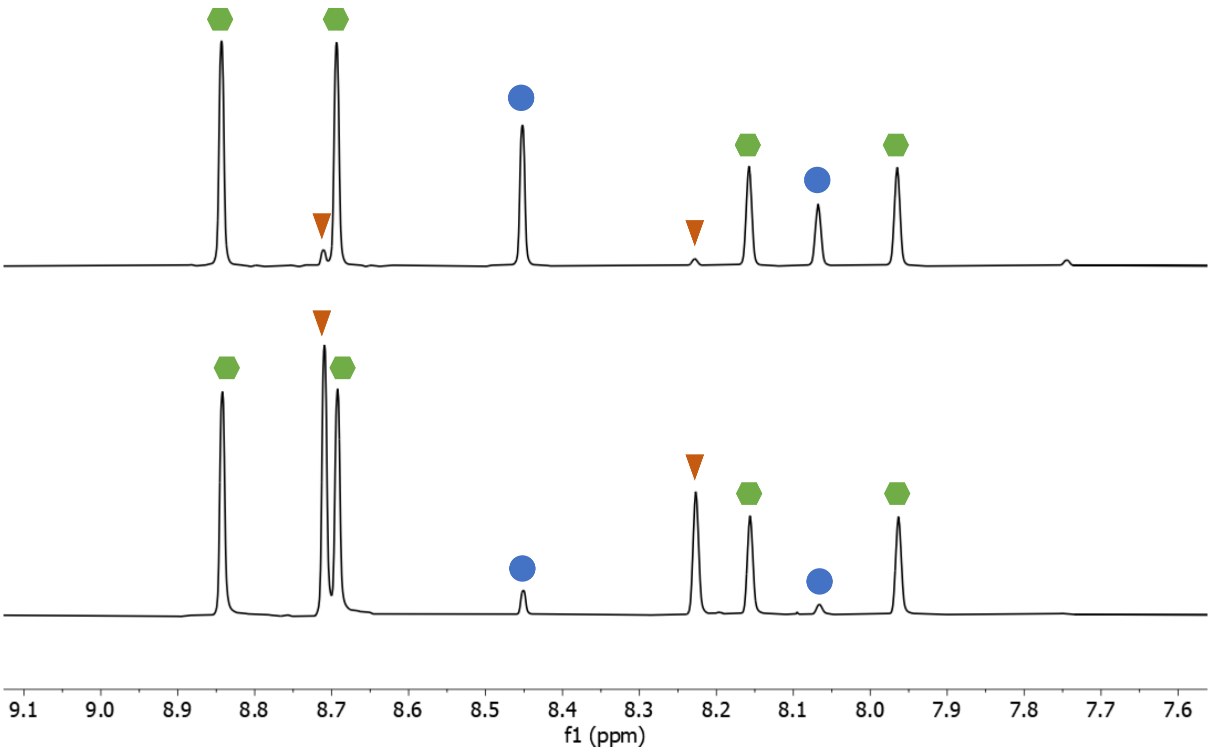

Figure S13: Stacked ^{1}H NMR (500 MHz, $CDCl_3$) spectra of 4, 13 and 14

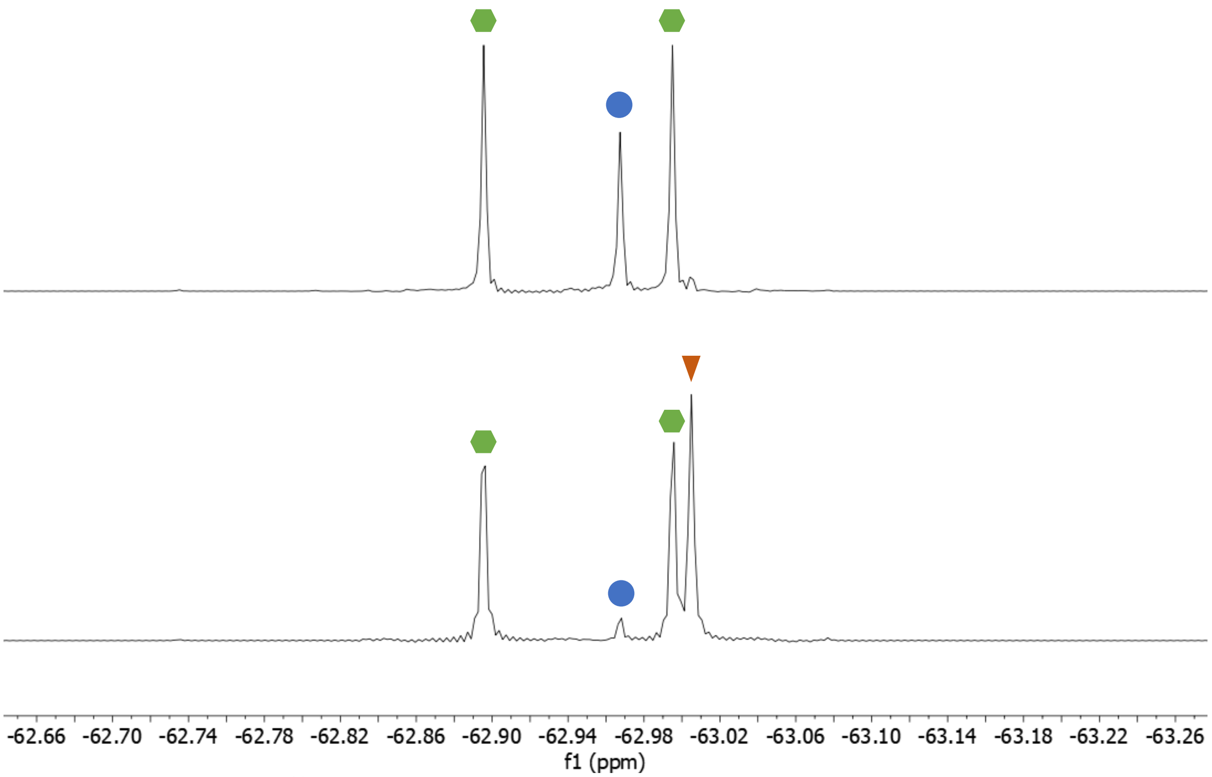

Figure S14: Stacked ^{19}F NMR (471 MHz, $CDCl_3$) spectra of 4, 13 and 14

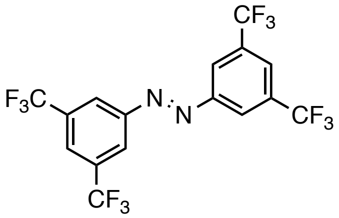

(*E*)-1,2-bis(3,5-bis(trifluoromethyl)phenyl)diazene (10)

^{1}H NMR (500 MHz, $CDCl_3$) (δ, ppm): 8.45 (d, *J* = 1.7 Hz, 4H), 8.07 (tt, *J* = 1.7, 0.8 Hz, 2H).
^{19}F NMR (471 MHz, $CDCl_3$) (δ, ppm): -62.97.
HRMS (ESI-TOF) Calcd. for $C_{16}H_6F_{12}N_2$ $[M]^+$: 454.0339, Found: 454.0334.

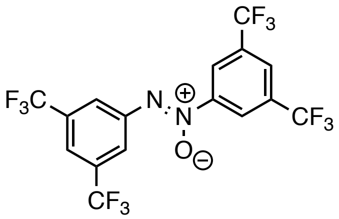

(*Z*)-1,2-bis(3,5-bis(trifluoromethyl)phenyl)diazene 1-oxide (11)

^{1}H NMR (500 MHz, $CDCl_3$) (δ, ppm): 8.84 (d, *J* = 1.6 Hz, 2H), 8.69 (d, *J* = 1.7 Hz, 2H), 8.16 (t, *J* = 0.8 Hz, 1H), 7.96 (t, *J* = 0.8 Hz, 1H).
^{19}F NMR (471 MHz, $CDCl_3$) (δ, ppm): -62.90, -63.00.
HRMS (ESI-TOF) Calcd. for $C_{16}H_6F_{12}N_2O$ $[M]^+$: 470.0289, Found: 470.0290.

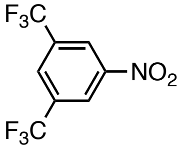

1-nitro-3,5-bis(trifluoromethyl)benzene (4)

^{1}H NMR (500 MHz, $CDCl_3$) (δ, ppm): 8.73 – 8.69 (m, 2H), 8.25 – 8.20 (m, 1H).
^{19}F NMR (471 MHz, $CDCl_3$) (δ, ppm): -63.01.

PhotoNMR studies

Visualizing Recombination Intermediates

^{1}H PhotoNMR spectra were collected over 16 hours in hope of visualizing the radical recombination intermediate in both activated (Figure S16) and unactivated (Figure S15) substrate reactions. We ran the experiments at 23 ºC continuously overnight. After 16 hours the reactions were stopped and analyzed further. Due to lack of stirring both experiments suffered from low conversion. We hypothesize that the mirroring downshifted peaks to the product (Figure S15 & S16) represents the radical recombination *N*-arylhydroxylamine ether. After the PhotoNMR experiment, the crude mixtures were run on HRMS (ESI-TOF) which indicated a mass of these potential intermediates **9** & **15** (Scheme S2 & Scheme S3).

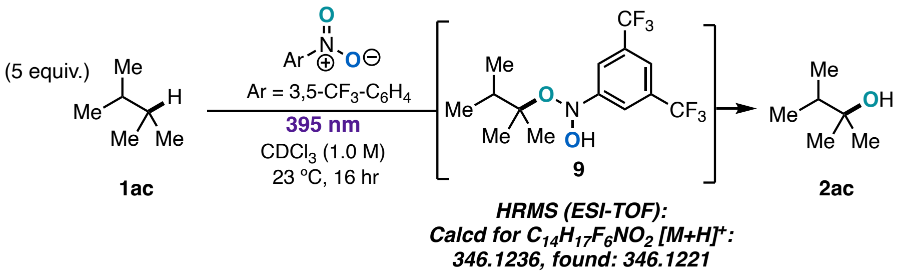

Scheme S2:^{1}H PhotoNMR experiment of **1ac** and **4** featuring key radical recombination intermediate peaks.

Even though the reaction overall had poor conversion we were able to detect the product as well as two intermediates. As seen in Figure S15 there is a distinct peak at around 5.0 ppm (teal dot)

that grows, decays, and shifts over the course of the reaction. We hypothesize that this could either be the first HAT intermediate before radical recombination or the OH from **9**. The next notable peaks occur between 1.6 ppm - 2.1 ppm (red dot), which correspond to the single proton between the two methyl groups. We hypothesize that **9** would have identical but downshifted peaks to the product due to the aromatic nitrogen aiding in pulling electron density away from the proton. We see this trend for the two other proton signals (green & purple). This is in alignment with the HRMS data suggesting we do form an *N*-arylhydroxylamine ether upon recombination (Scheme S2).

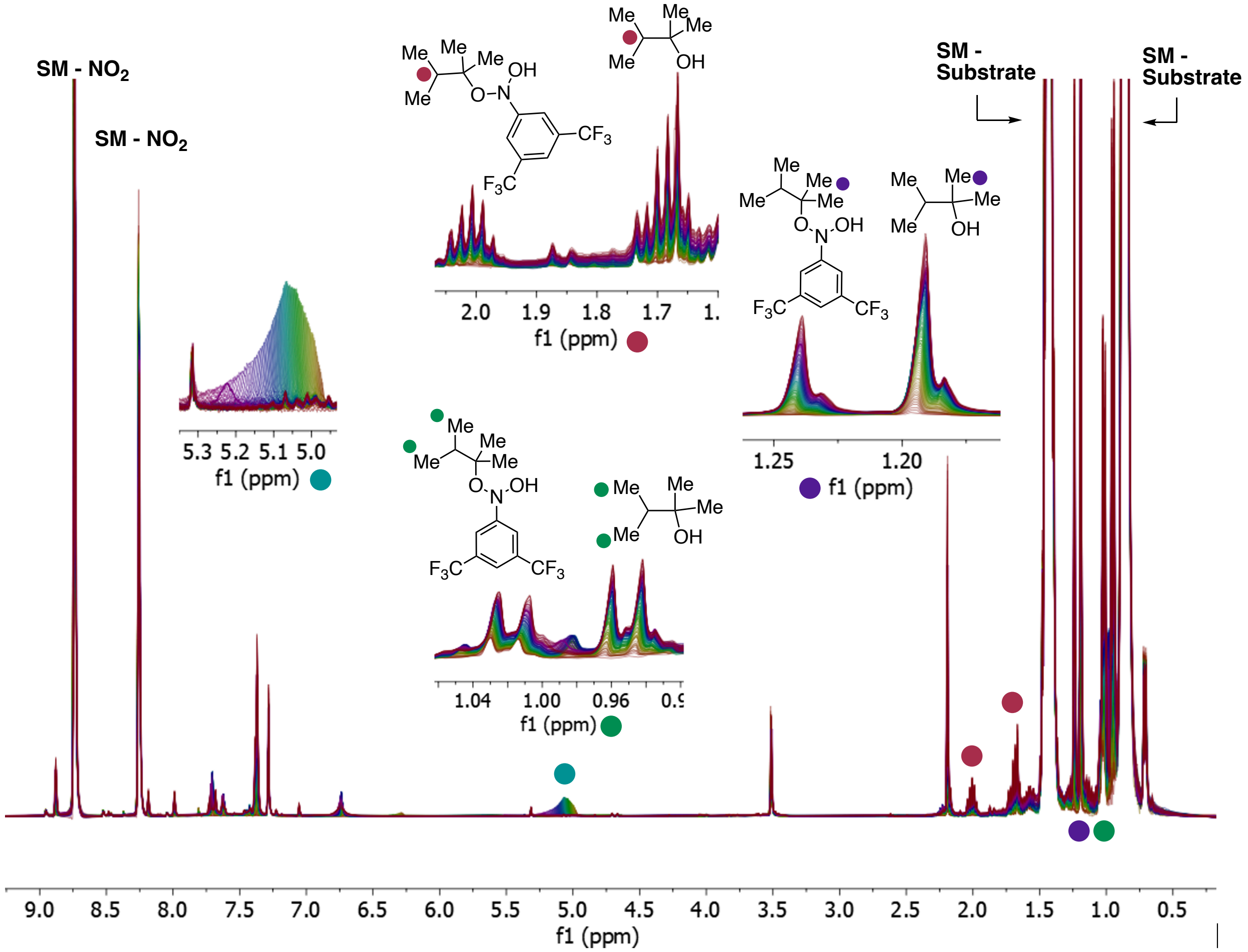

Figure S15: Superimposed 400 MHz ^{1}H NMR spectra ($CDCl_3$) of the reaction of **1ac** with **4** at 23 °C.

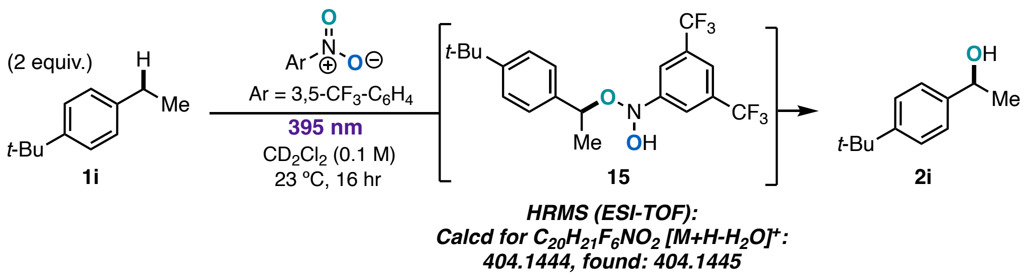

Scheme S3: ^{1}H PhotoNMR experiment of the reaction between **1i** and **4** featuring key radical recombination intermediate peaks

We decided to test **1i** to see if our activated (benzylic) substrates go through a similar pathway. As seen in Figure S16 we do get the same intermediates trends as the previous experiment suggesting both substrates undergo radical recombination. Similarly, to **1ac** there is a distinct peak at around 5.15 ppm (teal dot) that grows, shifts, and begins to decay over the course of the reaction monitoring. The next notable peaks are the identical quartet peaks (red dot), which correspond to the single benzylic proton adjacent to the oxygen. The green and purple dots also match the trend of identical but slightly shifted peaks suggesting the *N*-arylhydroxylamine ether upon recombination appears in benzylic systems as well.

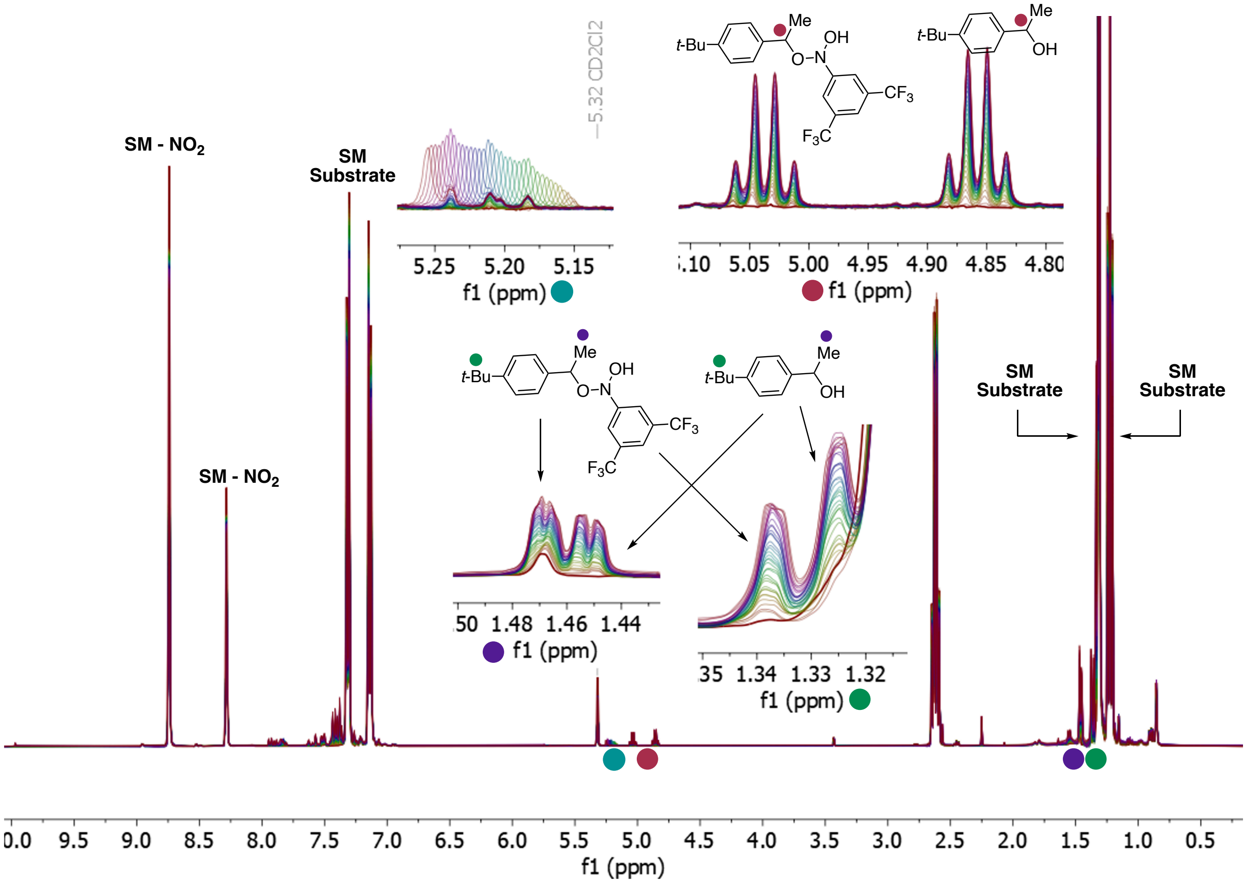

Figure S16: Superimposed 400 MHz ^{1}H NMR spectra (CD_2Cl_2) reaction of **1i** with **4** at 23 °C.

Ground State vs Excited State Control Experiment

We wanted to determine if a ground-state nitro group could also participate in the radical recombination step with the formed alkyl radical intermediate. To provide support for this mechanism, we ran *in situ* PhotoNMR with nitromethane, **4** and **1a.** Nitromethane acted as our ground-state surrogate since it cannot be photoexcited at 390 nm. Hence, if the formed alkyl radical reacts with the ground-states of the nitro-systems in solution, one would expect to detect the radical recombination adducts of the alkyl radical with the nitroarene and the nitromethane. We found that over the 16 hr of photoirradiation, no nitromethane was consumed nor detection of the radical recombination intermediate of nitromethane and the alky radical, only **4** was consumed to produce **2a** was observed (Figure S17 & S18). These results tell us that it is unlikely that the alkyl radical

interacts with the ground-state nitroarene leading to product formation under our reaction conditions.

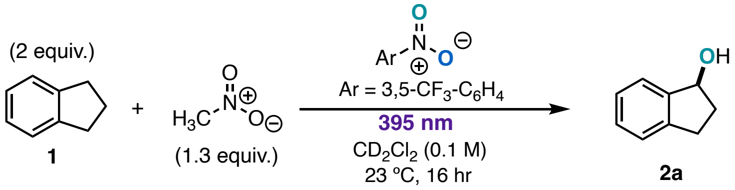

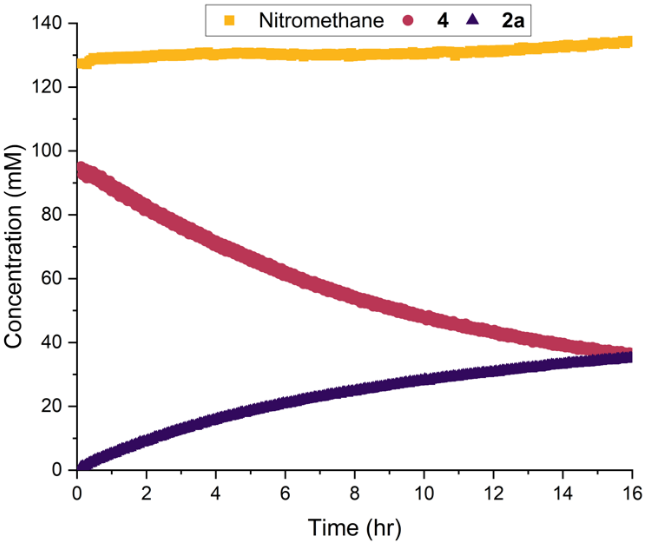

Figure S17: Concentration versus time of PhotoNMR experiment tracking nitromethane, **4** consumption and growth of **2a.**

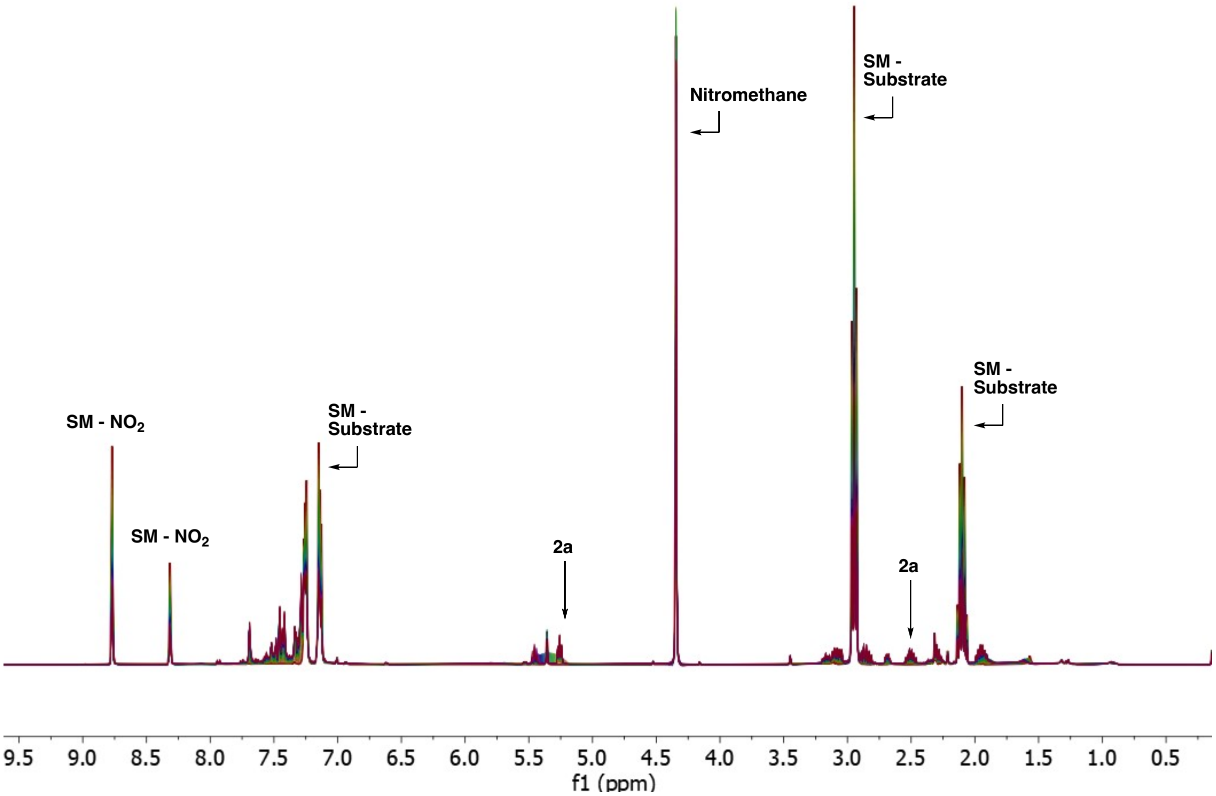

Figure S18: Superimposed 400 MHz ^{1}H NMR spectra (CD_2Cl_2) reaction of **1a** with **4** and nitromethane at 23 ºC over 16hr.

Light On/Off Studies

2-chloro-4-nitropyridine (15.9 mg, 0.1 mmol, 1 equiv.), indan (0.2 mmol, 2 equiv.), & CD_2Cl_2 (0.4 mL, 250 mM) were added under nitrogen to an NMR tube. Under a PhotoNMR setup, a fiber optic cable connected to a 395 nm lamp was added to the reaction, and ^{1}HNMR experiments were taken every 90 seconds. Product growth was monitored by the integration of the peak of the benzylic proton alpha to the formed alcohol. The product concentration was obtained by monitoring the disappearance of starting material at an initial known concentration. The lack of product formation in the dark during these studies is evidence against a radical chain mechanism.

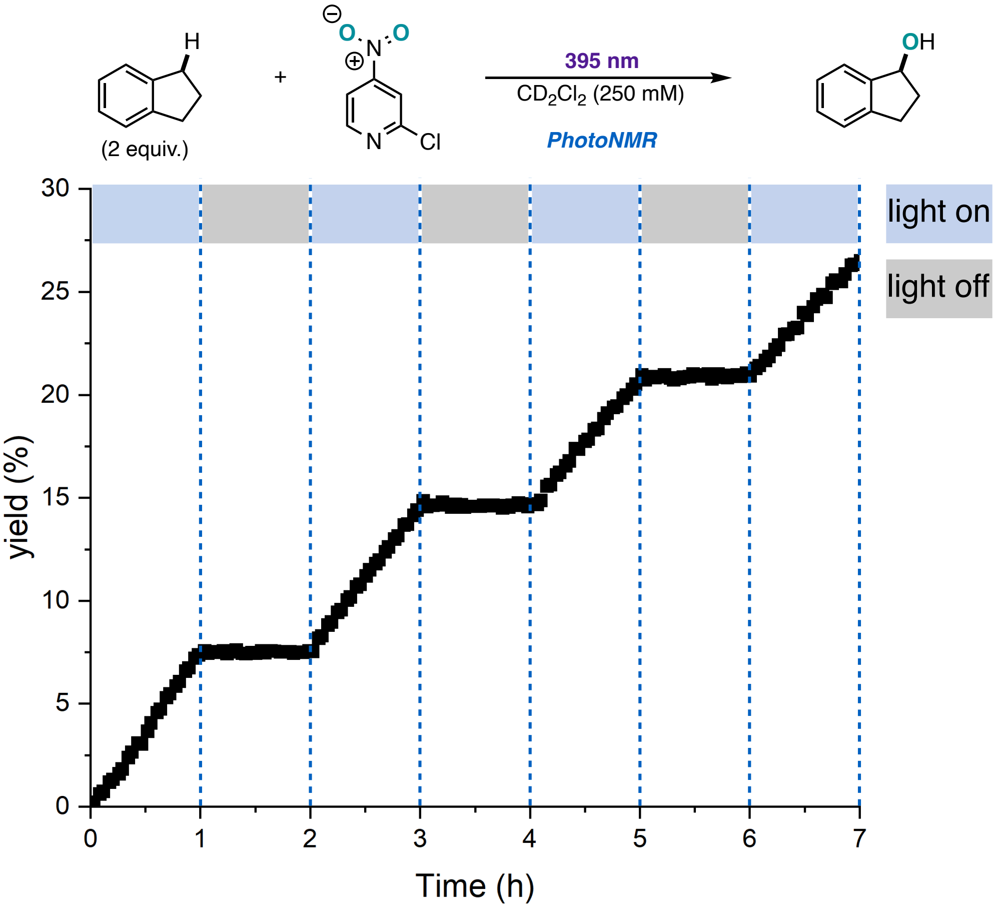

Figure S19: Light on/off study where production of indanol was monitored every 90 seconds. No product formation was observed when the reaction was dark.

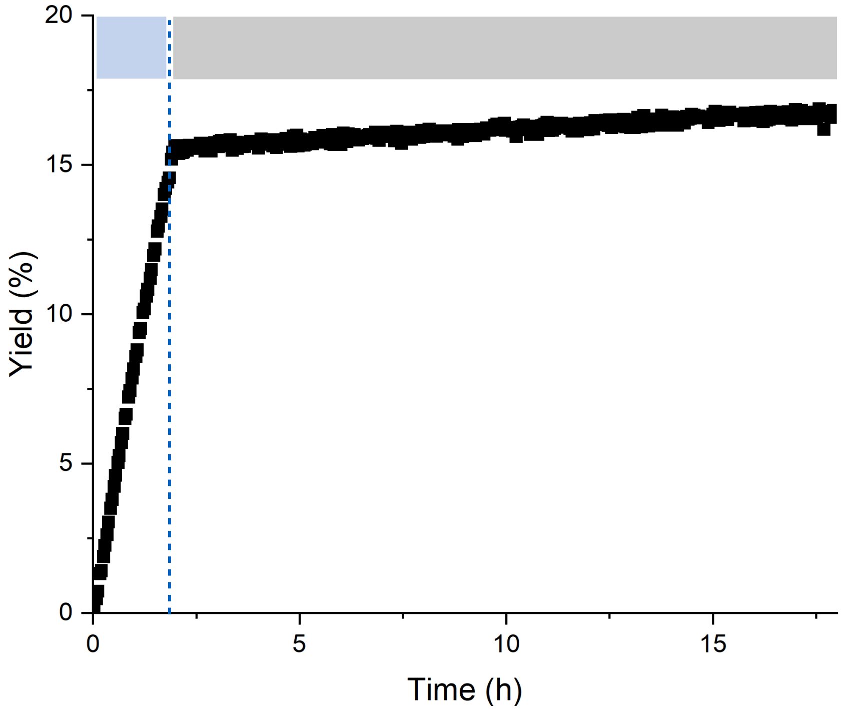

Figure S20: Radical chain mechanism probe where the reaction was irradiated for two hours before the fiber optic cable was turned off. The reaction was allowed to sit in the dark in the NMR magnet for 16 additional hours with ^{1}HNMR experiments continuing every 90 seconds. The yield increased <1% over the course of the 16 hours (within error of the instrument due to peak broadening over time).

Scale up procedure

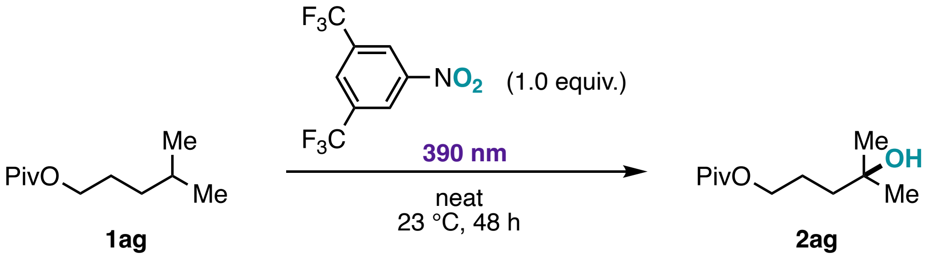

1ag (5.0 equiv.) **2ag** 0.436 g, 62% yield

Following General Procedure B, using unmodified reaction conditions. An oven-dried 50 mL round bottom flask with Teflon stir bar was charged with 3,5-bis(trifluoromethyl)nitrobenzene (972 mg, 3.750 mmol, 1.00 equiv.), and **1ag** (3.493 g, 18.750 mmol, 5.00 equiv.). The reaction flask was placed 3 cm in between two 390 nm Kessil lamps with a cooling fan. The reaction was stirred under irradiation for 48 h. Then, the irradiation was stopped, and the crude residue was adsorbed onto silica gel and purified by column chromatography using 25% EtOAc/Hexanes to give **2ag** (0.436 g, 62%). All spectroscopic data matched that previously reported.[33]

UV-Vis Spectrum of Nitroarenes

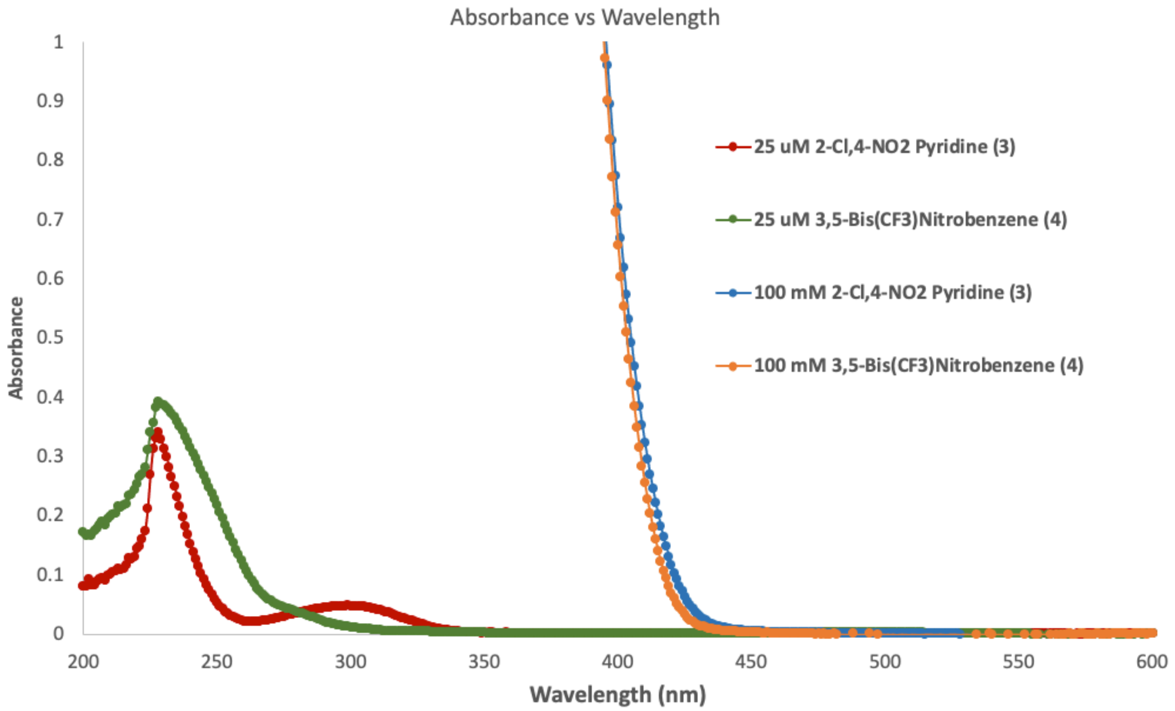

Figure S20: UV-Vis absorption spectrum of **3** & **4** at 25 μM and 100 mM (reaction concentration).

NMR spectra

^{1}H NMR (500 MHz, $CDCl_3$) of 2,3-dihydro-1H-inden-5-yl trifluoromethanesulfonate (1e)

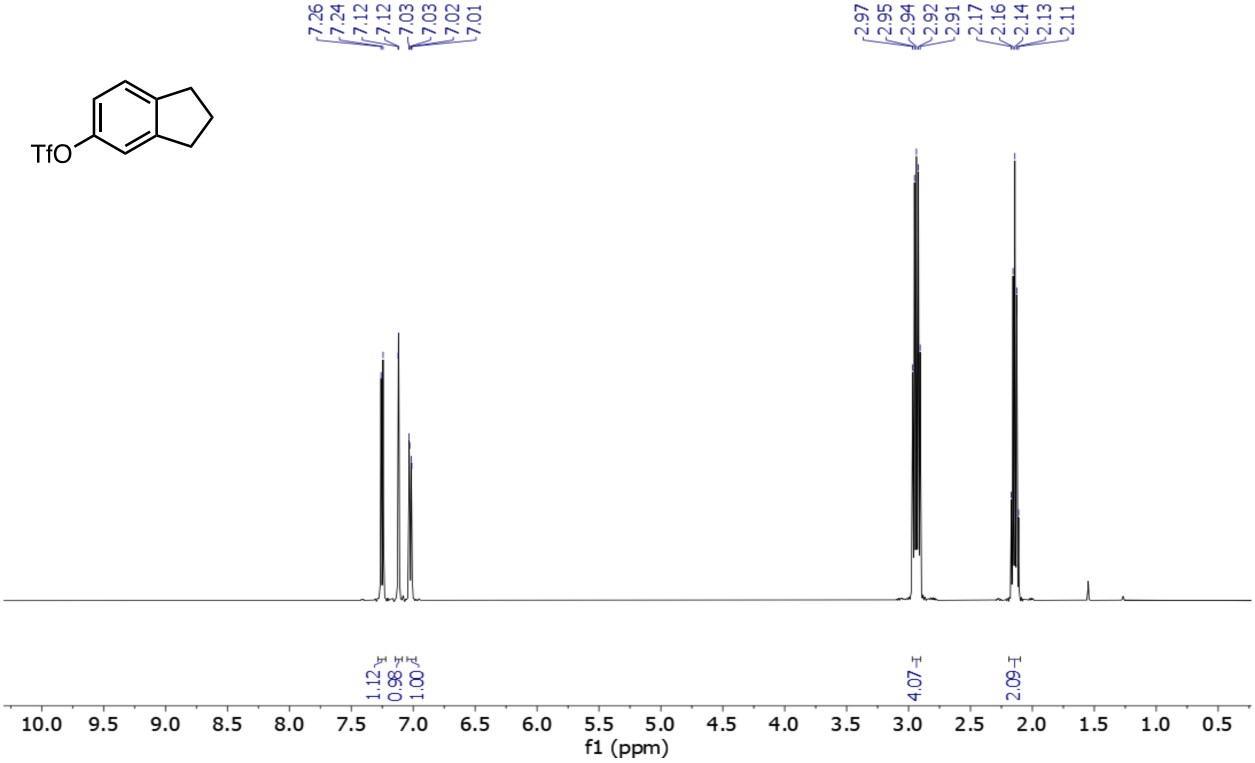

^{13}C NMR (126 MHz, $CDCl_3$) of 2,3-dihydro-1H-inden-5-yl trifluoromethanesulfonate (1e)

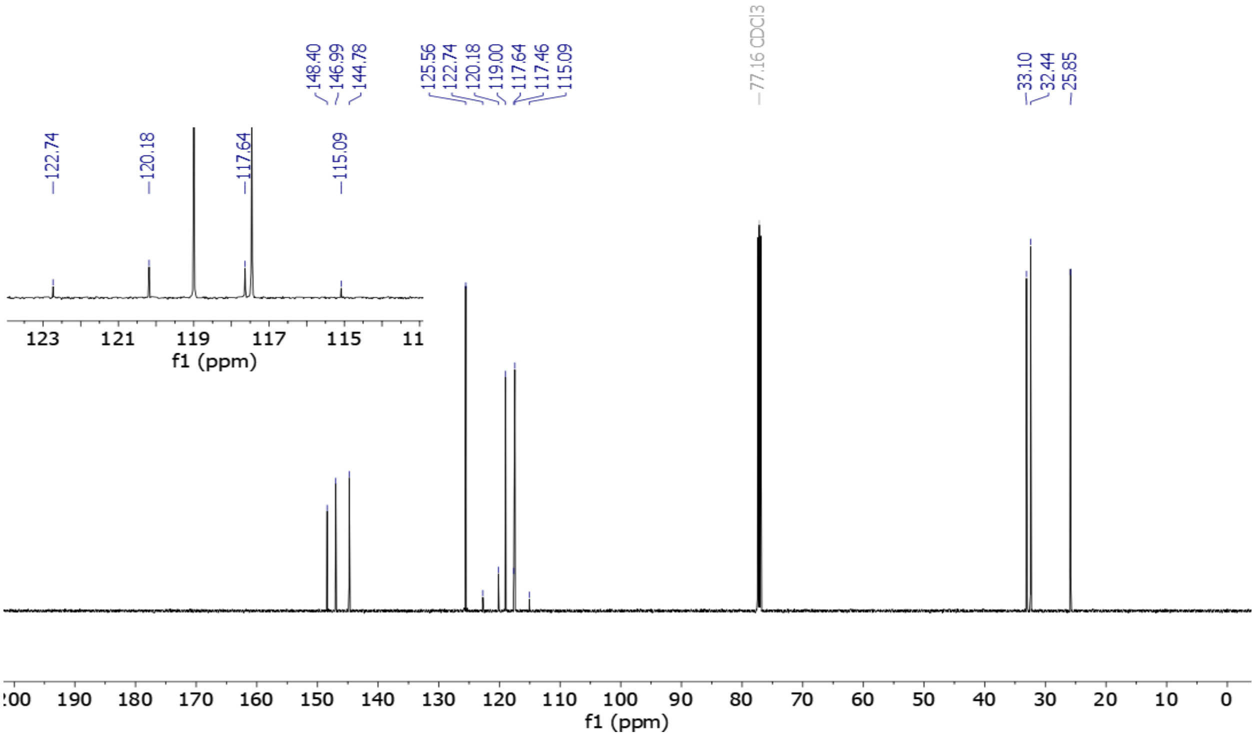

^{19}F NMR (471 MHz, $CDCl_3$) of 2,3-dihydro-1H-inden-5-yl trifluoromethanesulfonate (1e)

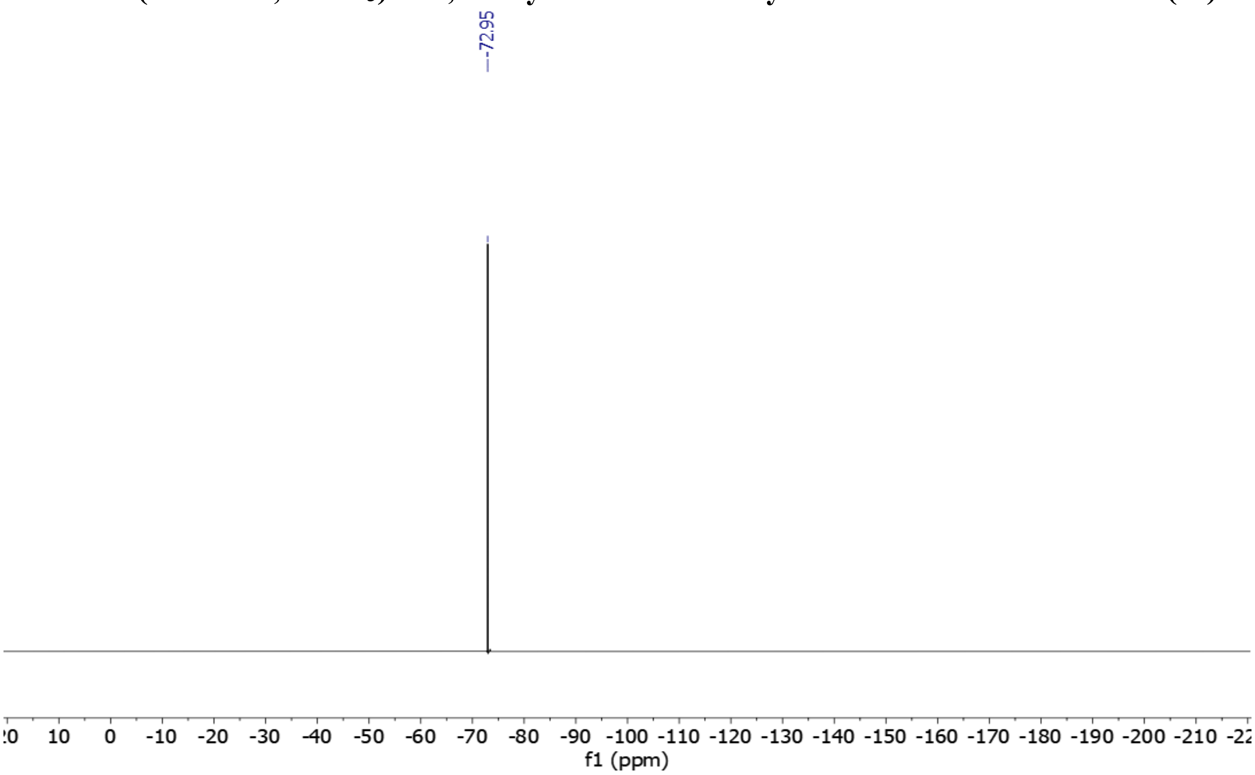

^{1}H NMR (400 MHz, CDCl₃) of 5-methylhexyl pivalate (1ah)

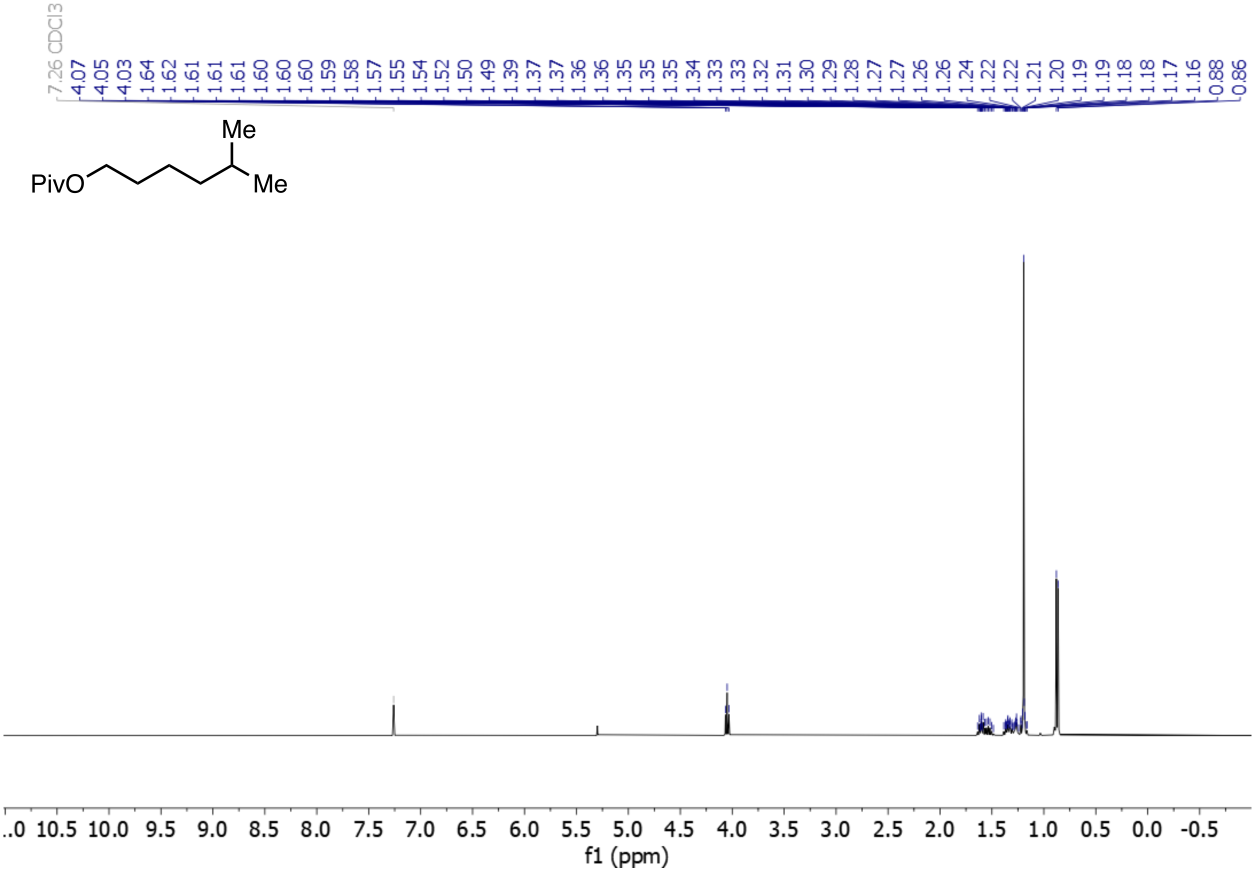

^{1}H NMR (500 MHz, $CDCl_3$) of ((5-methylhexyl)sulfonyl)benzene (1al)

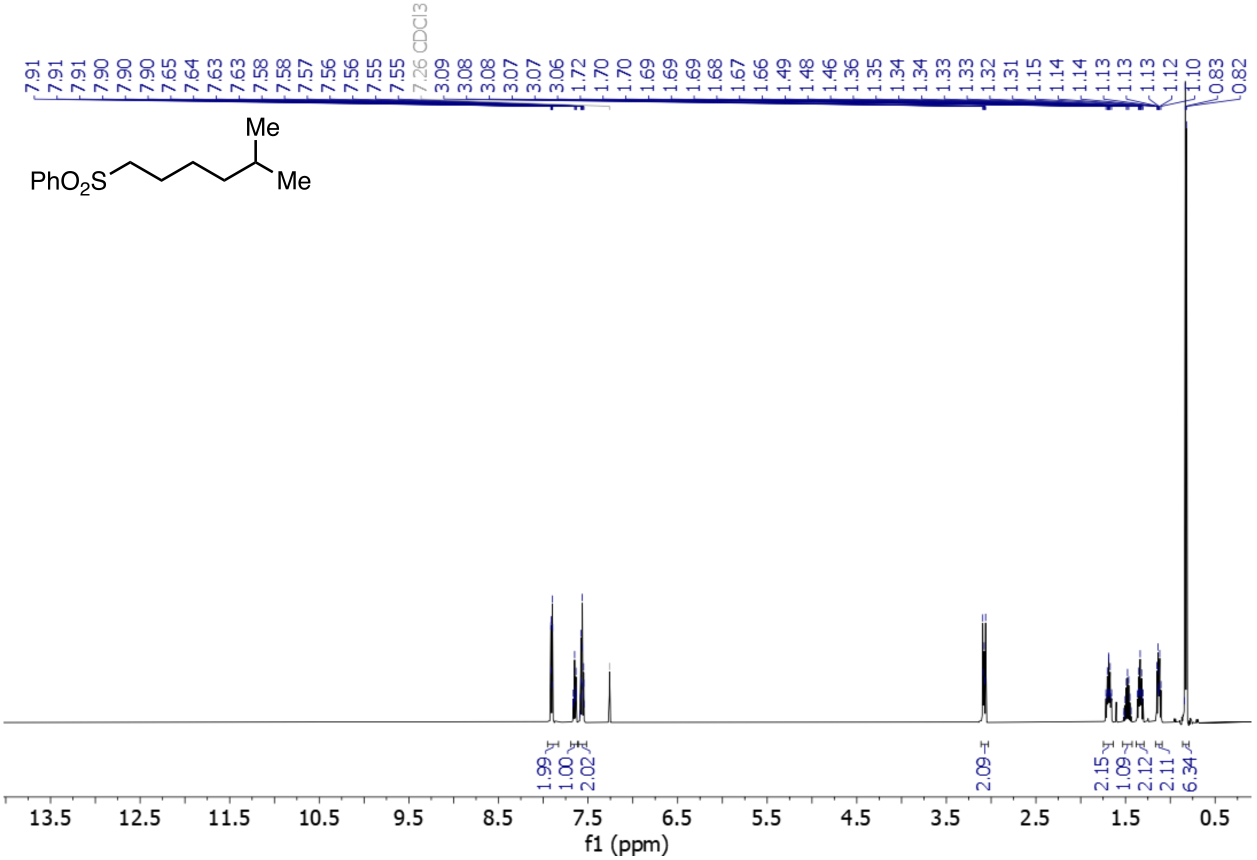

^{13}C NMR (126 MHz, $CDCl_3$) of ((5-methylhexyl)sulfonyl)benzene (1al)

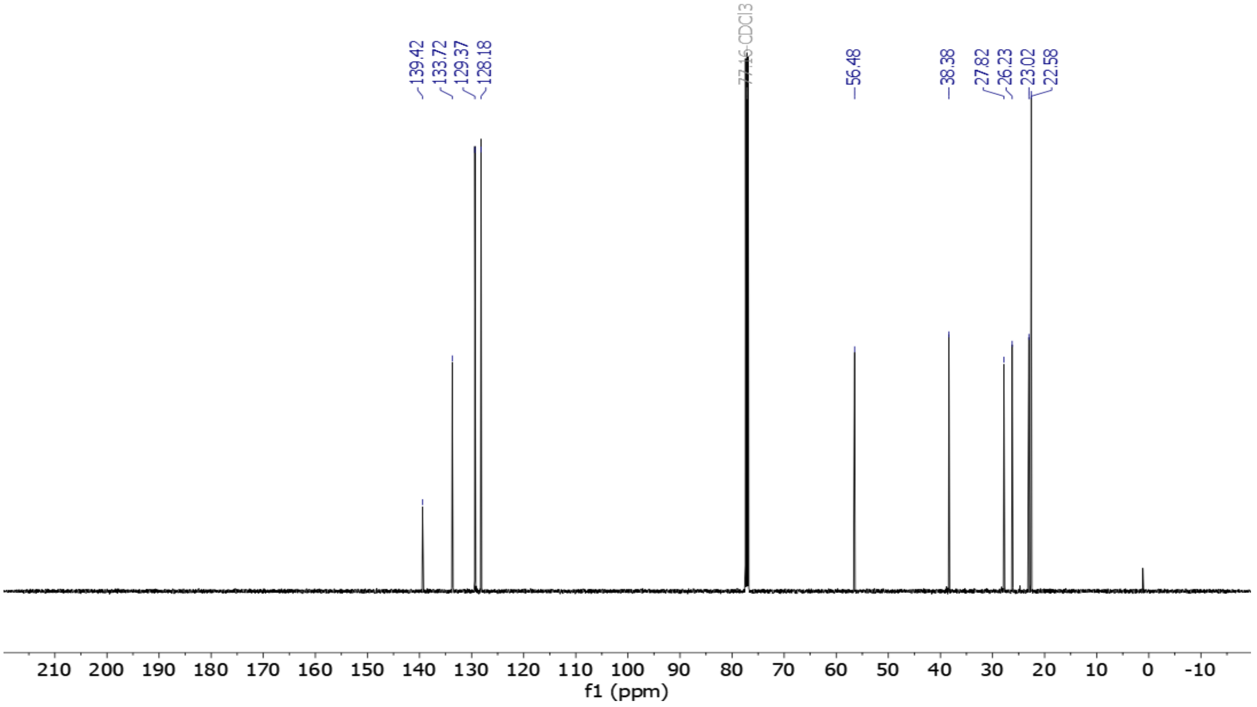

^{1}H NMR (500 MHz, $CDCl_3$) of 1-indanol (2a)

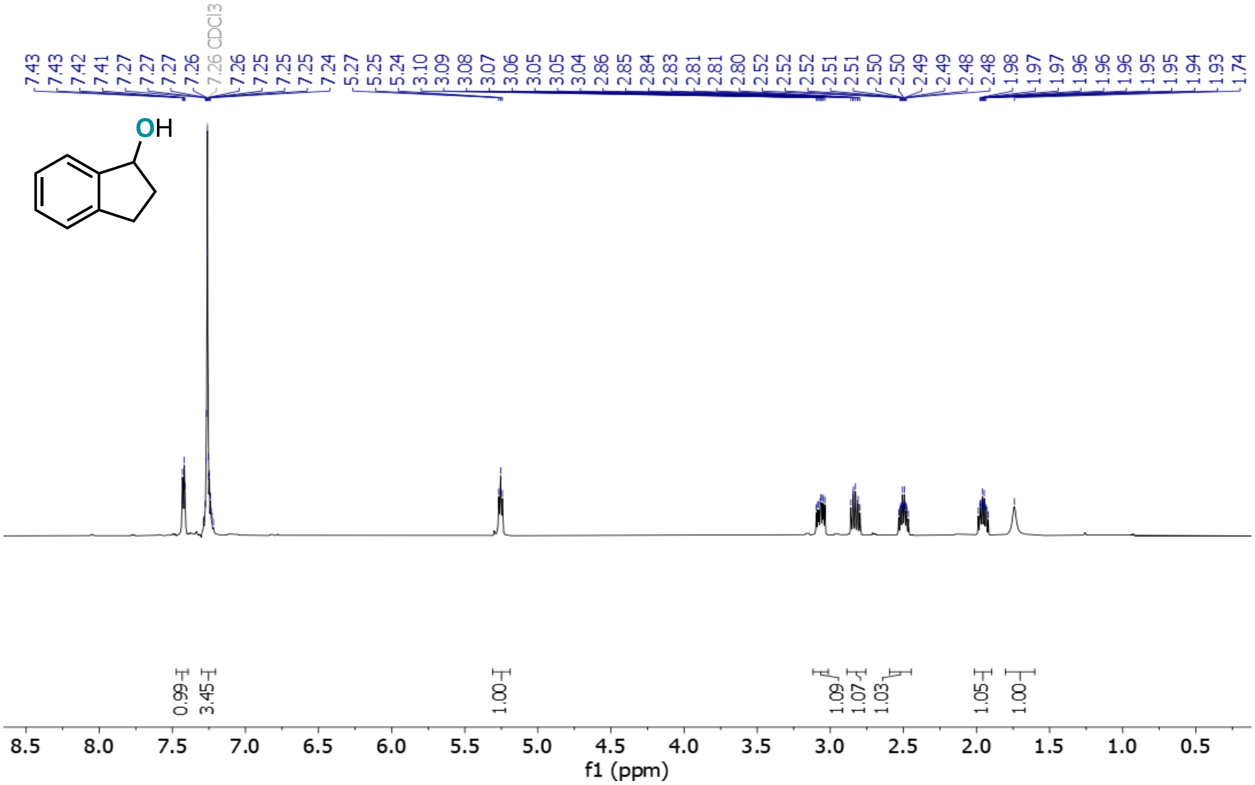

^{13}C NMR (126 MHz, $CDCl_3$) of 1-indanol (2a)

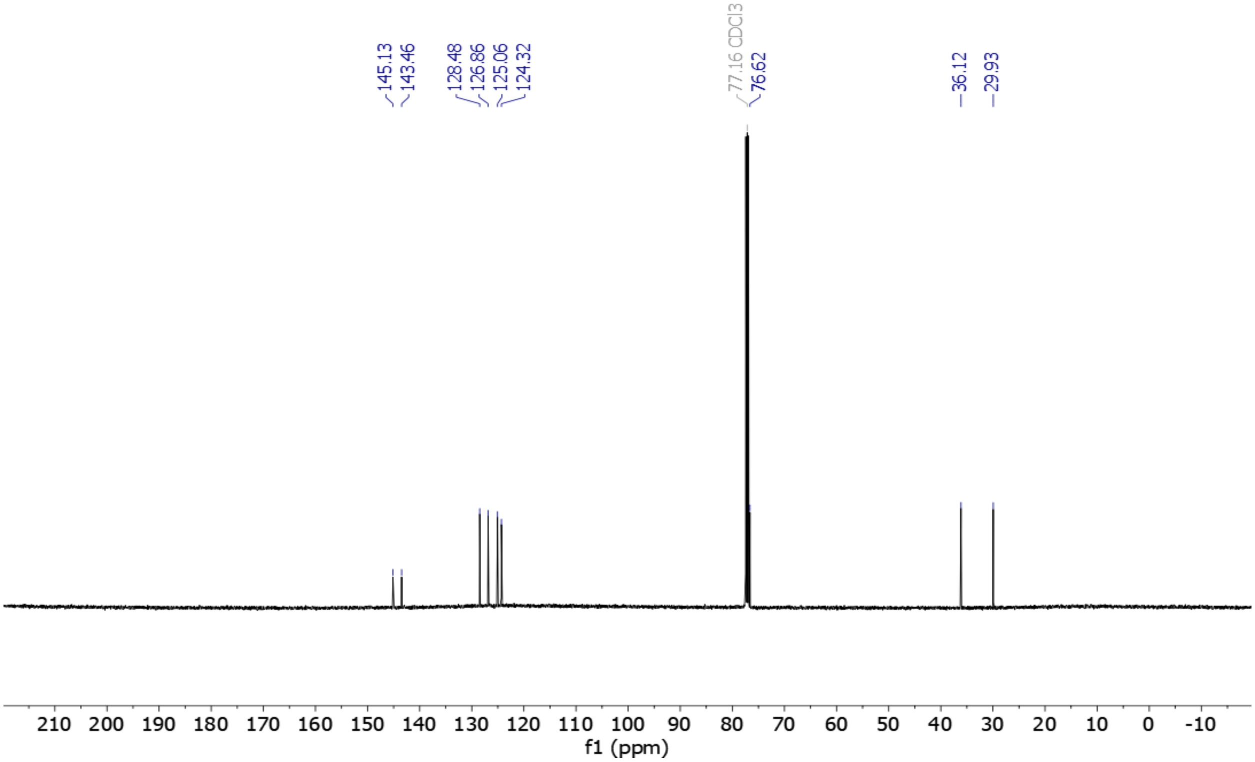

^{1}H NMR (500 MHz, CDCl₃) of 1,2,3,4-tetrahydronaphthalen-1-ol (2b)

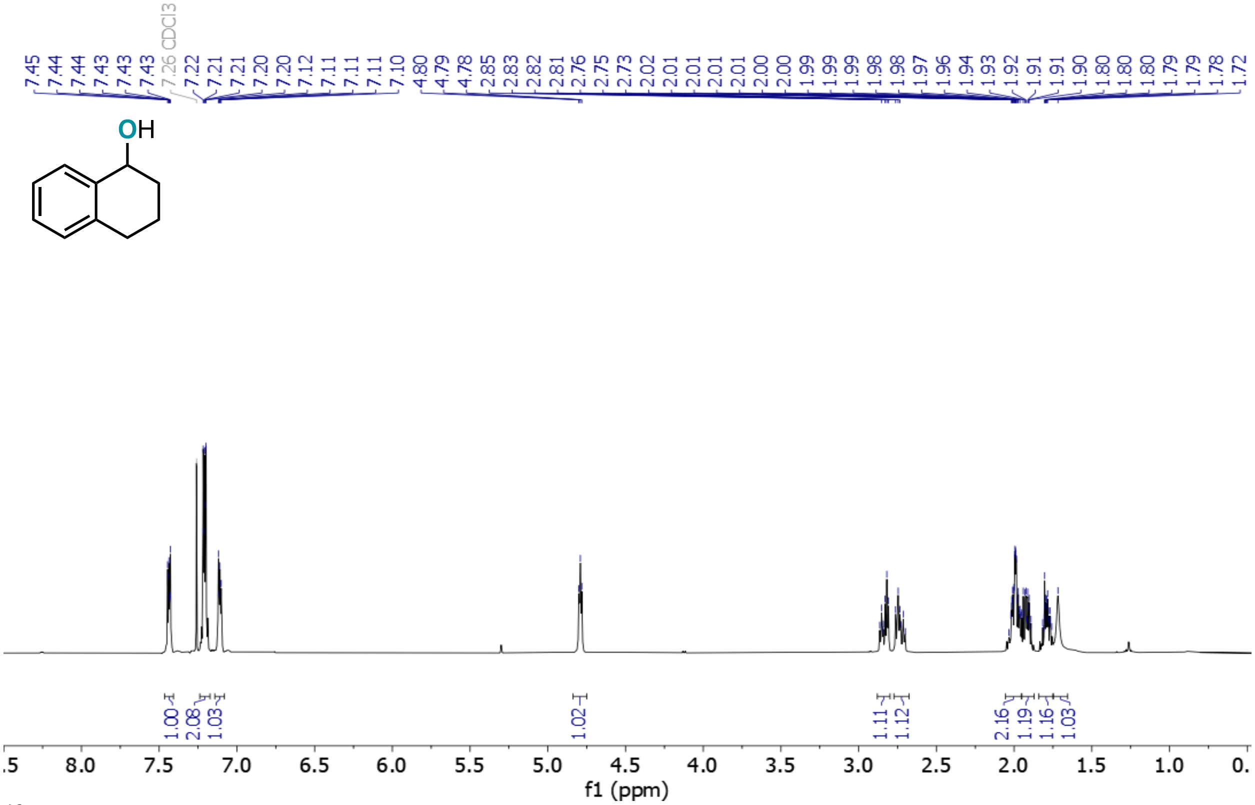

^{13}C NMR (126 MHz, CDCl₃) of 1,2,3,4-tetrahydronaphthalen-1-ol (2b)

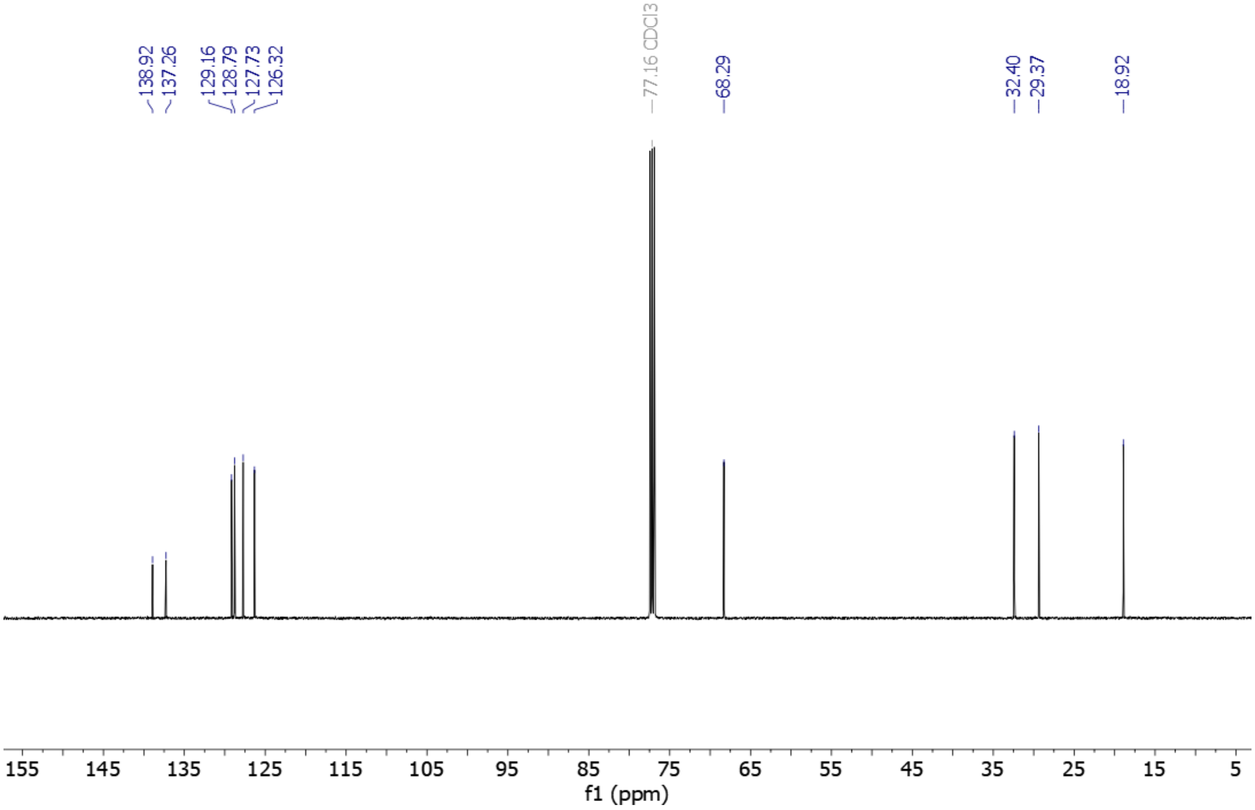

^{1}H NMR (500 MHz, $CDCl_3$) of 6,7,8,9-tetrahydro-5H-benzo[7]annulen-5-ol (2c)

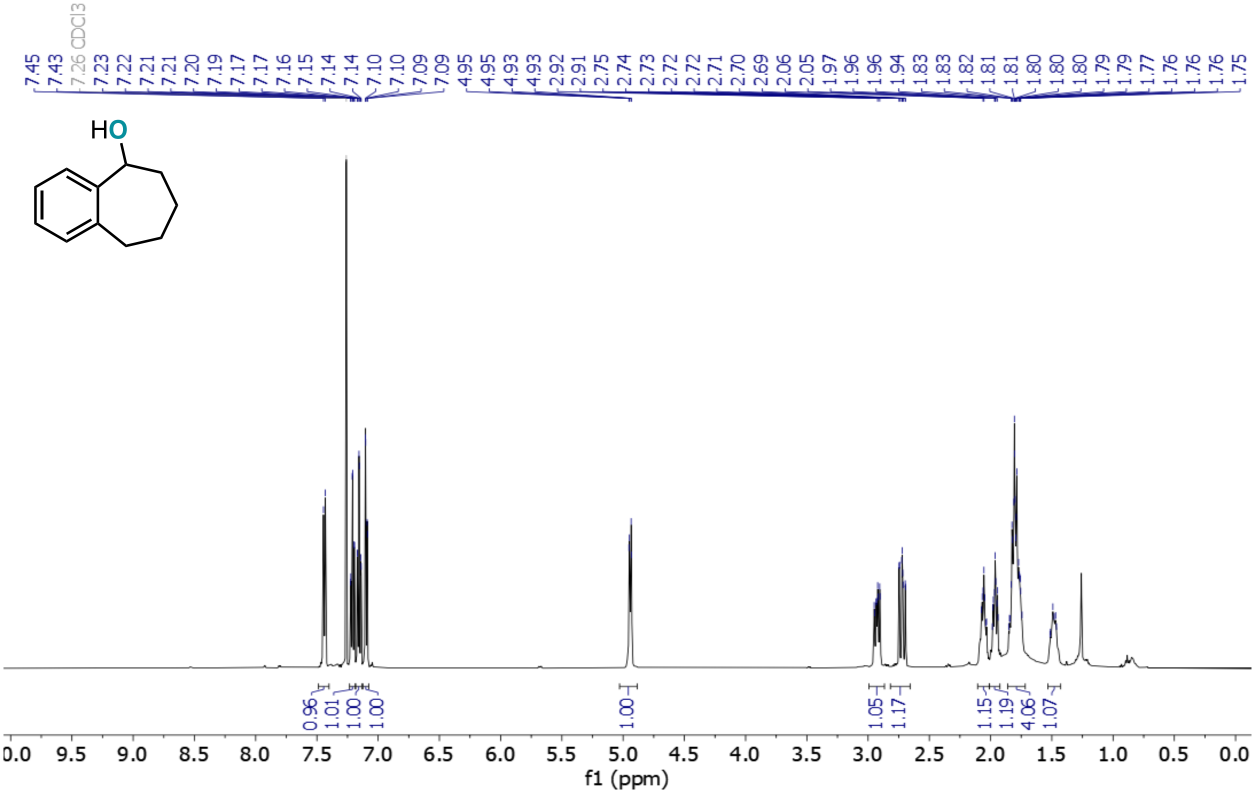

^{13}C NMR (101 MHz, $CDCl_3$) of 6,7,8,9-tetrahydro-5H-benzo[7]annulen-5-ol (2c)

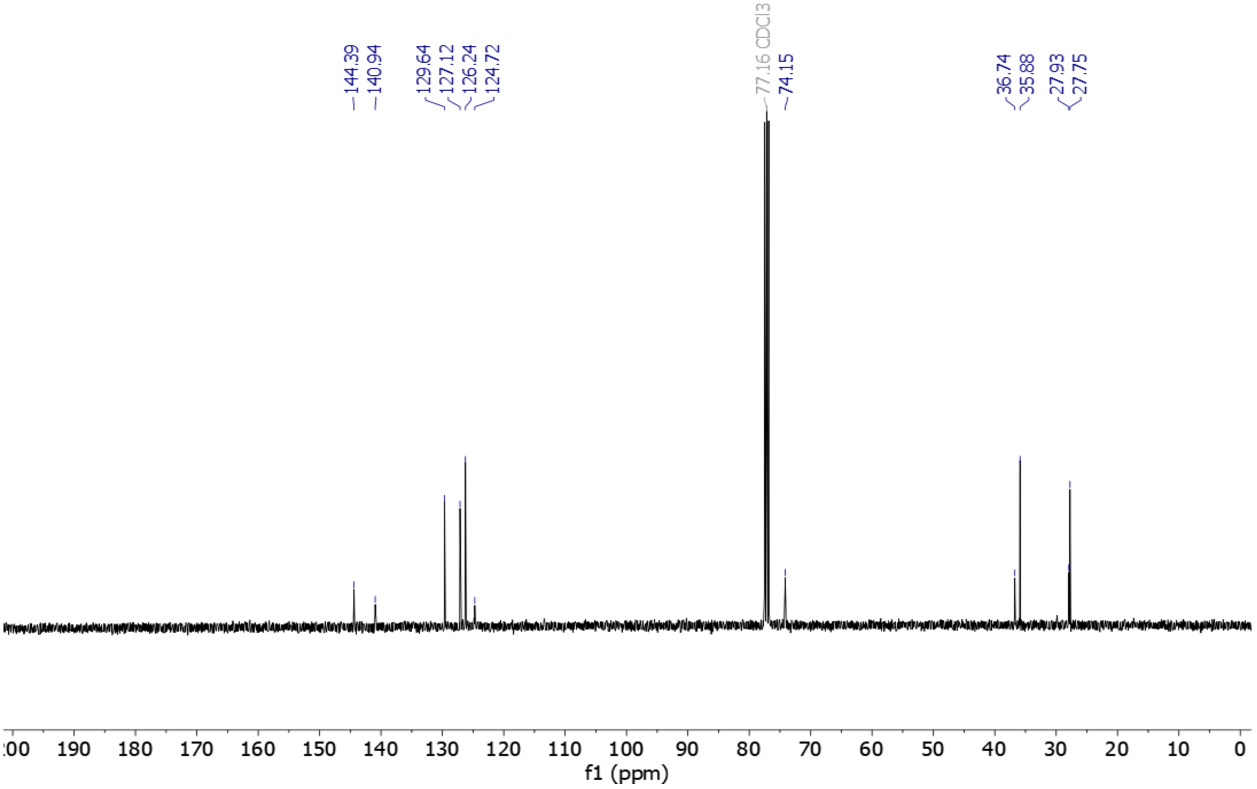

^{1}H NMR (500 MHz, $CDCl_3$) of *tert*-butyl (1-hydroxy-2,3-dihydro-1H-inden-5-yl)carbamate (2d)

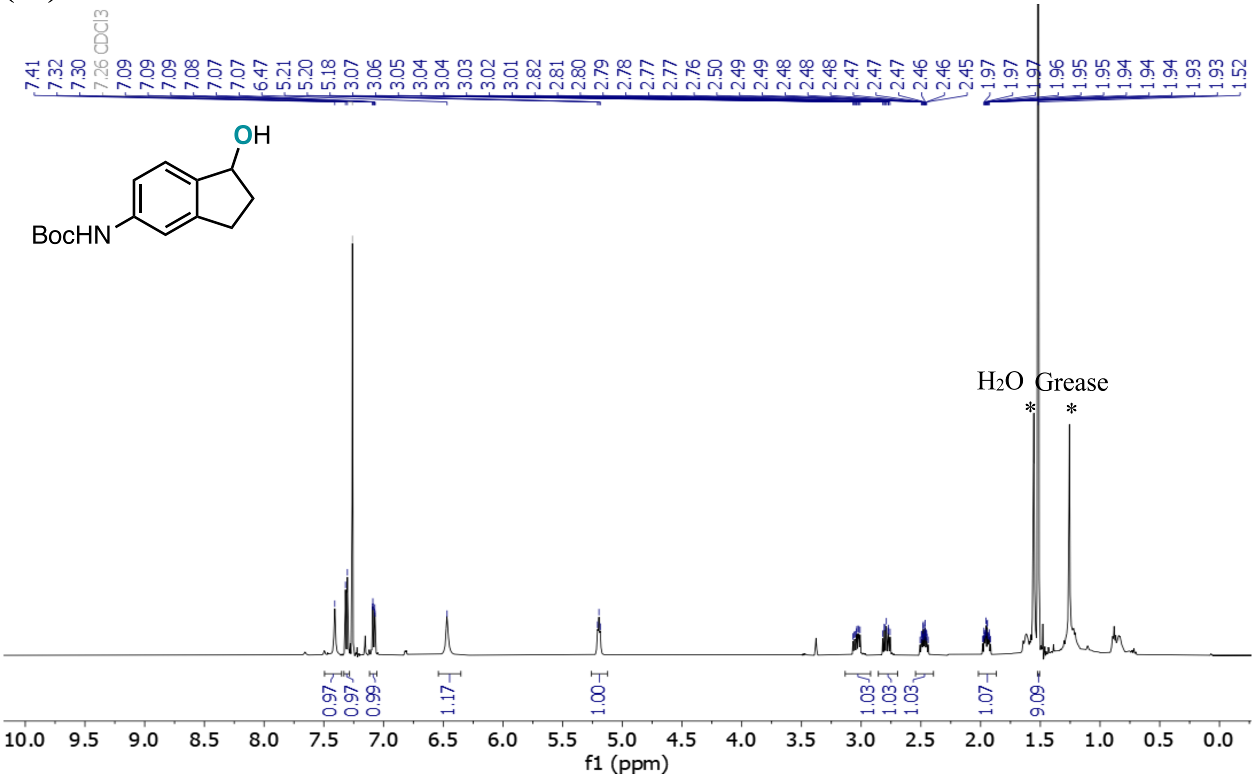

^{13}C NMR (126 MHz, $CDCl_3$) of *tert*-butyl (1-hydroxy-2,3-dihydro-1H-inden-5-yl)carbamate (2d)

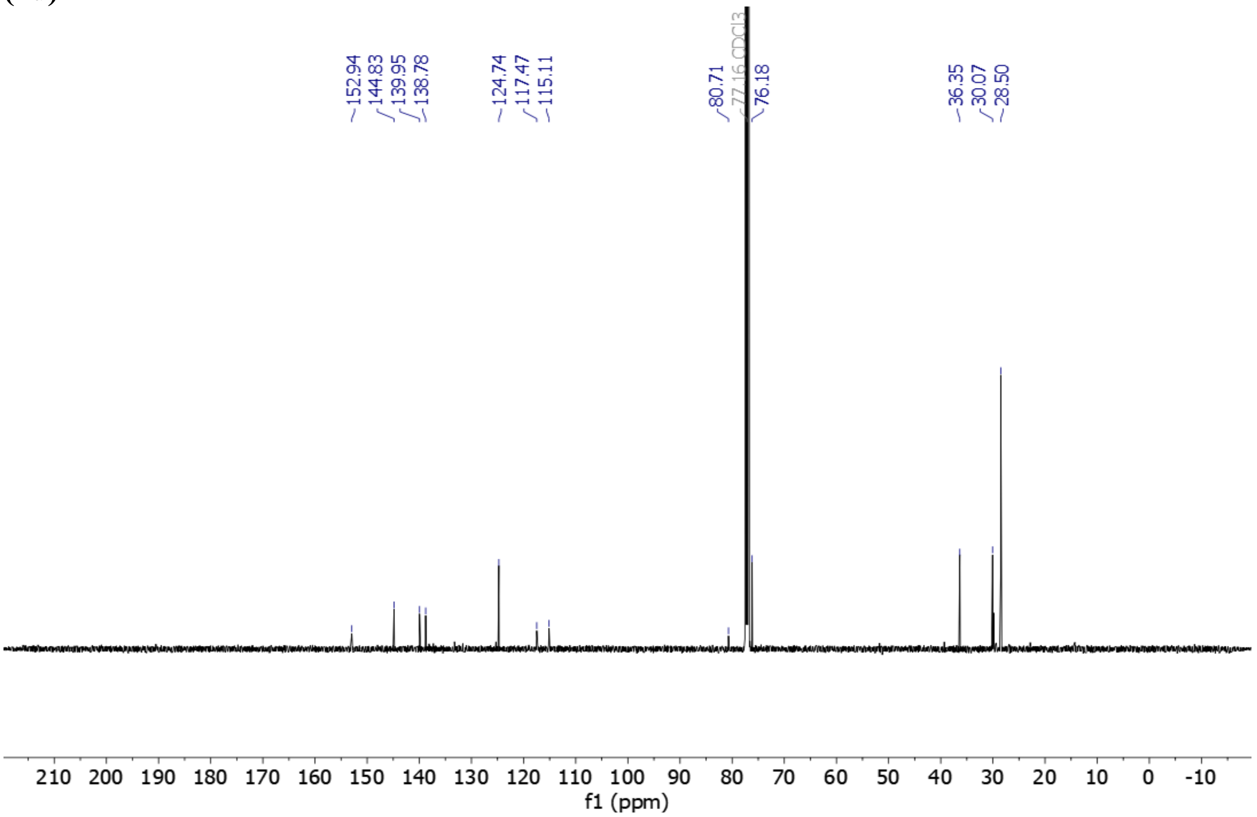

^{1}H–^{13}C HSQC (500 MHz, $CDCl_3$) of *tert*-butyl (1-hydroxy-2,3-dihydro-1H-inden-5-yl)carbamate (2d)

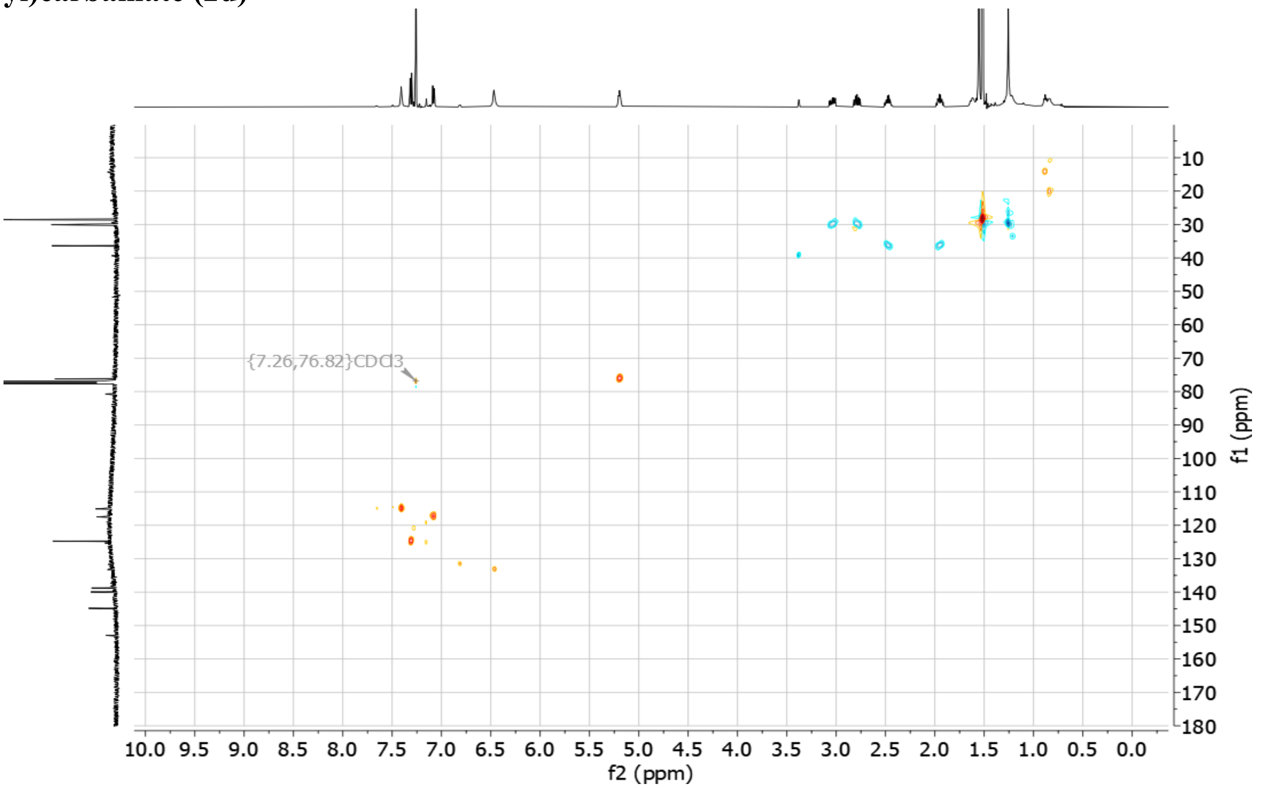

^{1}H–^{13}C HMBC (500 MHz, $CDCl_3$) of *tert*-butyl (1-hydroxy-2,3-dihydro-1H-inden-5-yl)carbamate (2d)

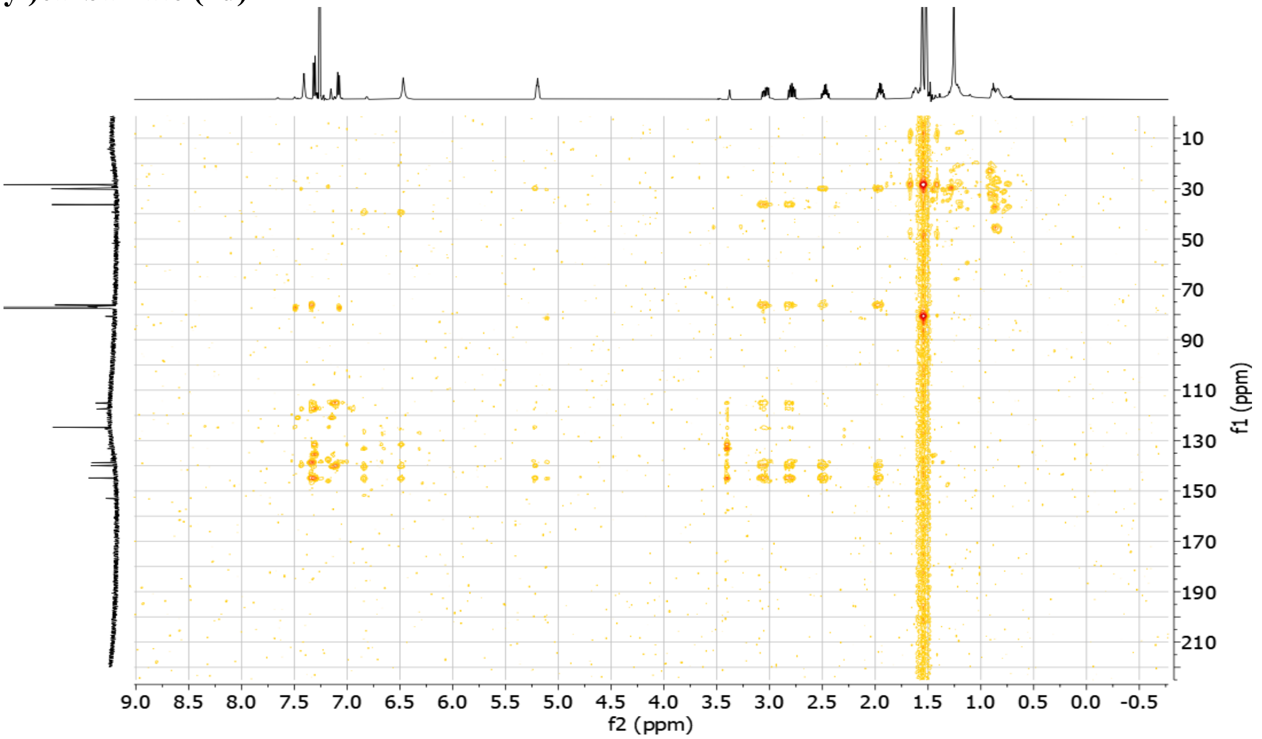

^{1}H NMR (500 MHz, $CDCl_3$) of 1-hydroxy-2,3-dihydro-1H-inden-5-yl trifluoromethane sulfonate (2e)

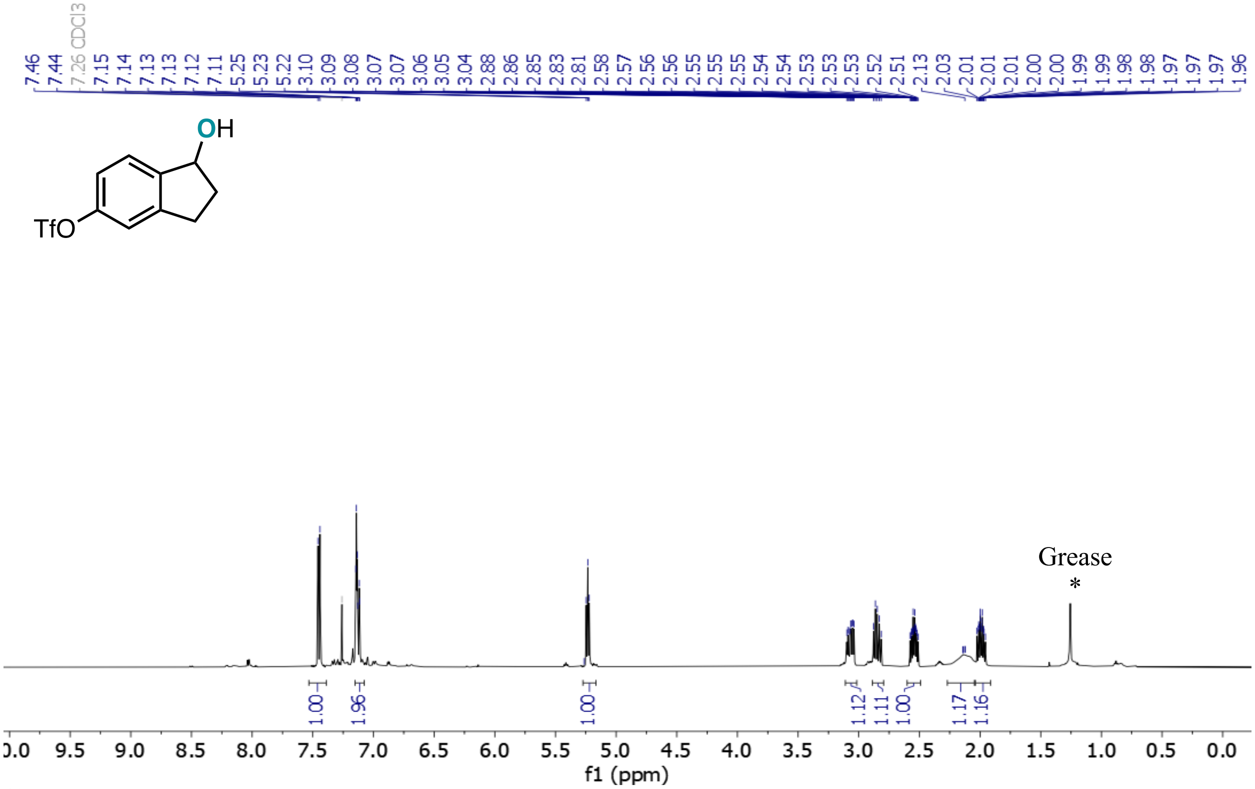

^{13}C NMR (126 MHz, $CDCl_3$) of 1-hydroxy-2,3-dihydro-1H-inden-5-yl trifluoromethane sulfonate (2e)

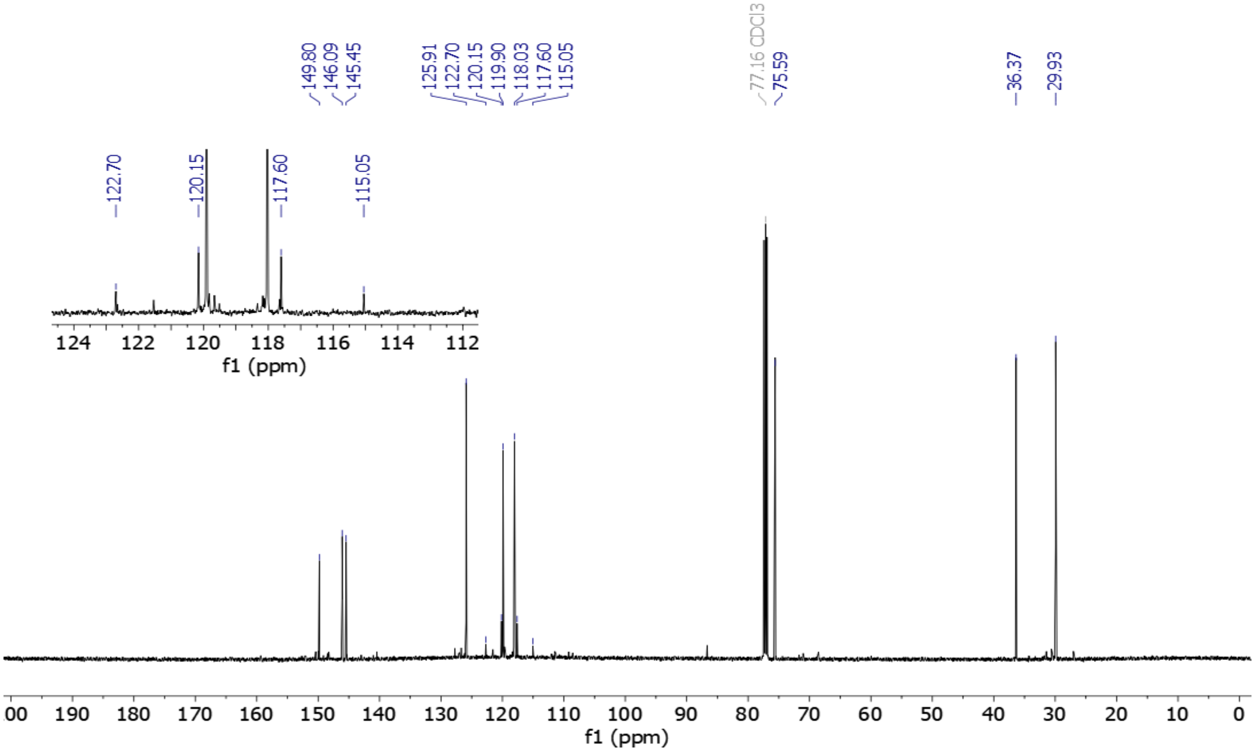

^{19}F NMR (471 MHz, $CDCl_3$) of 1-hydroxy-2,3-dihydro-1H-inden-5-yl trifluoromethane sulfonate (2e)

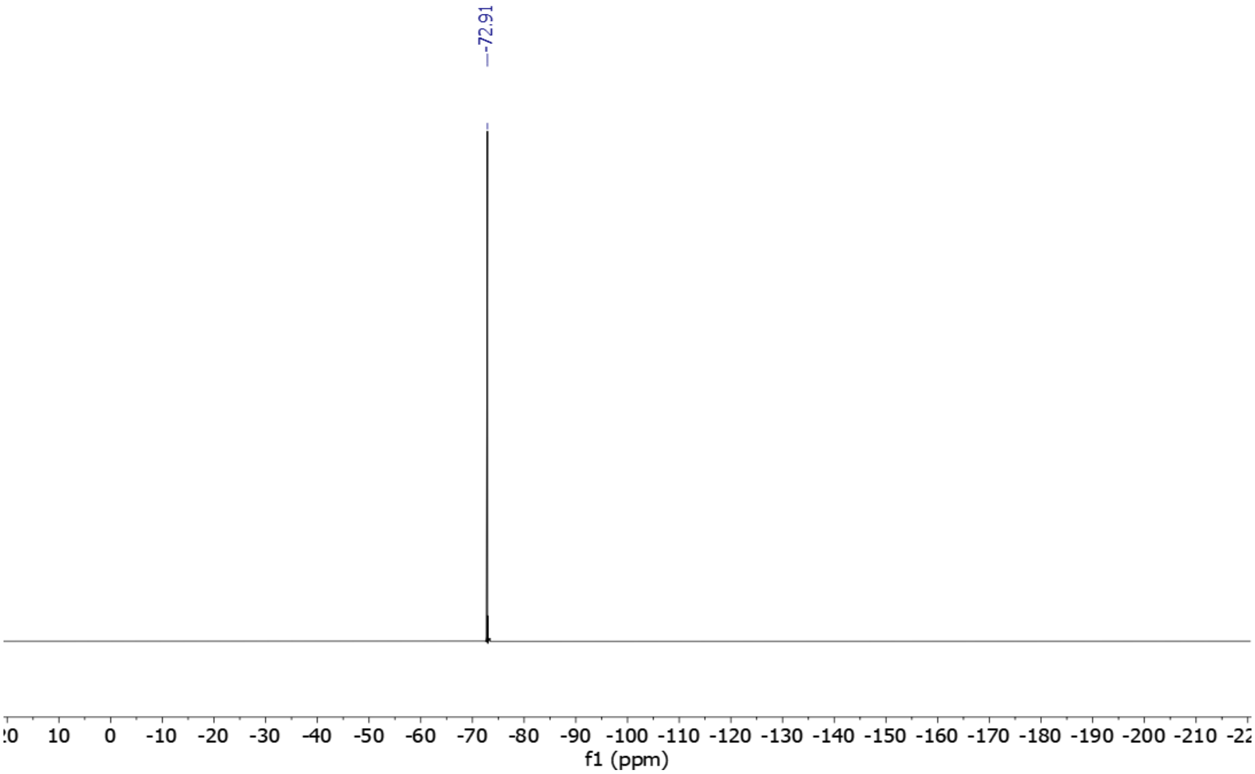

^{1}H–^{13}C HSQC (500 MHz, $CDCl_3$) of 1-hydroxy-2,3-dihydro-1H-inden-5-yl trifluoromethane sulfonate (2e)

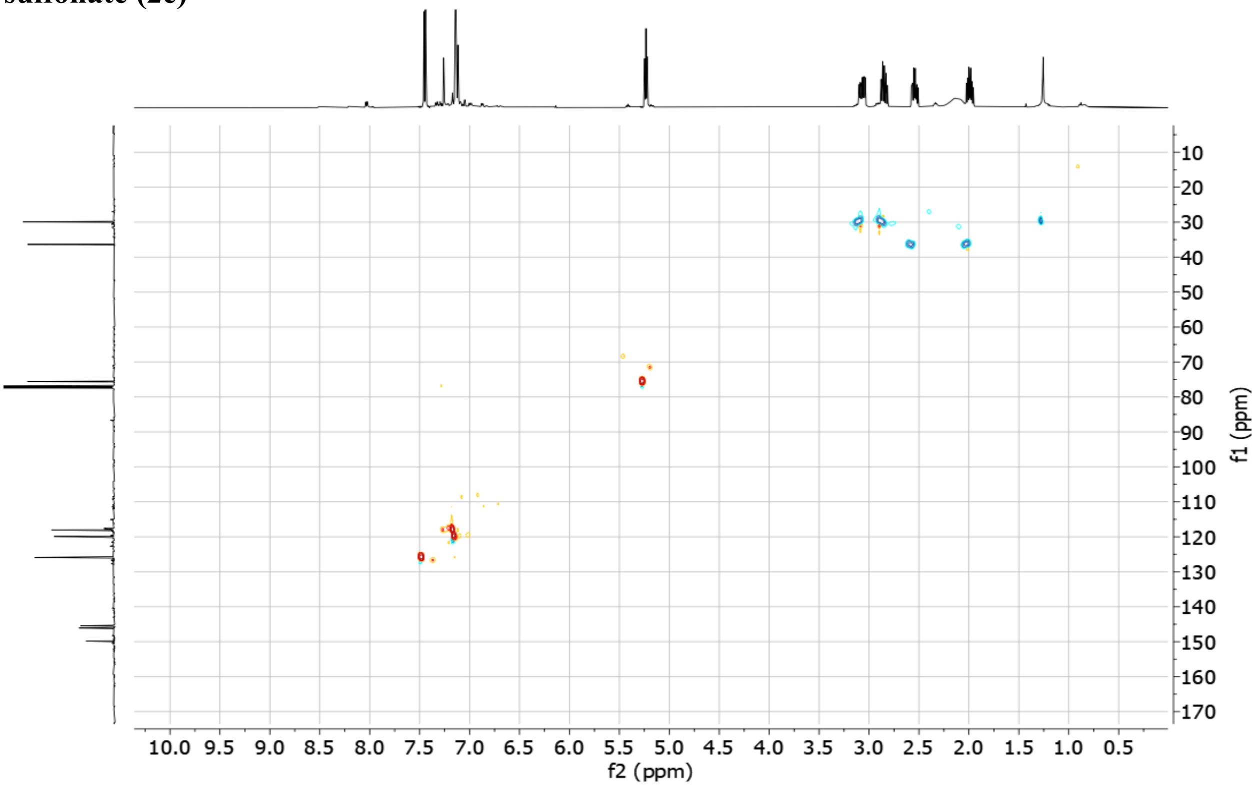

^{1}H–^{13}C HMBC (500 MHz, $CDCl_3$) of 1-hydroxy-2,3-dihydro-1H-inden-5-yl trifluoromethane sulfonate (2e)

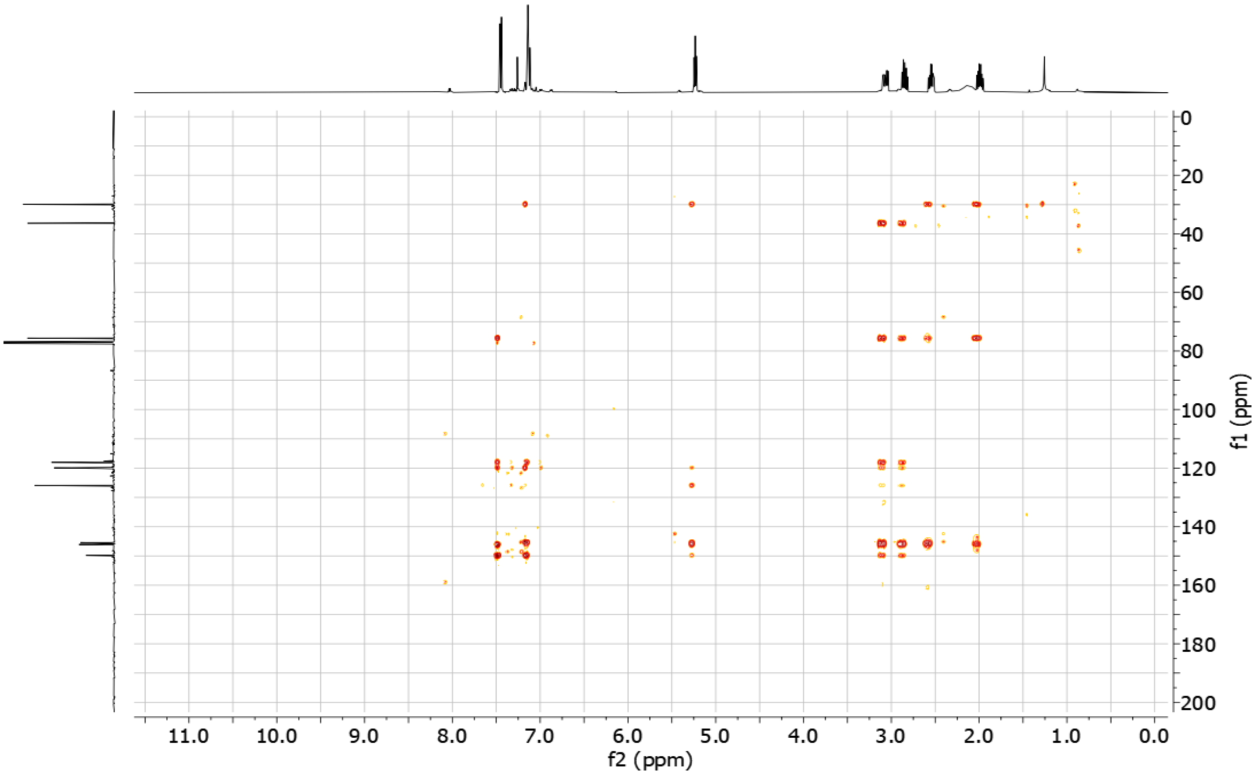

^{1}H NMR (400 MHz, $CDCl_3$) of 1-(6-(tert-butyl)-3-hydroxy-1,1-dimethyl-2,3-dihydro-1H-inden-4-yl)ethan-1-one (2f)

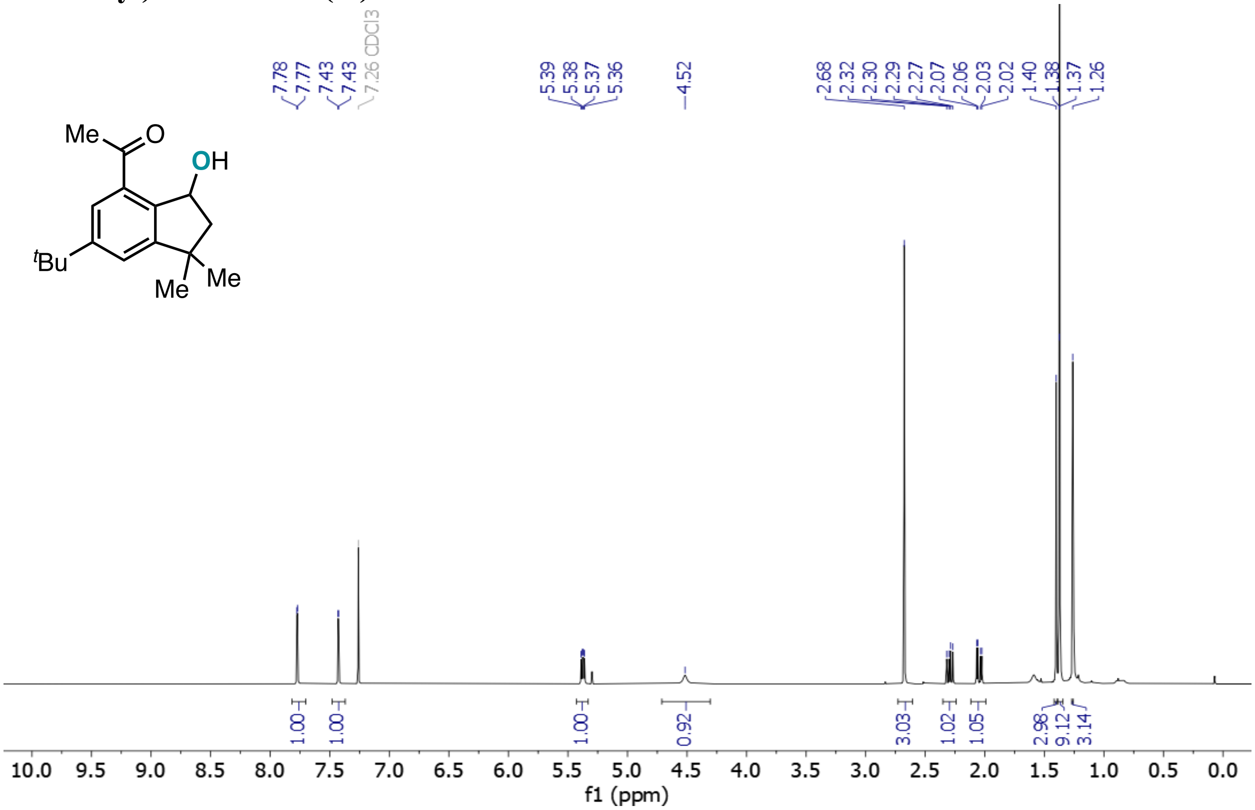

^{13}C NMR (101 MHz, $CDCl_3$) of 1-(6-(tert-butyl)-3-hydroxy-1,1-dimethyl-2,3-dihydro-1H-inden-4-yl)ethan-1-one (2f)

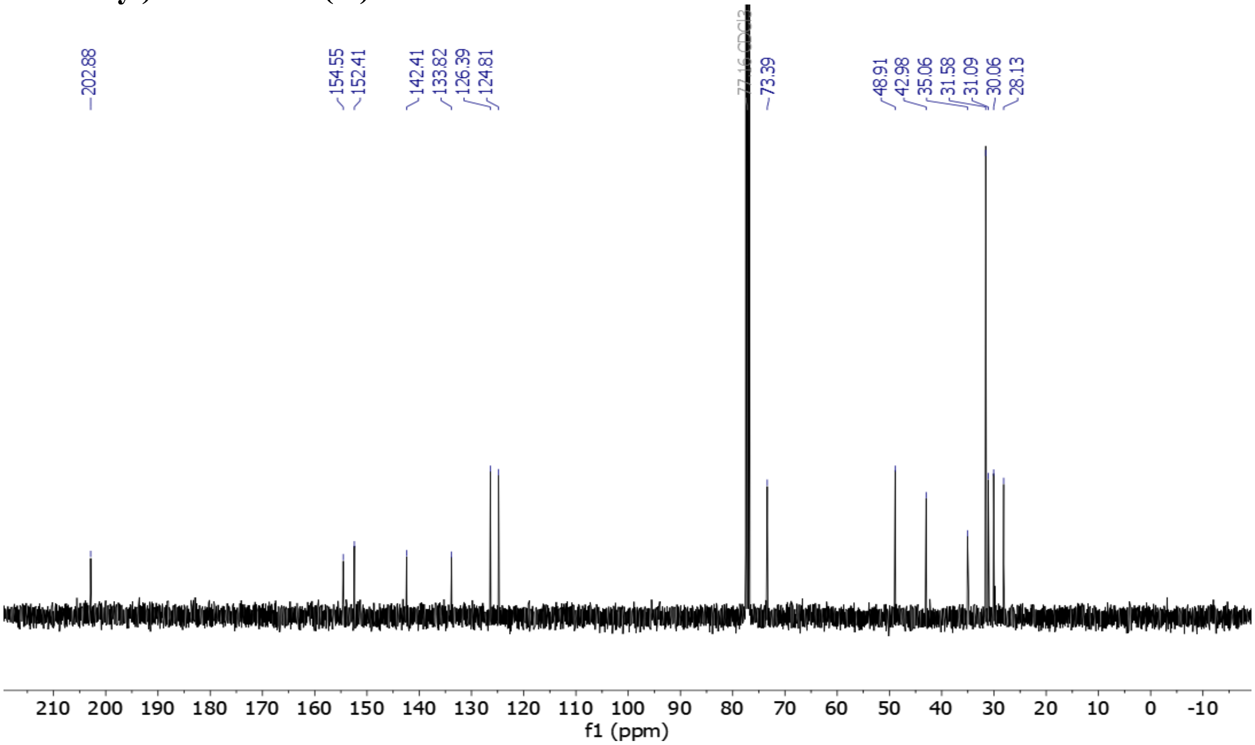

^{1}H NMR (500 MHz, $CDCl_3$) of 1-phenylethanol (2g)

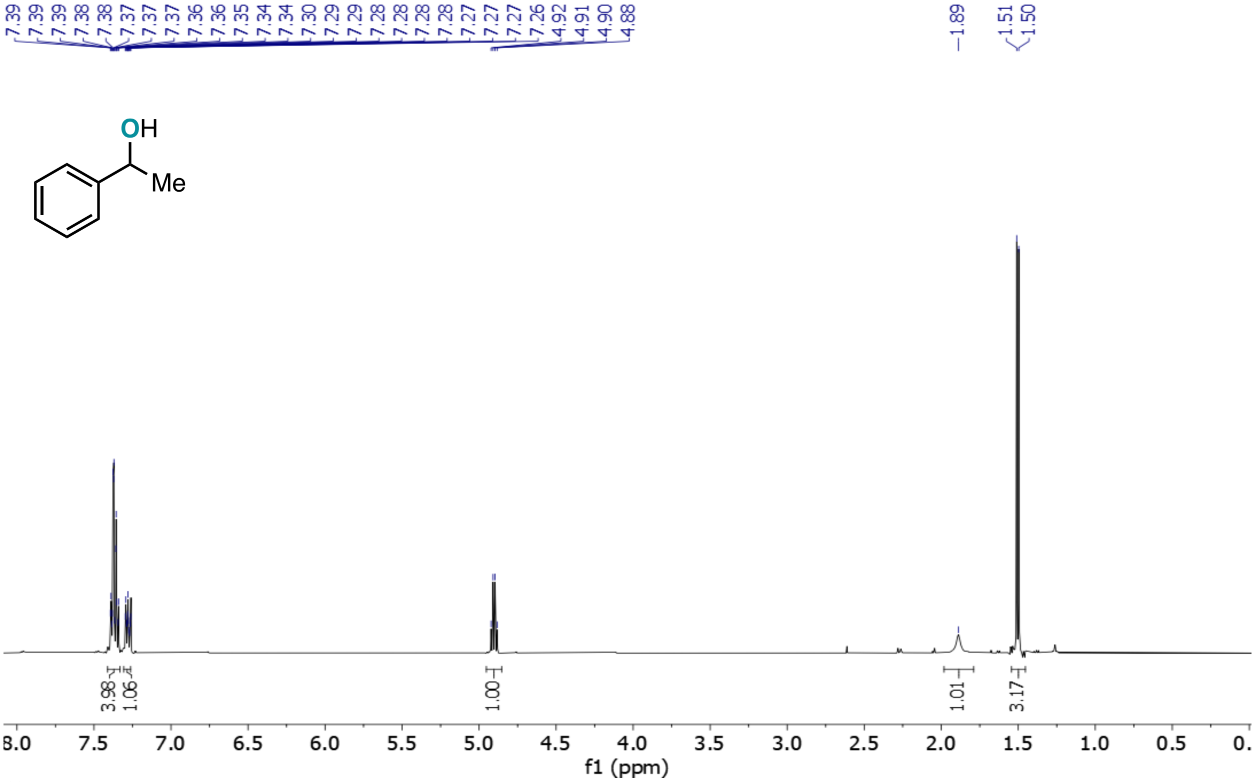

^{13}C NMR (126 MHz, $CDCl_3$) of 1-phenylethanol (2g)

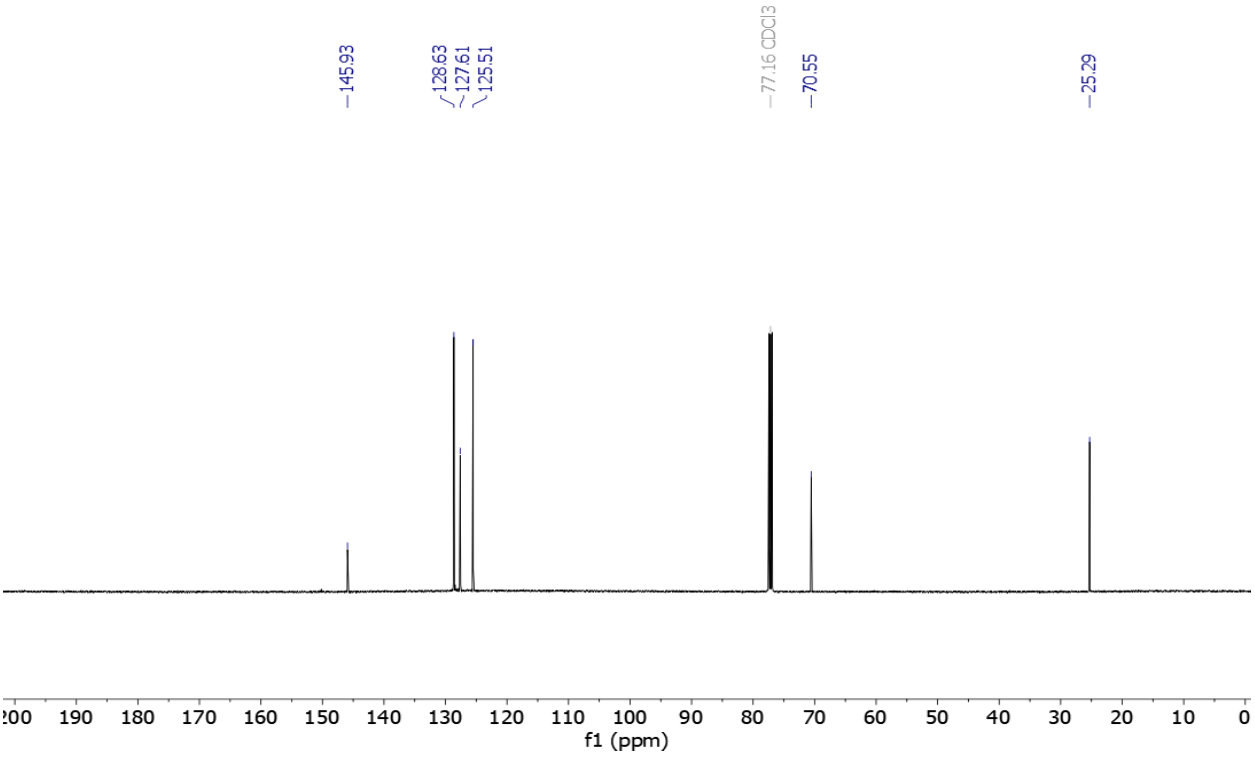

^{1}H NMR (500 MHz, $CDCl_3$) of 1-(4-methoxyphenyl)ethan-1-ol (2h)

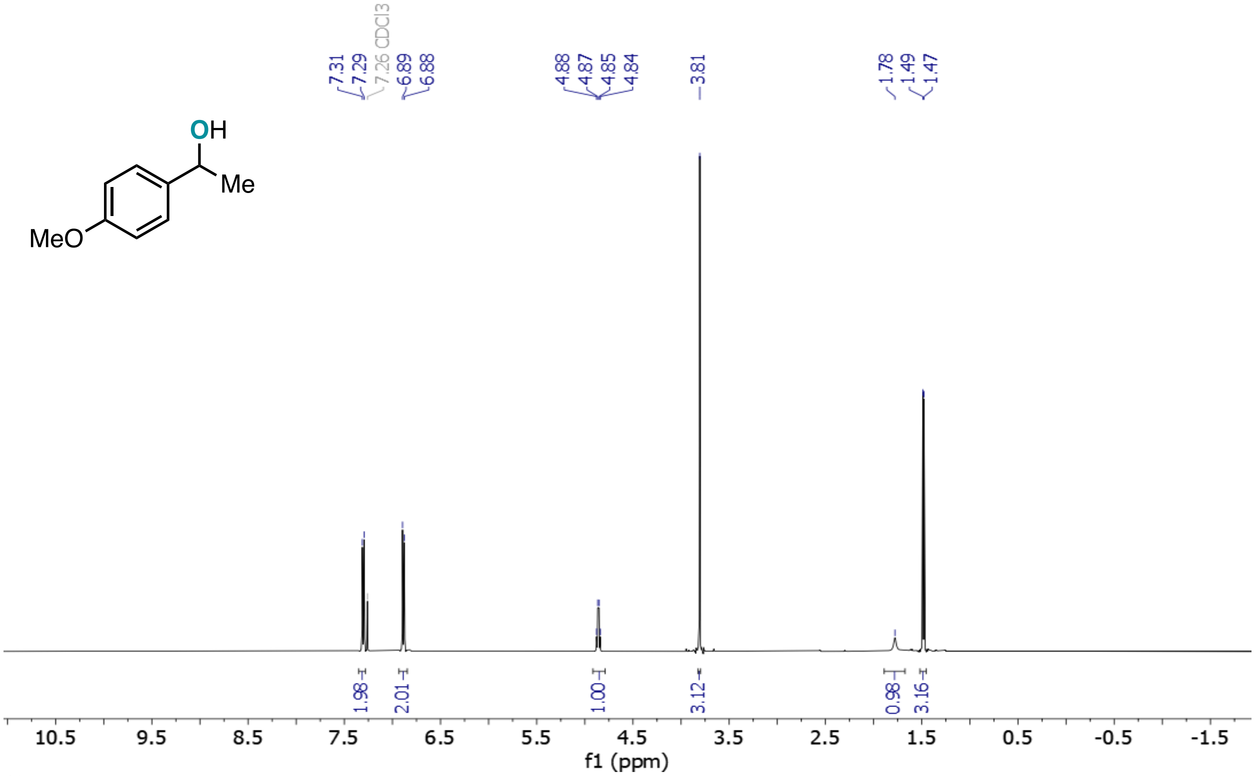

^{13}C NMR (126 MHz, $CDCl_3$) of 1-(4-methoxyphenyl)ethan-1-ol (2h)

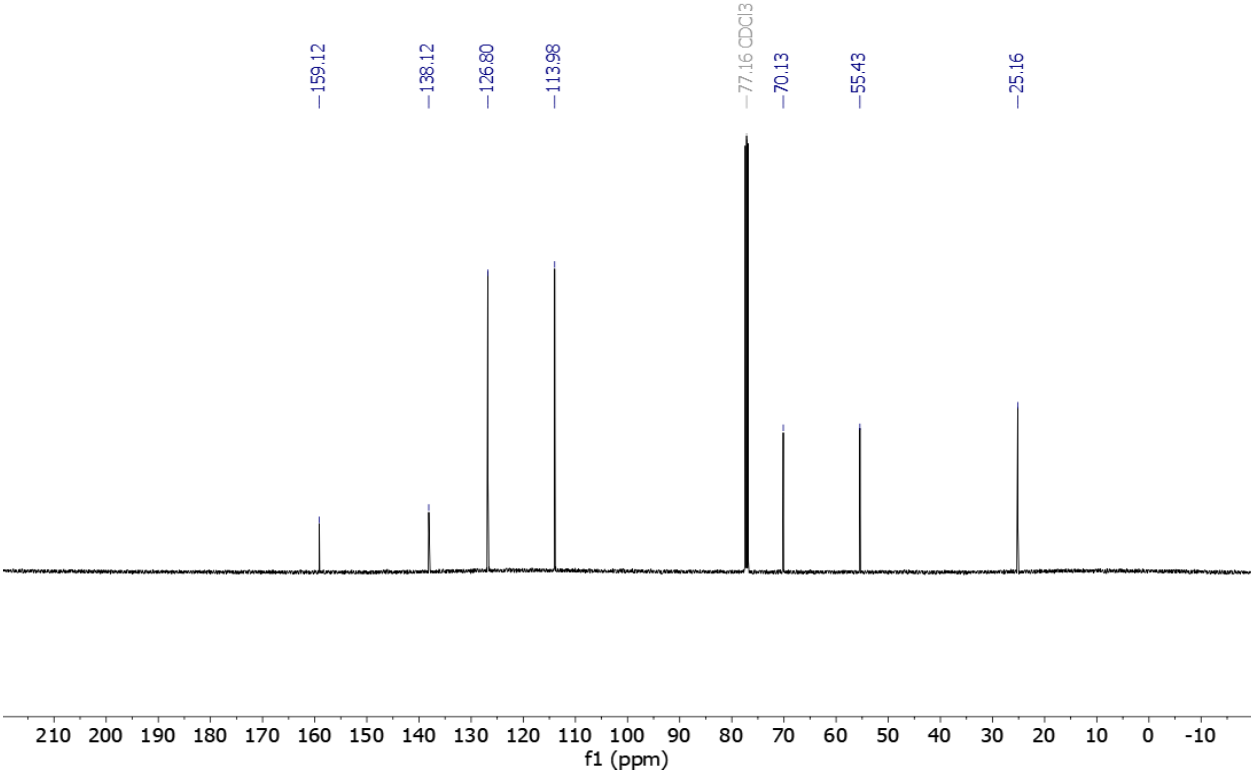

^{1}H NMR (400 MHz, CDCl$_3$) of 1-(4-(tert-butyl)phenyl)ethan-1-ol (2i)

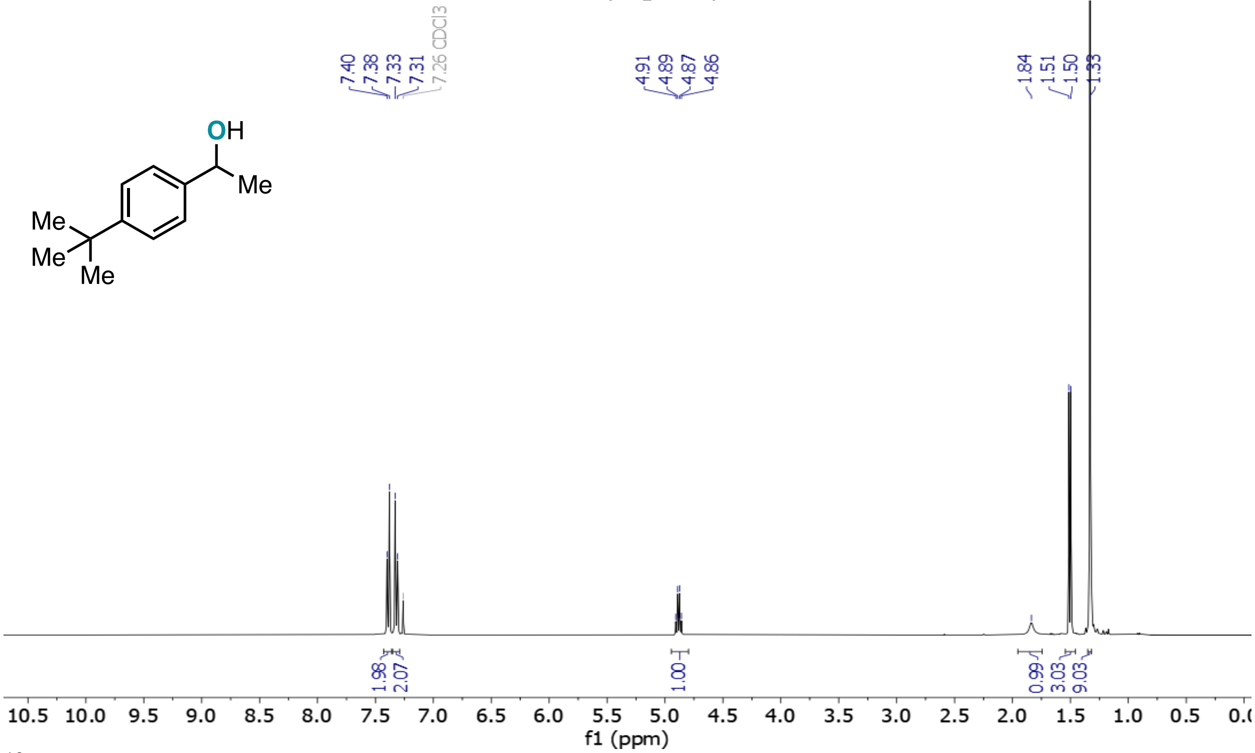

^{13}C NMR (101 MHz, CDCl$_3$) of 1-(4-(tert-butyl)phenyl)ethan-1-ol (2i)

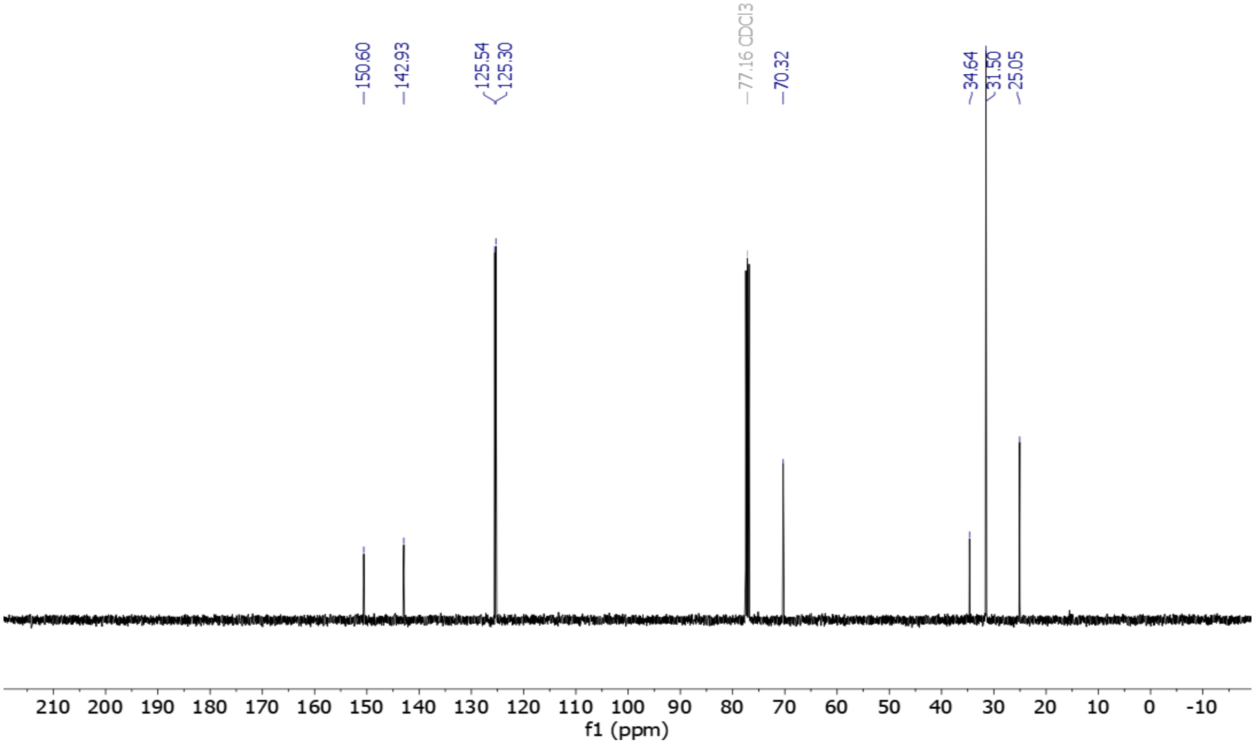

^{1}H NMR (500 MHz, $CDCl_3$) of 4-(1-hydroxyethyl)phenyl acetate (2j)

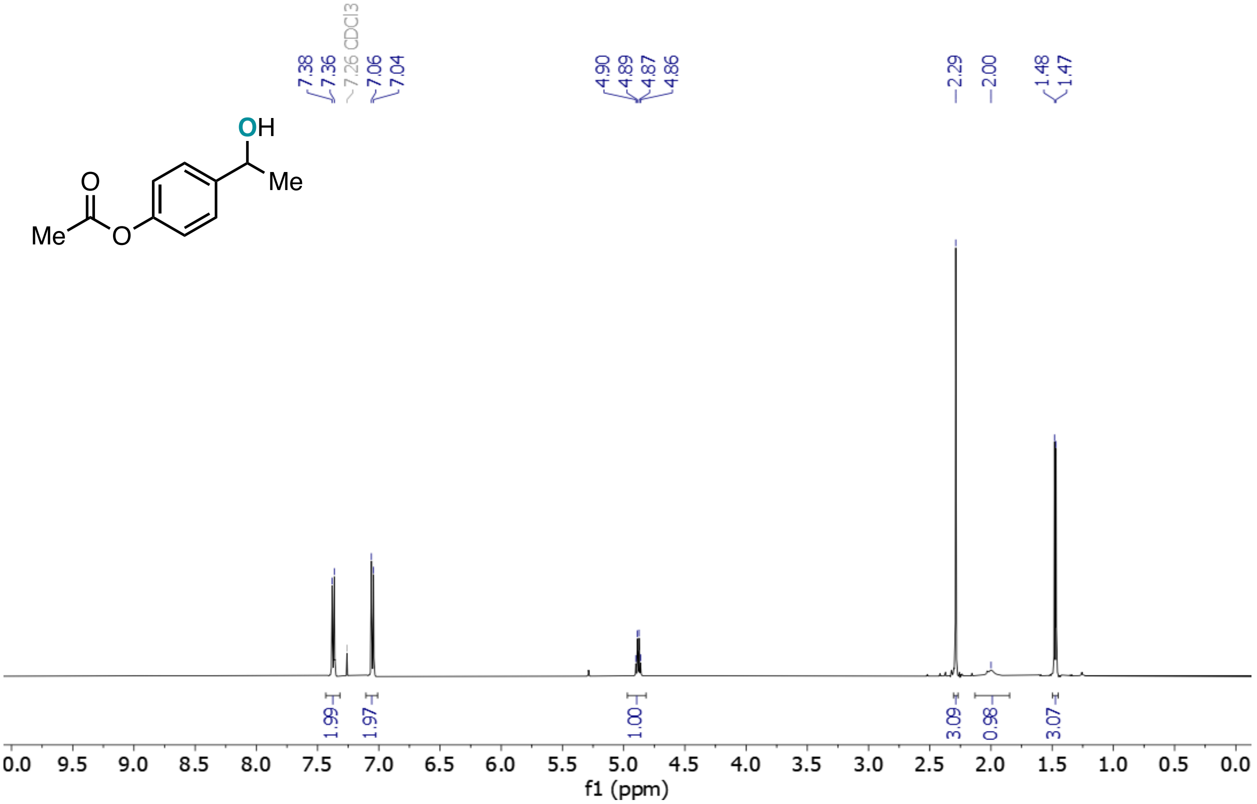

^{13}C NMR (126 MHz, $CDCl_3$) of 4-(1-hydroxyethyl)phenyl acetate (2j)

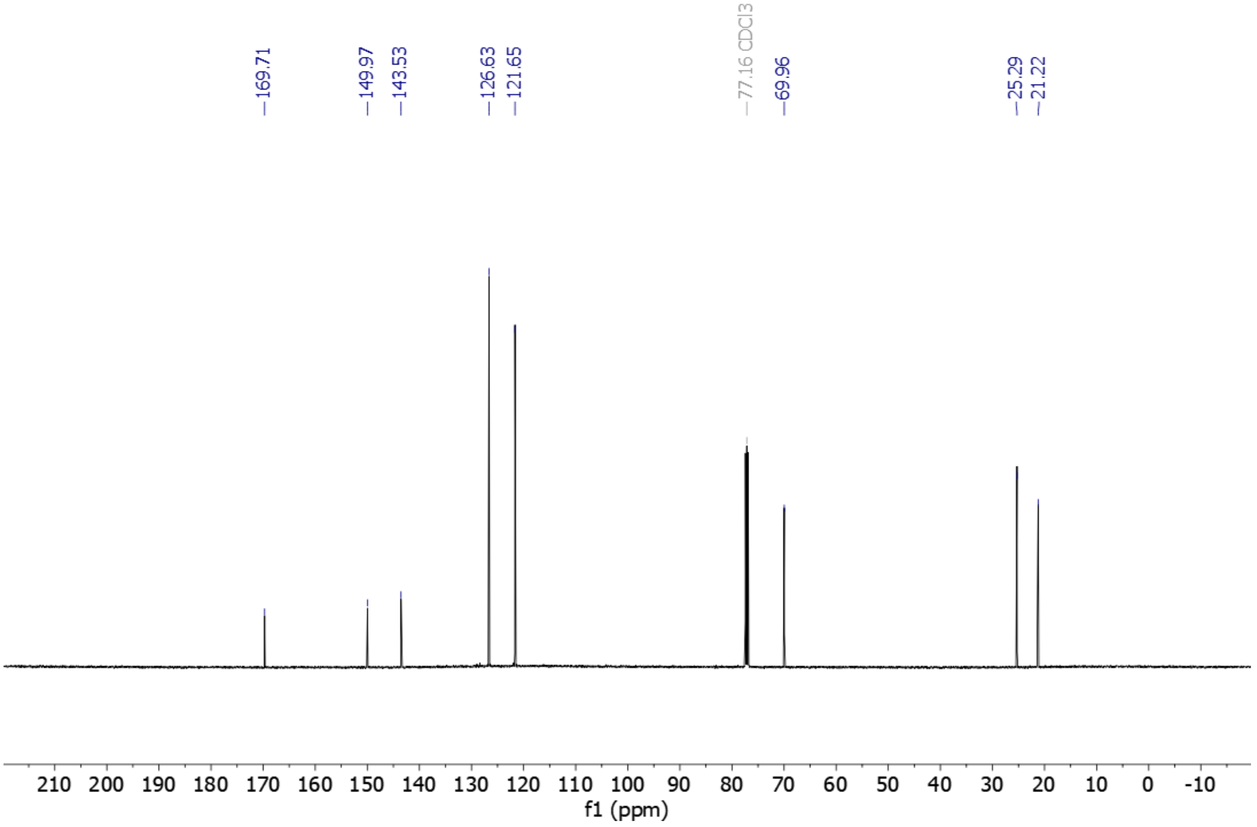

^{1}H NMR (400 MHz, $CDCl_3$) of 1-(4-(trifluoromethyl)phenyl)ethan-1-ol (2k)

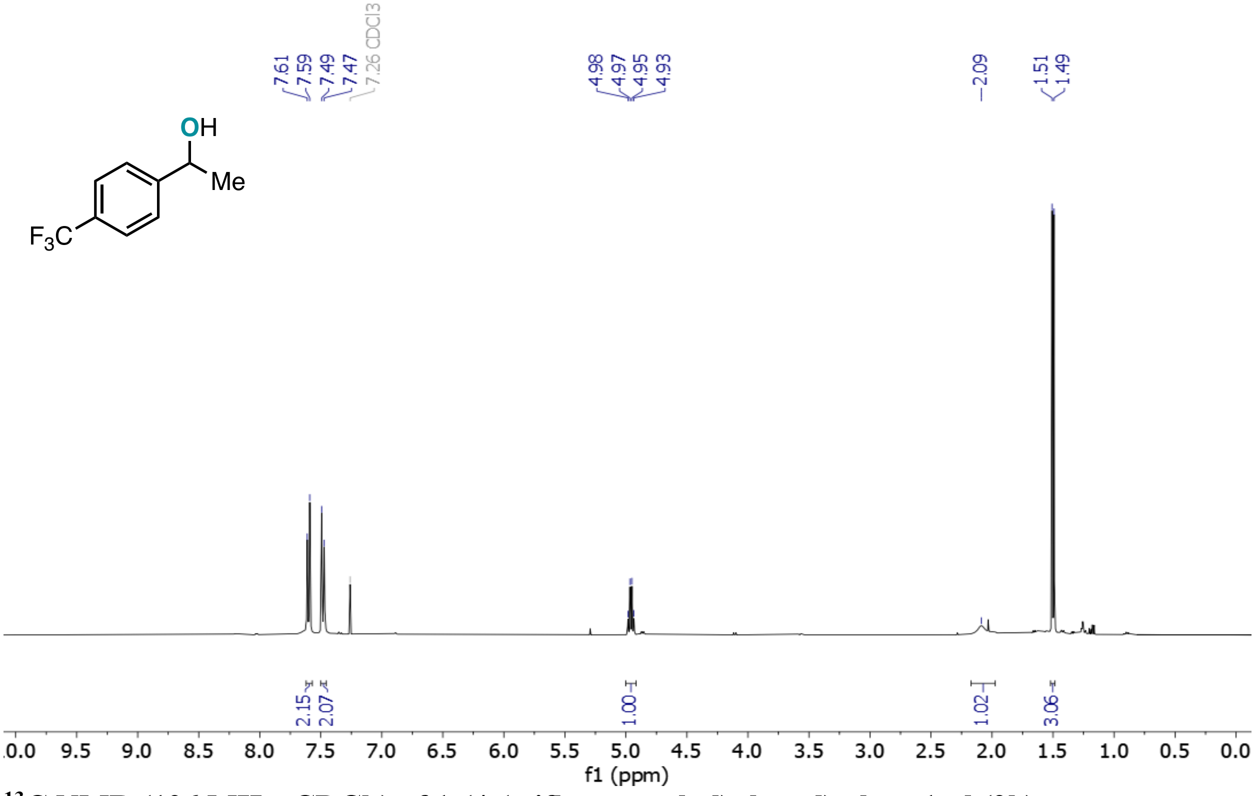

^{13}C NMR (126 MHz, $CDCl_3$) of 1-(4-(trifluoromethyl)phenyl)ethan-1-ol (2k)

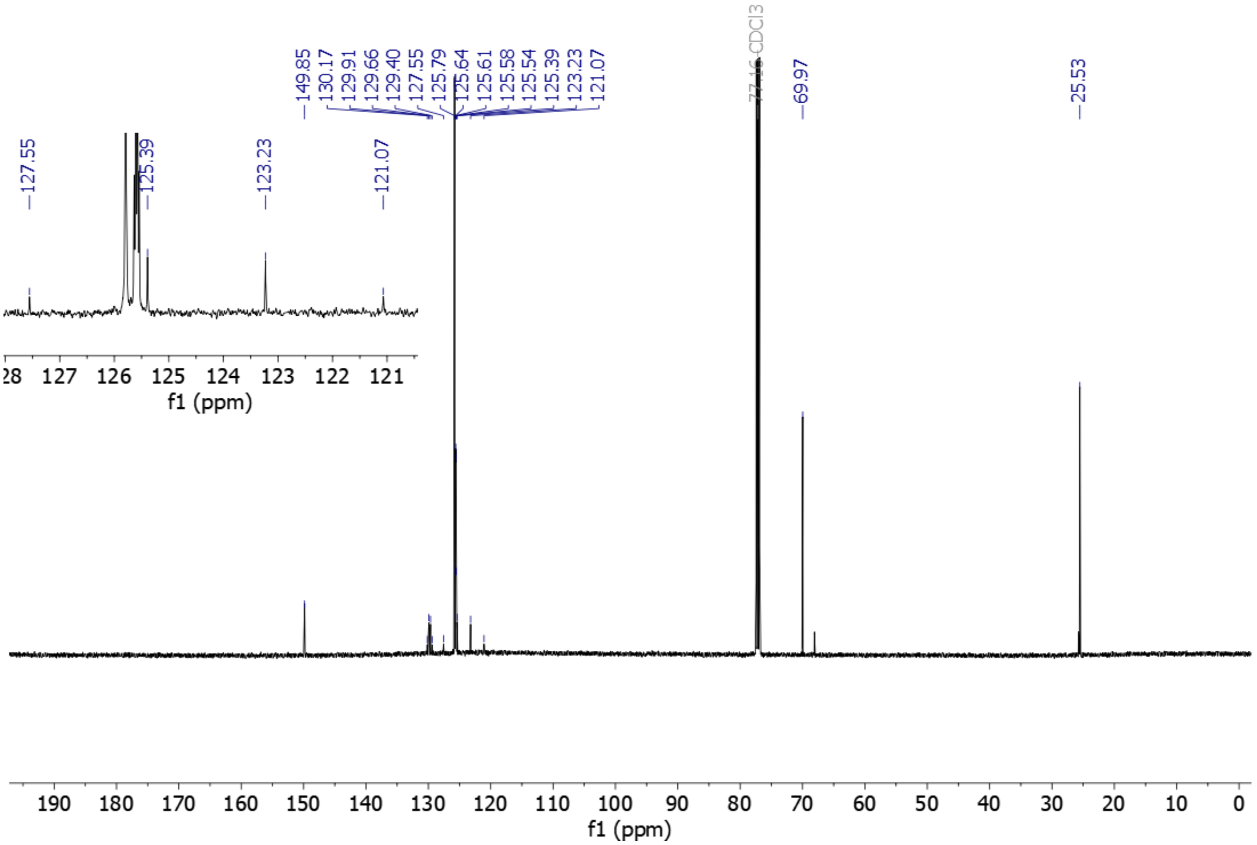

^{19}F NMR (377 MHz, $CDCl_3$) of 1-(4-(trifluoromethyl)phenyl)ethan-1-ol (2k)

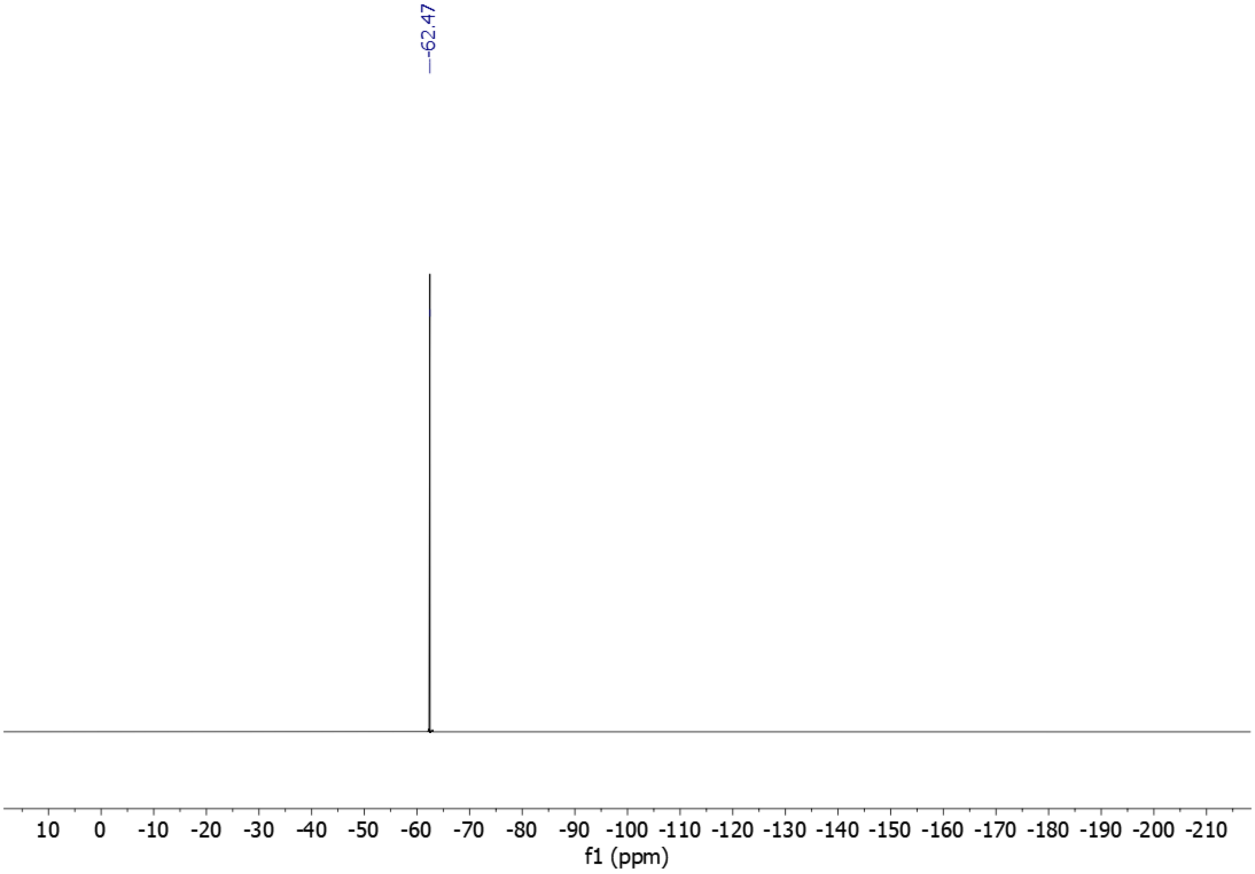

^{1}H NMR (500 MHz, $CDCl_3$) of 1-(4-chlorophenyl)ethan-1-ol (2l)

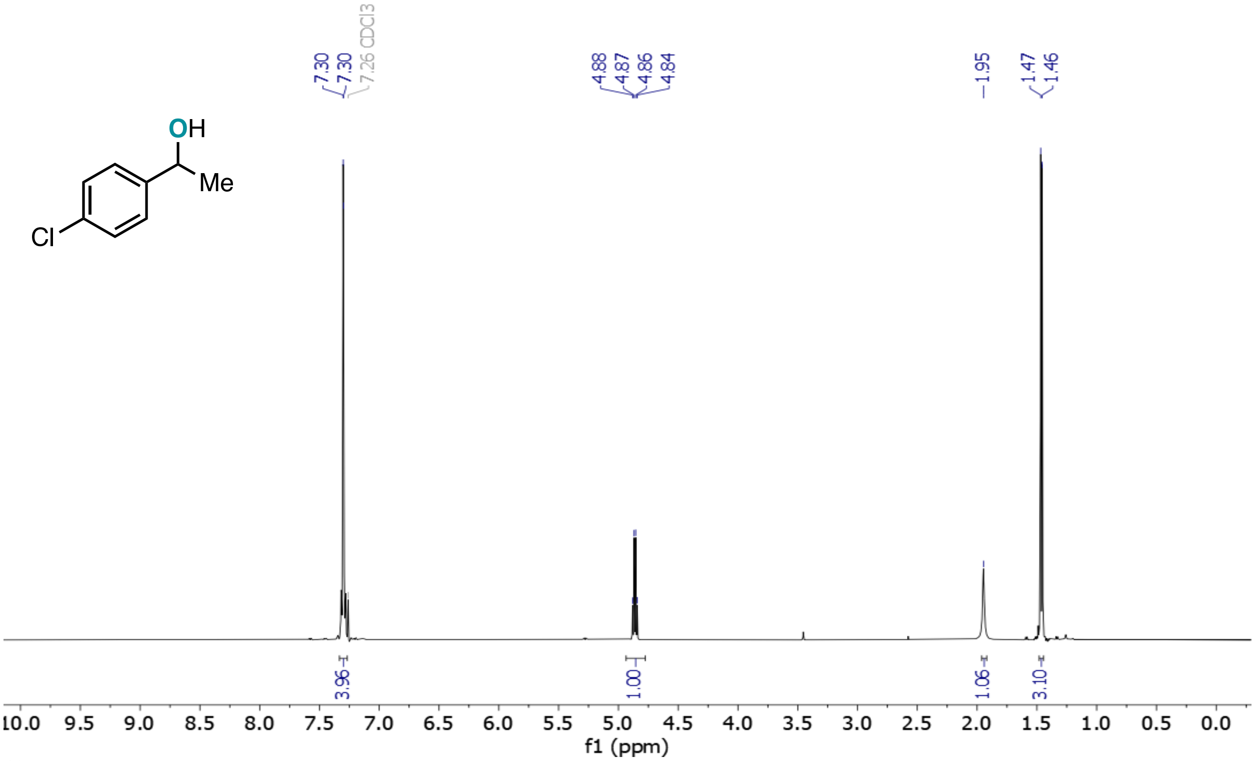

^{13}C NMR (126 MHz, $CDCl_3$) of 1-(4-chlorophenyl)ethan-1-ol (2l)

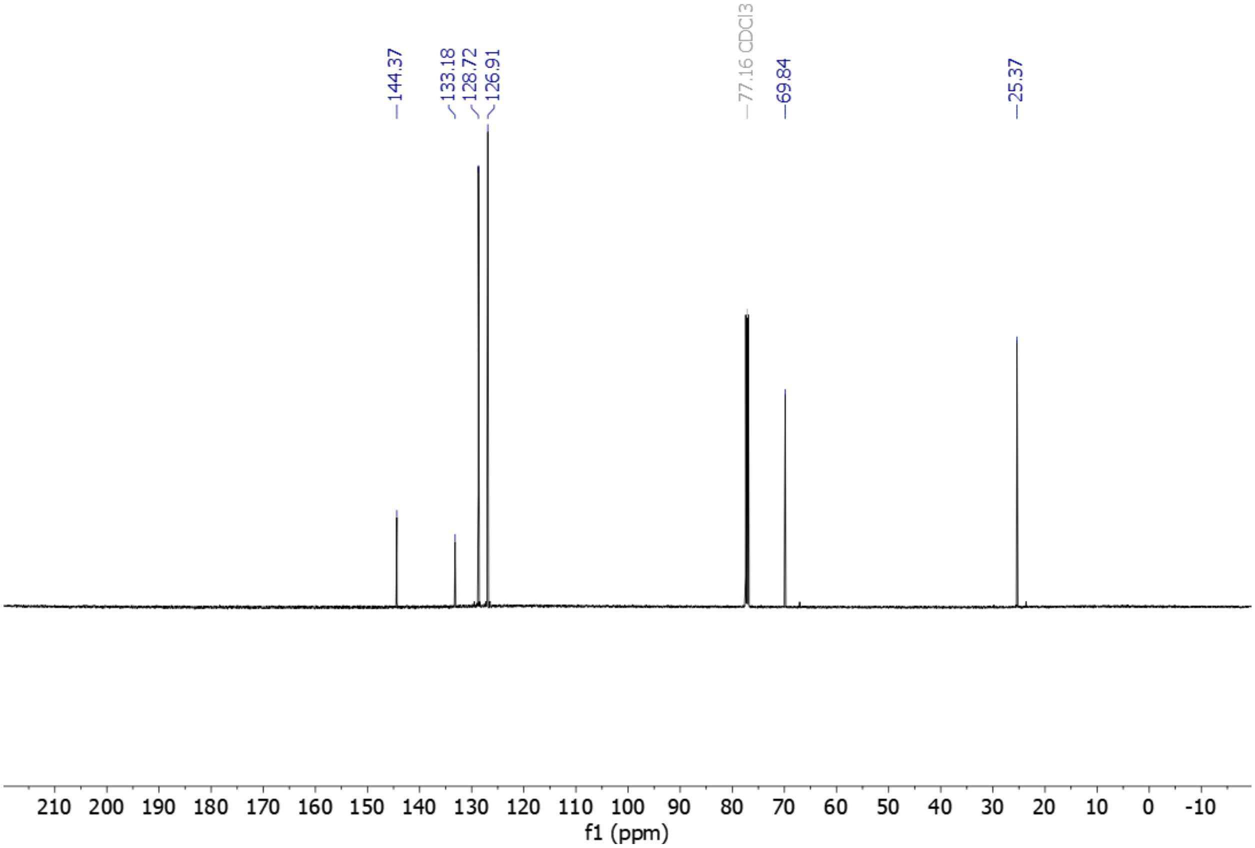

^{1}H NMR (500 MHz, CDCl$_3$) of 1-(4-fluorophenyl)ethan-1-ol (2m)

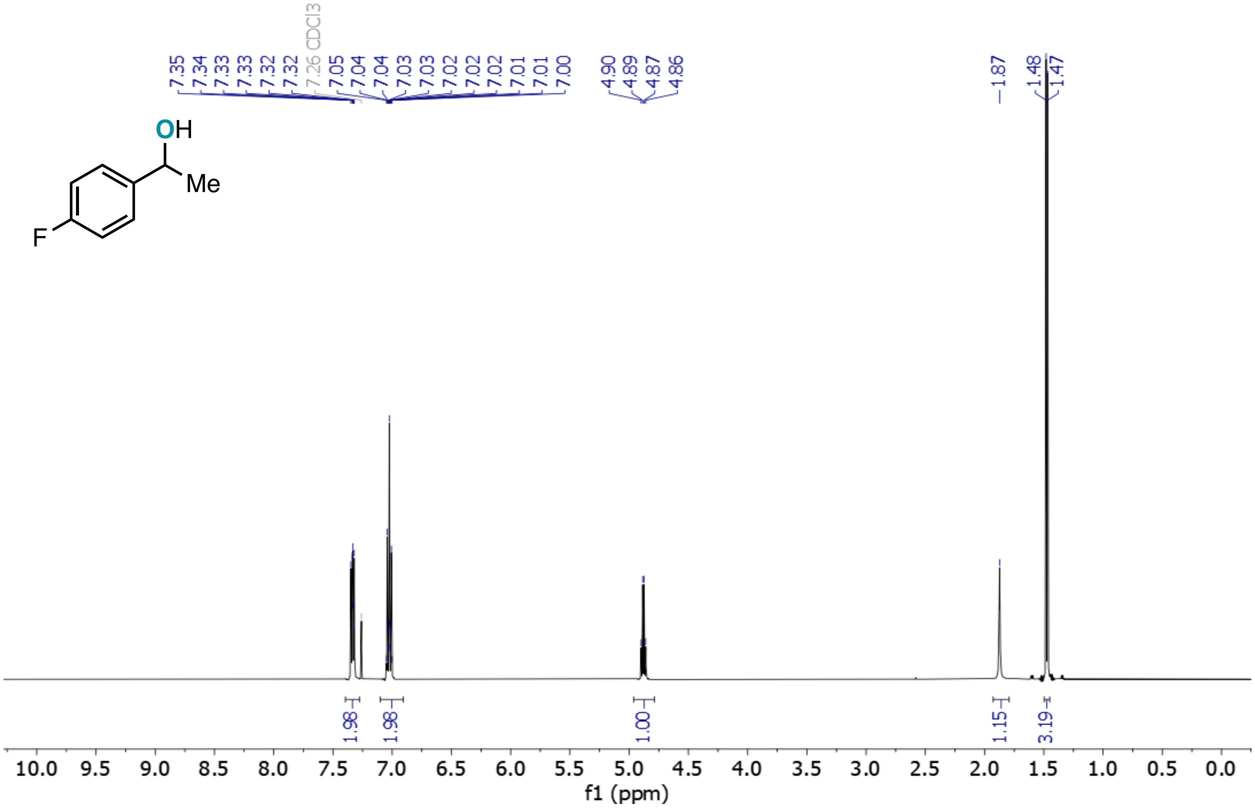

^{13}C NMR (126 MHz, CDCl$_3$) of 1-(4-fluorophenyl)ethan-1-ol (2m)

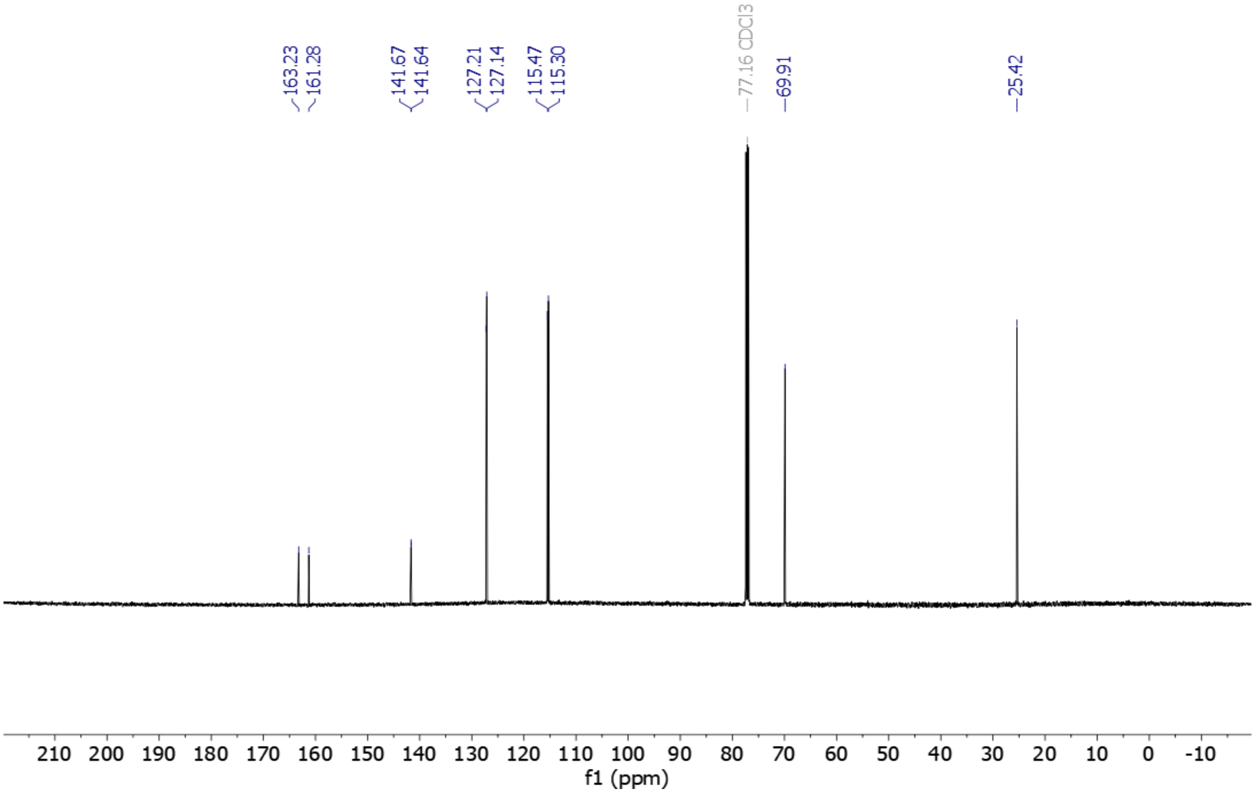

^{19}F NMR (471 MHz, $CDCl_3$) of 1-(4-fluorophenyl)ethan-1-ol (2m)

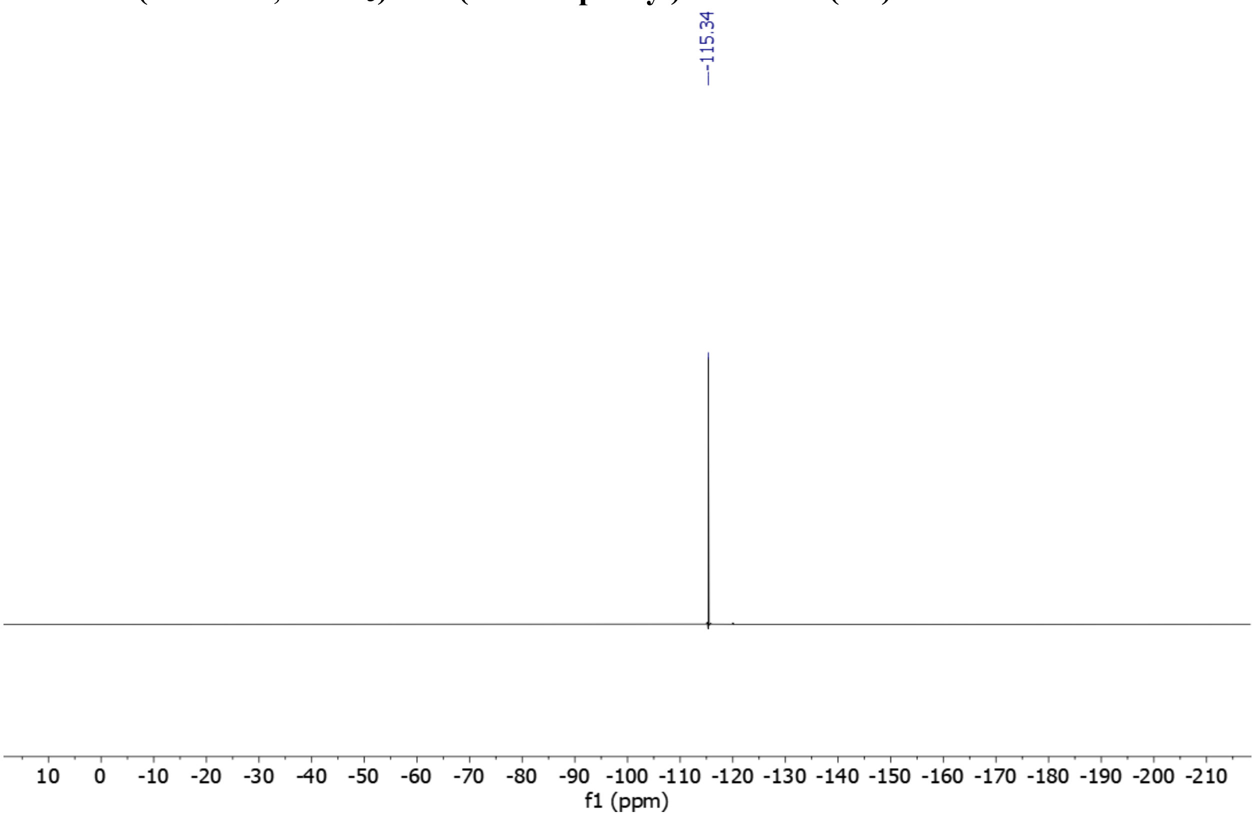

^{1}H NMR (400 MHz, $CDCl_3$) of 1-(4-(4,4,5,5-tetramethyl-1,3,2-dioxaborolan-2-yl)phenyl)ethan-1-ol (2n)

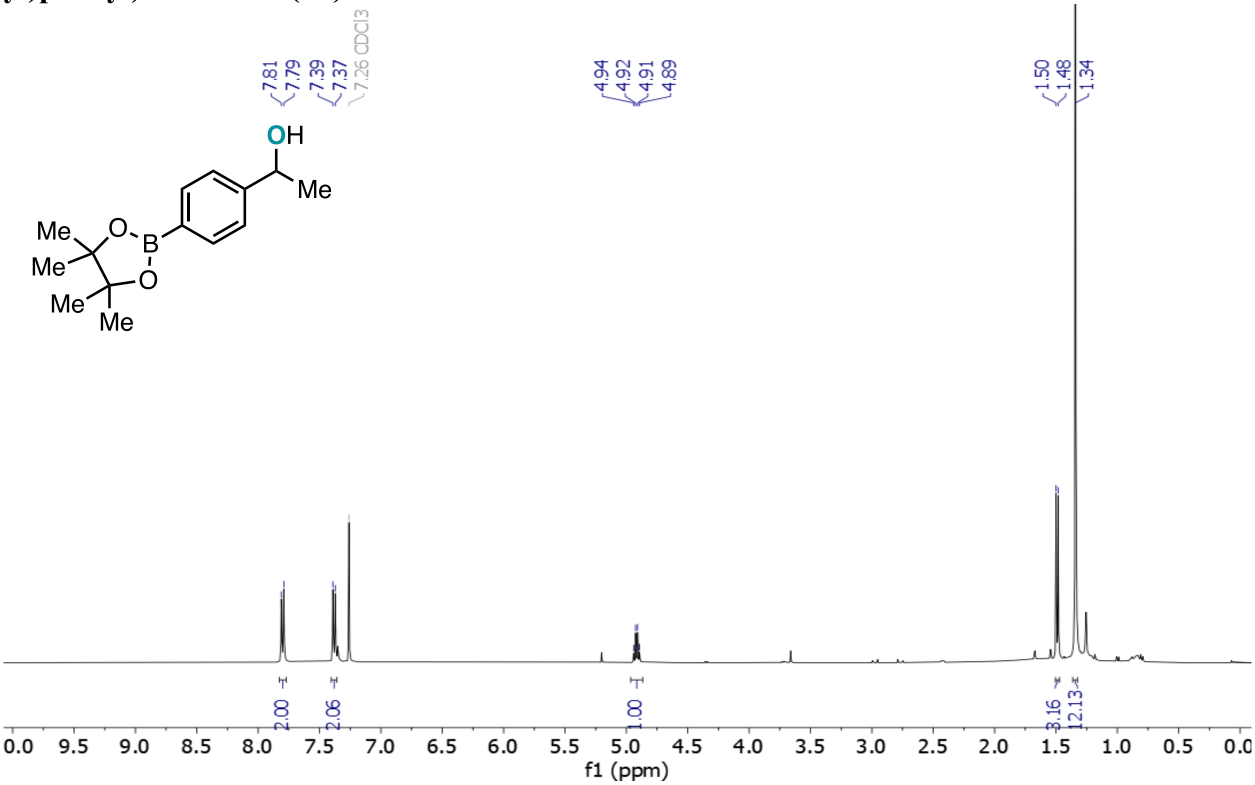

^{13}C NMR (101 MHz, $CDCl_3$) of 1-(4-(4,4,5,5-tetramethyl-1,3,2-dioxaborolan-2-yl)phenyl) ethan-1-ol (2n)

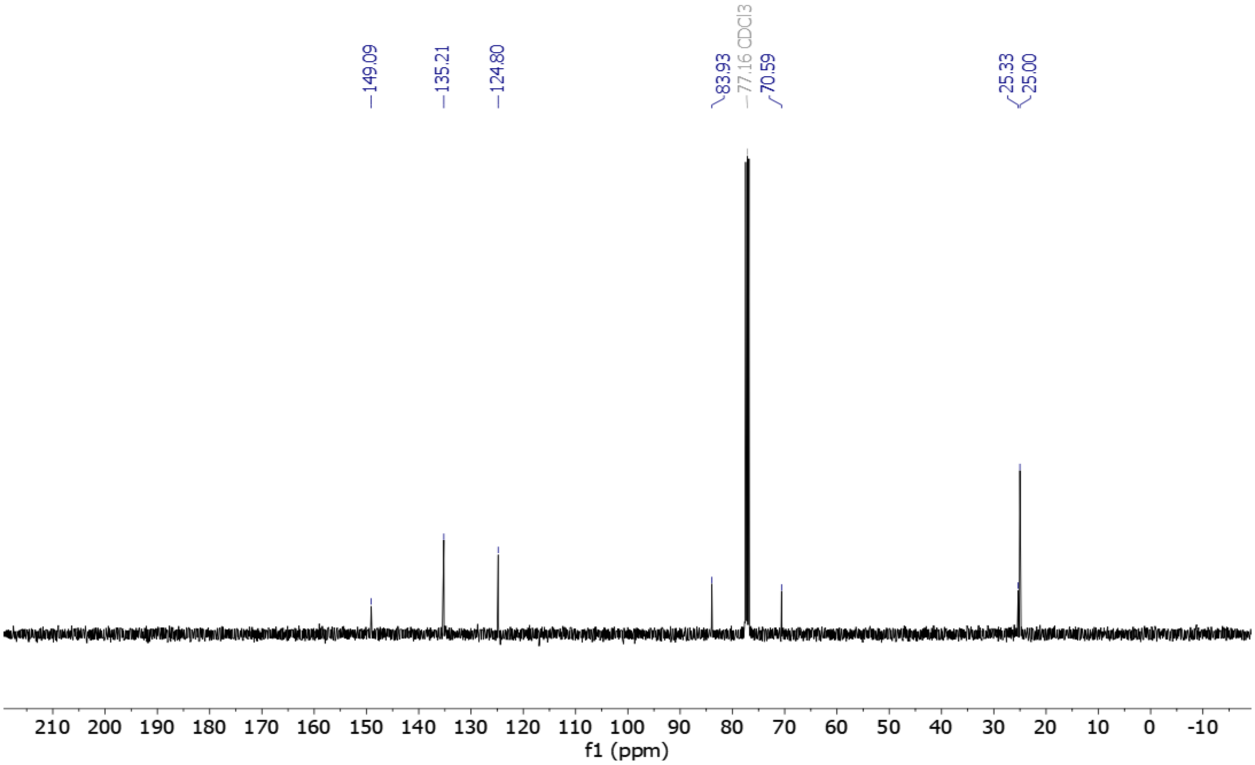

^{1}H NMR (500 MHz, $CDCl_3$) of Phenylmethanol (2o)

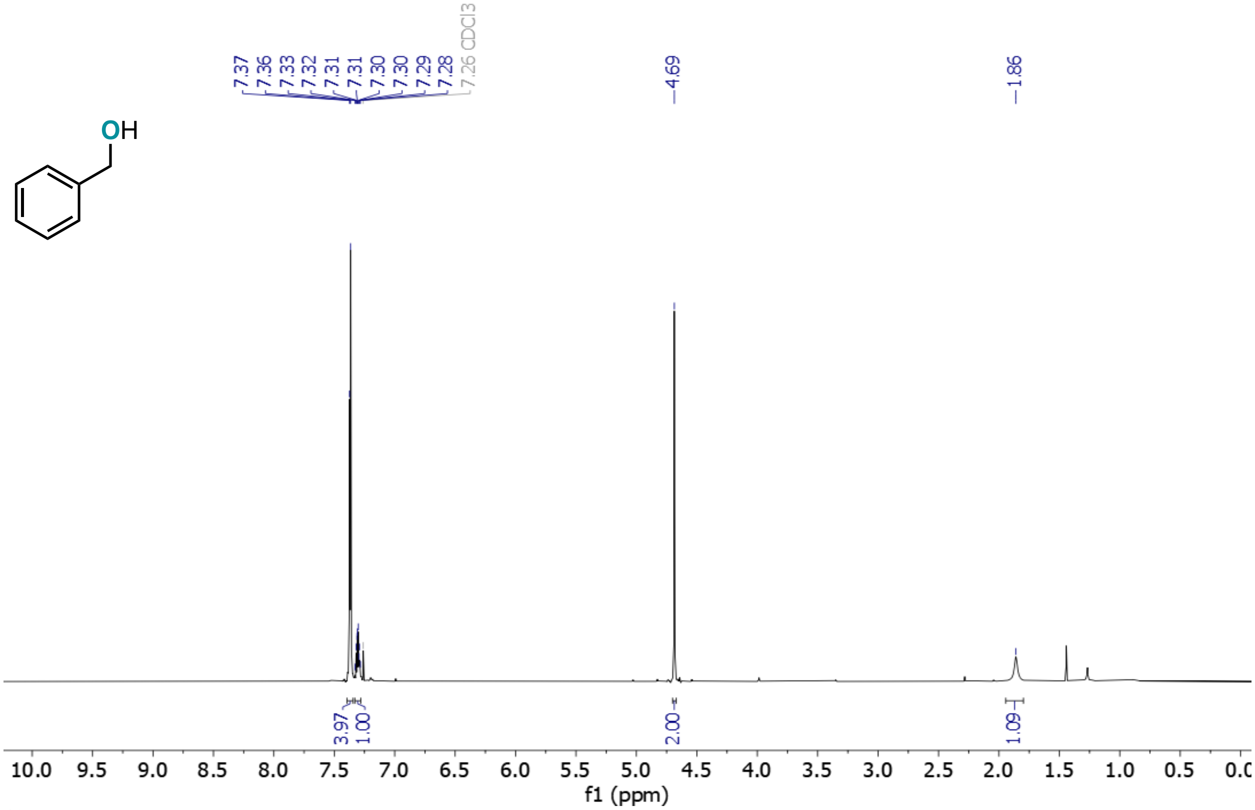

^{13}C NMR (126 MHz, $CDCl_3$) of Phenylmethanol (2o)

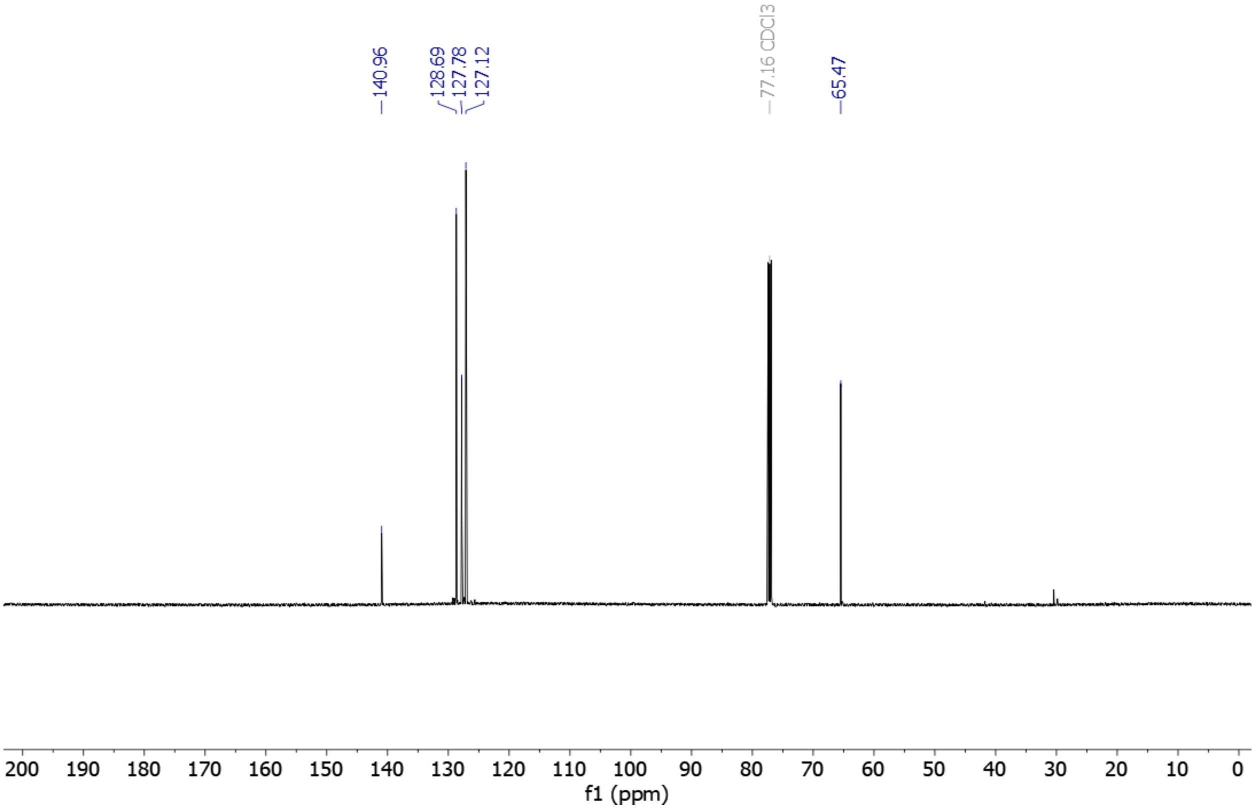

^{1}H NMR (500 MHz, $CDCl_3$) of (4-methoxyphenyl)methanol (2p)

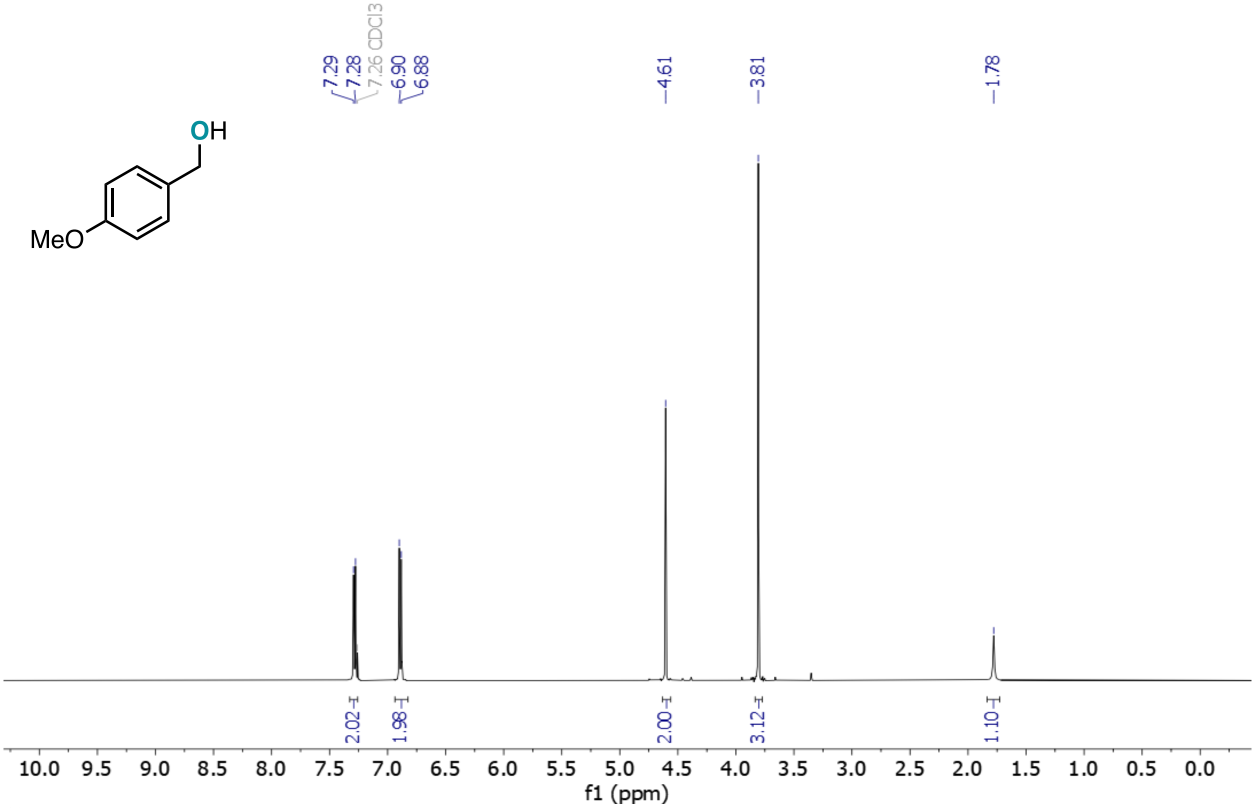

^{13}C NMR (126 MHz, $CDCl_3$) of (4-methoxyphenyl)methanol (2p)

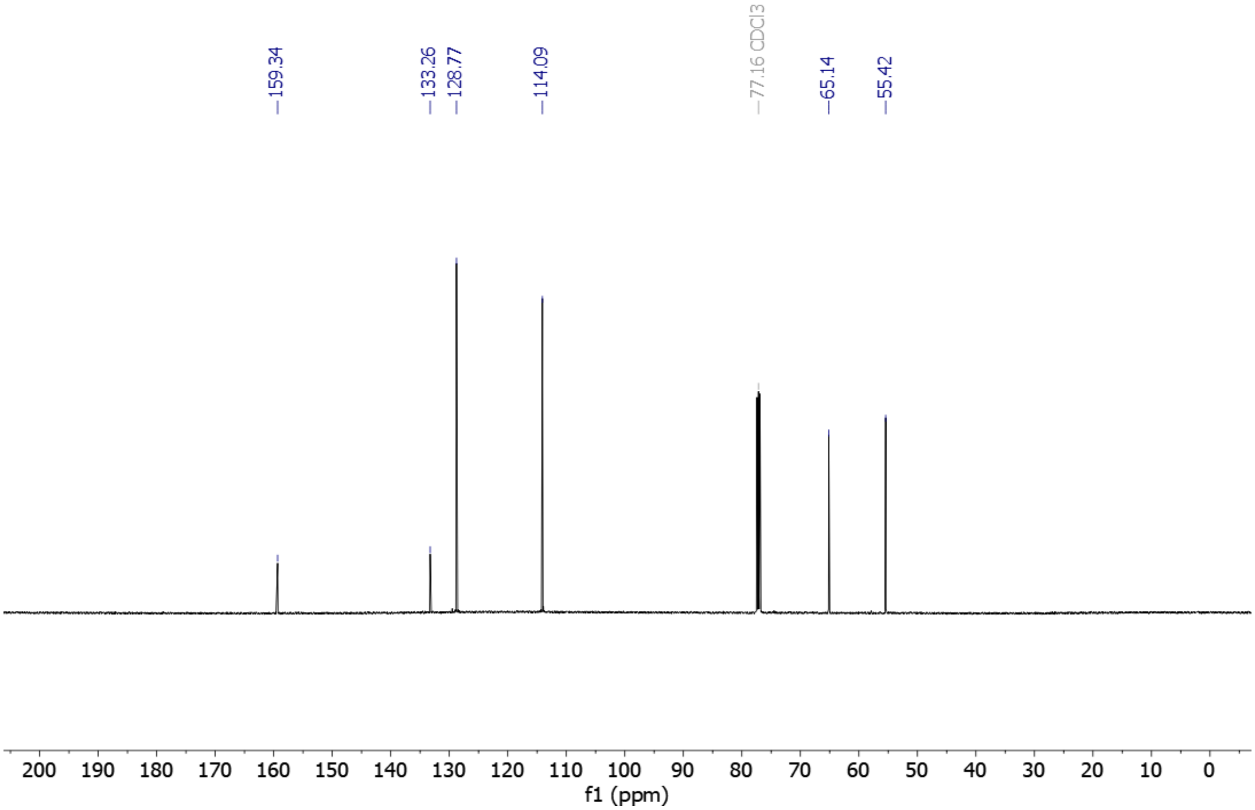

^{1}H NMR (400 MHz, $CDCl_3$) of *m*-tolylmethanol (2q)

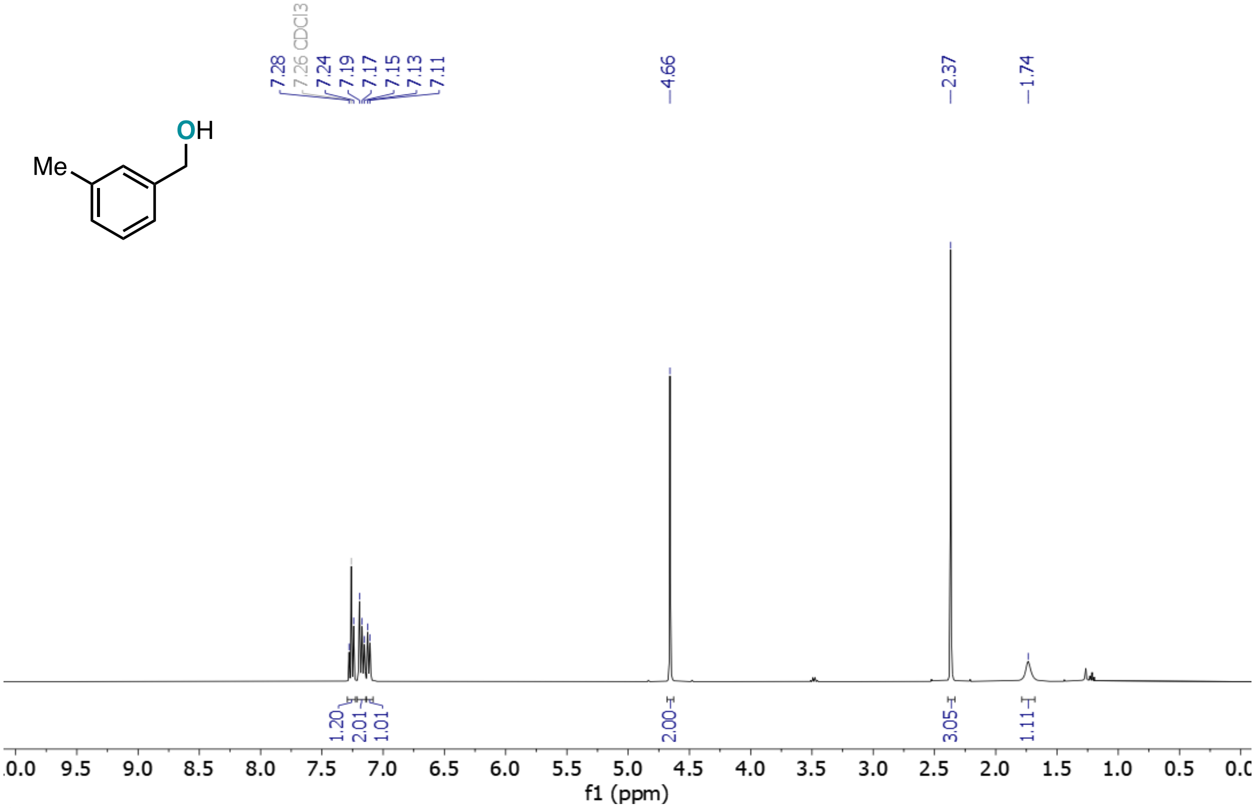

^{13}C NMR (101 MHz, $CDCl_3$) of *m*-tolylmethanol (2q)

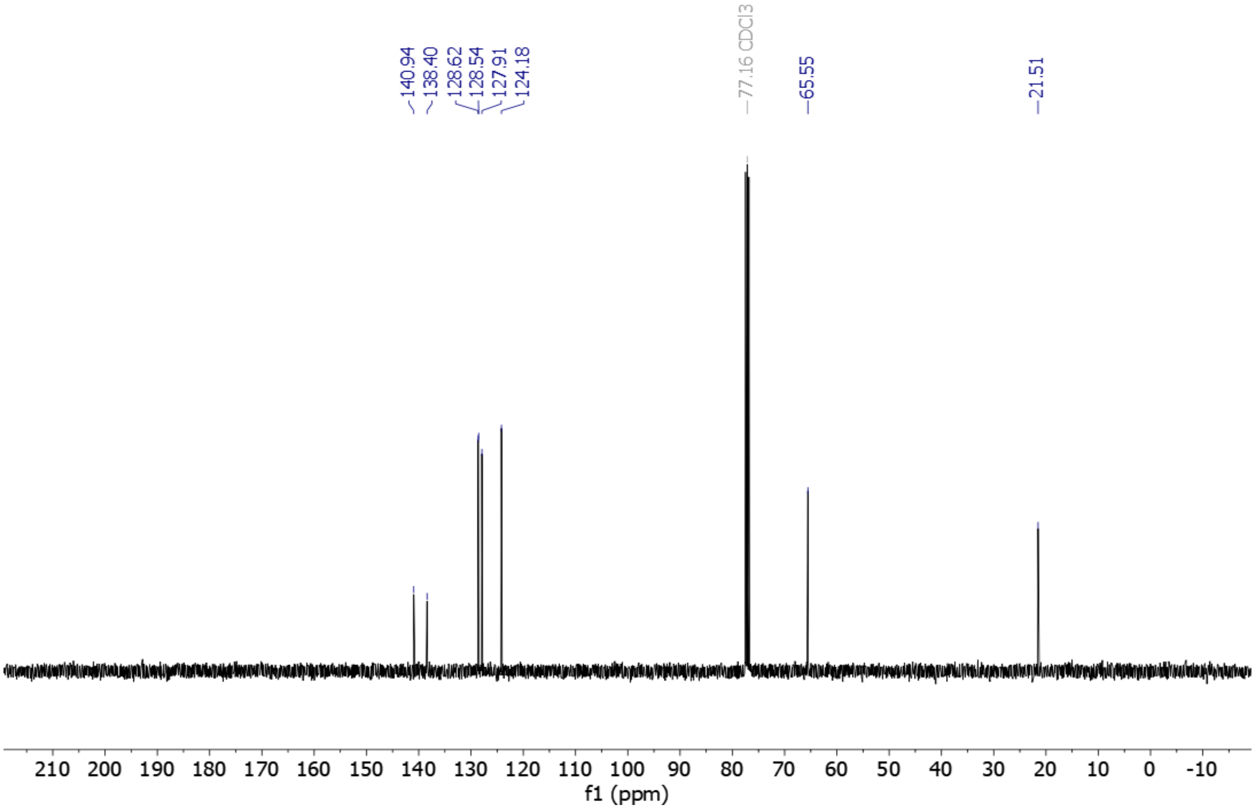

^{1}H NMR (500 MHz, $CDCl_3$) of 1-(3-ethylphenyl)ethan-1-ol (2r)

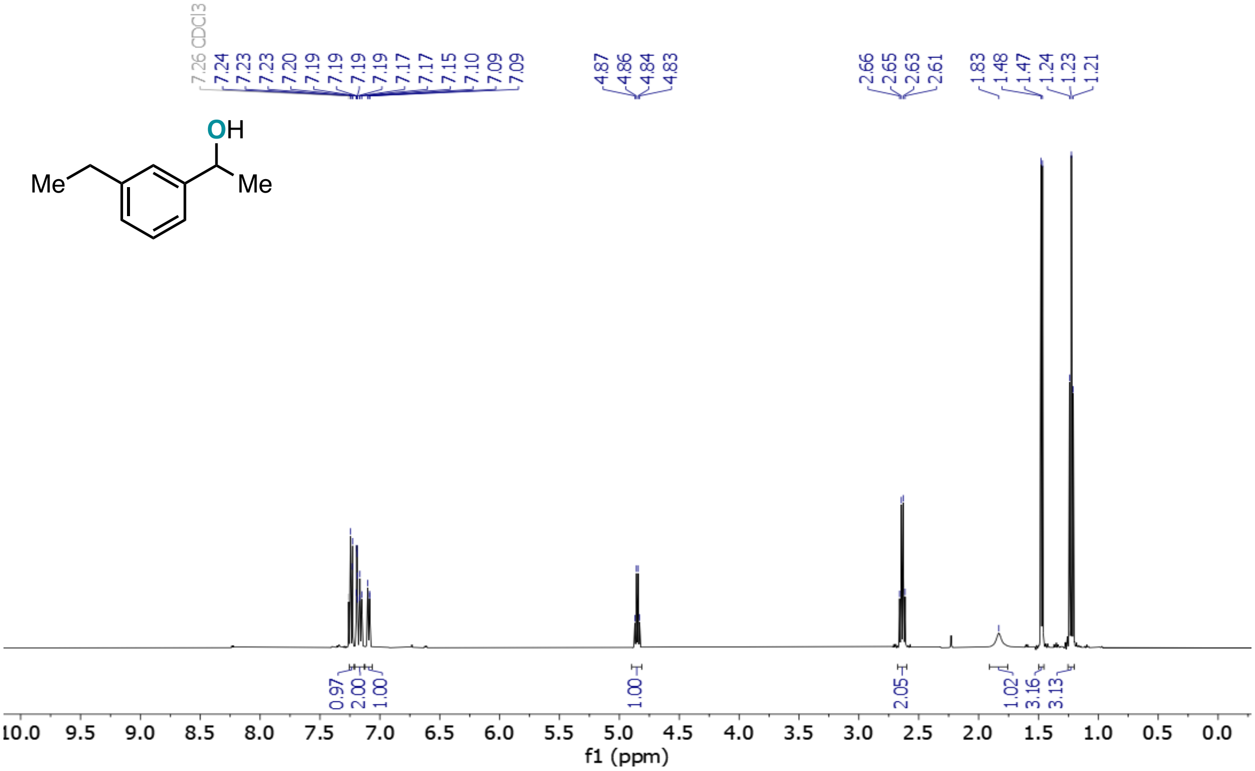

^{13}C NMR (126 MHz, $CDCl_3$) of 1-(3-ethylphenyl)ethan-1-ol (2r)

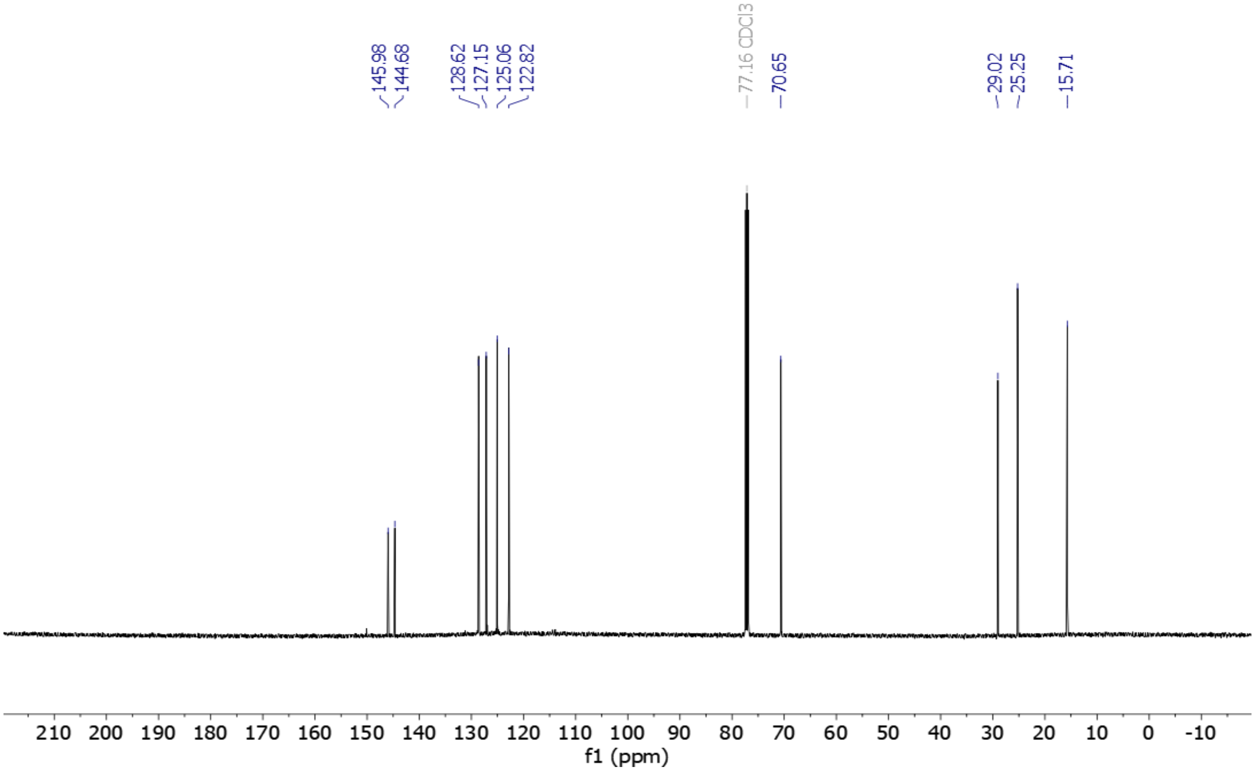

¹H NMR (500 MHz, CDCl₃) of 1-(p-tolyl)ethan-1-ol (2s)

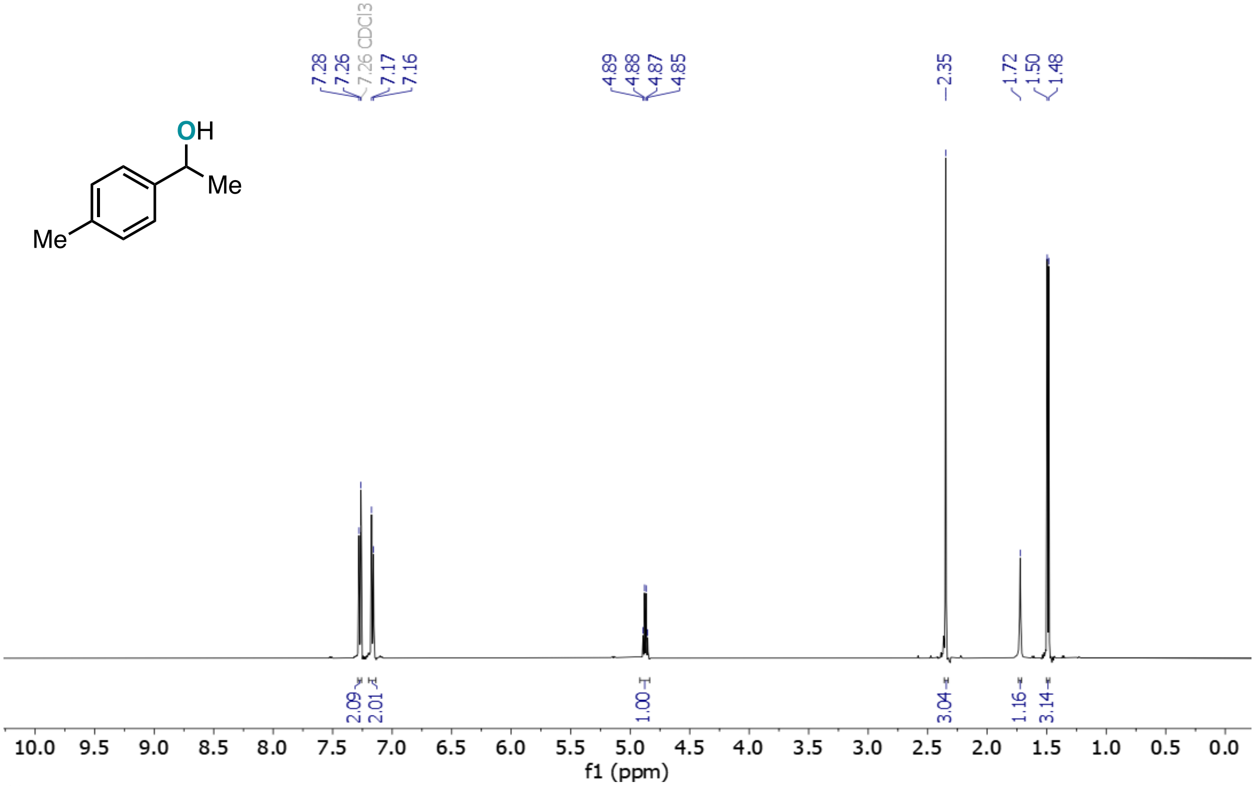

¹³C NMR (126 MHz, CDCl₃) of 1-(p-tolyl)ethan-1-ol (2s)

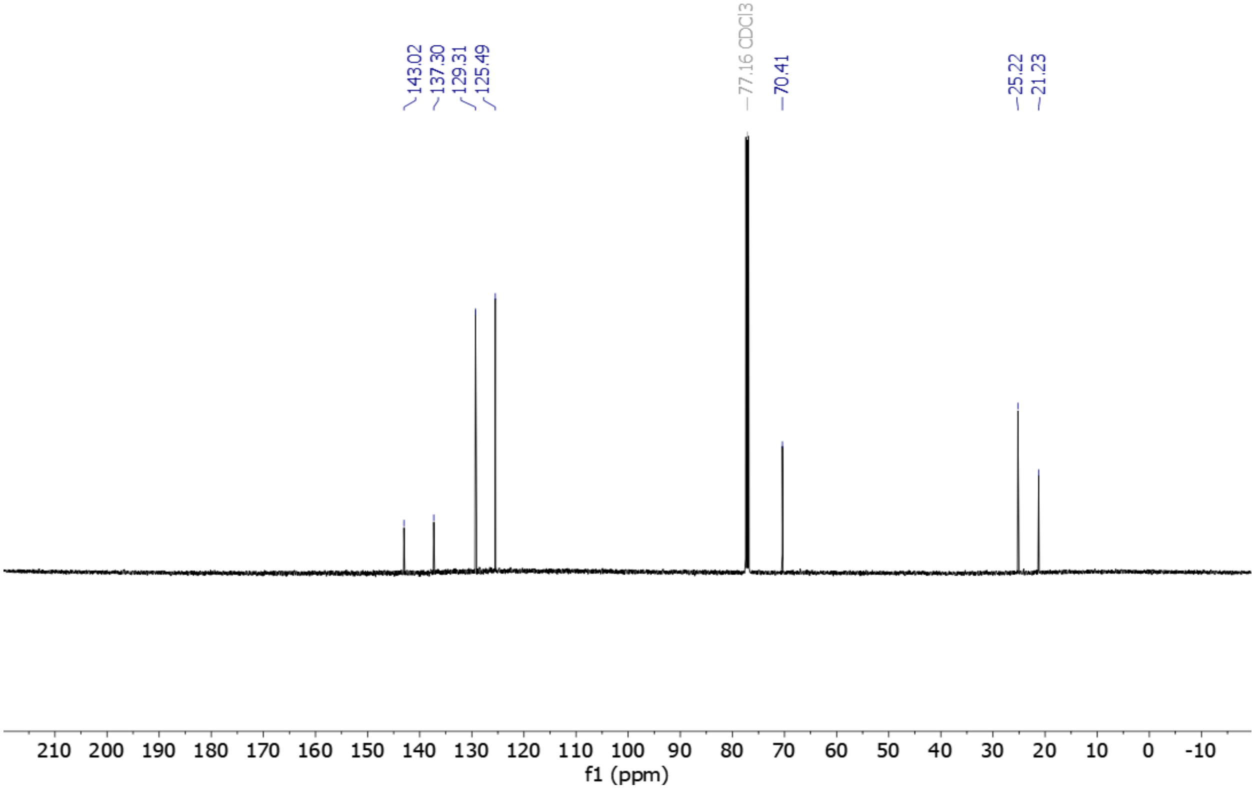

^{1}H NMR (400 MHz, CDCl$_3$) of 1-(4-isopropylphenyl)ethan-1-ol (2t)

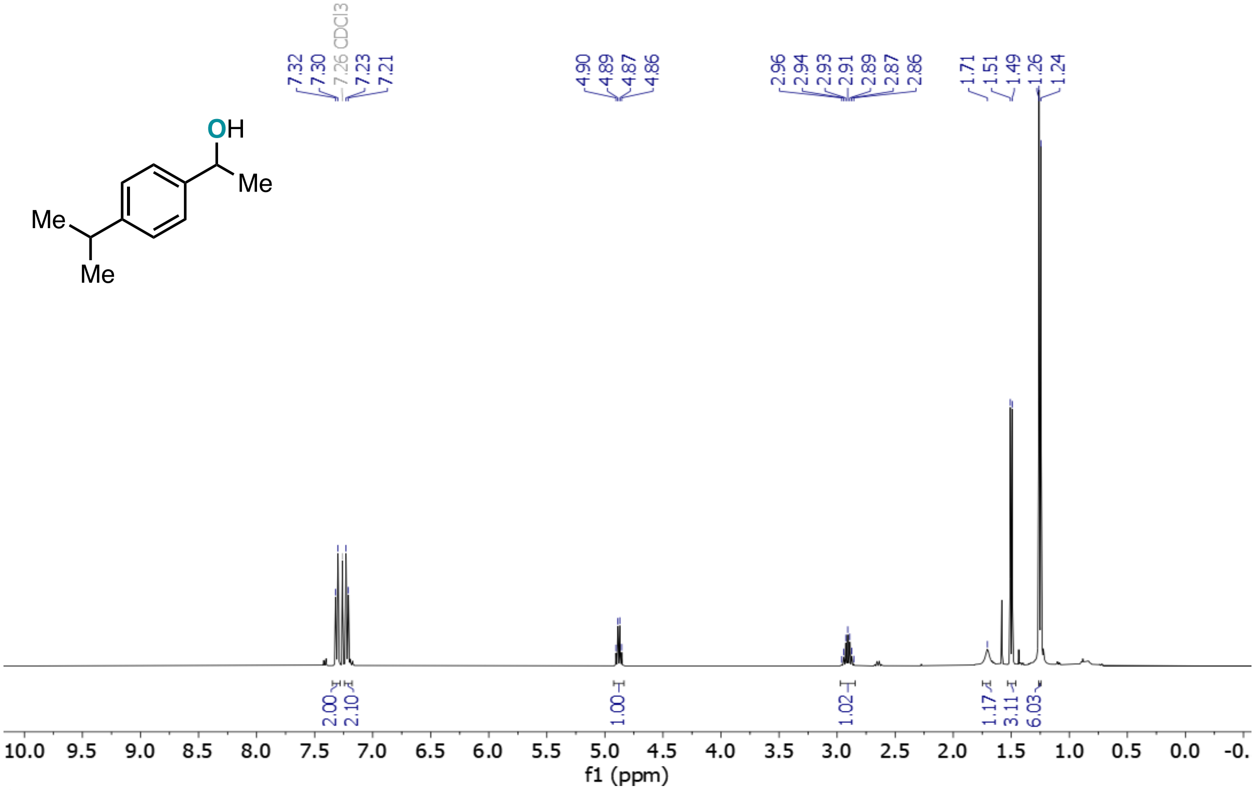

^{13}C NMR (101 MHz, CDCl$_3$) of 1-(4-isopropylphenyl)ethan-1-ol (2t)

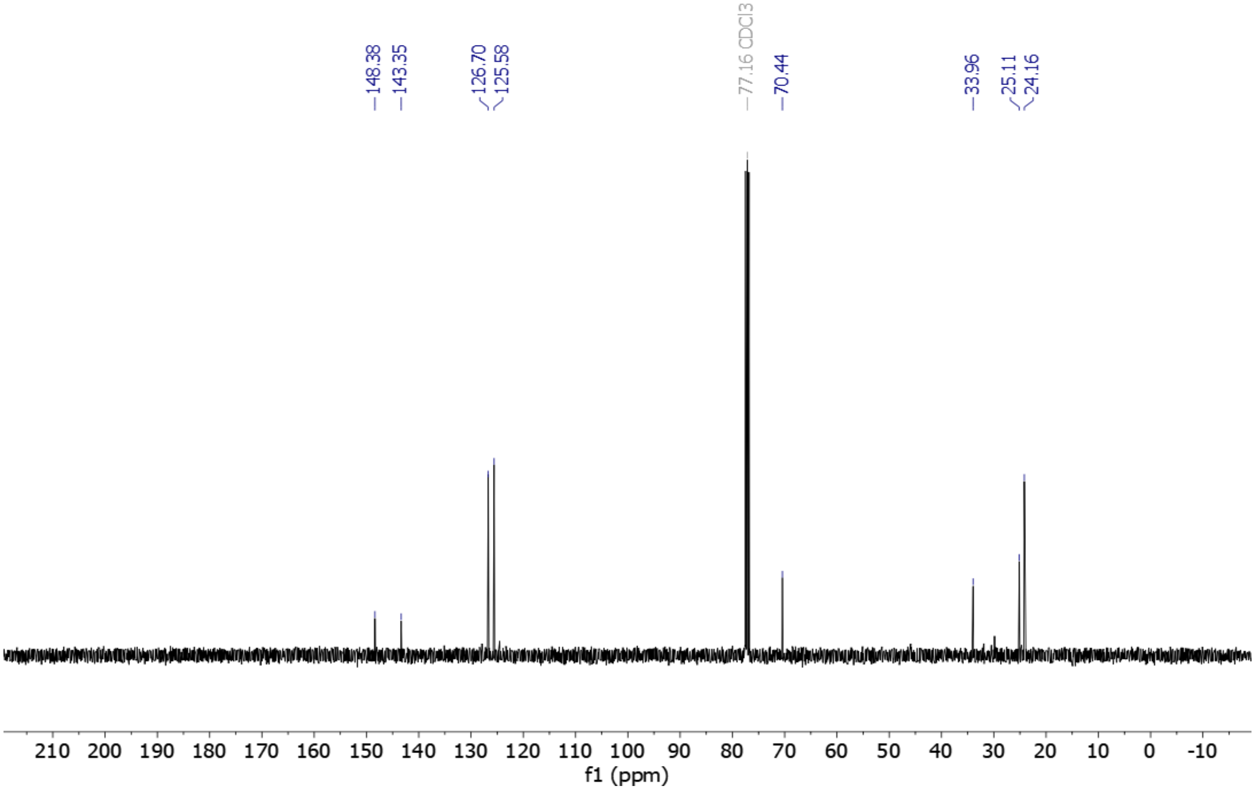

^{1}H NMR (500 MHz, $CDCl_3$) of (4-isopropylphenyl)methanol (2u)

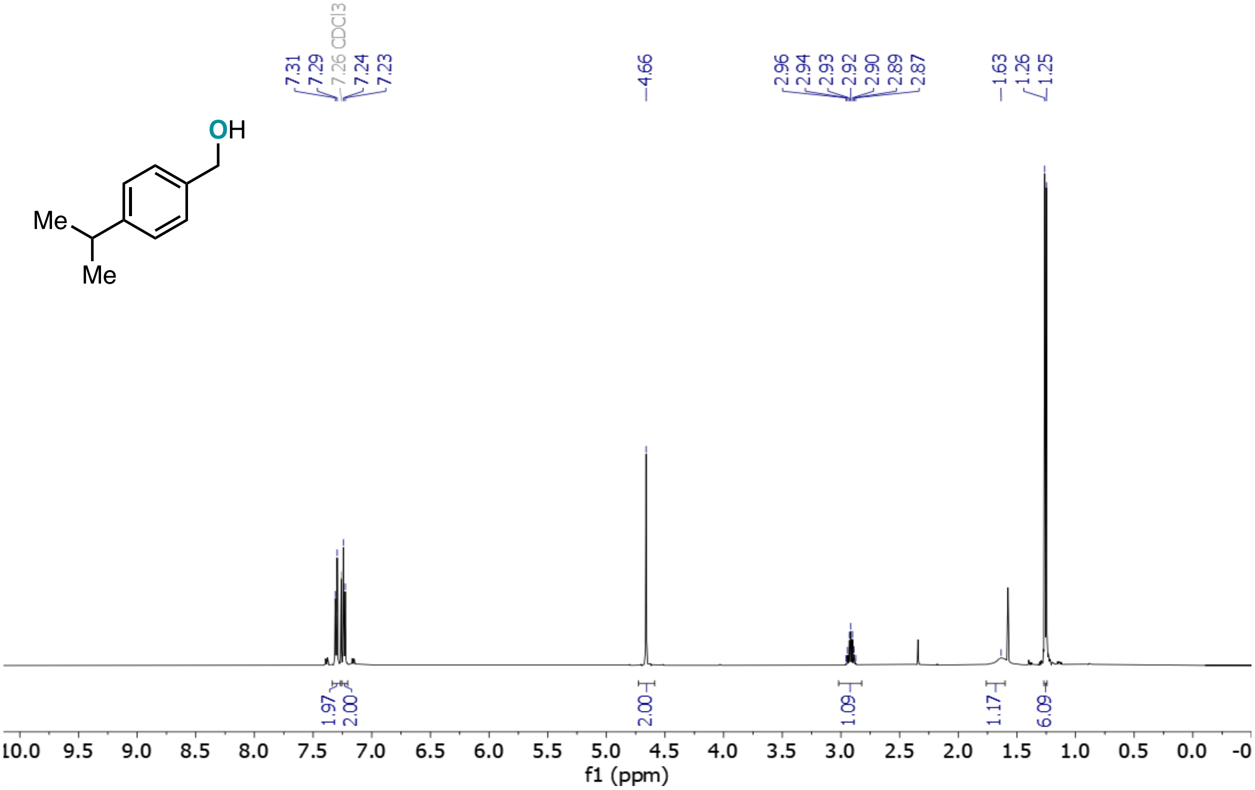

^{13}C NMR (126 MHz, $CDCl_3$) of (4-isopropylphenyl)methanol (2u)

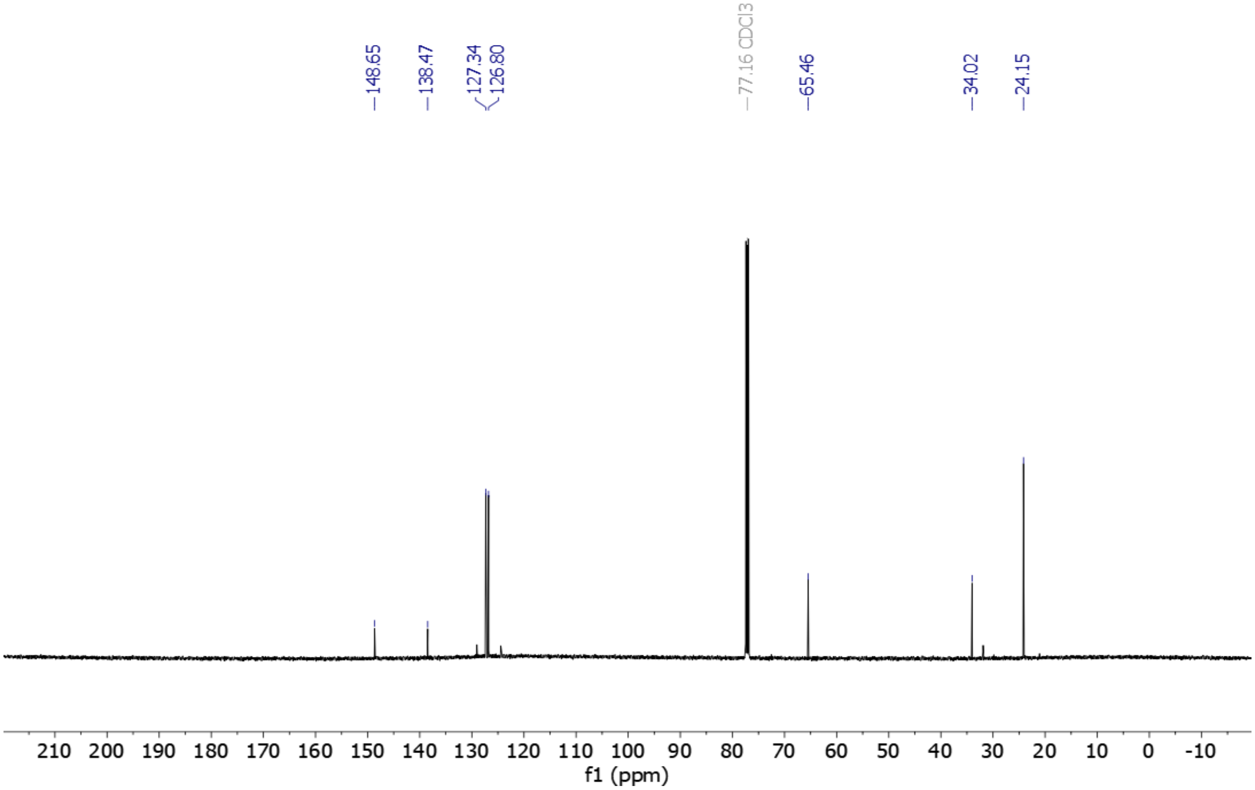

^{1}H NMR (500 MHz, $CDCl_3$) of 1-phenylbutan-1-ol (2v)

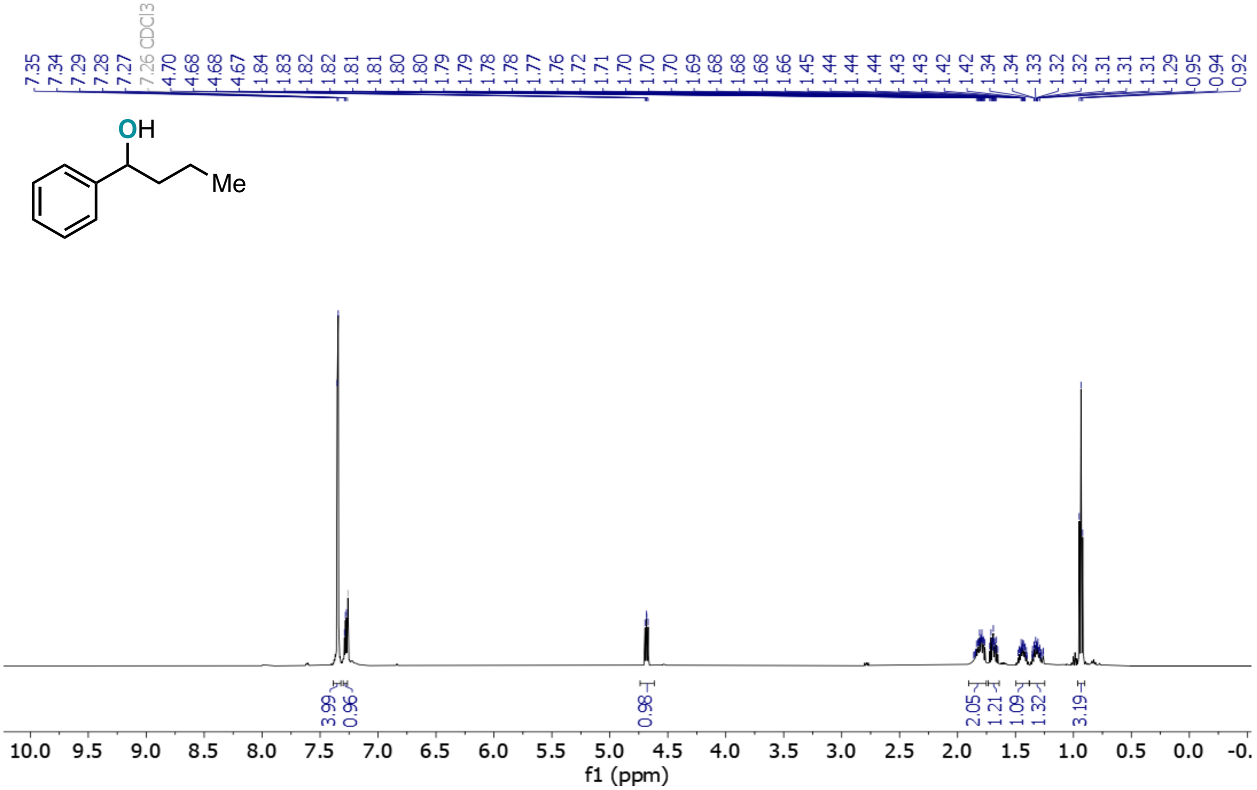

^{13}C NMR (126 MHz, $CDCl_3$) of 1-phenylbutan-1-ol (2v)

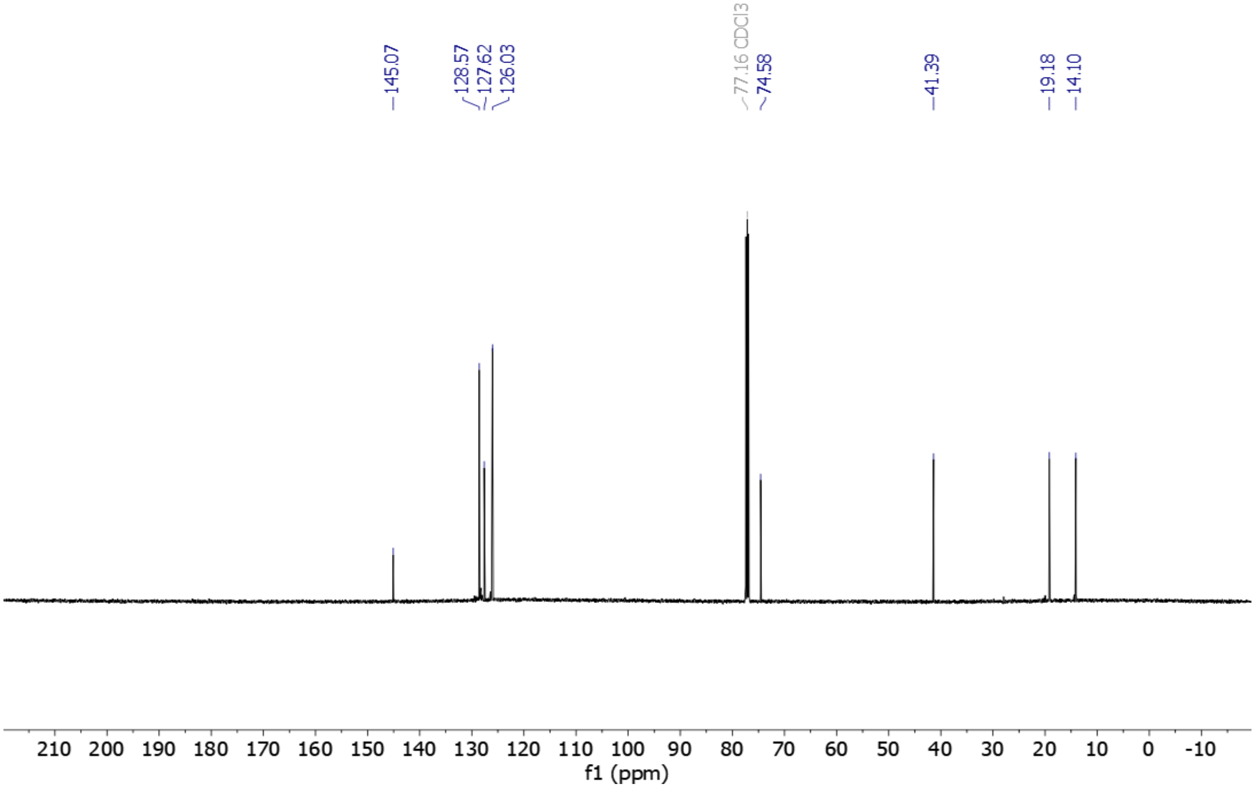

^{1}H NMR (500 MHz, $CDCl_3$) of 1-phenylbutane-1,4-diol (2w)

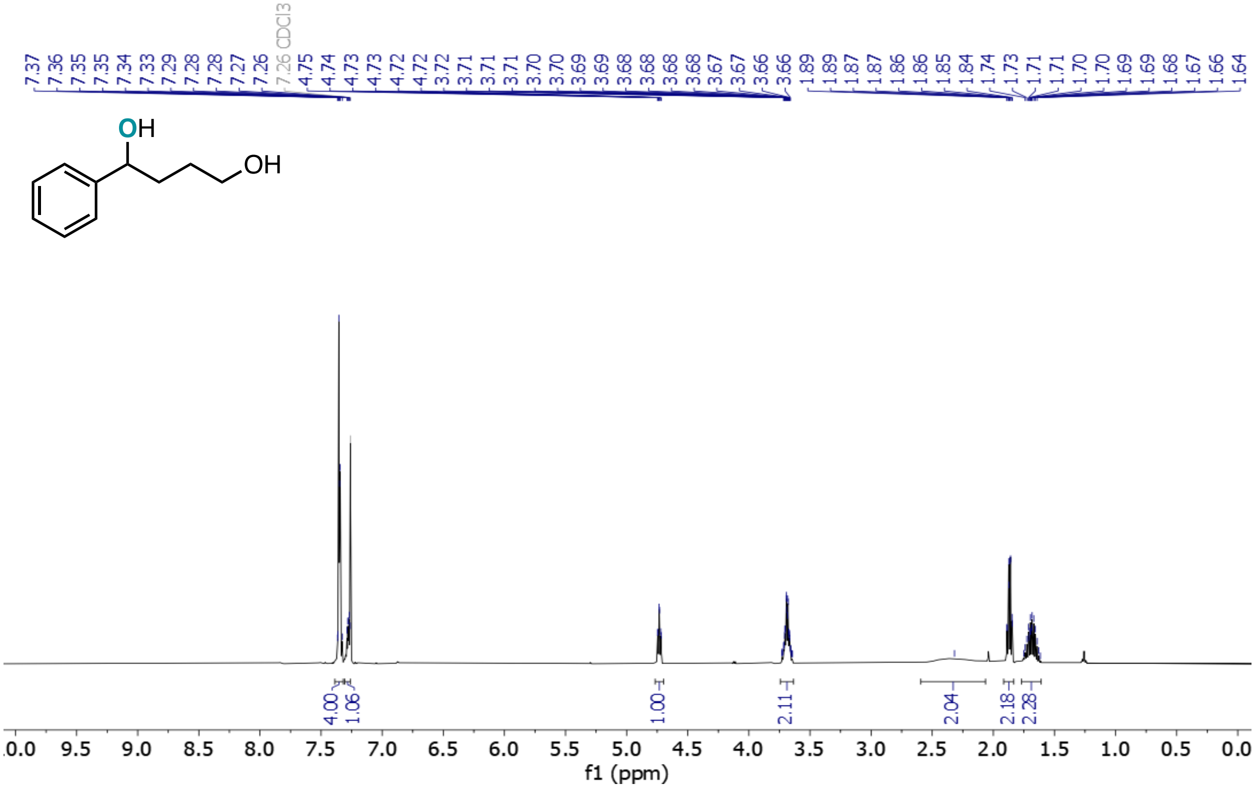

^{13}C NMR (126 MHz, $CDCl_3$) of 1-phenylbutane-1,4-diol (2w)

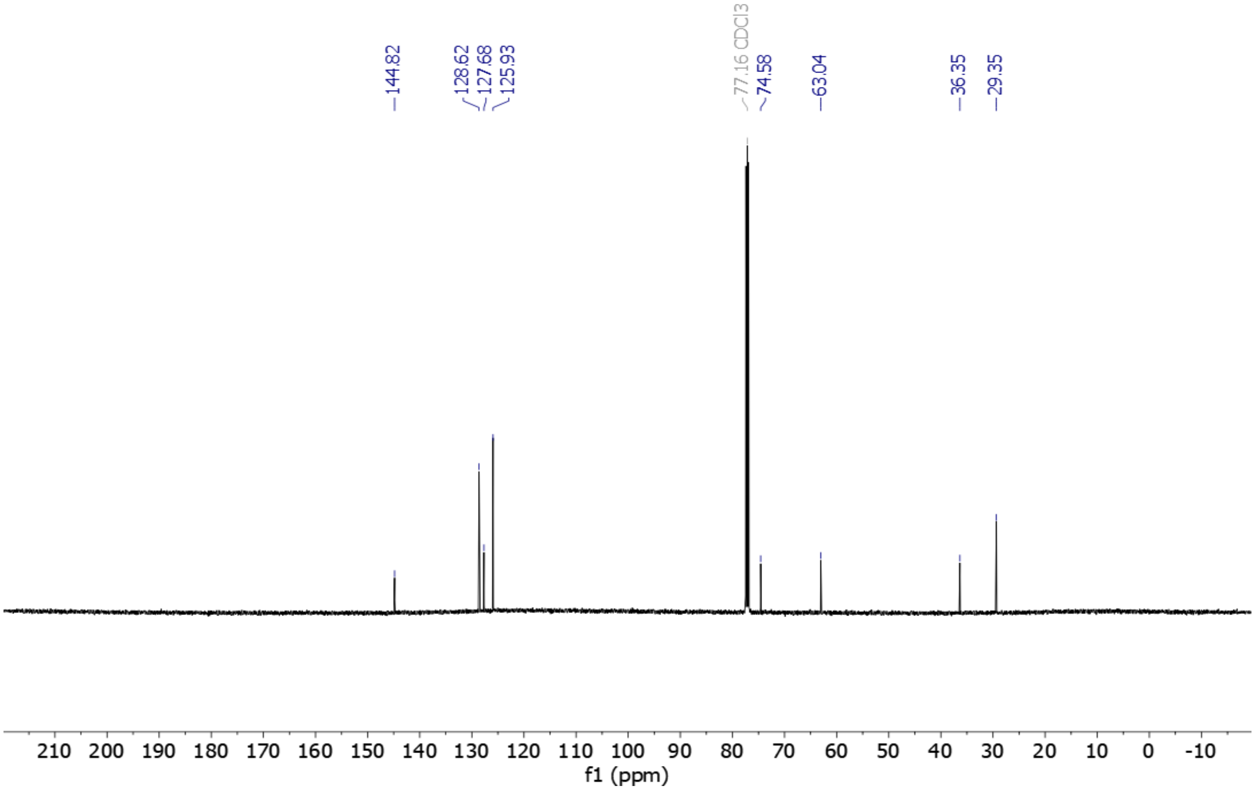

^{1}H NMR (500 MHz, $CDCl_3$) of 5-phenyldihydrofuran-2(3H)-one (2x)

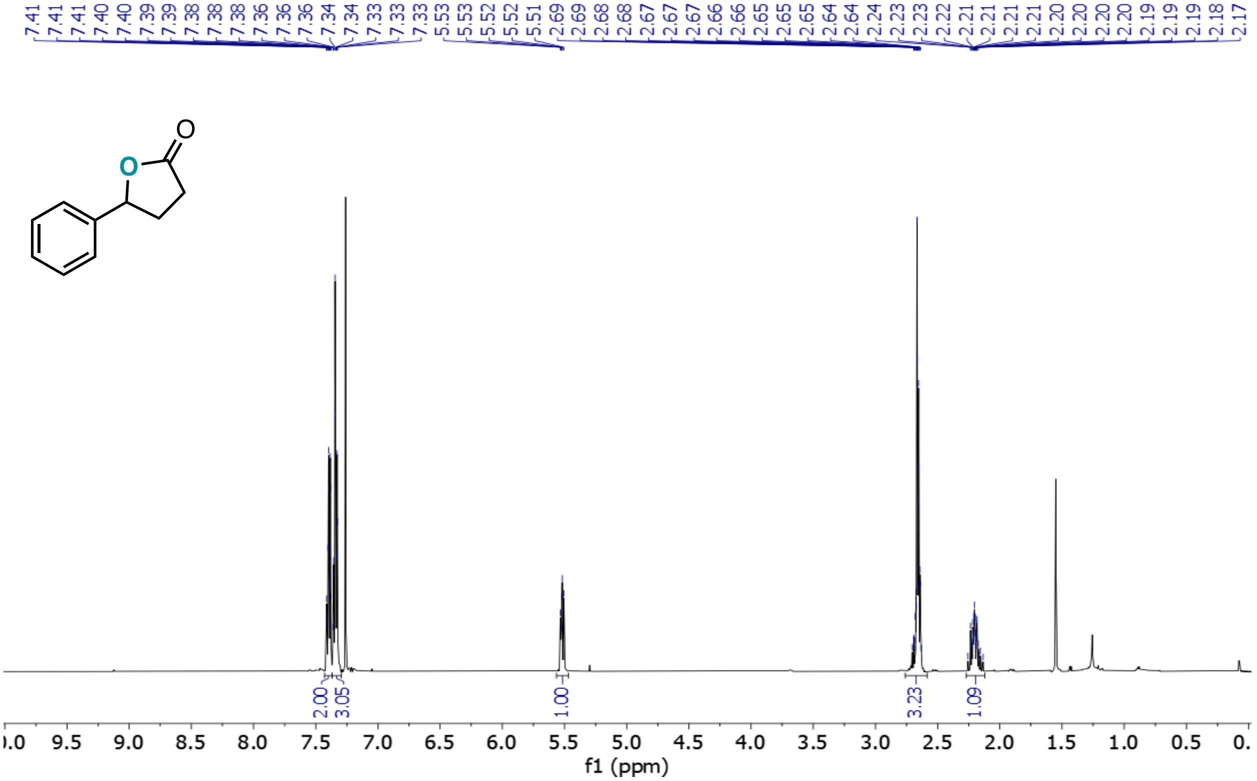

^{13}C NMR (126 MHz, $CDCl_3$) of 5-phenyldihydrofuran-2(3H)-one (2x)

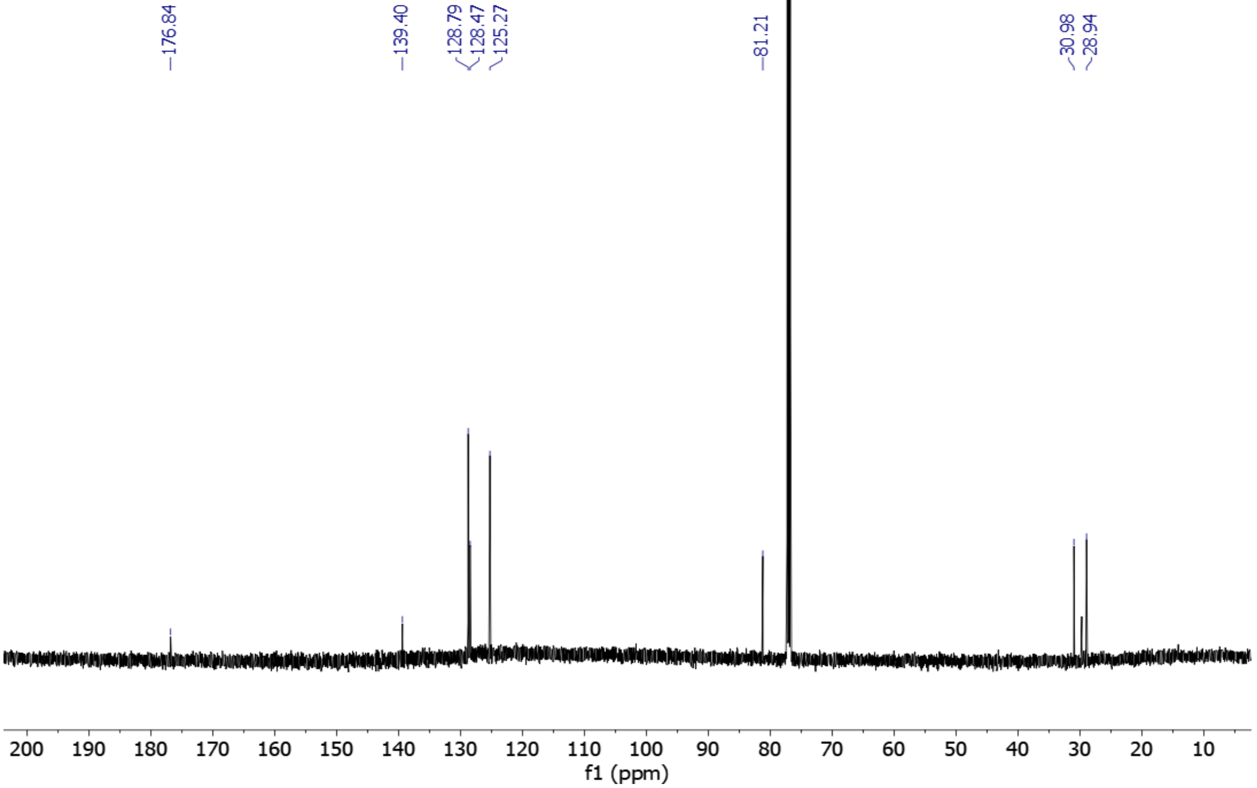

^{1}H NMR (500 MHz, $CDCl_3$) of cyclopropyl(phenyl)methanol (2y)

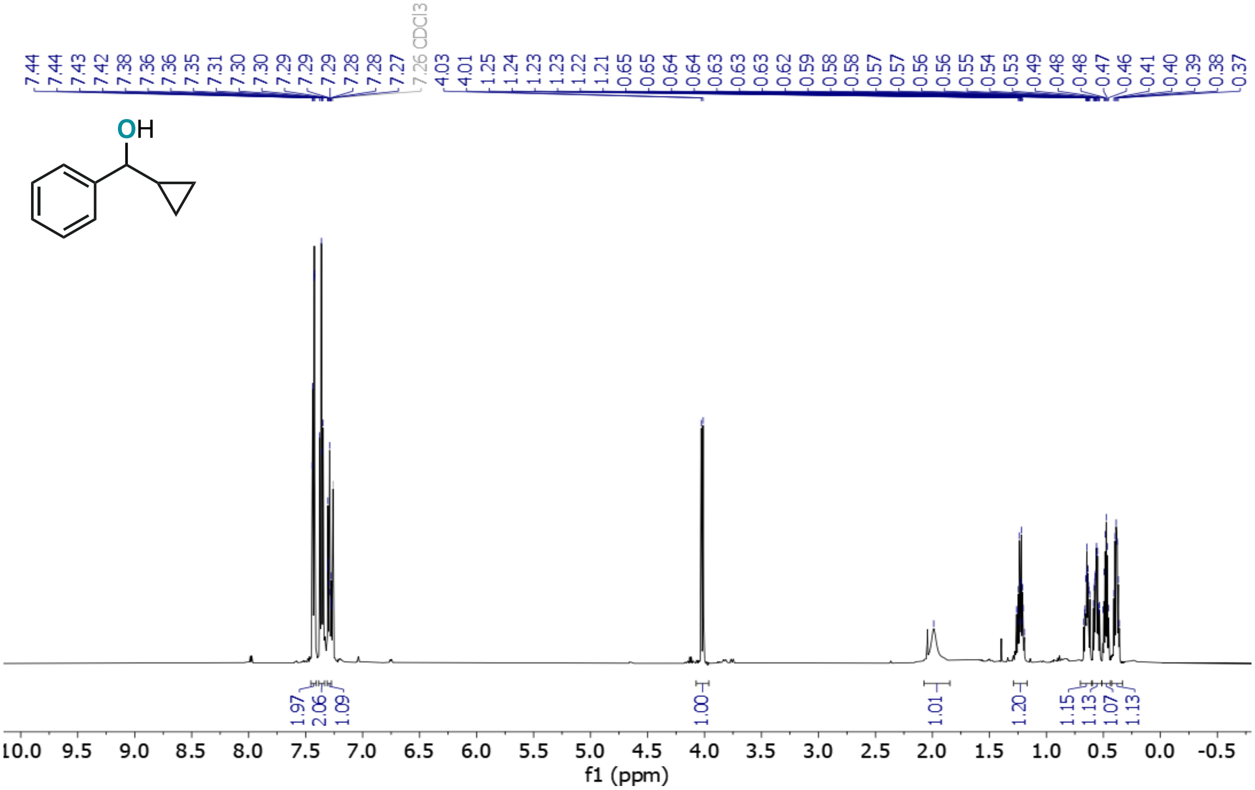

^{13}C NMR (126 MHz, $CDCl_3$) of cyclopropyl(phenyl)methanol (2y)

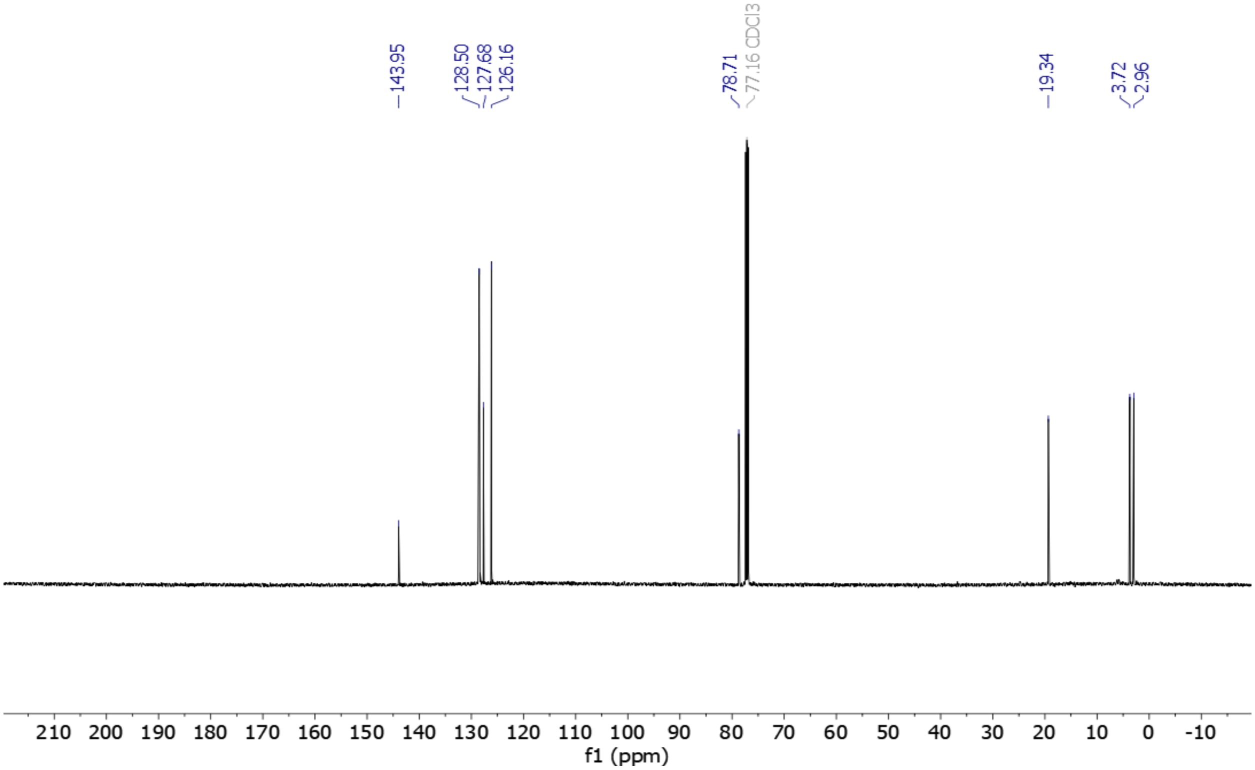

^{1}H NMR (400 MHz, CDCl$_3$) of methyl 2-(4-(1-hydroxy-2-methylpropyl)phenyl)propanoate (2z)

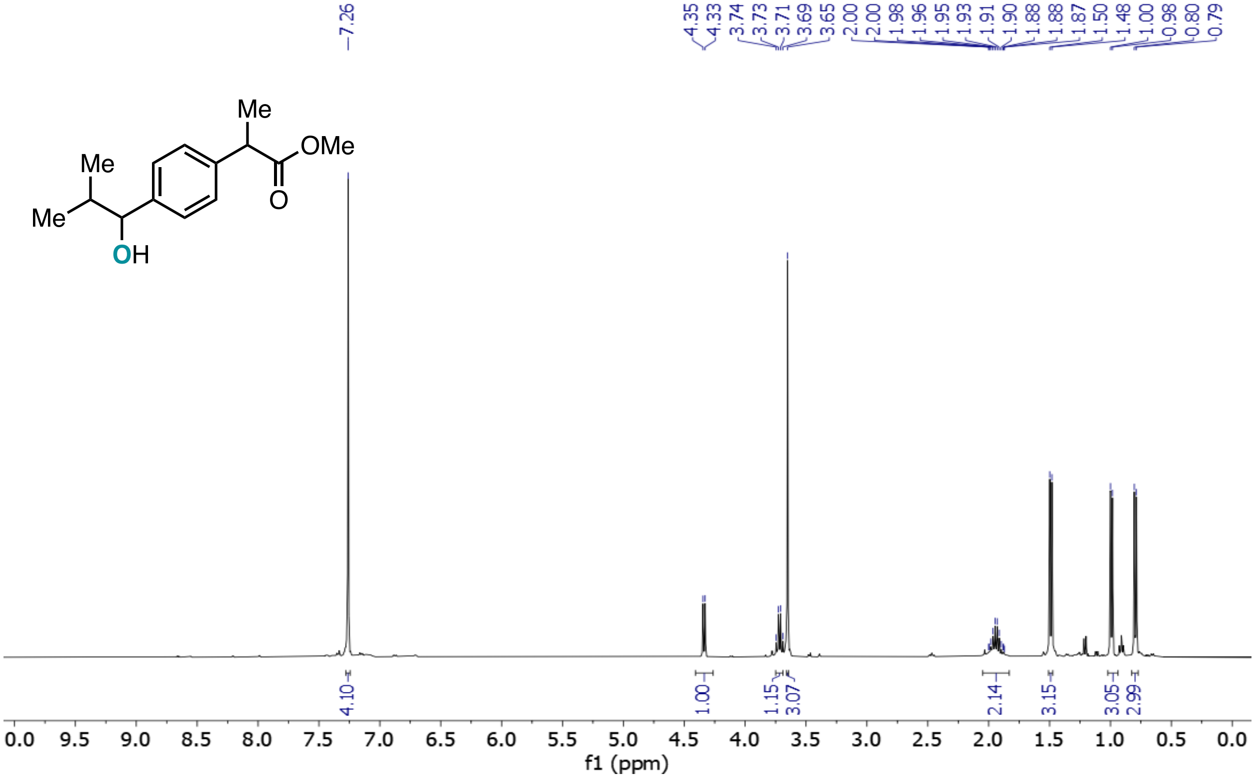

^{13}C NMR (101 MHz, CDCl$_3$) of methyl 2-(4-(1-hydroxy-2-methylpropyl)phenyl)propanoate (2z)

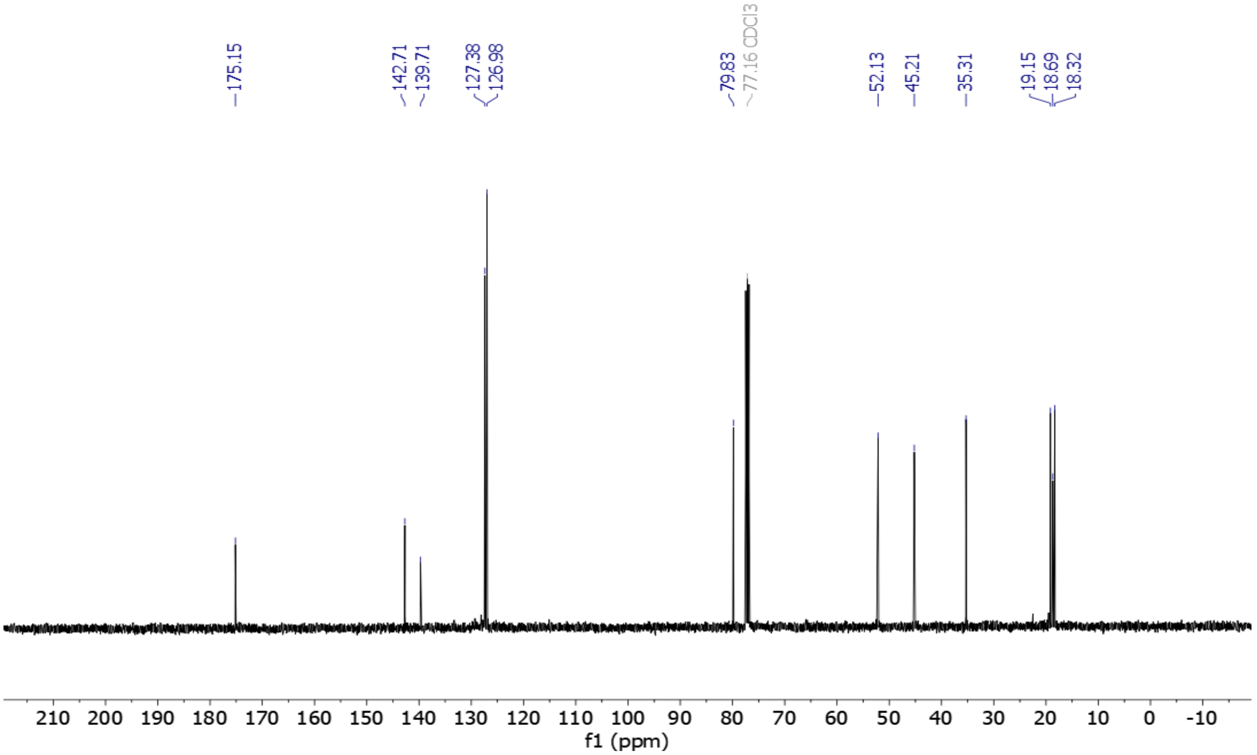

^{1}H NMR (400 MHz, $CDCl_3$) of adamantan-1-ol (2ad)

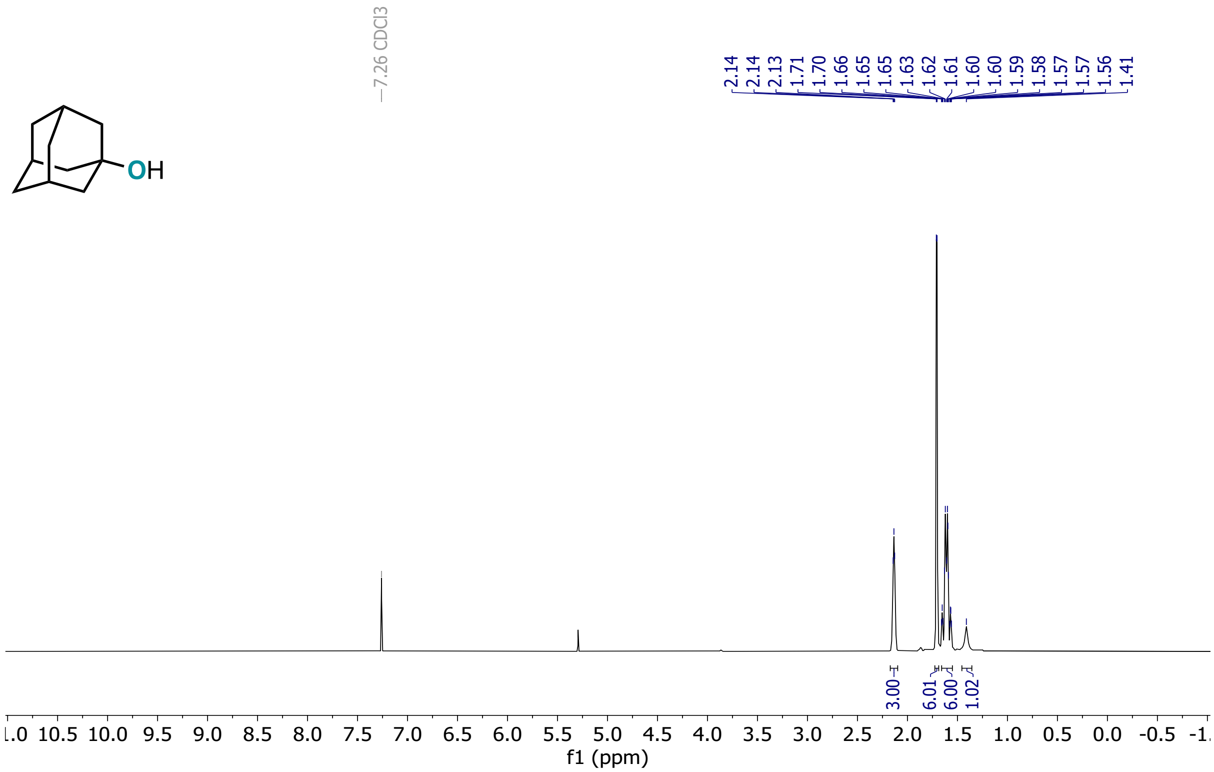

^{13}C NMR (100 MHz, $CDCl_3$) of adamantan-1-ol (2ad)

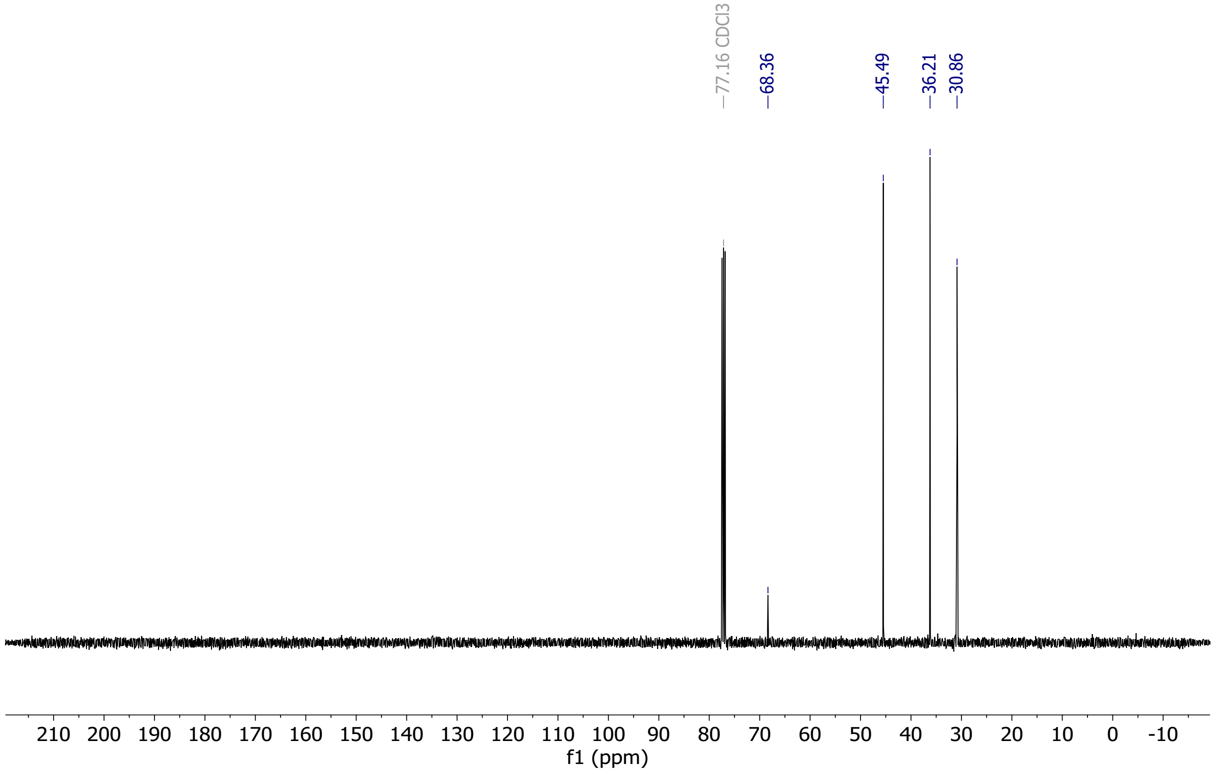

^{1}H NMR (500 MHz, $CDCl_3$) of 3-hydroxyadamantan-1-yl pivalate (2ae)

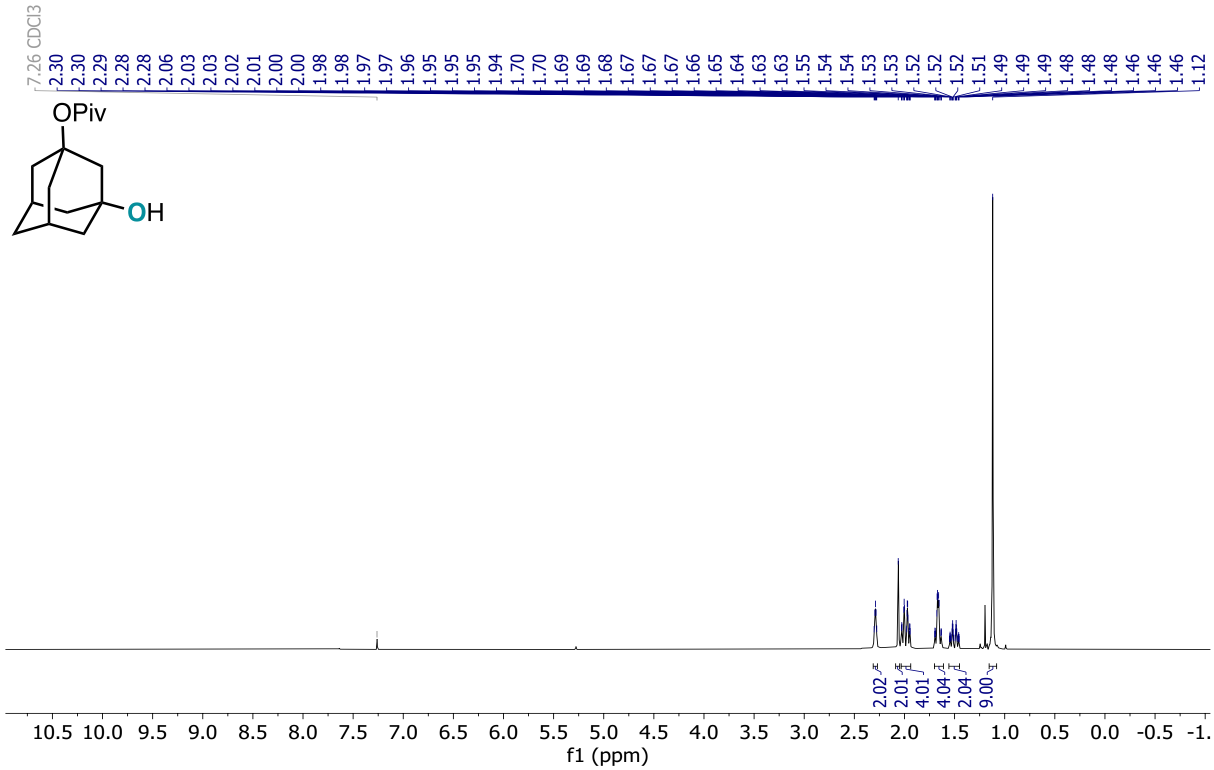

^{13}C NMR (100 MHz, $CDCl_3$) of 3-hydroxyadamantan-1-yl pivalate (2ae)

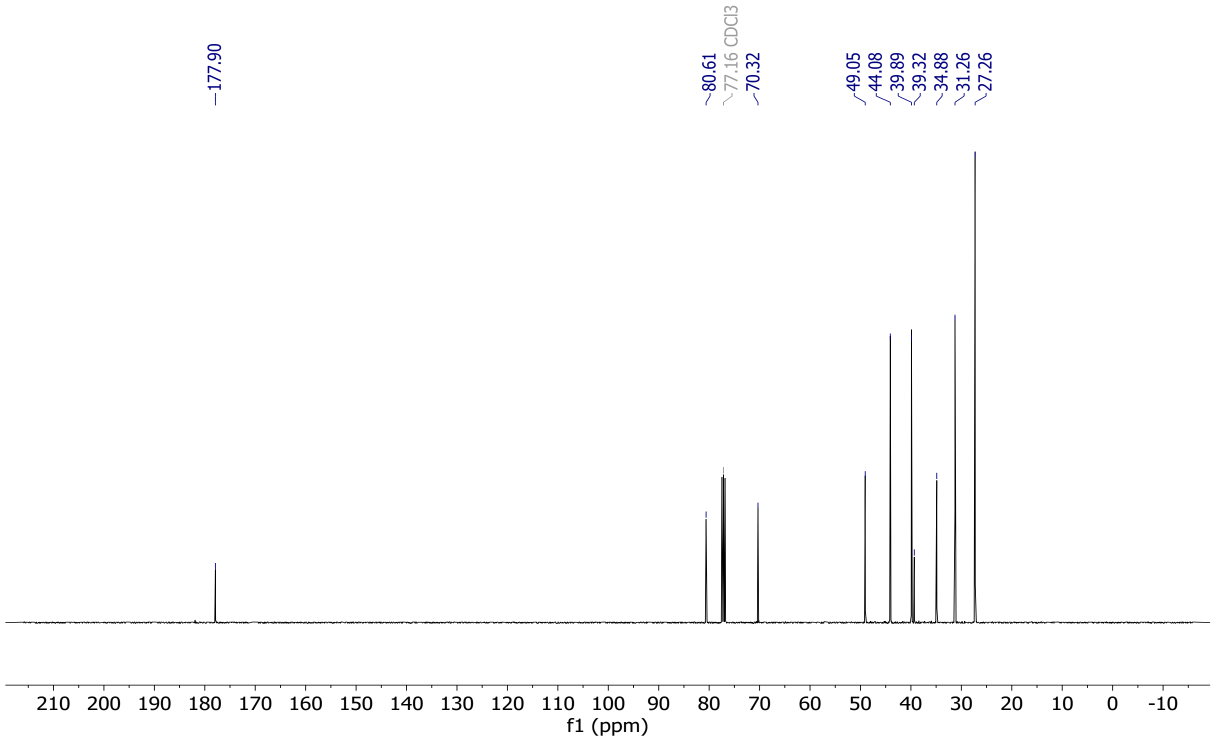

^{1}H NMR (400 MHz, CDCl$_3$) of 5-hydroxy-5-methylhexan-2-yl pivalate (2af)

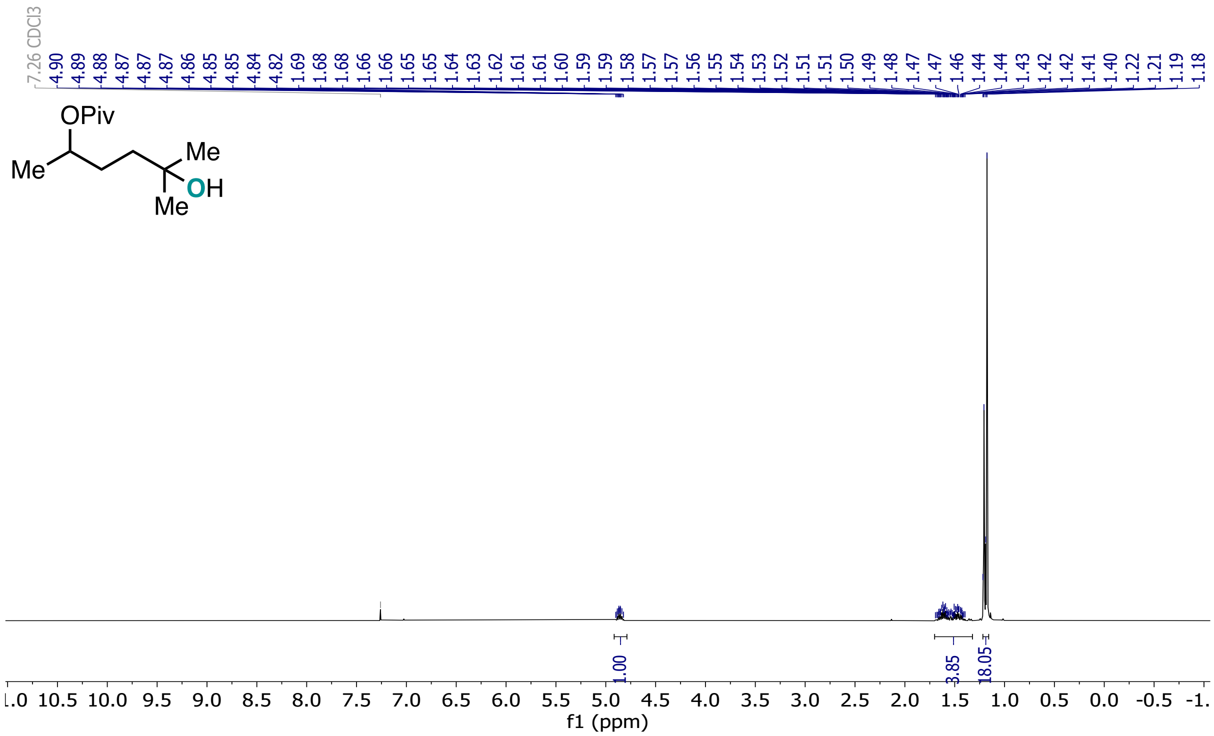

^{13}C NMR (100 MHz, CDCl$_3$) of 5-hydroxy-5-methylhexan-2-yl pivalate (2af)

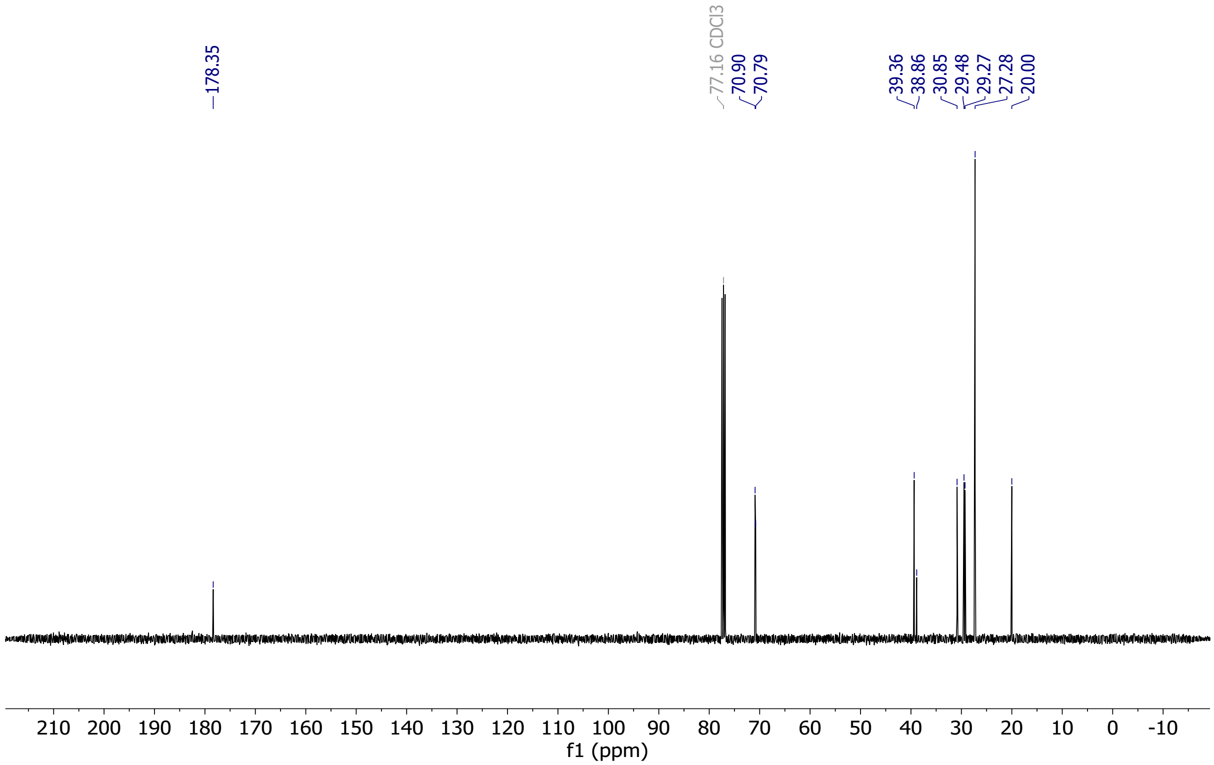

^{1}H NMR (500 MHz, $CDCl_3$) of 4-hydroxy-4-methylpentyl pivalate (2ag)

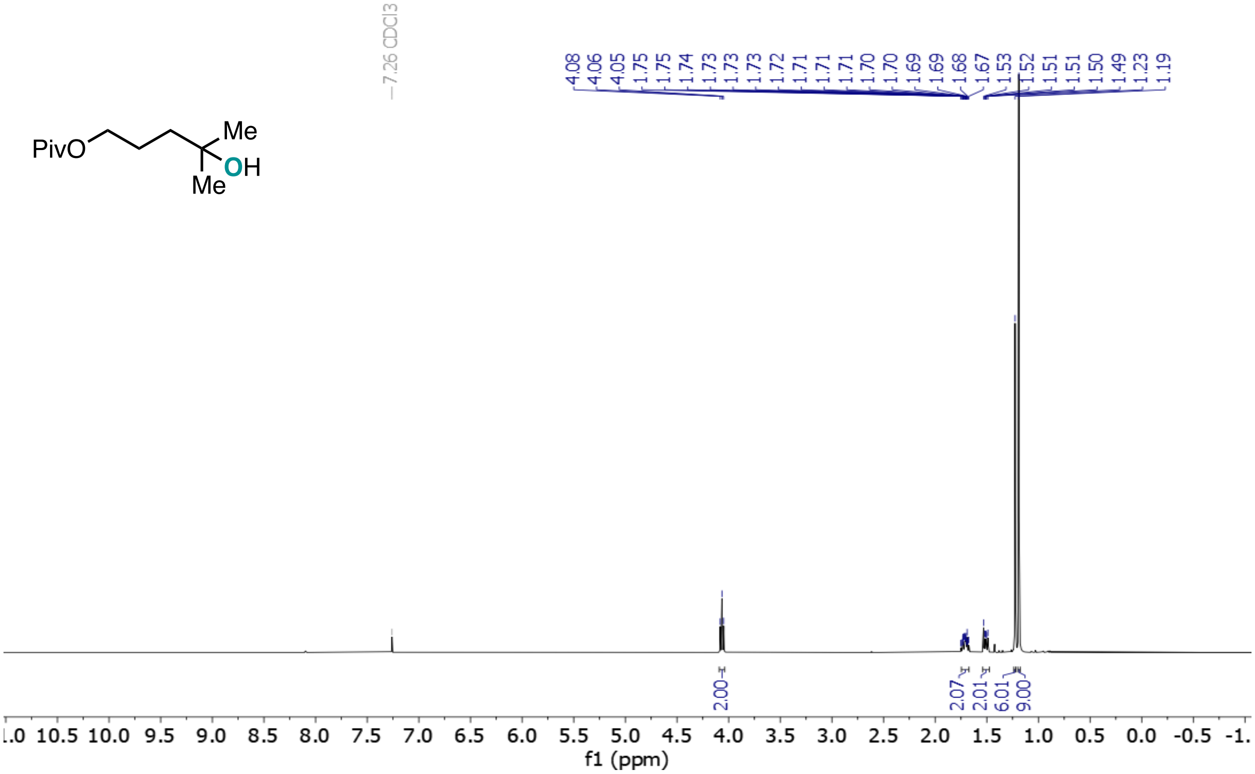

^{13}C NMR (126 MHz, $CDCl_3$) of 4-hydroxy-4-methylpentyl pivalate (2ag)

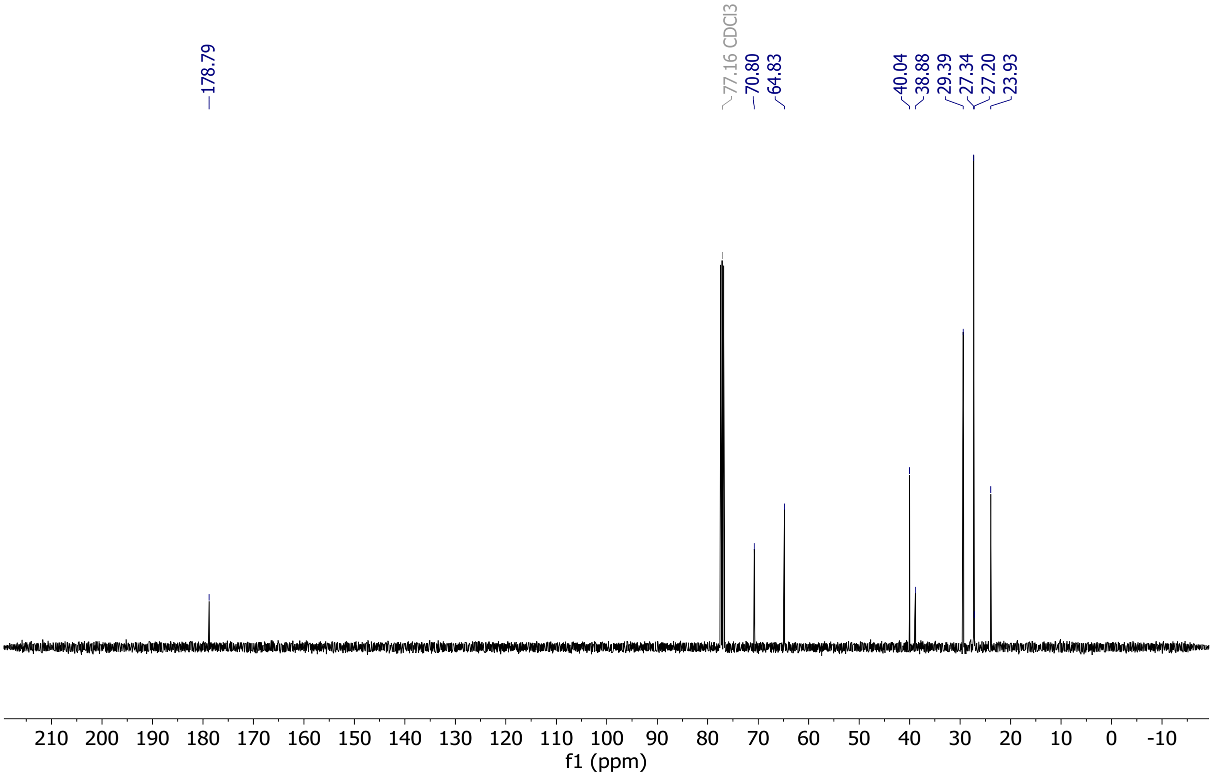

^{1}H NMR (400 MHz, $CDCl_3$) of 5-hydroxy-5-methylhexyl pivalate (2ah)

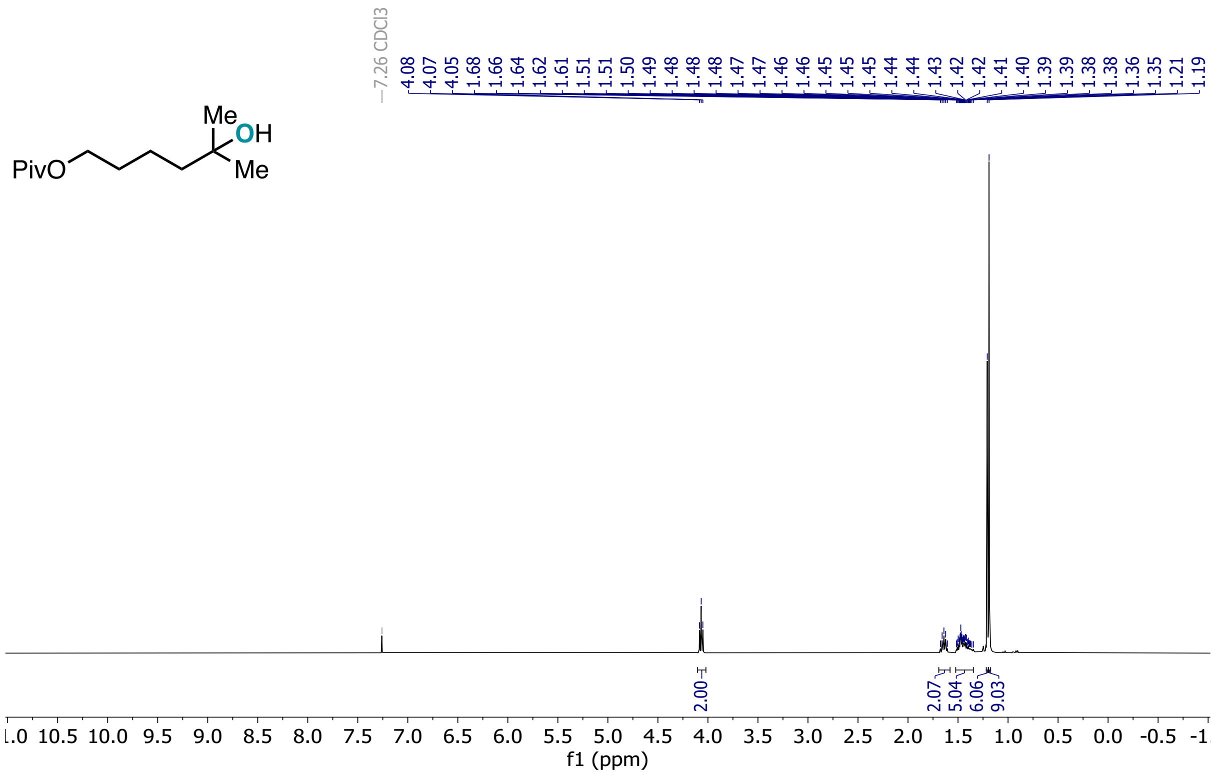

^{13}C NMR (100 MHz, $CDCl_3$) of 5-hydroxy-5-methylhexyl pivalate (2ah)

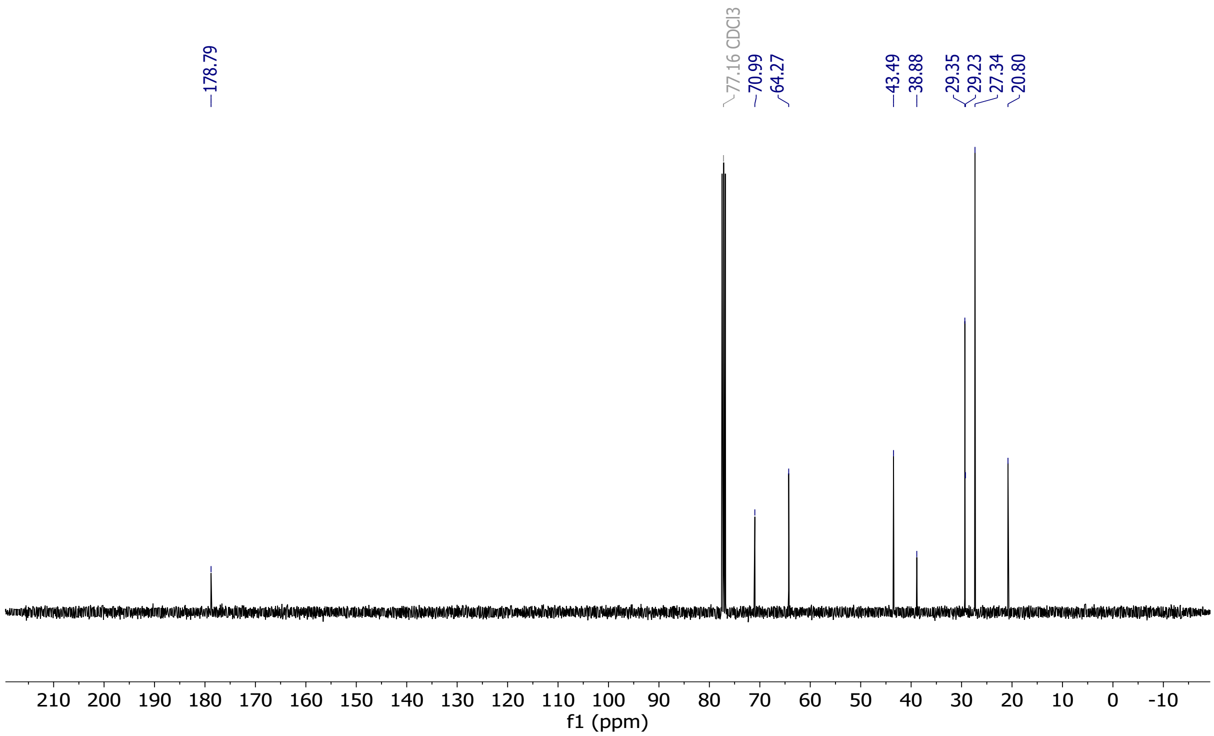

^{1}H NMR (400 MHz, CDCl$_3$) of (1s,4s)-4-hydroxy-4-methylcyclohexyl pivalate (2ai)

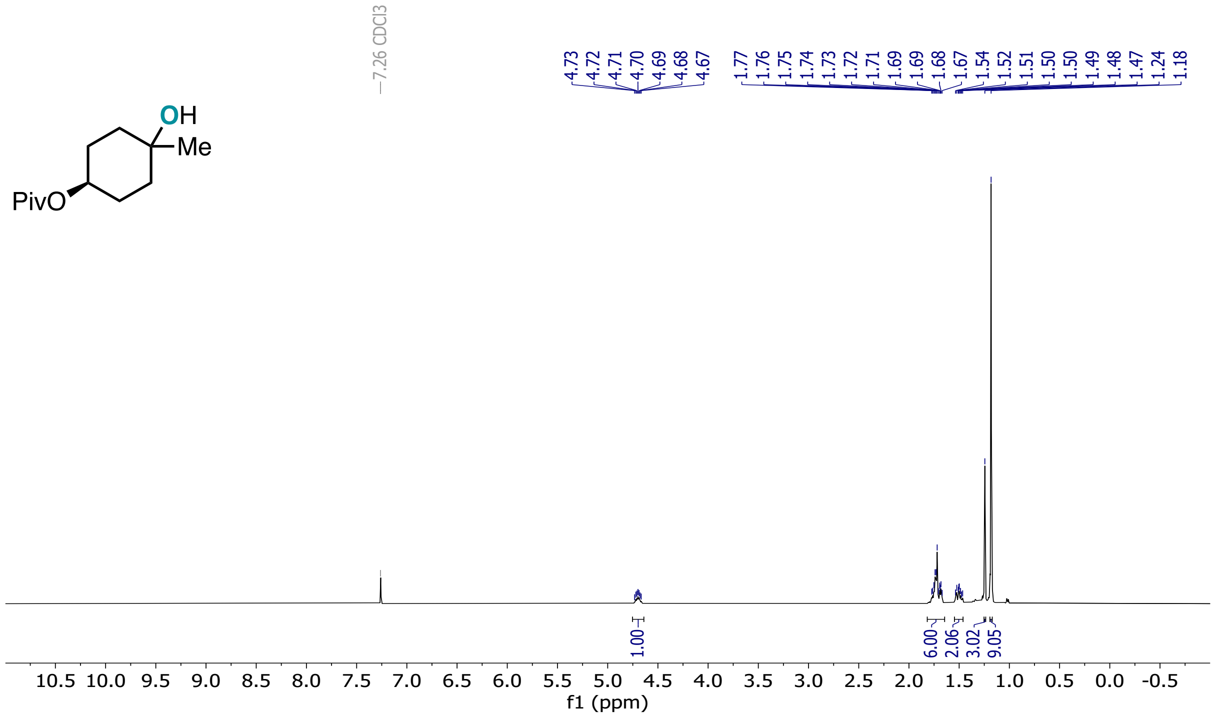

^{13}C NMR (100 MHz, CDCl$_3$) of (1*s*,4*s*)-4-hydroxy-4-methylcyclohexyl pivalate (2ai)

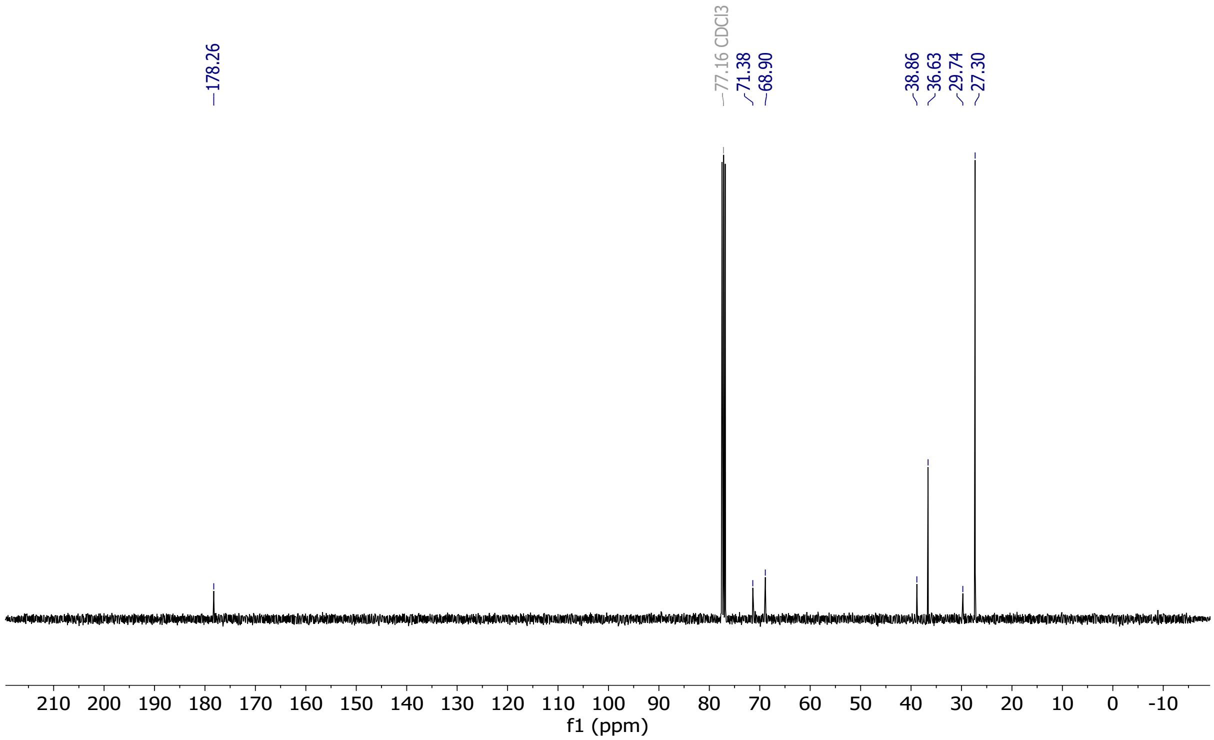

^{1}H NMR (400 MHz, CDCl$_3$) of 5-hydroxy-5-methylhexanenitrile (2aj)

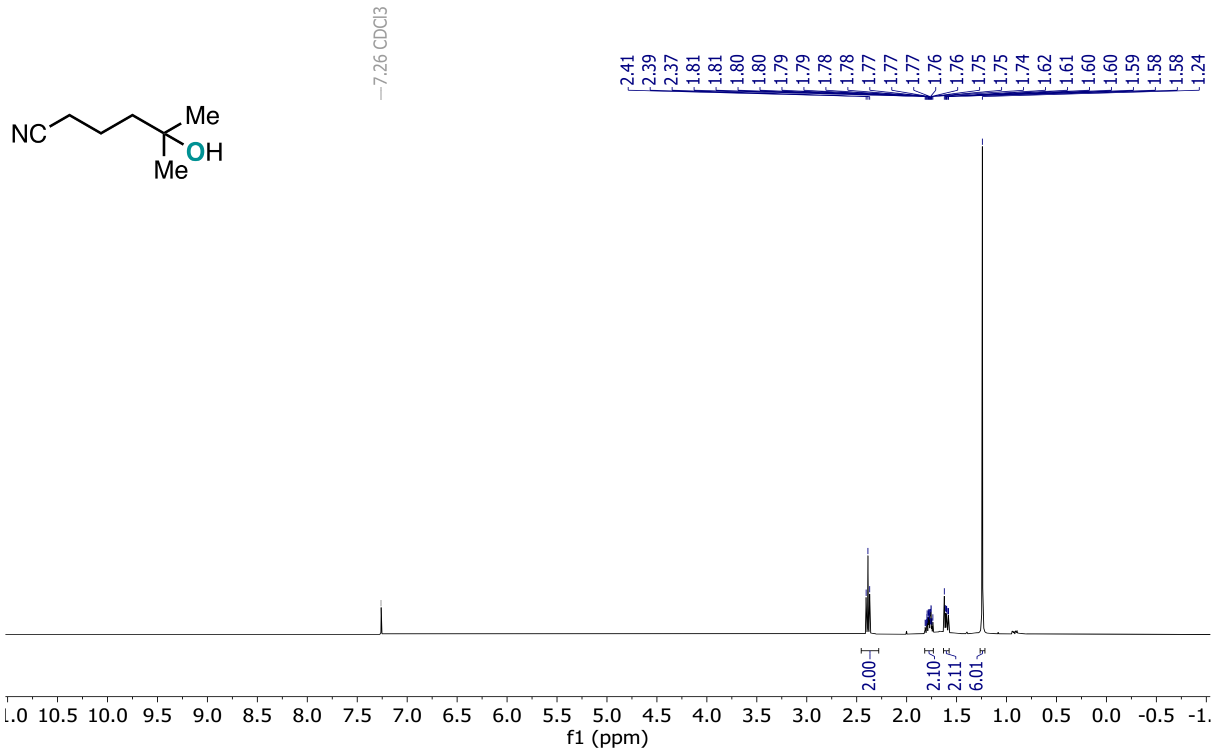

13C NMR (100 MHz, CDCl$_3$) of 5-hydroxy-5-methylhexanenitrile (2aj)

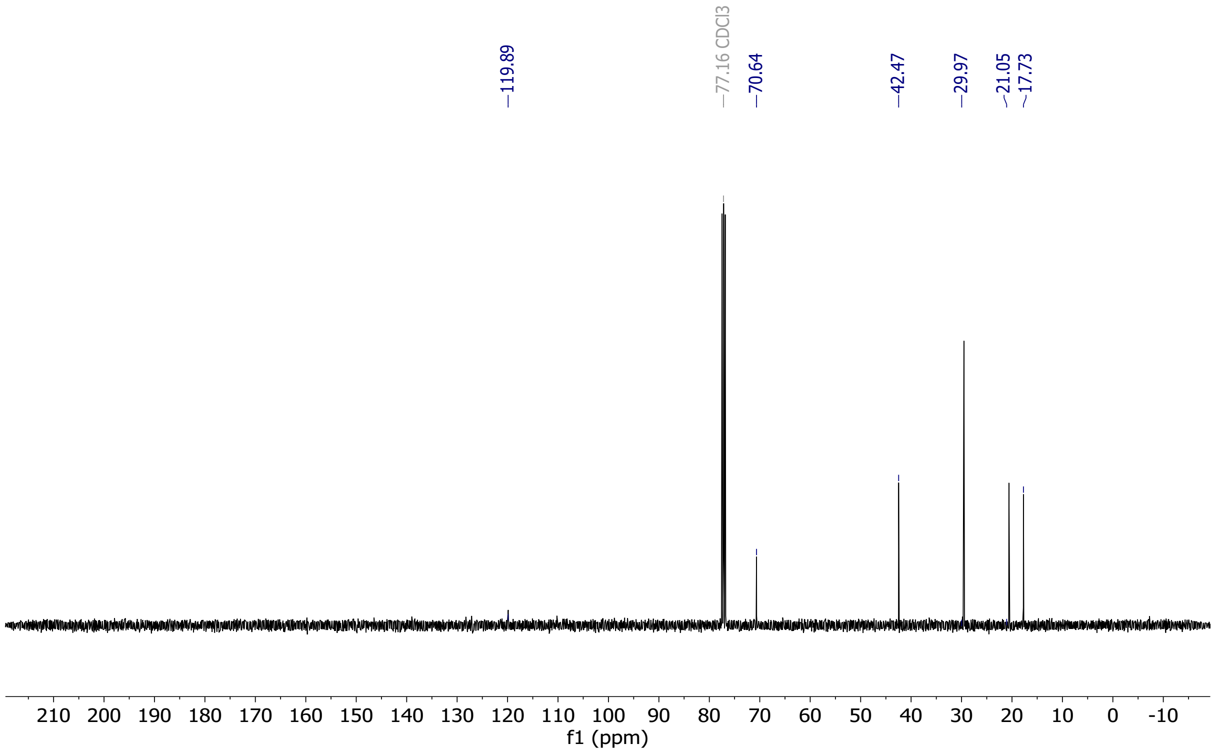

^{1}H NMR (400 MHz, CDCl$_3$) of 2-(4-hydroxy-4-methylpentyl)isoindoline-1,3-dione (2ak)

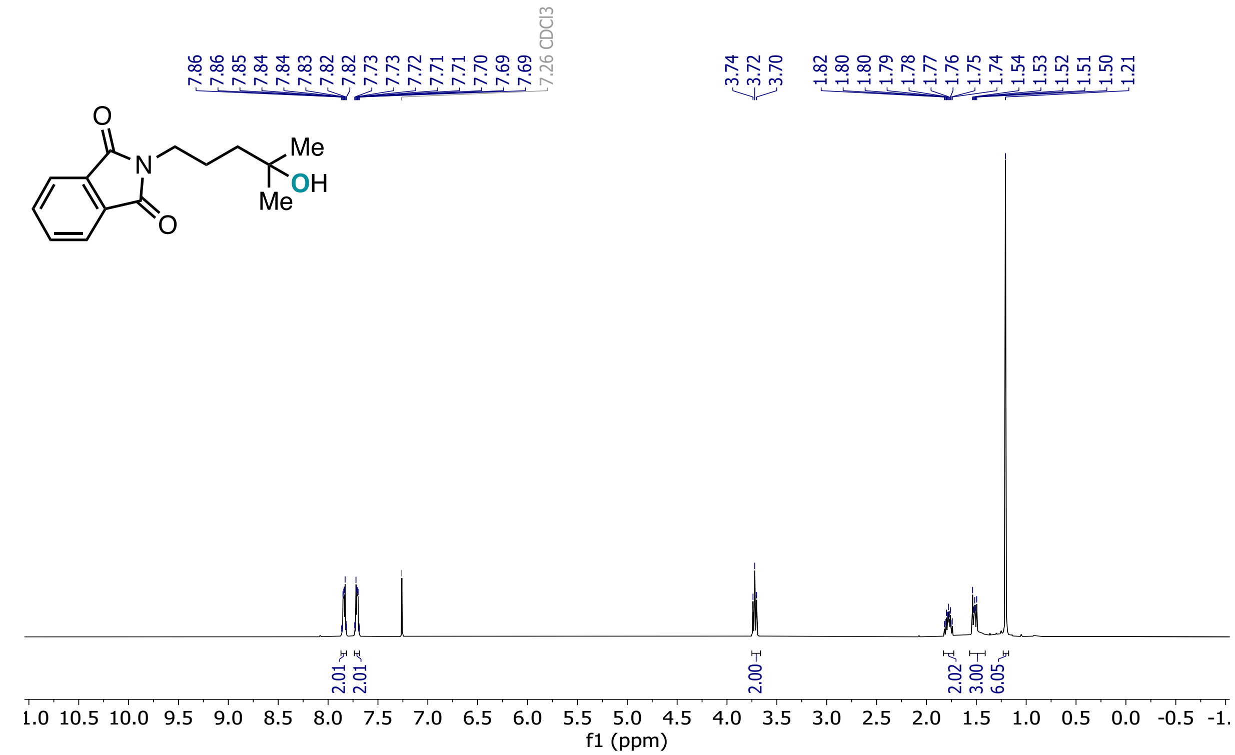

^{13}C NMR (100 MHz, CDCl$_3$) of 2-(4-hydroxy-4-methylpentyl)isoindoline-1,3-dione (2ak)

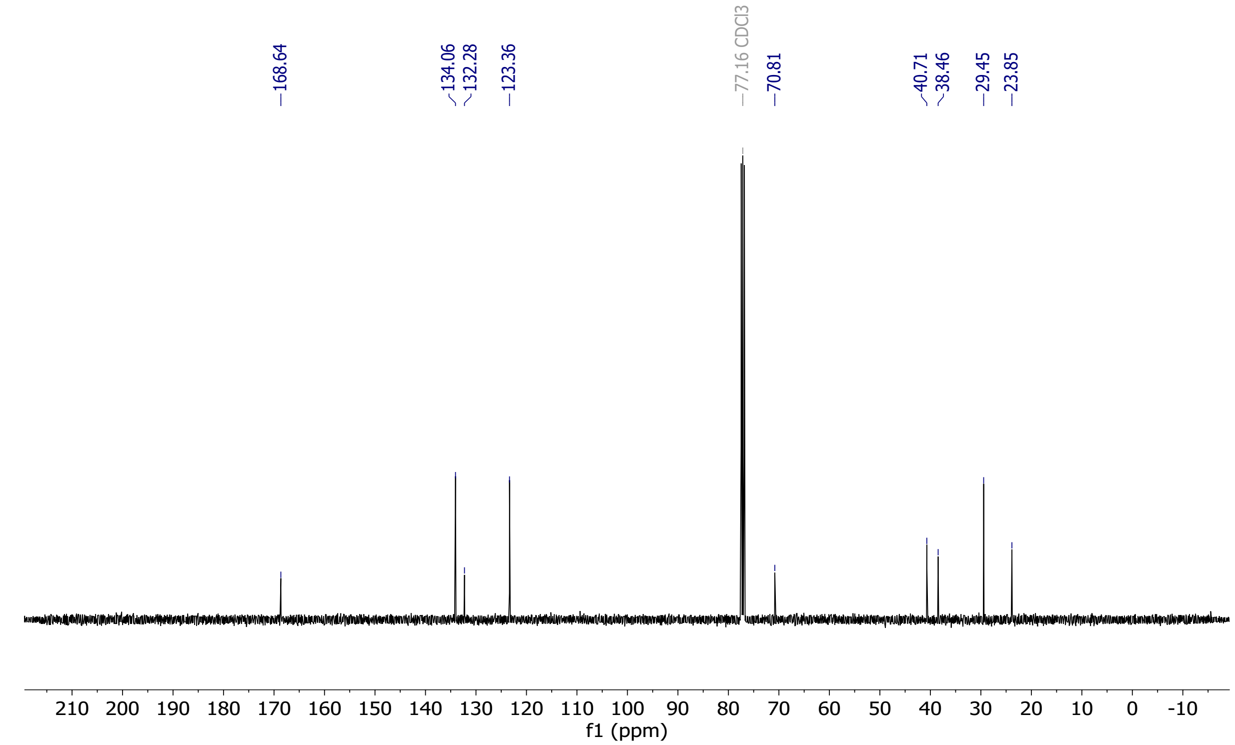

^{1}H NMR (400 MHz, $CDCl_3$) of 2-methyl-6-(phenylsulfonyl)hexan-2-ol (2al)

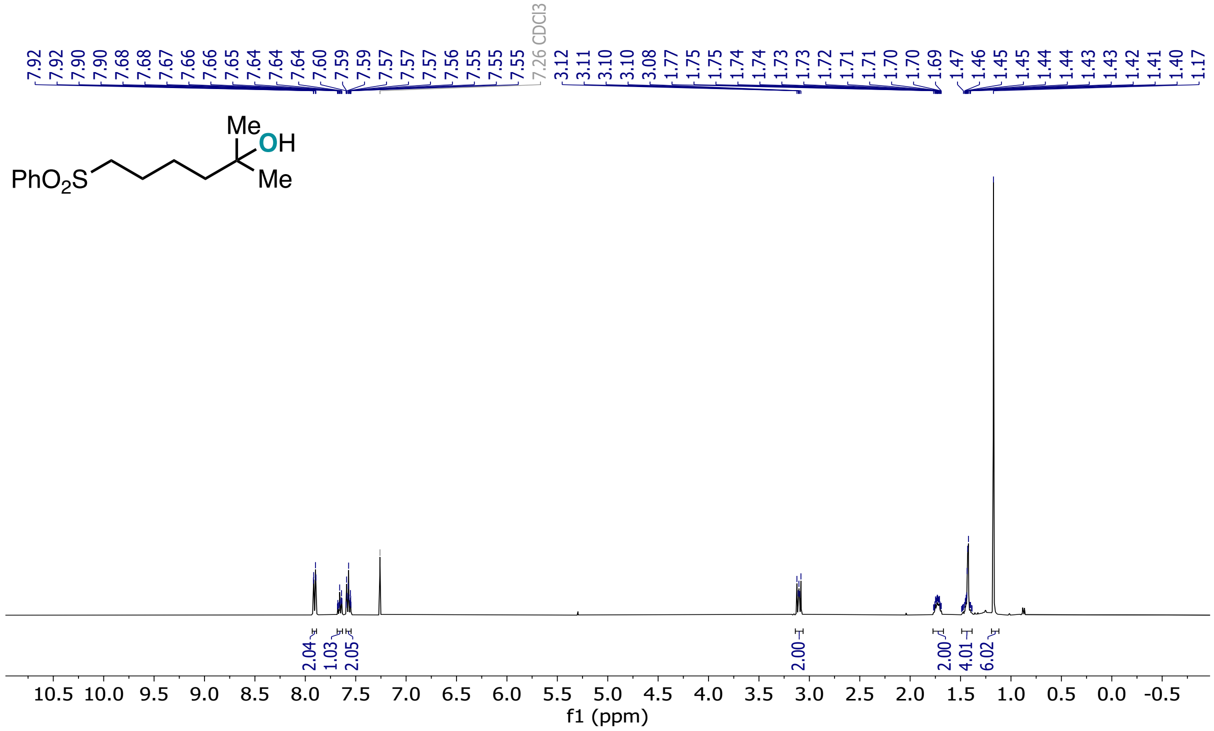

^{13}C NMR (100 MHz, $CDCl_3$) of 2-methyl-6-(phenylsulfonyl)hexan-2-ol (2al)

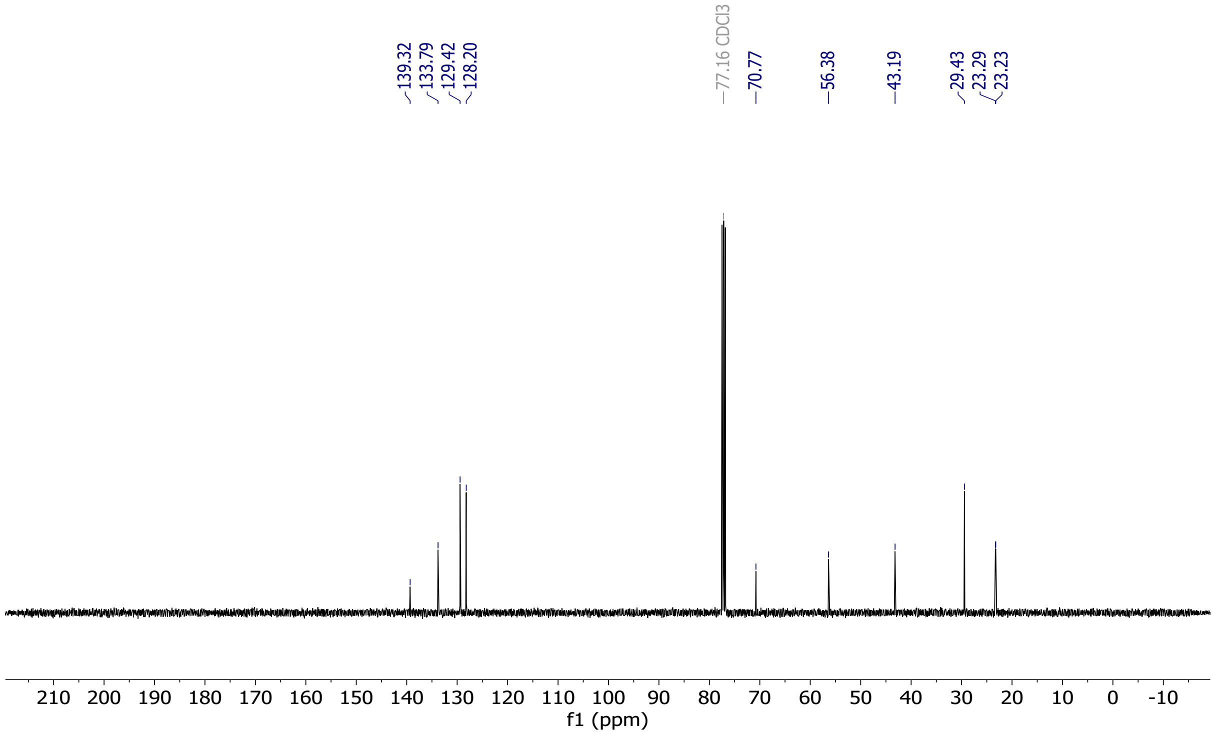

^{1}H NMR (400 MHz, Acetic Acid-d$_4$) of (*R*)-2-(3-hydroxy-3-methylbutyl)-4-oxooxetane-2-carboxylic acid (2am)

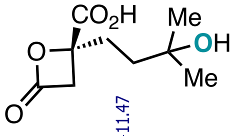

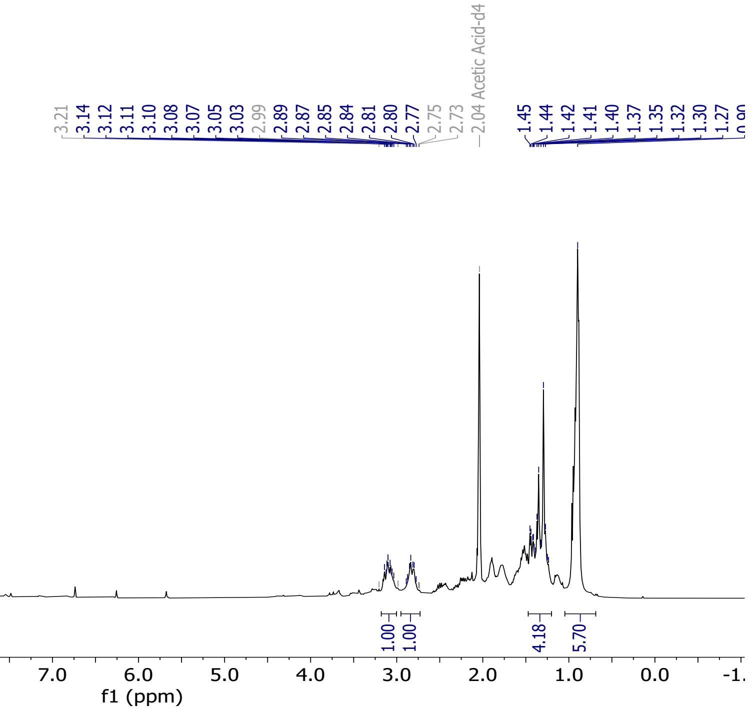

^{13}C NMR (100 MHz, Acetic Acid-d$_4$) of (*R*)-2-(3-hydroxy-3-methylbutyl)-4-oxooxetane-2-carboxylic acid (2am)

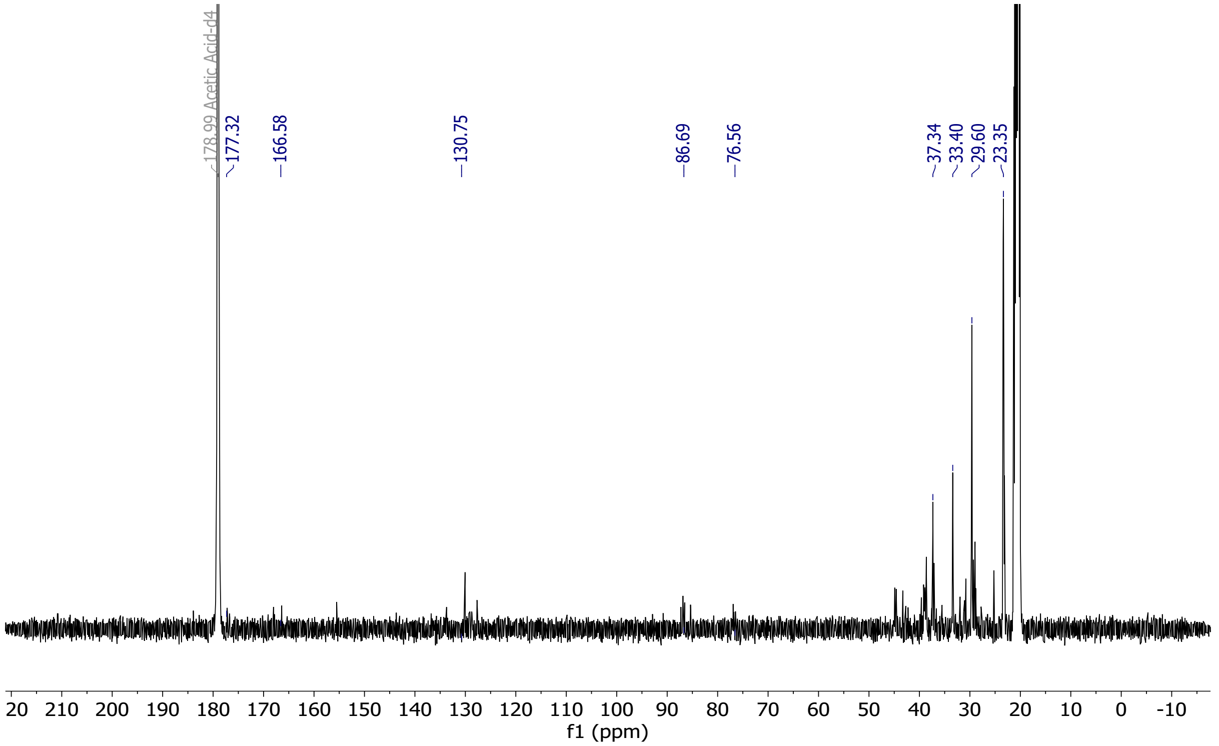

^1H NMR (400 MHz, $CDCl_3$) of bicyclo[2.2.1]heptan-2-ol (2an)

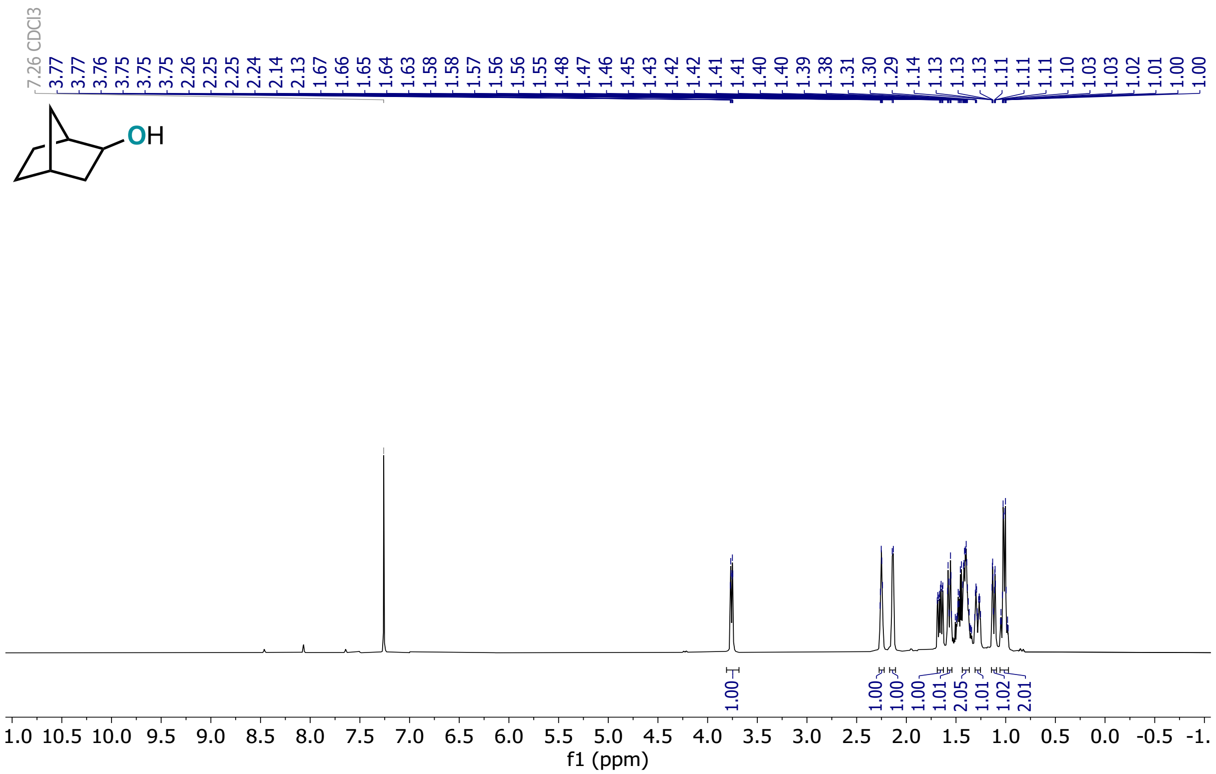

^{13}C NMR (100 MHz, $CDCl_3$) of bicyclo[2.2.1]heptan-2-ol (2an)

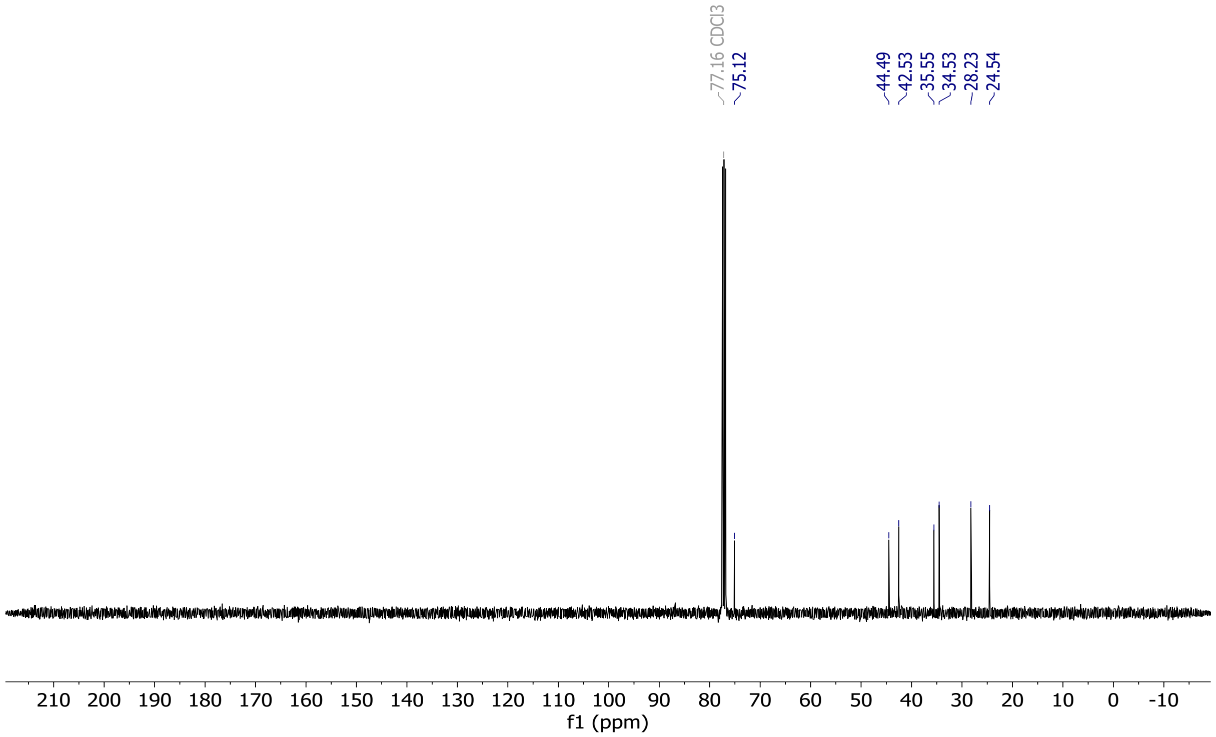

^{1}H NMR (400 MHz, $CDCl_3$) of cyclohexanol (2ao)

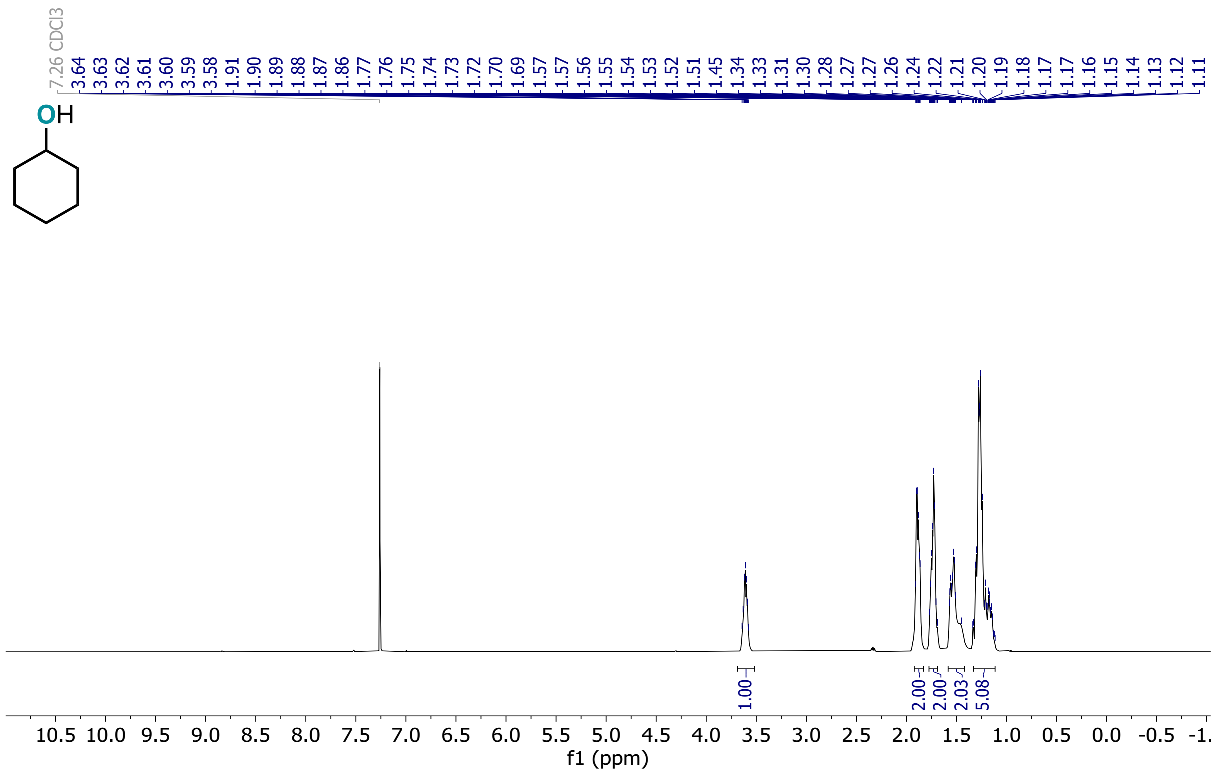

^{13}C NMR (100 MHz, $CDCl_3$) of cyclohexanol (2ao)

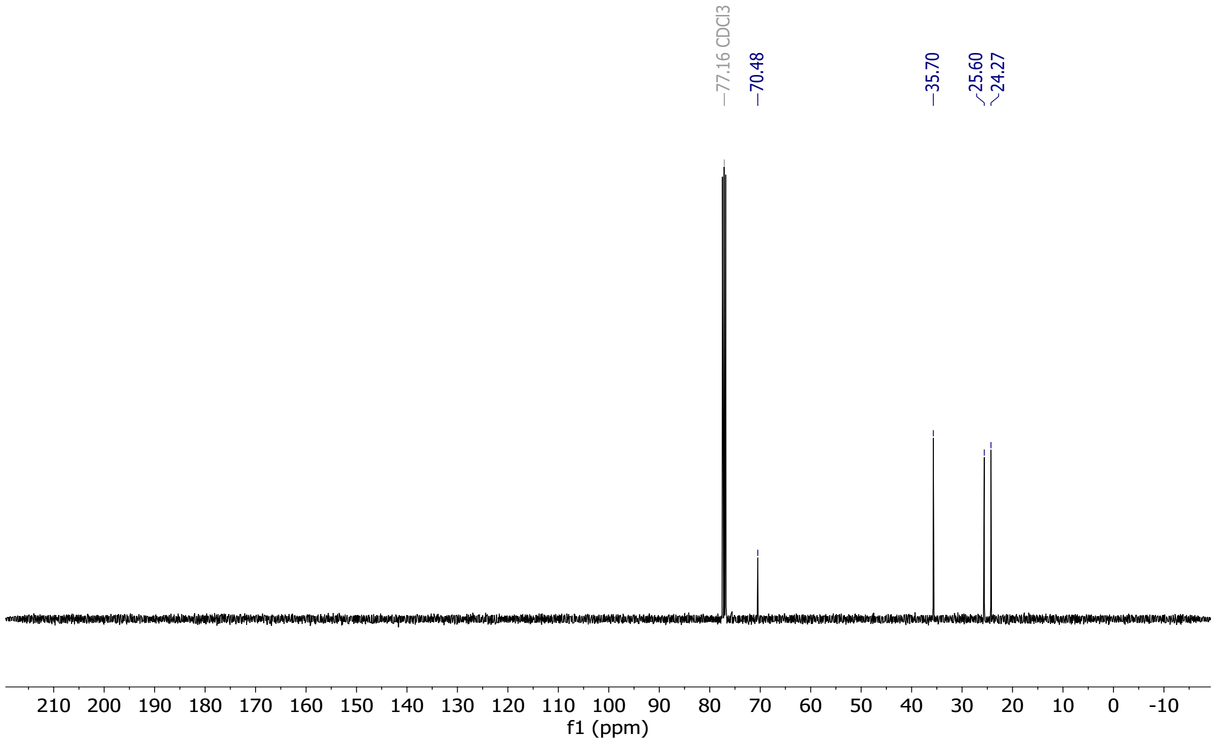

^{1}H NMR (400 MHz, CD_2Cl_2) of Cycloheptanol (2ap)

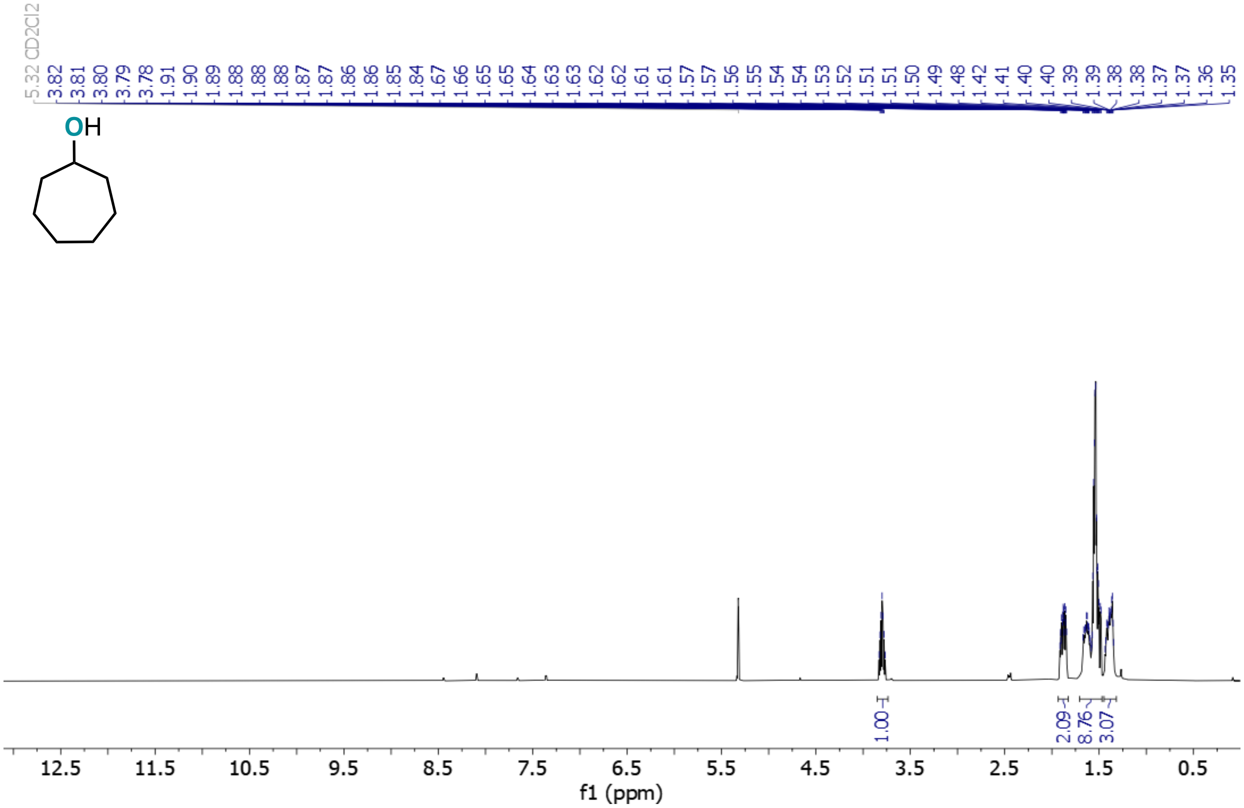

^{13}C NMR (126 MHz, CD_2Cl_2) of Cycloheptanol (2ap)

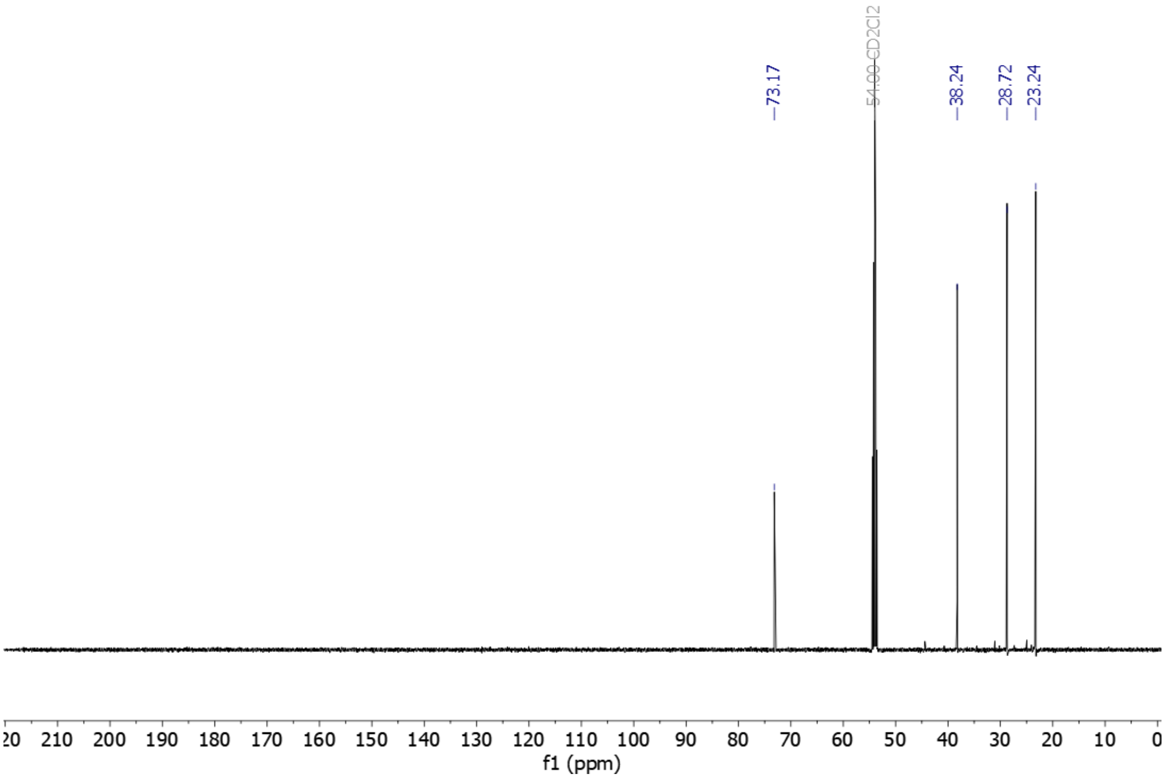

^{1}H NMR (400 MHz, $CDCl_3$) of cyclooctanol (2aq)

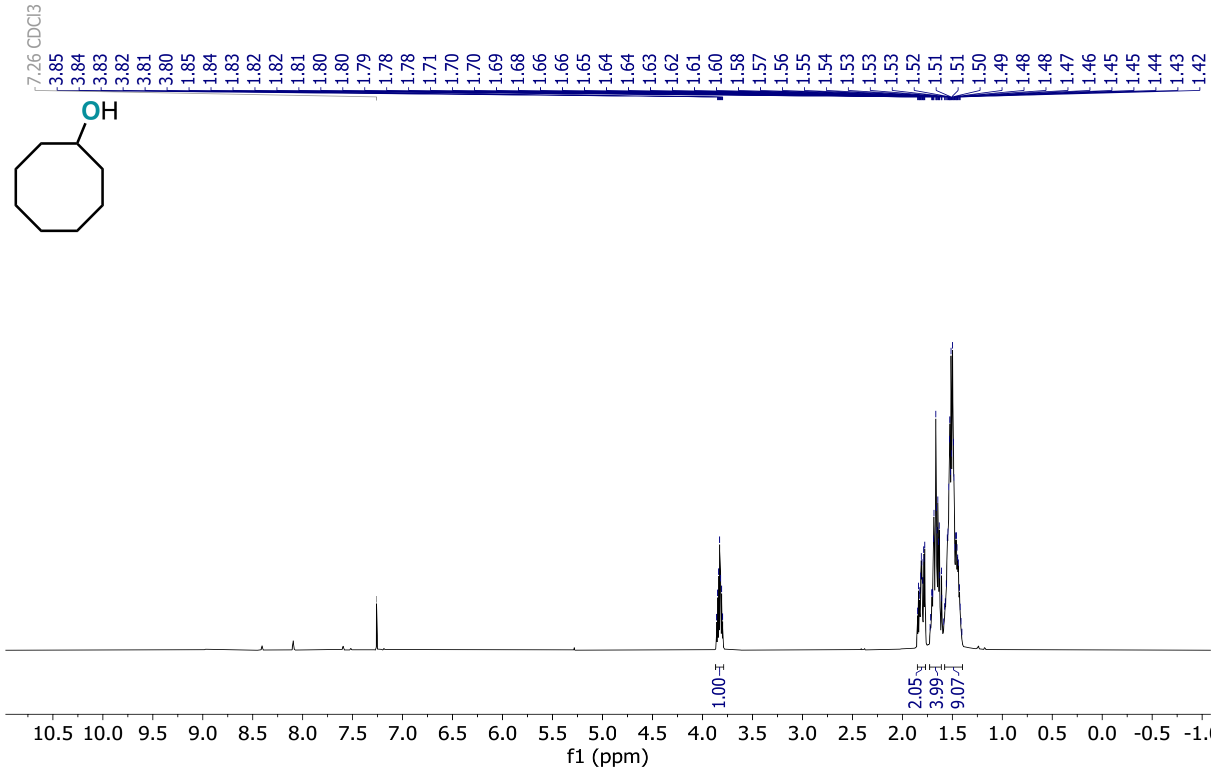

^{13}C NMR (100 MHz, $CDCl_3$) of cyclooctanol (2aq)

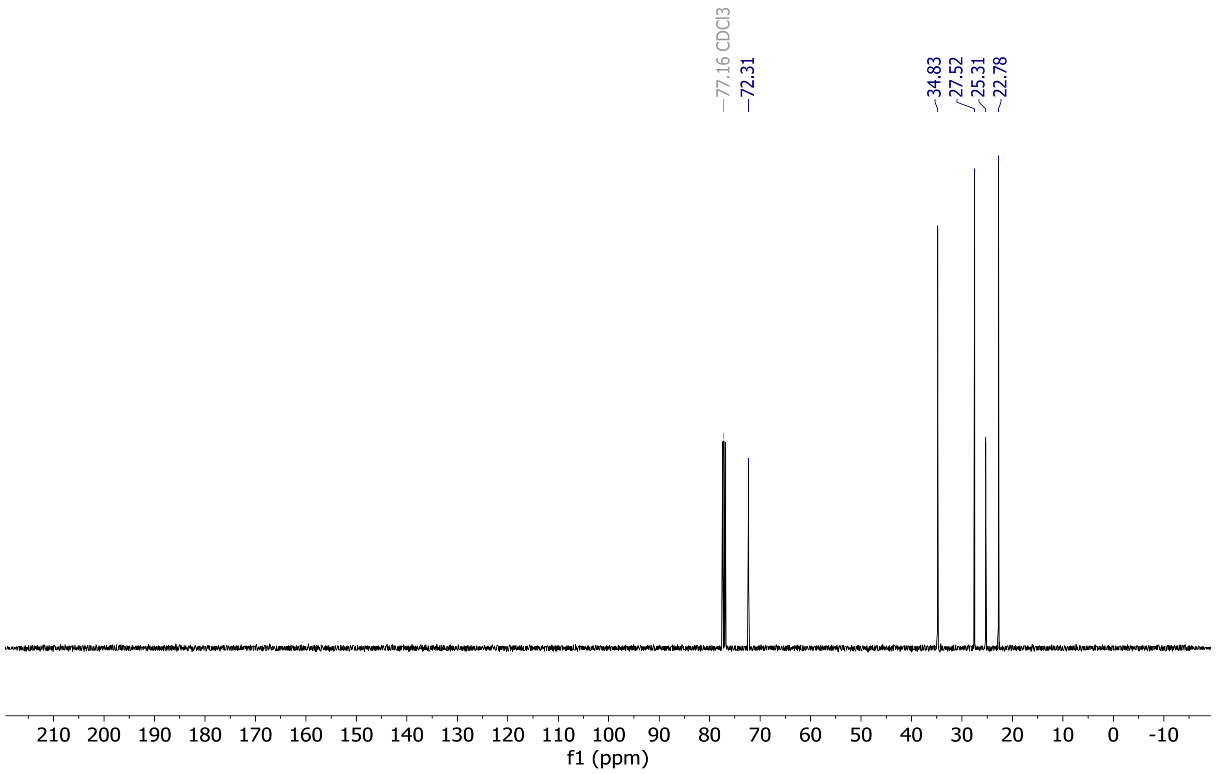

NMR yield spectra

^{1}H NMR (500 MHz, $CDCl_3$) of 2R,3R,4S,5R,6S)-2-(acetoxymethyl)-6-(2-(hydroxymethyl)phenoxy)tetrahydro-2H-pyran-3,4,5-triyl triacetate (2aa)

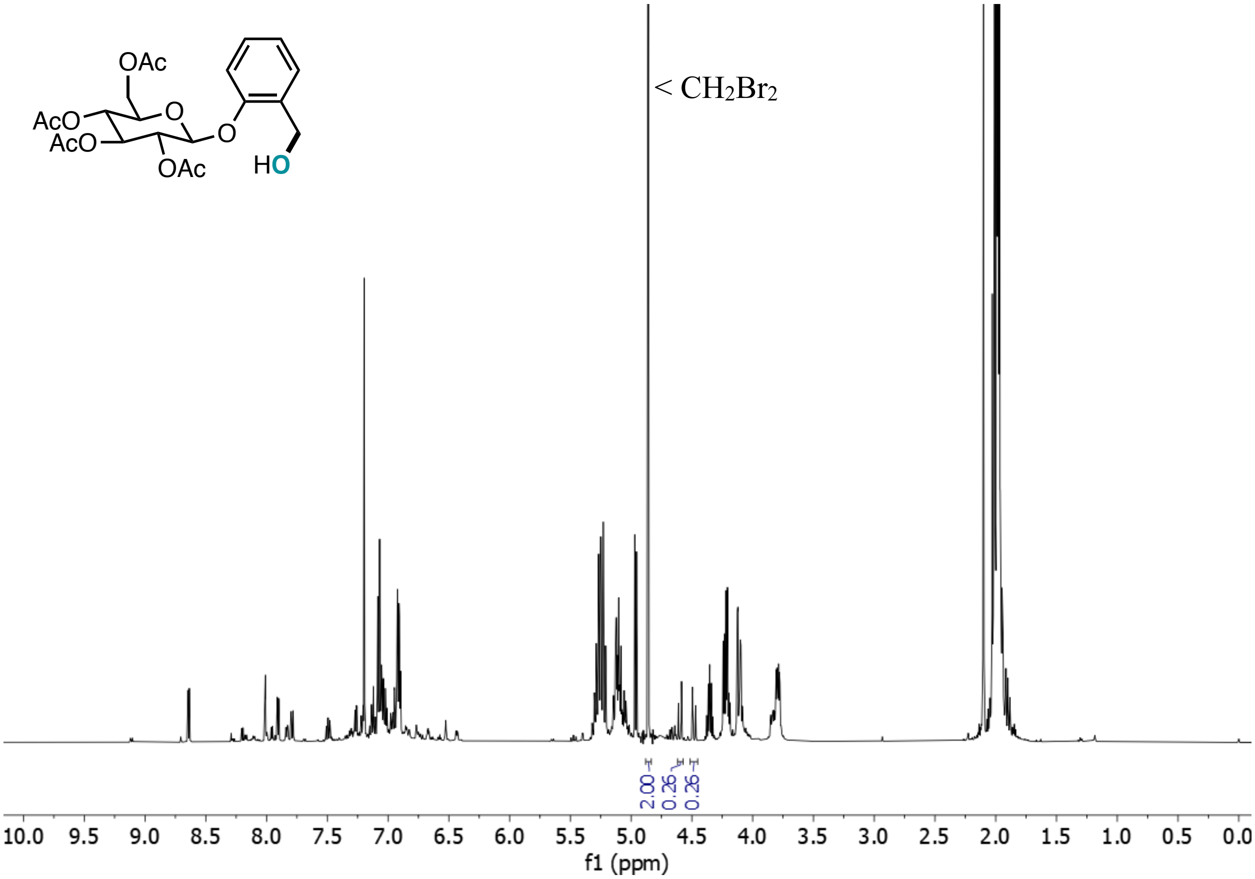

^{1}H NMR (500 MHz, $CDCl_3$) of 2R,3R,4S,5R,6S)-2-(acetoxymethyl)-6-(2-(hydroxymethyl)phenoxy)tetrahydro-2H-pyran-3,4,5-triyl triacetate (2aa) with 1,3,5-trimethoxybenzene as an external standard

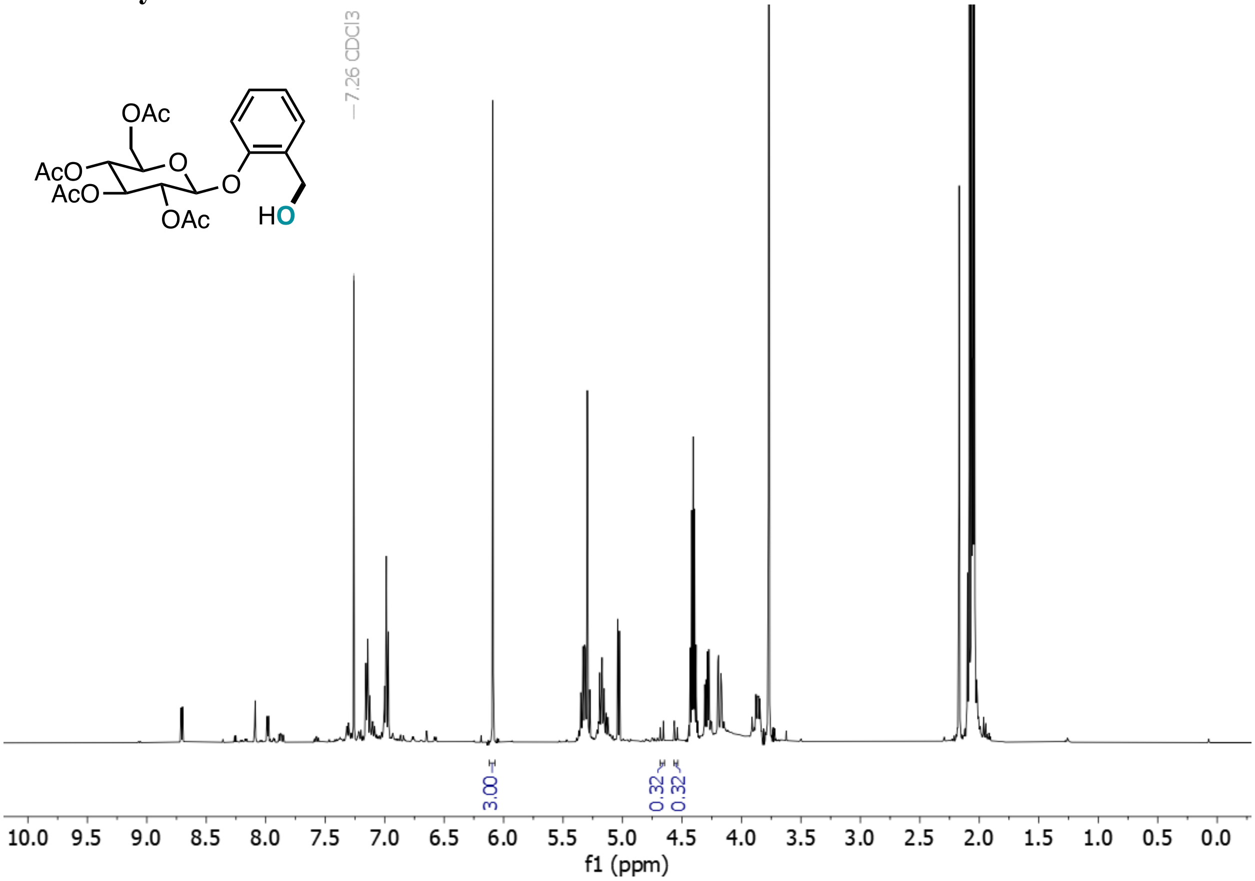

^{1}H NMR (500 MHz, CDCl$_3$) of (8R,9S,13S,14S)-6-hydroxy-13-methyl-17-oxo-7,8,9,11,12,13,14,15,16,17-decahydro-6H-cyclopenta[a]phenanthren-3-yl acetate (2ab)

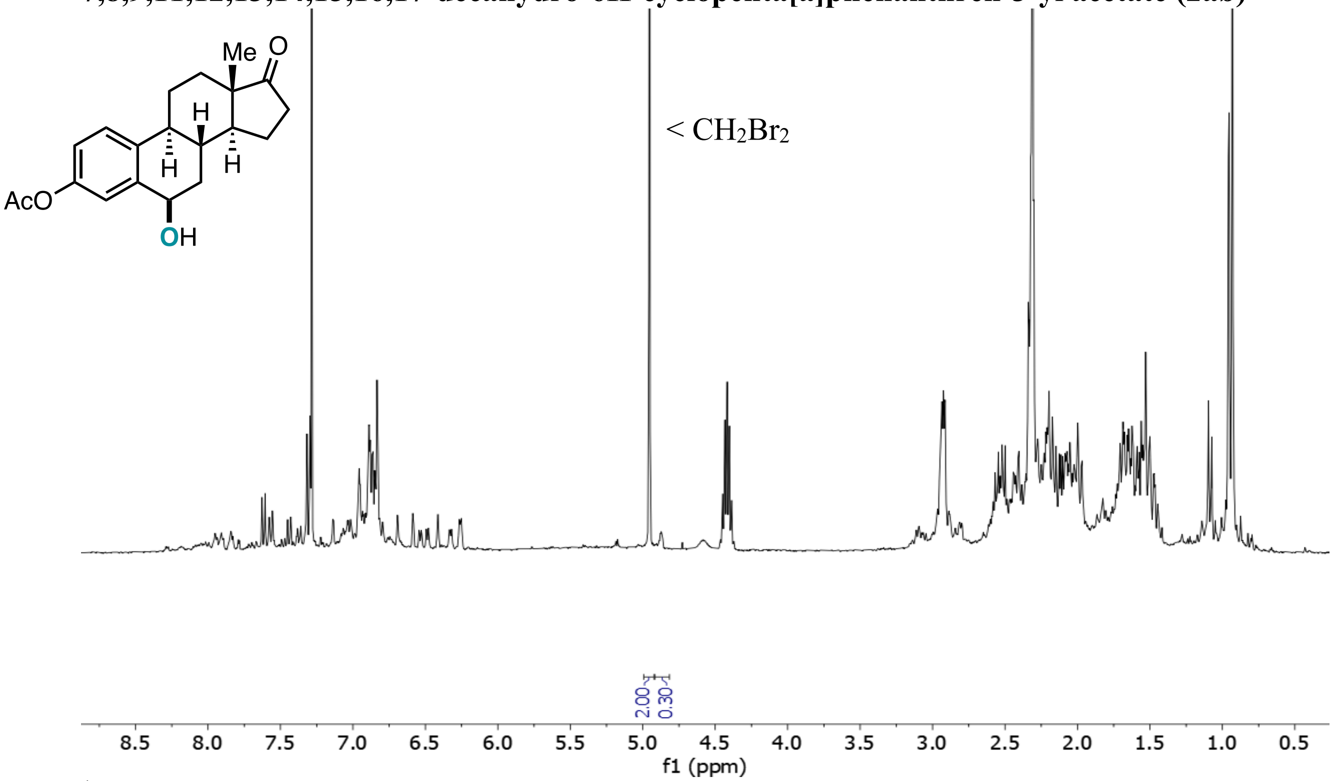

^{1}H NMR (500 MHz, CDCl$_3$) of (2ab) zoomed in from 3.8 ppm – 5.8 ppm

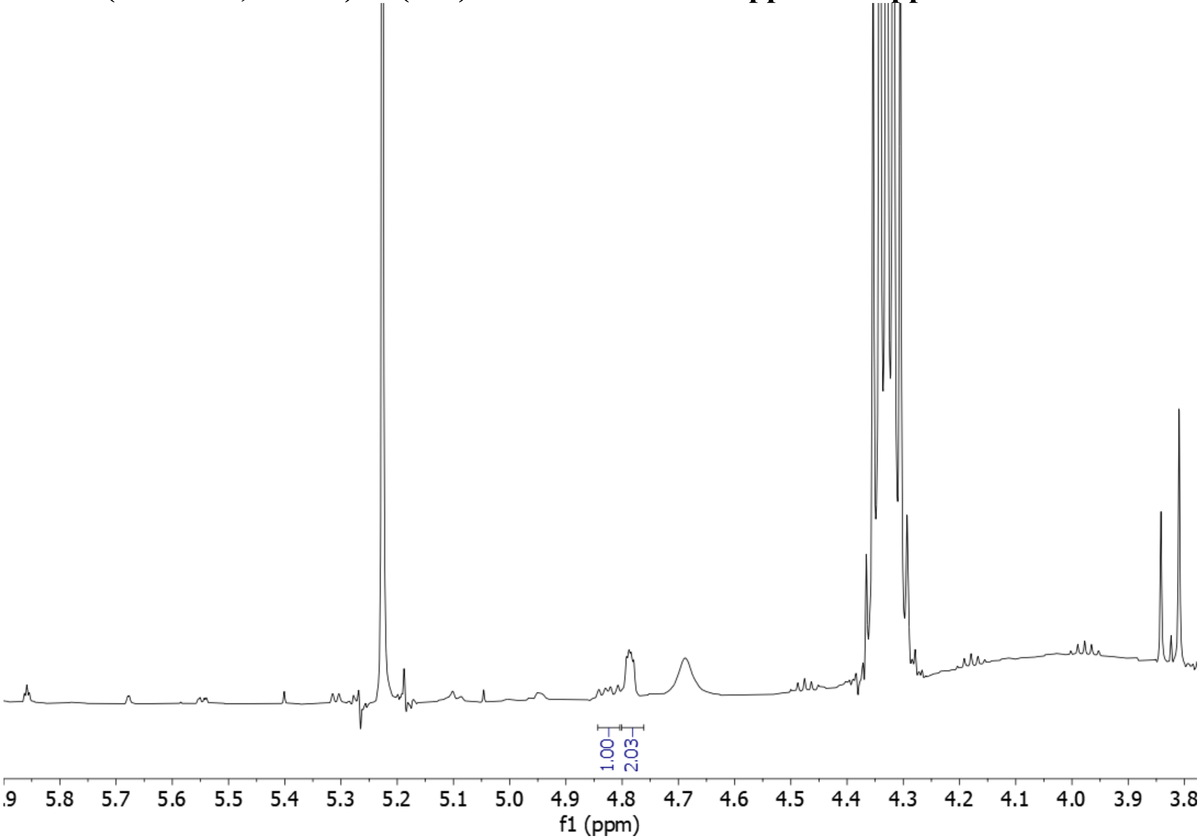

^{1}H NMR (500 MHz, $CDCl_3$) of (8R,9S,13S,14S)-6-hydroxy-13-methyl-17-oxo-7,8,9,11,12,13,14,15,16,17-decahydro-6H-cyclopenta[a]phenanthren-3-yl acetate (2ab) with 1,3,5-trimethoxybenzene as an external standard.

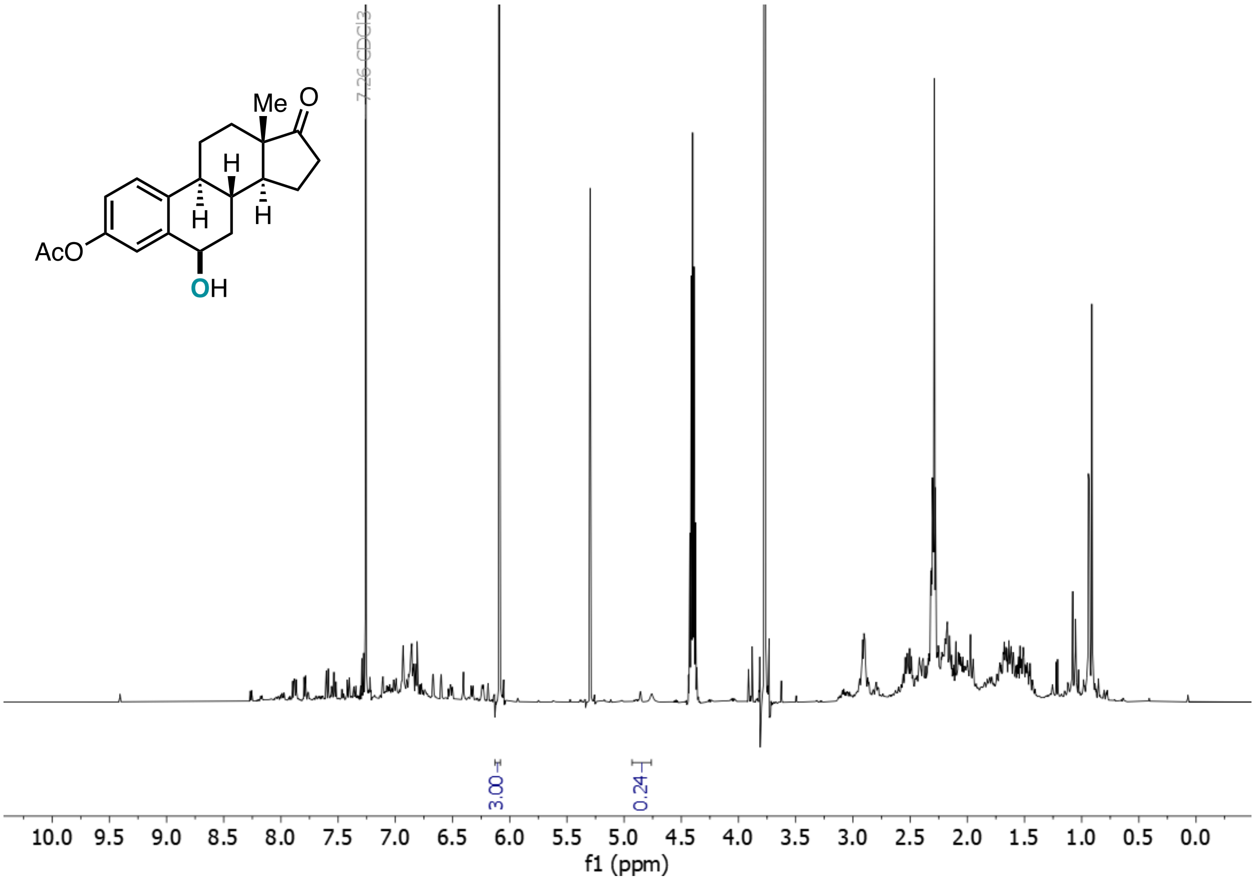

^{1}H NMR (400 MHz, $CDCl_3$) of 2,3-dimethylbutan-2-ol (2ac)

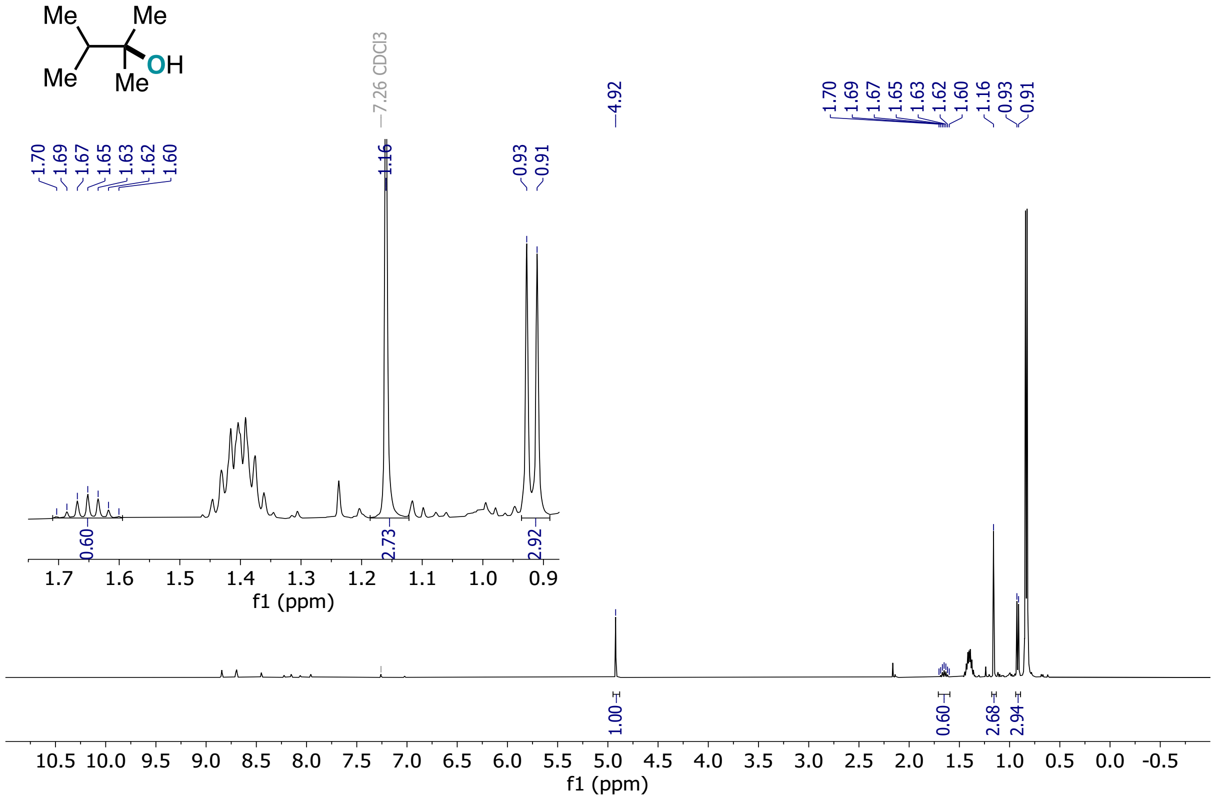

References

1. Wang, Y.; Di, H.; Chen, F.; Xu, Y.; Xiao, Q.; Wang, X.; Wei, H.; Lu, Y.; Zhang, L.; Zhu, J.; Lan, L.; Li, J., Discovery of Benzocycloalkane Derivatives Efficiently Blocking Bacterial Virulence for the Treatment of Methicillin-Resistant S. aureus (MRSA) Infections by Targeting Diapophytoene Desaturase (CrtN). *J. Med. Chem.* **2016,** *59*, 4831-4848.
2. Lim, J.-O.; Jin, M.-K.; Ryu, H.; Kang, D. W.; Lee, J.; Pearce, L. V.; Tran, R.; Toth, A.; Blumberg, P. M., Conformationally constrained analogues of N′-(4-tert-butylbenzyl)-N-(4-methylsulfonylaminobenzyl)thiourea as TRPV1 antagonists. *Eur. J. Med. Chem.* **2009,** *44*, 322-331.
3. Zhang, S.; Li, Y.; Wang, T.; Li, M.; Wen, L.; Guo, W., Electrochemical Benzylic C(sp3)–H Isothiocyanation. *Org. Lett.* **2022,** *24*, 1742-1746.
4. Calow, A. D. J.; Dailler, D.; Bower, J. F., Carbonylative N-Heterocyclization via Nitrogen-Directed C–C Bond Activation of Nonactivated Cyclopropanes. *J. Am. Chem. Soc.* **2022,** *144*, 11069-11074.
5. Jones, C. A. H.; Schley, N. D., Selective demethylation of O-aryl glycosides by iridium-catalyzed hydrosilylation. *Chem. Comm.* **2021,** *57*, 5953-5956.
6. Purushottamachar, P.; Kwegyir-Afful, A. K.; Martin, M. S.; Ramamurthy, V. P.; Ramalingam, S.; Njar, V. C. O., Identification of Novel Steroidal Androgen Receptor Degrading Agents Inspired by Galeterone 3β-Imidazole Carbamate. *ACS Med. Chem. Lett.* **2016,** *7*, 708-713.
7. Baldwin, S. W.; Haut, S. A., Reductive deoxygenation of esters with trichlorosilane. *J. Org. Chem* **1975,** *40*, 3885-3887.
8. Thyer, R.; Robotham, S. A.; Brodbelt, J. S.; Ellington, A. D., Evolving tRNASec for Efficient Canonical Incorporation of Selenocysteine. *J. Am. Chem. Soc* **2015,** *137*, 46-49.
9. Caliskan, G.; Briber, R. M.; Thirumalai, D.; Garcia-Sakai, V.; Woodson, S. A.; Sokolov, A. P., Dynamic Transition in tRNA is Solvent Induced. *J. Am. Chem. Soc* **2006,** *128*, 32-33.
10. Alaux, S.; Kusk, M.; Sagot, E.; Bolte, J.; Jensen, A. A.; Bräuner-Osborne, H.; Gefflaut, T.; Bunch, L., Chemoenzymatic Synthesis of a Series of 4-Substituted Glutamate Analogues and Pharmacological Characterization at Human Glutamate Transporters Subtypes 1−3. *J. Med. Chem.* **2005,** *48*, 7980-7992.
11. Bonaparte, A. C.; Betush, M. P.; Panseri, B. M.; Mastarone, D. J.; Murphy, R. K.; Murphree, S. S., Novel Aerobic Oxidation of Primary Sulfones to Carboxylic Acids. *Org. Lett.* **2011,** *13*, 1447-1449
12. Weber, S.; Stöger, B.; Kirchner, K., Hydrogenation of Nitriles and Ketones Catalyzed by an Air-Stable Bisphosphine Mn(I) Complex. *Org. Lett.* **2018,** *20*, 7212-7215.
13. Chen, S.; Yan, D.; Xue, M.; Hong, Y.; Yao, Y.; Shen, Q., Tris(cyclopentadienyl)lanthanide Complexes as Catalysts for Hydroboration Reaction toward Aldehydes and Ketones. *Org. Lett.* **2017,** *19*, 3382-3385.
14. Sahoo, R. K.; Mahato, M.; Jana, A.; Nembenna, S., Zinc Hydride-Catalyzed Hydrofuntionalization of Ketones. *J. Org. Chem* **2020,** *85,* 11200-11210.
15. Lopez, M. A.; Buss, J. A.; Stahl, S. S., Cu-Catalyzed Site-Selective Benzylic Chlorination Enabling Net C–H Coupling with Oxidatively Sensitive Nucleophiles. *Org. Lett.* **2022,** *24*, 597-601.
16. Thiyagarajan, S.; Gunanathan, C., Ruthenium-Catalyzed Selective Hydrogenation of Epoxides to Secondary Alcohols. *Org. Lett.* **2019,** *21*, 9774-9778.

17. Yasukawa, T.; Kobayashi, S., Oxygenation of Styrenes Catalyzed by N-Doped Carbon Incarcerated Cobalt Nanoparticles. *Bull. Chem. Soc. Jpn.* **2019,** *92*, 1980-1985.
18. Zhu, K.; Shaver, M. P.; Thomas, S. P., Stable and Easily Handled FeIII Catalysts for Hydrosilylation of Ketones and Aldehydes. *Eur. J. Org. Chem.* **2015,** *2015*, 2119-2123.
19. Magre, M.; Paffenholz, E.; Maity, B.; Cavallo, L.; Rueping, M., Regiodivergent Hydroborative Ring Opening of Epoxides via Selective C–O Bond Activation. *J. Am. Chem. Soc.* **2020,** *142*, 14286-14294.
20. Britton, L.; Docherty, J. H.; Nichol, G. S.; Dominey, A. P.; Thomas, S. P., Iron-Catalysed C(sp2)-H Borylation with Expanded Functional Group Tolerance. *Chin. J. Chem.* **2022,** *40*, 2875-2881.
21. Kovalenko, O. O.; Adolfsson, H., Highly Efficient and Chemoselective Zinc-Catalyzed Hydrosilylation of Esters under Mild Conditions. *Chem. Eur. J.* **2015,** *21*, 2785-2788.
22. Laali, K. K.; Herbert, M.; Cushnyr, B.; Bhatt, A.; Terrano, D., Benzylic oxidation of aromatics with cerium(IV) triflate; synthetic scope and mechanistic insight. *J. Chem. Soc. Perkin Trans. 1* **2001,** 6, 578-583.
23. Liu, M.; Zhang, Z.; Song, J.; Liu, S.; Liu, H.; Han, B., Nitrogen Dioxide Catalyzed Aerobic Oxidative Cleavage of C(OH)–C Bonds of Secondary Alcohols to Produce Acids. *Angew. Chem. Int. Ed.***2019,** *58*, 17393-17398.
24. Wang, R.; Tang, Y.; Xu, M.; Meng, C.; Li, F., Transfer Hydrogenation of Aldehydes and Ketones with Isopropanol under Neutral Conditions Catalyzed by a Metal–Ligand Bifunctional Catalyst [Cp*Ir(2,2′-bpyO)(H2O)]. *J. Org. Chem* **2018,** *83*, 2274-2281.
25. Baek, J.; Si, T.; Kim, H. Y.; Oh, K., Bioinspired o-Naphthoquinone-Catalyzed Aerobic Oxidation of Alcohols to Aldehydes and Ketones. *Org. Lett.* **2022,** *24*, 4982-4986.
26. Duhamel, T.; Muñiz, K., Cooperative iodine and photoredox catalysis for direct oxidative lactonization of carboxylic acids. *Chem. Comm.* **2019,** *55*, 933-936.
27. Hosseini, S. N.; Johnston, J. R.; West, F. G., Evidence for heterolytic cleavage of a cyclic oxonium ylide: implications for the mechanism of the Stevens [1,2]-shift. *Chem. Comm.* **2017,** *53*, 12654-12656.
28. Rentmeister, A.; Arnold, F. H.; Fasan, R., Chemo-enzymatic fluorination of unactivated organic compounds. *Nat. Chem. Bio.* **2009,** *5*, 26-28.
29. Spring, S. A.; Goggins, S.; Frost, C. G., Ratiometric electrochemical detection of β-galactosidase. *Org. Biomol. Chem.* **2017,** *15*, 7122-7126.
30. Zhao, L.-M.; Meng, Q.-Y.; Fan, X.-B.; Ye, C.; Li, X.-B.; Chen, B.; Ramamurthy, V.; Tung, C.-H.; Wu, L.-Z., Photocatalysis with Quantum Dots and Visible Light: Selective and Efficient Oxidation of Alcohols to Carbonyl Compounds through a Radical Relay Process in Water. *Angew. Chem. Int. Ed.* **2017,** *56*, 3020-3024.
31. Haslinger, S.; Raba, A.; Cokoja, M.; Pöthig, A.; Kühn, F. E., Iron-catalyzed oxidation of unreactive CH bonds: Utilizing bio-inspired axial ligand modification to increase catalyst stability. *J. Catal.* **2015,** *331*, 147-153.
32. Torigoe, T.; Ohmura, T.; Suginome, M., Utilization of a Trimethylsilyl Group as a Synthetic Equivalent of a Hydroxyl Group via Chemoselective C(sp3)–H Borylation at the Methyl Group on Silicon. *J. Org. Chem* **2017,** *82*, 2943-2956.
33. McNeill, E.; Du Bois, J., Ruthenium-Catalyzed Hydroxylation of Unactivated Tertiary C−H Bonds. *J. Am. Chem. Soc* **2010,** *132*, 10202-10204.
34. Jana, S.; Ghosh, M.; Ambule, M.; Sen Gupta, S., Iron Complex Catalyzed Selective C–H Bond Oxidation with Broad Substrate Scope. *Org. Lett.* **2017,** *19*, 746-749.

35. Martin-Mingot, A.; Compain, G.; Liu, F.; Jouannetaud, M.-P.; Bachmann, C.; Frapper, G.; Thibaudeau, S., Dications in superacid HF/SbF5: When superelectrophilic activation makes possible fluorination and/or C–H bond activation. *J. Fluor. Chem.* **2012,** *134*, 56-62.
36. Howell, J. M.; Feng, K.; Clark, J. R.; Trzepkowski, L. J.; White, M. C., Remote Oxidation of Aliphatic C–H Bonds in Nitrogen-Containing Molecules. *J. Am. Chem. Soc.* **2015,** *137*, 14590-14593.
37. Xie, Y.; Wang, N.; Cheng, B.; Zhai, H., Total Synthesis of (±)-Cephalosol. *Org. Lett.* **2012,** *14*, 3-5.
38. Konev, M. O.; Hanna, L. E.; Jarvo, E. R., Intra- and Intermolecular Nickel-Catalyzed Reductive Cross-Electrophile Coupling Reactions of Benzylic Esters with Aryl Halides. *Angew. Chem. Int. Ed.* **2016,** *55*, 6730-6733.
39. Pitts, C. R.; Ling, B.; Snyder, J. A.; Bragg, A. E.; Lectka, T., Aminofluorination of Cyclopropanes: A Multifold Approach through a Common, Catalytically Generated Intermediate. *J. Am. Chem. Soc.* **2016,** *138*, 6598-6609.
40. Cai, Y.; Zhang, J.-W.; Li, F.; Liu, J.-M.; Shi, S.-L., Nickel/N-Heterocyclic Carbene Complex-Catalyzed Enantioselective Redox-Neutral Coupling of Benzyl Alcohols and Alkynes to Allylic Alcohols. *ACS Catal.* **2019,** *9*, 1-6.
41. Powers, I. G.; Andjaba, J. M.; Luo, X.; Mei, J.; Uyeda, C., Catalytic Azoarene Synthesis from Aryl Azides Enabled by a Dinuclear Ni Complex. *J. Am. Chem. Soc.* **2018,** *140*, 4110-4118.
42. Gomez, Laura; et al *Angew. Chem., Int. Ed.* **2009**, *48*, 5720-5723.
43. McNeill, E.; Du Bois, J., Ruthenium-Catalyzed Hydroxylation of Unactivated Tertiary C−H Bonds. *J. Am. Chem. Soc.* **2010**, *132*, 10202–10204.
44. Antoniotti, S.; Fernandez, X.; Duñach, E., Reaction design for evaluation of the substrate range of hydrolases. *Biocatal. Biotransformation.* **2008**, 26, 228-234.
45. Karlsson, S.; Hallberg, A.; Gronowitz, S., Hydrozirconation of (E)-3-methoxy-1-phenyl-1-propene and (E)-3-phenyl-2propenol. *J. Organomet. Chem.*, **1990**, *403*, 133-144.
